# Agronomic, Physiological and Genetic Changes Associated With Evolution, Migration and Modern Breeding in Durum Wheat

**DOI:** 10.3389/fpls.2021.674470

**Published:** 2021-07-08

**Authors:** Conxita Royo, Karim Ammar, Dolors Villegas, Jose M. Soriano

**Affiliations:** ^1^Sustainable Field Crops Programme, Institute for Food and Agricultural Research and Technology (IRTA), Lleida, Spain; ^2^International Maize and Wheat Improvement Center (CIMMYT), Texcoco, Mexico

**Keywords:** genetic structure, association mapping, hotspots, haplotype blocks, yield components

## Abstract

A panel of 172 Mediterranean durum wheat landraces and 200 modern cultivars was phenotyped during three years for 21 agronomic and physiological traits and genotyped with 46,161 DArTseq markers. Modern cultivars showed greater yield, number of grains per spike (NGS) and harvest index (HI), but similar number of spikes per unit area (NS) and grain weight than the landraces. Modern cultivars had earlier heading but longer heading-anthesis and grain-filling periods than the landraces. They had greater RUE (Radiation Use Efficiency) up to anthesis and lower canopy temperature at anthesis than the landraces, but the opposite was true during the grain-filling period. Landraces produced more biomass at both anthesis and maturity. The 120 genotypes with a membership coefficient *q* > 0.8 to the five genetic subpopulations (SP) that structured the panel were related with the geographic distribution and evolutionary history of durum wheat. SP1 included landraces from eastern countries, the domestication region of the “Fertile Crescent.” SP2 and SP3 consisted of landraces from the north and the south Mediterranean shores, where durum wheat spread during its migration westward. Decreases in NS, grain-filling duration and HI, but increases in early soil coverage, days to heading, biomass at anthesis, grain-filling rate, plant height and peduncle length occurred during this migration. SP4 grouped modern cultivars gathering the CIMMYT/ICARDA genetic background, and SP5 contained modern north-American cultivars. SP4 was agronomically distant from the landraces, but SP5 was genetically and agronomically close to SP1. GWAS identified 2,046 marker-trait associations (MTA) and 144 QTL hotspots integrating 1,927 MTAs. Thirty-nine haplotype blocks (HB) with allelic differences among SPs and associated with 16 agronomic traits were identified within 13 QTL hotspots. Alleles in chromosomes 5A and 7A detected in landraces were associated with decreased yield. The late heading and short grain-filling period of SP2 and SP3 were associated with a hotspot on chromosome 7B. The heavy grains of SP3 were associated with hotspots on chromosomes 2A and 7A. The greater NGS and HI of modern cultivars were associated with allelic variants on chromosome 7A. A hotspot on chromosome 3A was associated with the high NGS, earliness and short stature of SP4.

## Introduction

Durum wheat (*Triticum turgidum* ssp. *durum*) is an important cereal crop grown on around 17 million ha worldwide with a global production of 33.6 million tons in 2020 (http://www.agr.gc.ca/eng/industry-markets-and-trade/canadian-agri-food-sector-intelligence/crops/reports-and-statistics-data-for-canadian-principal-field-crops/?id=1378743094676). Its production is concentrated in the variable, often low rainfall regions of the Mediterranean basin and in the northern plains of Canada and the United States (https://ec.europa.eu/eurostat/data/database). The Mediterranean Basin embraces countries between 27° and 47°N and between 10°W and 37°E extending over three continents and a coastline of 46,000 km (Royo et al., 2017). The region produces close to half of the world durum crop but remains the largest importer and the largest consumer of the grain, mostly in form of pasta and couscous, but also as a number of other semolina products such as frike, bourghul, and unleavened breads.

Wheat was domesticated ~10,000 years BP in the mountainous areas of southwest Asia and Iran, Turkey, Syria, Lebanon, Israel, Palestine and Jordan, in the region often referred to as the “Fertile Crescent” (Vavilov, 1951; Harlan, 1992). From this area, wheat spread to the west of the Mediterranean Basin about 3,000 years ago to finally reach the Iberian Peninsula (Feldman, 2001; MacKey, 2005). The dynamic environmental conditions occurring during wheat migration westward from its center of origin facilitated its spread and induced evolutionary changes in the original types (Harlan, 1992; Zeven, 1998). The combination of natural selection for traits that augment fitness to the diverse agro-ecological zones existing within the Mediterranean Basin (Cleveland and Soleri, 2007), and farmer-mediated selection for traits deemed of interest resulted in the local establishment of landraces. According to Camacho-Villa et al. (2005) a landrace is “*a dynamic population of a cultivated plant that has historical origin, distinct identity, and lacks formal crop improvement, as well as often being genetically diverse, locally adapted, and associated with traditional farming systems*.” The prevalent environmental conditions of the regions colonized by durum wheat landraces have driven their productive strategy and resulted in the selection of adaptive advantages to the different territories (Moragues et al., 2006a). The diversity of landraces has been related mainly to their geographical origin, as evidenced by their local adaptation to their areas of localized spread (Lopes et al., 2015). A relationship has been established between growth and development and yield formation characteristics of Mediterranean durum wheat landraces and the climate of the areas where they are endemic (Moragues et al., 2006a,b; Royo et al., 2014). Mediterranean landraces hold a rich genetic diversity for many traits, including for those which are considered economically important in today's agricultural production systems, which makes them invaluable sources of biodiversity within the species (Nazco et al., 2012; Lopes et al., 2015).

Landraces, as such, represented the totality of commercial crops up to the first half of the twentieth century, before they were progressively abandoned in favor of more productive types. First, original landraces were replaced by superior selections made within them. From the early 1970's they were massively displaced from farmer fields by the semi-dwarf types of the “Green Revolution” bred and selected by breeders around the world for a wide range of characteristics providing more adaptation to more intensive commercial production systems (Royo et al., 2009). In the general case of wheat, and in particular in the case of durum wheat, the primary breeding event that occurred during the “Green Revolution” consisted of the use and dissemination of the semi-dwarf gene *Rht-B1b* originally transferred from the bread wheat Japanese variety “Norin10” (Autrique et al., 1996). This gene increased earliness, reduced plant height without substantial decreases in total plant dry weight and dramatically improved the harvest index (McCaig and Clarke, 1995; De Vita et al., 2007; Royo et al., 2007), drastically transforming the wheat plant to produce more grain per unit of biomass. This change in plant architecture made the wheat plant much more competitive in modern agriculture production systems. By making the plant shorter, less susceptible to lodging and more responsive to input (water and fertilizer), dwarfing genes also provided a unique opportunity for crop intensification, especially in irrigated or high rainfall environments.

However, it has been suggested that the genetic diversity remaining after the dramatic displacement of landraces by modern cultivars may have been significantly reduced, partly due to the use of a reduced number of ancestors in modern breeding programs. A study including 51 cultivars derived from the CIMMYT/ICARDA (International Maize and Wheat Improvement Center/International Center for Agricultural Research in the Dry Areas) breeding programs found that 15 ancestors contributed to 72% of the genetic makeup of improved cultivars (Autrique et al., 1996). Although many public and private institutions around the world have contributed to the genetic improvement of durum wheat, the germplasm developed by CIMMYT and ICARDA has had an unprecedented global impact (Royo et al., 2010; Lantican et al., 2016). In fact, among recently cultivated spring durum wheats, the CIMMYT germplasm pool has emerged as one of the three most representative according to its pattern of adaptability and geographical distribution (Royo et al., 2009).

Traditionally, with few exceptions, landraces have not been essential nor regular components of the crossing strategies in breeding programs due to their unfavorable agronomic characteristics and the need to conduct specific backcrossing steps and selection in large segregating populations to identify progenies without these detrimental characteristics or unfavorable alleles. However, in the current context of rapidly mutating pathogen populations and weather conditions becoming increasingly warmer, landraces can be viewed as a reservoir of largely unused genetic diversity, that can be exploited to broaden the genetic bases of disease resistance (Aoun et al., 2016; Kthiri et al., 2019) or for enhanced adaptation to erratic and suboptimal environmental conditions. Landraces have also been shown to harbor considerable allelic variation for High Molecular Weight Glutenin (HMWG) subunits contrary to the modern germplasm which is characterized by a drastically limited variation at these loci controlling gluten strength (Nazco et al., 2012, 2014a,b). Given the relatively limited variation for grain micro-nutrient (iron and zinc primarily) within the modern durum germplasm (Magallanes-Lopez et al., 2017) and the wide range of micro-nutrients observed in landraces (Sciacca et al., 2018; Hernandez-Espinosa et al., 2020), these can be choice donors for breeding programs involved in raising the nutritional value of the durum grain.

Exploiting such useful genetic variability will require a breeding scheme that would allow the selection of useful or favorable traits/alleles from a landrace while ensuring the selection against all associated performance-inhibiting traits/alleles. Preliminary studies have been conducted to that end. Soriano et al. (2017) identified molecular marker-trait associations in Mediterranean landraces for yield components, phenology and biomass. A further study identified 23 marker alleles with a differential frequency in landraces from east and west regions of the Mediterranean Basin, which affected important agronomic traits (Soriano et al., 2018). More recently, a GWAS approach employing eigenvectors has been used to identify 89 selective sweeps, represented as QTL hotspots, among landraces and modern cultivars and to quantify gene flow among them (Soriano et al., 2021).

In the present study, we have used a panel of 172 Mediterranean landraces from 21 countries and 200 modern cultivars of diverse origin, previously structured in five genetic subpopulations by Soriano et al. (2021), to: (i) document important quantitative agronomic and physiological attributes differentiating landraces from modern cultivars, (ii) explore the relationship between the genetic structure and the agronomic performance of the accessions included in the collection, and (iii) identify genomic regions controlling agronomic performance that differentiate between the main germplasm pools identified in the panel.

## Materials and Methods

### Plant Material

We tested a panel of 372 durum wheat genotypes including 172 landraces from 21 Mediterranean countries and 200 modern cultivars of diverse origins (Supplementary Table 1). The aim was to sample a large portion of the unexplored genetic diversity of ancient durums from the Mediterranean Basin and a representative fraction of the major spring durum wheat germplasm pools presently cultivated. The landraces were selected based on their genetic variability from a larger panel of accessions provided by public gene banks (Centro de Recursos Fitogenéticos INIA-Spain, ICARDA Germplasm Bank and USDA Germplasm Bank). The landraces were bulk-purified, always selecting the dominant type (usually with a frequency above 80% of the bulk), and the seed was increased ensuring a common seed origin for all lines. All landraces except two of them (cultivars “Carlantino” and “Verdiel”) had spring growth habit (Royo et al., 2020). The genetic structure of the panel used has been recently described by Soriano et al. (2021) using 46,161 DArTseq markers. A first classification, related to the main historical breeding periods, separated landraces and modern cultivars, but a further analysis structured the collection in five subpopulations (SP) (Supplementary Table 1). To address the second objective of the current study the 120 genotypes with a membership coefficient *q* > 0.80 to these subpopulations were used.

### Plant Phenotyping

Field experiments were conducted during the 2013, 2014, and 2015 seasons in Lleida (41°40'N, 0°20'E, 260 m.a.s.l.), north-eastern Spain. The experiments were carried out in a non-replicated modified augmented design with two replicated checks (cultivars “Avispa” and “Euroduro”) at a ratio of 1:5 between checks and tested genotypes. The plots measured 3.6 m^2^ and comprised eight rows spaced 0.15 m apart. Sowing density was adjusted to 250 viable seeds m^−2^. Planting dates were 4-December 2012, 27-November 2013 and 21-November 2014. Average minimum and maximum monthly temperatures and rainfall were calculated from daily data recorded for a weather station close to the experimental fields. Soil moisture was monitored in one of the repeated checks from the seedling stage by means of soil probes (model EC-20, ECH2O Dielectric Aquameter, Decagon Devices, Inc.) located at three depths (0–10, 10–25 and 25–40 cm) (Supplementary Figure 1). Given the organic matter content of the soil (2.7%) and in order to avoid the lodging of landraces, nitrogen fertilization was avoided and at seed bed 75 u.P_2_O_5_ and 125 u.K_2_O were applied. Arthropod pests were controlled with Chlorpyrifos 48% (0.2 l/ha), Deltamethrin 2.5% (3 l/ha) and Esfenvalerate 5% (0.3 l/ha). For disease control Trifloystrobin 37.5% + Cyproconazole 16% (0.35 l/ha) and Epoxiconazole 12.5% (1 l/ha) were used. Weeds were controlled with Ioxynil 7.5% + Bromoxynil 7.5% + MCPP 37.5% (3 l/ha) and Fluroxypyr 20% (1 l/ha).

Plants from each plot were monitored on a twice-weekly basis to record the following growth stages (Zadoks et al., 1974): GS11 (first leaf emerged), GS55 (heading), GS65 (anthesis) and GS87 (physiological maturity). A plot was considered to have reached a given developmental stage when ~50% of the plants exhibited the stage-specific phenotypic characteristics. From this data the number of days from emergence to heading (DEH), days from heading to anthesis (DHA) and days from anthesis to maturity (DAM) were determined. Aboveground dry matter (DM, g m^−2^) was determined at GS65 by weighing samples from a 0.5-m-long section of a central row from each plot after oven-drying them at 70°C for 48 h. Before plot harvesting, samples from a 1-m-long section were pulled from a central row of each plot and weighed, and the number of spikes per m^2^ (NS), grains per spike (NGS) and the weight of grains in the sample were recorded on a whole sample basis. Harvest index (HI) was computed by dividing the grain weight by the total weight of the plants contained in 1-m-long row sample. Plant height (PH, cm) was measured at GS87 in three main stems per plot from the tillering node to the top of the spike, excluding the awns. The length of the peduncle (PL, cm) was measured on the same stems. Grain yield was determined by mechanically harvesting the plots at ripening and was expressed at a 12% moisture level. Grain weight (W, mg grain^−1^) was determined by counting the grains in 10 g drawn randomly from harvested grains of each plot. The mean grain-filling rate (GFR, mg d^−1^) was calculated for each plot as the ratio between grain weight and the number of days from anthesis to physiological maturity. Canopy temperature was measured around noon on sunny days with an infrared thermometer (Raynger II, Raytek Inc.) and air temperature was simultaneously measured using a digital thermometer (Boneco 7041). Canopy temperature depression (CTD) was determined at anthesis (CTD_A_) and again two weeks after it, when the crop was at milky-dough grain stage (CTD_Mi_), as CTD = Ta − Tc, where Ta was the air temperature and Tc the canopy temperature mean of three measures per plot. Photosynthetically active radiation intercepted by the crop canopy (IPAR) was determined on clear days from 12:00 to 15:00 h (local time) every three-weeks from emergence to physiological maturity using a portable ceptometer (AccuPAR model LP-80, Decagon Devices Inc., Pullman, WA, USA), using the following equation (Li et al., 2009):

$$IPAR=100\;x\;\frac{\text{PARi}-\text{PARt}-\text{PARr}}{\text{PARi}}$$

where PAR_i_, PAR_t_ and PAR_r_ designating incident, transmitted and reflected radiations, respectively. Two measurements were made in each plot and the cumulated amount of PAR absorbed by the canopy (MJ m^−2^) from emergence to heading (cPAR_EH_), from heading to anthesis (cPAR_HA_) and from anthesis to maturity (cPAR_AM_) were determined. Radiation use efficiency (RUE, g MJ^−1^) from emergence to anthesis (RUE_EA_) and during grain filling or anthesis to maturity (RUE_AM_) were determined using the following equation (Plenet et al., 2000):

$$\text{RUE}=\left({\text{DM}}_{\text{n}}-{\text{DM}}_{\text{n}-1}\right)/\left({\text{cPAR}}_{\text{n}}-{\text{cPAR}}_{\text{n}-1}\right)$$

where: DM is the above-ground biomass measured at dates *n* and *n*−1; cPAR_*n*_ and cPAR_n–1_ are the cumulated amounts of PAR absorbed by the canopy at dates *n* and *n*−1. Digital pictures were taken every second week, from emergence to the beginning of jointing. Three pictures were taken on each plot around noon with a digital camera held at about 60 cm above and parallel to the canopy. The software BreedPix (Casadesús and Villegas, 2014) was used to estimate the green area (GA) of each plot at each image acquisition occasion. The green area accumulated 90 days from emergence (GA_90d_) was then calculated by adding the GA daily values obtained by linear interpolation.

### Genotyping

DNA isolation was performed from leaf samples following the method reported by Doyle and Doyle (1987). High-throughput genotyping was performed at Diversity Arrays Technology Pty Ltd (Canberra, Australia) (http://www.diversityarrays.com) with the DArTseq genotyping by sequencing platform. A total of 46,161 markers were used to genotype the association mapping panel, including 35,837 PAVs (presence/absence variants) and 10,324 SNPs. Markers were ordered according to the consensus map of wheat v4 available at https://www.diversityarrays.com/technology-and-resources/genetic-maps/ (Diversity Arrays Technology Pty Ltd, Canberra, Australia).

### Data Analyses

Raw data were fitted to a linear mixed model with the check cultivars as fixed effects and the genotype, row number and column number as random effects. Restricted maximum likelihood (REML) was used to estimate the variance components and to produce the best linear unbiased predictors (BLUPs) of each accession in each year. The MIXED procedure of the SAS statistical package (SAS Institute Inc, Cary, NC, USA) was used with the Kenward-Roger correction due to the unbalanced number of genotypes within subpopulations. Multiple linear regression was performed for the 372 genotypes among the genetic structure coefficients (Soriano et al., 2021) and the average values across years of the phenotypic traits using JMP V.14 software (SAS Institute Inc.). To compare mean values of the analyzed traits between landraces and modern cultivars and between subpopulations, the sum of squares of the genotype effect in the ANOVAs was partitioned into differences between the two types of germplasm or subpopulations, and the genotypic variance retained within them, which was used as error term. Means were compared at *P* < 0.05 using the Tukey-Kramer correction with the SAS statistical package. Mean genotypic values across years were used to perform a hierarchical cluster analysis by the Ward method of the JMP V.14 software (SAS Institute Inc.) Principal component analysis was performed on the correlation matrix using the mean values across years of genotypes with a membership coefficient *q* > 0.80 to the five genetic subpopulations determined by Soriano et al. (2021). Pearson correlation coefficients were calculated between traits using the mean genotype data across years.

A GWAS was performed for the landraces and modern cultivars using the BLUPs of the measured traits for each year and across years using a mixed linear model (MLM) with TASSEL software version 5.0 (Bradbury et al., 2007).

The MLM accounted for population structure using a principal component analysis (PCA) matrix with 6 principal components as the fixed effect and a kinship (K) matrix as the random effect (PCA + K) at the optimum compression level following the equation:

y=Xβ+Zu+e

where y is the trait value, β is the fixed effect for the marker and u is a vector of random effects not associated for the markers; X and Z are incidence matrices linking y to β and u. Finally, e is the undetected vector of random residual.

A false discovery rate (FDR) threshold (Benjamini and Hochberg, 1995) was established at *P* < 0.05 for considering a Marker-Trait Association (MTA) as significant and the results were expressed with the associated *P*-values on a -log10 scale.

QTL hotspots were defined based on the LD decay at 1cM reported by Soriano et al. (2021). A hotspot was defined when at least two MTAs from different years were included within the same LD block. Subsequently, to identify haplotype differences among SPs for the analyzed traits, haplotype blocks (HB) were defined within QTL hotspots when the frequency of the haplotype differences among SPs were higher than 60%.

## Results

A graphical summary of the monthly temperature profiles and precipitation that prevailed during each experimental year is presented in Supplementary Figure 1. The 2013 season was characterized by an average rainfall (358 mm) while the 2014 and 2015 season were rather dry (203 and 271 mm, respectively). However, average experiment yields were of moderate magnitude: 5.05 t/ha, 3.33 t/ha and 4.41 t/ha in 2013, 2014, and 2015, respectively. During the 3 years, fall and winter temperatures were cold-to-cool (minimum daily temperatures below 10°C) until the end of April. The last months of the season were relatively warm but never excessively. All genotypes were able to develop and mature properly and no substantial lodging was observed, certainly not enough to affect final performance of any landrace or modern cultivars. Furthermore, there was no incidence of any disease or pest that could have differentially affected susceptible genotypes.

### Phenotypical Attributes Discriminating Landraces From Modern Cultivars

The ANOVAs showed statistically significant differences between landraces and modern cultivars for most traits (Table 1). The higher yield observed in modern cultivars compared to landraces was associated with a greater NGS and a substantially higher HI. Both types of germplasm had similar NS and W. Landraces were characterized by a longer time to heading compared to modern genotypes, by shorter grain filling duration (time from heading to anthesis and from anthesis to physiological maturity) but with a greater GFR. The duration of each phenological period was strongly associated with the radiation absorbed during the same period as shown by the significant (*P* < 0.001) correlation observed for both landraces and modern cultivars (data not shown). On average, landraces were 30 cm taller than the modern cultivars, with a peduncle that was 11 cm longer. They also produced more aboveground biomass, both at anthesis and at physiological maturity. CTD at anthesis was significantly higher in modern cultivars but this trend was reversed at the milky-dough grain stage. RUE up to anthesis was significantly higher in modern cultivars, but the opposite was true during the grain-filling period. Diagrams of frequency distribution for each trait and germplasm type are shown in Supplementary Figure 2. They confirm the above-mentioned trends based on means and provide additional information, by looking at the extent of the overlap between the distributions from both germplasm types, on the extent to which different traits differentiate modern cultivars from landraces. The least overlap in frequency distribution between these two groups was observed for PH, HI, D_EH_, D_AM_ and ultimately yield.

**Table 1:** Analyses of variance and mean values ± SE for agronomic traits of a panel of durum wheat Mediterranean landraces and modern cultivars of diverse origin.

**Trait**	**Landraces (*n* = 172)**	**Modern (*n* = 200)**	***F* value**	***P*-value**
Yield (kg ha^−1^)	3967 ± 36.5	4515 ± 33.4	340.91	<0.0001
NS	337 ± 1.9	338 ± 1.6	0.53	0.4867
NGS	30.4 ± 0.28	33.4 ± 0.25	59.38	<0.0001
W (mg grain^−1^)	44.1 ± 0.32	44.5 ± 0.34	1.04	0.3549
HI	0.38 ± 0.001	0.46 ± 0.002	937.66	<0.0001
D_EH_	130.6 ± 0.55	124.4 ± 0.50	312.80	<0.0001
D_HA_	4.6 ± 0.08	6.2 ± 0.08	259.41	<0.0001
D_AM_	31.3 ± 0.22	33.8 ± 0.21	275.25	<0.0001
GFR (mg day^−1^)	1.43 ± 0.012	1.32 ± 0.008	67.65	<0.0001
PL (cm)	45.7 ± 0.43	34.6 ±0.20	556.09	<0.0001
PH (cm)	108.3 ± 0.73	77.6 ± 0.38	658.59	<0.0001
DM_A_ (g m^−2^)	759 ± 4.2	712 ± 3.5	81.51	<0.0001
DM_M_ (g m^−2^)	1180 ± 12.9	1096 ±9.1	35.19	<0.0001
CTD_A_ (°C)	0.75 ± 0.12	1.12 ± 0.08	6.43	0.0116
CTD_Mi_ (°C)	−0.36 ± 0.10	−0.65 ± 0.10	11.86	0.0006
cPAR_EH_ (MJ m^−2^)	280 ± 1.9	230 ± 1.5	340.31	<0.0001
cPAR_HA_ (MJ m^−2^)	33.4 ± 0.53	38.3 ±0.47	69.05	<0.0001
cPAR_AM_ (MJ m^−2^)	166 ± 2.5	172 ± 2.3	33.33	<0.0001
RUE_EA_ (g MJ^−1^)	2.47 ± 0.02	2.69 ± 0.02	80.08	<0.0001
RUE_AM_ (g MJ^−1^)	2.53 ± 0.06	2.24 ± 0.05	12.31	0.0003
GA_90d_	141 ± 2.9	136 ± 2.7	38.46	<0.0001

*Data are means across three years. Numerator D.F. = 1, Denominator D.F. = 370*.

*NS, number of spikes m^−2^; NGS, number of grains spike^−1^; W, grain weight; HI, harvest index; DEH, days from emergence to heading; DHA, days from heading to anthesis; DAM, days from anthesis to maturity; GFR, grain filling rate; PL, peduncle length; PH, plant height; DM_A_, dry matter at anthesis; DM_M_, dry matter at maturity; CTD_A_, canopy temperature depression at anthesis; CTD_Mi_, canopy temperature depression at milky-dough grain stage; cPAR_EH_, absorbed radiation from emergence to heading; cPAR_HA_, absorbed radiation from heading to anthesis; cPAR_AM_, absorbed radiation from anthesis to maturity; RUE_EA_, radiation use efficiency from emergence to anthesis; RUE_AM_, radiation use efficiency from anthesis to maturity; GA_90d_, green area accumulated at 90 days from emergence*.

**Figure 1:**
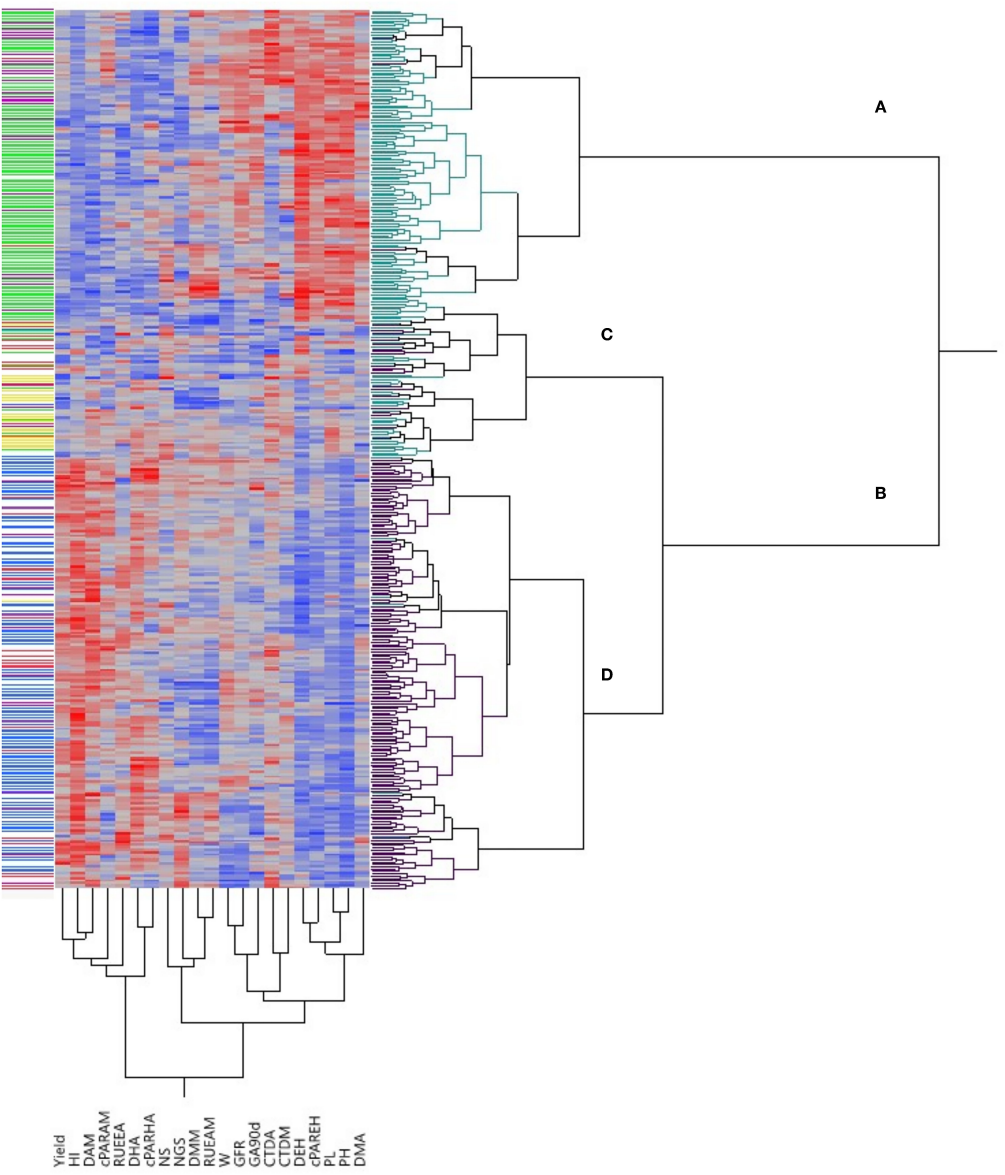
Bi-dimensional clustering showing the relationships between the 372 genotypes included in this study according to the traits shown in the bottom part of the figure. Red and blue colors in the columns denote high and low values, respectively, for each trait, with color intensity associated to trait values. Green and black cluster lines at right correspond to landraces and modern cultivars, respectively. Cluster lines at left indicate genetic subpopulations: yellow, SP1; green, SP2; purple, SP3; blue, SP4; red, SP5; white, admixed. NS, number of spikes/m^2^; NGS, number of grains/spike; W, grain weight; HI, harvest index; DEH, days from emergence to heading; DHA, days from heading to anthesis; DAM, days from anthesis to maturity; GFR, grain filling rate; PL, peduncle length; PH, plant height; DM_A_, dry matter at anthesis; DM_M_, dry matter at maturity; CTD_A_, canopy temperature depression at anthesis; CTD_Mi_, canopy temperature depression at milky-dough grain stage; cPAR_EH_, absorbed radiation from emergence to heading; cPAR_HA_, absorbed radiation from heading to anthesis; cPAR_AM_, absorbed radiation from anthesis to maturity; RUE_EA_, radiation use efficiency from emergence to anthesis; RUE_AM_, radiation use efficiency from anthesis to maturity; GA_90d_, green area accumulated at 90 days from emergence.

The bi-dimensional clustering shown in Figure 1 illustrates the relationships between phenotypic traits and their relative value (the darker the color, the higher the value) in the two types of germplasm. The horizontal cluster grouped accessions according to their phenotypic similarity based on the traits considered in the vertical cluster. Two main clusters included the majority of landraces (in cluster A) separated from the majority of modern cultivars (found in cluster B). However, the latter was further divided into clusters C and D, with cluster C containing both landraces and modern cultivars. Cluster A was characterized by superior values for D_EH_, cPAR_EH_, PL, PH and DM_A_, while cluster D grouped genotypes with more yield, HI, D_AM_, cPAR_AM_, RUE_EA_, D_HA_ and cPAR_HA_. Three modern cultivars were identified in cluster A: the Canadian cultivars “Macoun” and “Waskana” that were placed jointly with the landraces “Entrelargo de Montijo” and “Trigo Glutinoso” from Spain and France, respectively, and the US cultivar “West Bred Laker,” which clustered in the same branch than the Lebanese landrace “IG-84856.” Conversely, four landraces were grouped in cluster D jointly with modern cultivars. The Italian landrace “Capeiti” was close to the modern Syrian cultivars “Awalbit 7” and “Omrabi 3.” A phenotypic similarity was also detected between the Israeli landraces “Etith” and “JM-3987” and the modern cultivars “Annouar” and “Fjord” from Morocco and USA, respectively. Finally, the Italian landrace “Hymera” clustered close to the modern American “Duraking.” Cluster C in Figure 1 was in a common branch with modern cultivars, but nevertheless included a large number of landraces. Six of them were phenotypically similar to improved cultivars: the three Egyptian landraces “1P1,” “Milagro” and “Giza 2,” which were in the same branch than the North-American cultivars “Medora,” “Lakota” and “AC Avonlea,” respectively. The Italian “IG-83920” was close to the modern cultivars “Ward” from USA and “Quabrach-1” from Syria', while the Crete “IG-96851” and the Lebanese “9981” were in the same branch than the Canadian “Springfield” and “Wakooma,” respectively.

Correlation coefficients between traits for landraces and modern cultivars are shown in Supplementary Figure 3. Some strong relationships between agronomic traits were consistently found in both types of germplasm, such as the negative correlation coefficients between HI and D_EH_, and between NGS with W and GFR. Similarly, consistent associations were identified in landraces and modern cultivars between NGS and RUE_AM_ and between GFR and W. Correlation coefficients with yield showed that the traits that mostly influenced it were W (*r* = 0.54, *P* < 0.0001) and GFR (*r* = 0.45, *P* < 0.0001) in landraces, but HI (*r* = 0.48, *P* < 0.0001) in modern cultivars. It was also noticeable the different sign in landraces and modern cultivars of the correlation coefficients between CTD_A_ and D_EH_ (*r* = −0.46, *P* < 0.0001 in landraces and *r* = 0.17, *P* < 0.014 in modern cultivars).

### Relationship Between Genetic Structure and Agronomic Performance

To quantify the relation between trait variation and population structure, multiple linear regressions were carried out among population structure coefficients (Supplementary Table 1) and phenotypic performance (Supplementary Table 2). The *R*^2^ values ranged from 0.0471 for RUE_AM_ to 0.7416 for HI (Table 2). The results pointed out the selection during the breeding process for yield, phenology and plant size.

**Table 2:** Multiple linear regression (*R*^2^) estimation of the impact of genetic population structure on the phenotypic traits.

**Trait**	**Multiple *R^**2**^***
Yield	0.4859
NS	0.1244
NGS	0.1147
W	0.1875
HI	0.7416
D_EH_	0.6823
D_HA_	0.5477
D_AM_	0.4901
GFR	0.2988
PL	0.5772
PH	0.7207
DM_A_	0.2569
DM_M_	0.1489
CTD_A_	0.0829
CTD_Mi_	0.0600
cPAR_EH_	0.6232
cPAR_HA_	0.2464
cPAR_AM_	0.1055
RUE_EA_	0.2242
RUE_AM_	0.0471
GA90d	0.1957

*NS, number of spikes m^−2^; NGS, number of grains spike^−1^; W, grain weight; HI, harvest index; DEH, days from emergence to heading; DHA, days from heading to anthesis; DAM, days from anthesis to maturity; GFR, grain filling rate; PL, peduncle length; PH, plant height; DM_A_, dry matter at anthesis; DM_M_, dry matter at maturity; CTD_A_, canopy temperature depression at anthesis; CTD_Mi_, canopy temperature depression milky-dough grain stage; cPAR_EH_, absorbed radiation from emergence to heading; cPAR_HA_, absorbed radiation from heading to anthesis; cPAR_AM_, absorbed radiation from anthesis to maturity; RUE_EA_, radiation use efficiency from emergence to anthesis; RUE_AM_, radiation use efficiency from anthesis to maturity; GA_90d_, green area accumulated at 90 days from emergence*.

**Figure 2:**
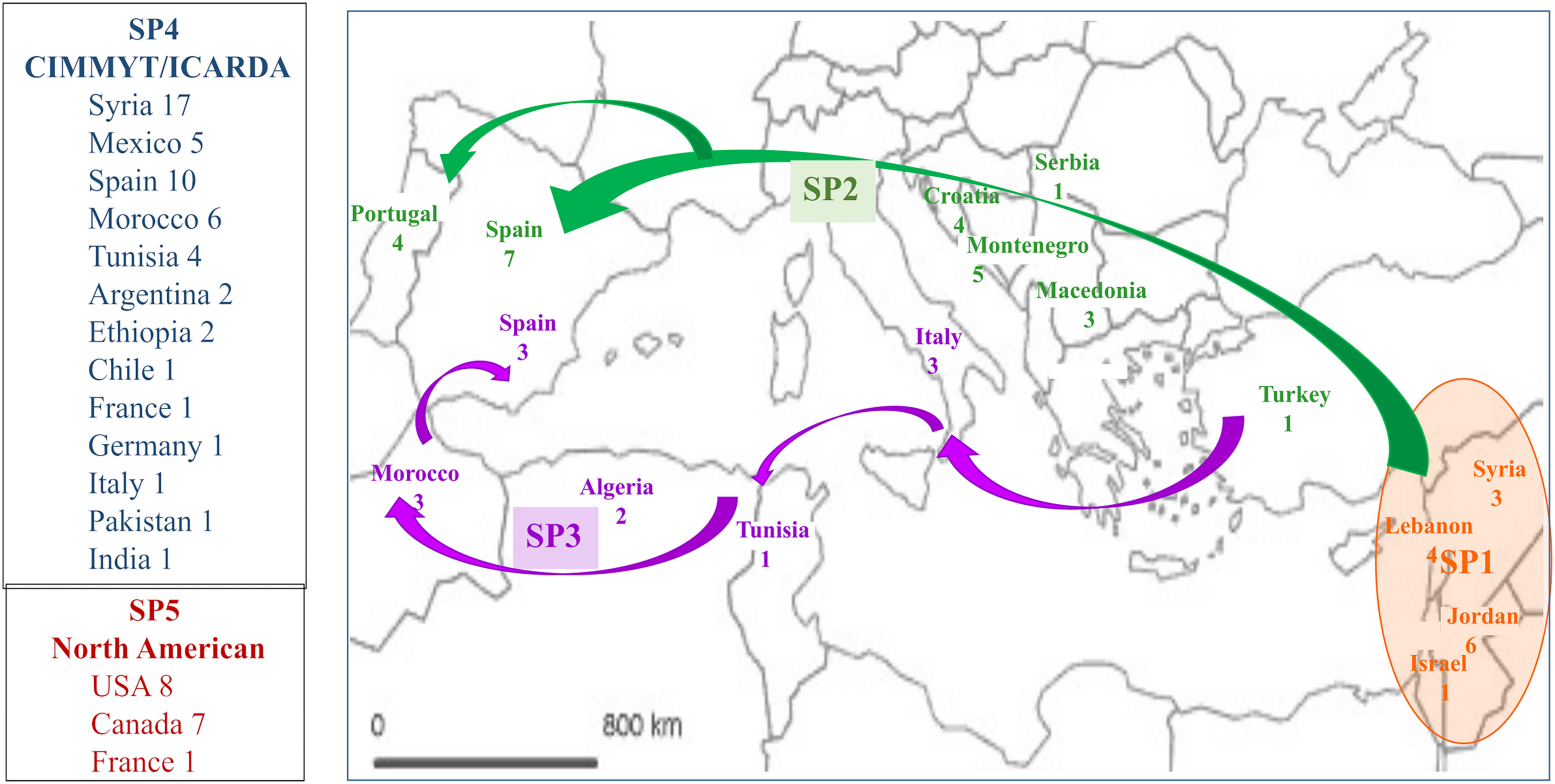
Origin of the 120 accessions with a membership coefficient *q* > 0.80 to each of five genetic subpopulations. SP1, SP2 and SP3 include landraces from the area of wheat domestication and the routes of expansion of wheat cultivation within the Mediterranean Basin (Feldman, 2001). SP4 and SP5 (on the left side of the map) include modern cultivars. Numbers indicate the number of accessions from each origin.

The selection of members with a membership coefficient *q* > 0.80 to each of five genetic subpopulations identified 120 genotypes (Supplementary Table 1). SP1 contained 15 landraces, 13 (87%) from Jordan, Israel, Lebanon and Syria, countries close to the area of tetraploid wheat domestication, plus two Italian landraces “Aziziah 17/45,” *q* = 0.999) and “IG-83920,” *q* = 0.907). SP2 included 25 landraces from northern Mediterranean countries (Turkey, Macedonia, Montenegro, Serbia, Croatia, Spain and Portugal) and one landrace, “Douro Boukowo” cataloged as Moroccan. SP3 included 11 landraces from western Mediterranean countries (Italy, Tunisia, Algeria, Morocco and Spain), and the modern Canadian cultivar “Macoun” (*q* = 0.999). SP4 grouped 52 modern cultivars related to the CIMMYT and ICARDA germplasm pools. The origins more represented in this SP were Syria (33%), Spain (19%), Morocco (11%), Mexico (10%) and Tunisia (8), with a minor number of accessions from Argentina, Chile, Ethiopia, France, Germany, India, Italy and Pakistan (Supplementary Table 1). Finally, SP5 included 16 modern cultivars, 15 of them from USA and Canada and the French cultivar “Auroc” (*q* = 0.949), which is very likely genetically related with the north-American germplasm pool. These results revealed a different origin for each subpopulation i.e., SP1, SP2, and SP3 containing landraces from eastern, northern and western Mediterranean countries, respectively, SP4 grouping modern cultivars derived from the international centers CIMMYT and ICARDA, and SP5 grouping modern north-American cultivars (Figure 2).

**Figure 3:**
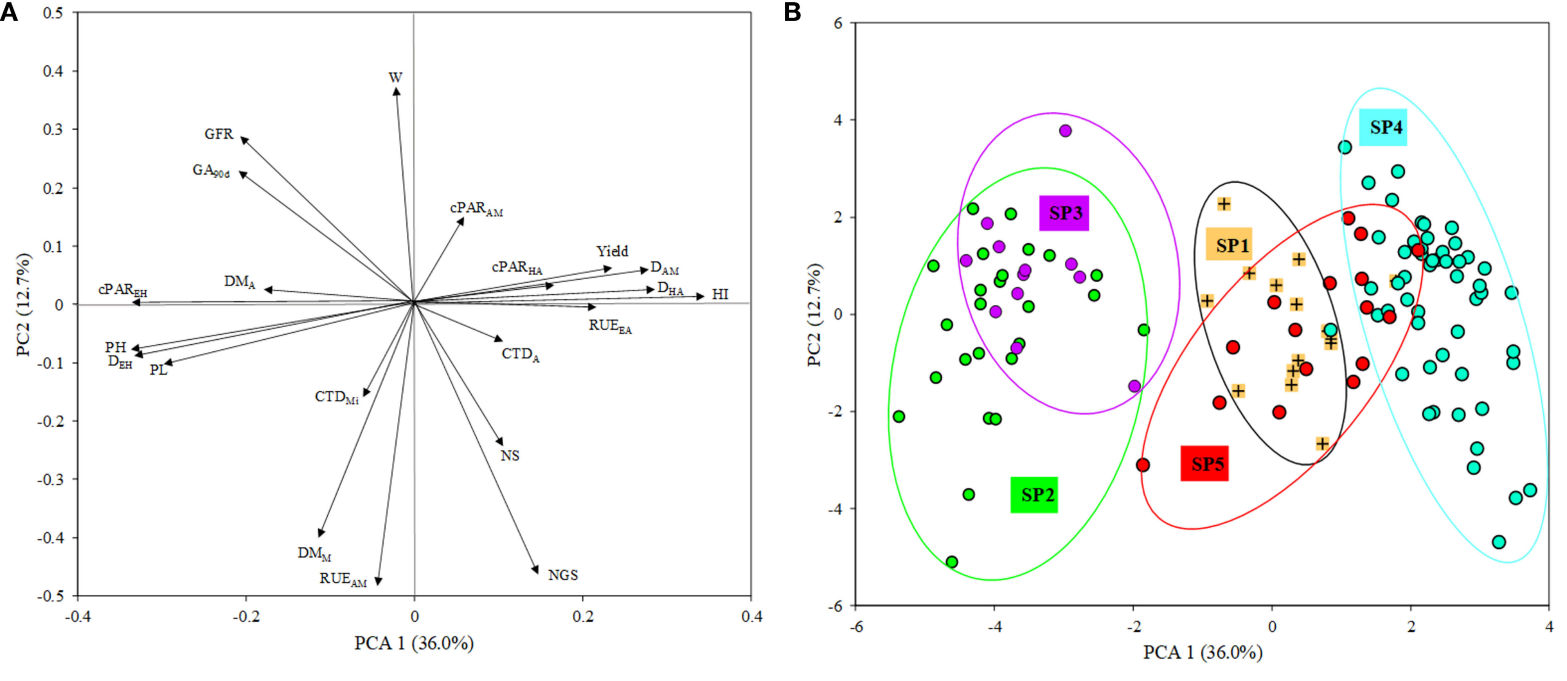
Biplot of principal component analysis (PCA). **(A)** Eigenvalues of the correlation matrix symbolized as vector representing the traits measured in the study. NS, number of spikes/m^2^; NGS, number of grains/spike; W, grain weight; HI, harvest index; DEH, days from emergence to heading; DHA, days from heading to anthesis; DAM, days from anthesis to maturity; GFR, grain filling rate; PL, peduncle length; PH, plant height; DM_A_, dry matter at anthesis; DM_M_, dry matter at maturity; CTD_A_, canopy temperature depression at anthesis; CTD_Mi_, canopy temperature depression at milky-dough grain stage; RAD_EH_, absorbed radiation from emergence to heading; RAD_HA_, absorbed radiation from heading to anthesis; RAD_AM_, absorbed radiation from anthesis to maturity; RUE_EA_, radiation use efficiency from emergence to anthesis; RUE_AM_, radiation use efficiency from anthesis to maturity; GA_90d_, green area accumulated at 90 days from emergence. **(B)** Points representing durum wheat landraces and modern cultivars with a membership coefficient *q* > 0.80 to five genetic subpopulations (SP).

A graphical representation illustrating the relationship between the five genetic subpopulations and the phenotypic traits analyzed in the current study was obtained through multivariate analysis. The first two axes of a principal component analysis (PCA) conducted with the mean data across years of the 120 genotypes accounted for around 49% of the variability contained in the phenotypic data, with PC1 explaining 36.0% of it (Figure 3). The main traits affecting PC1 were HI, D_HA_, D_AM_, yield and RUE_EA_ in a positive direction and cPAR_EH_, D_EH_, PH and PL in the negative direction (Figure 3A). The distribution of points representing genotypes within the plane formed by the two main PC axes clustered those from SP1 close to their origin and discretely separated from those belonging to SP2 and SP3, which grouped at the negative direction of PC1, overlapping with each other. Genotypes corresponding to SP4 were located in the positive direction of the same axis, while genotypes from SP5 overlapped with those of SP1 and SP4 (Figure 3B). The genetic structure of the whole panel of 372 genotypes is shown in the left part of the phenotype-based clustering of Figure 1.

Mean values of phenotypic traits for each subpopulation are shown in Table 3. The results are consistent with the trends noted in Table 1 when comparing the means of landraces and those of modern cultivars: the average yield of SP4 was greater than those observed for any of the other groups and this superiority was mirrored by greater HI and NGS. This was the case even when compared to the other modern group, SP5. Average values of early radiation use efficiency (RUE_EA_) was significantly higher in both modern cultivar SPs than in the landrace SPs. Table 3 shows average values of SP1 intermediate between those observed in the other SPs for traits such as yield, NS, HI, PH, cPAR_EH_ and GA_90d_, as already suggested by the multivariate analysis. Average values for SP2 were reduced by 6% for NS and 10% for HI with no significant changes in NGS or W relative to those for SP1. Landraces from SP3 were characterized by a greater average yield compared to those from SP1 and SP2 and that superiority was paralleled by a higher W. The analysis of crop development revealed that the average time to heading of SP1 was lower than those observed for SP2 and SP3, while the opposite was true for the duration of the period from heading to maturity. This was associated with a greater average GFR for SP2 and SP3. Landraces from SP2 and SP3 were, on average, significantly taller than those from SP1 and produced more aboveground biomass at anthesis. Differences between SP in cPAR followed a similar trend than that observed for phenological traits. In terms of green leaf are accumulated, GA_90d_, the average value for landraces from SP2 and SP3 were greater than the values for landraces from SP1, the latter being similar to those for the modern cultivars SPs.

**Table 3:** Analyses of variance and mean values ± SE for the studied traits on durum wheat Mediterranean landraces and modern cultivars with a membership coefficient *q* > 0.80 to five genetic subpopulations (SP).

**Trait**	**Modern cultivars**		**Mediterranean landraces**			***F* value**	***P*-value**
	**SP4 (CIMMYT + ICARDA)**	**SP5 (North-American)**	**SP1 (Eastern)**	**SP2 (Northern)**	**SP3 (Western)**		
Yield (kg ha^−1^)	4516 ± 35.7^A^	4270 ± 78.1^B^	3860 ±71.7^C^	3846 ± 57.6^C^	4187 ± 25.9^B^	36.03	<0.0001
NS	341 ± 2.5^AB^	346 ± 5.8^AB^	353 ± 5.4^A^	331 ± 4.9^BC^	322 ± 4.3^C^	5.41	0.0005
NGS	33.4 ± 0.66^A^	31.9 ± 0.41^AB^	29.7 ± 0.75^B^	30.1 ± 0.71^B^	29.1 ± 0.79^B^	6.07	0.0002
W (mg grain^−1^)	45.0 ± 0.58^B^	42.4 ± 0.42^C^	42.9 ± 0.54^C^	43.2 ± 0.60^C^	48.8 ± 0.63^A^	8.78	<0.0001
HI	0.47 ± 0.003^A^	0.43 ± 0.006^B^	0.41 ± 0.002^B^	0.37 ± 0.004^C^	0.37 ± 0.003^C^	151.71	<0.0001
D_EH_	123.3 ± 0.14^E^	126.8 ± 0.67^C^	124.3 ± 0.70^D^	134.4 ± 0.38^A^	131.4 ± 0.30^B^	188.55	<0.0001
D_HA_	6.61 ± 0.11^A^	5.46 ± 0.22^B^	6.01 ± 0.20^B^	4.45 ± 0.14^C^	3.89 ± 0.13^D^	52.36	<0.0001
D_AM_	33.6 ± 0.16^A^	32.5 ± 0.37^BC^	33.4 ± 0.24^AB^	30.4 ± 0.22^D^	31.6 ± 0.24^C^	36.76	<0.0001
GFR (mg day^−1^)	1.34 ± 0.017^C^	1.32 ± 0.012^C^	1.30 ± 0.016^C^	1.46 ± 0.025^B^	1.57 ± 0.018^A^	18.59	<0.0001
PL (cm)	34.2 ± 0.38^D^	38.0 ± 1.21^C^	44.5 ± 0.81^B^	49.3 ± 0.97^A^	44.7 ± 1.15^B^	79.91	<0.0001
PH (cm)	74.4 ± 0.66^D^	90.0 ± 3.37^C^	91.2 ± 0.91^C^	121.0 ± 2.14^A^	108.8 ± 1.91^B^	159.74	<0.0001
DM_A_ (g m^−2^)	711 ± 5.4^C^	729 ± 10.4^BC^	692 ± 8.6^C^	760 ± 9.6^B^	804 ± 19.8^A^	16.47	<0.0001
DM_M_ (g m^−2^)	1095 ± 16.7^B^	1102 ± 32.5^AB^	1082 ± 31.2^B^	1168 ± 30.0^AB^	1221 ± 40.7^A^	3.50	0.0084
CTD_A_ (°C)	0.82 ± 0.14^B^	1.82 ± 0.31^A^	1.23 ± 0.30^AB^	−0.54 ± 0.30^C^	1.69 ± 0.38^AB^	15.87	<0.0001
CTD_Mi_ (°C)	−0.74 ± 0.19^A^	−0.71 ± 0.35^A^	−0.65 ± 0.33^A^	−0.58 ± 0.30^A^	−0.09 ± 0.36^A^	1.35	0.2547
cPAR_EH_ (MJ m^−2^)	222 ± 2.1^C^	239 ± 5.1^B^	242 ± 4.3^B^	293 ± 3.7^A^	289 ± 4.6^A^	95.71	<0.0001
cPAR_HA_ (MJ m^−2^)	40.2 ± 0.70^A^	36.8 ± 1.33^AB^	36.8 ± 1.22^AB^	35.4 ± 1.01^B^	30.5 ± 0.63^C^	11.81	<0.0001
cPAR_AM_ (MJ m^−2^)	167.5 ± 1.6^AB^	170.5 ± 2.5^A^	170.9 ± 2.6^A^	161.4 ± 1.8^B^	170.6 ± 3.1^A^	3.13	0.0176
RUE_EA_ (g MJ^−1^)	2.7 ± 0.03^A^	2.6 ± 0.05^AB^	2.5 ± 0.05^BC^	2.4 ± 0.05^C^	2.5 ± 0.08^BC^	12.39	<0.0001
RUE_AM_ (g MJ^−1^)	2.3 ± 0.10^A^	2.1 ± 0.17^A^	2.3 ± 0.18^A^	2.4 ± 0.17^A^	2.5 ± 0.26^A^	0.53	0.7154
GA_90d_	135.4 ± 1.0^B^	135.0 ± 1.4^B^	136.4 ± 1.5^B^	143.1 ± 1.4^A^	149.0 ± 1.5^A^	15.45	<0.0001

*Data are means across three years. Numerator D.F. = 4, Denominator D.F. = 115*.

*NS, number of spikes m^−2^; NGS, number of grains spike^−1^; W, grain weight; HI, harvest index; DEH, days from emergence to heading; DHA, days from heading to anthesis; DAM, days from anthesis to maturity; GFR, grain filling rate; PL, peduncle length; PH, plant height; DM_A_, dry matter at anthesis; DM_M_, dry matter at maturity; CTD_A_, canopy temperature depression at anthesis; CTD_Mi_, canopy temperature depression at milky-dough grain stage; cPAR_EH_, absorbed radiation from emergence to heading; cPAR_HA_, absorbed radiation from heading to anthesis; cPAR_AM_, absorbed radiation from anthesis to maturity; RUE_EA_, radiation use efficiency from emergence to anthesis; RUE_AM_, radiation use efficiency from anthesis to maturity; GA_90d_, green area accumulated at 90 days from emergence. Means within columns with different letters are significantly different at P < 0.05 following a Tukey test*.

### Identification of Genomic Regions Differentiating Between Subpopulations

A total of 46,161 DArTseq markers were used to genotype the durum wheat collection as reported by Soriano et al. (2021). After removing markers with allelic frequency lower than 5%, those with more than 30% of missing data and duplicated markers, a total of 23,716 remained, 19,030 of those consisting of PAVs and 4,686 of actual SNPs.

A GWAS was performed using the 23,716 markers deemed to be suitable and phenotypic data of the 21 traits listed in Tables 1–3, separately for each of the 3 years of the study. A total of 2,046 MTAs were identified using a common threshold at a −log10 *P* = 3, with 273 of these MTAs being above the FDR threshold at −log10 *P*>4.6 (Table 4, Supplementary Table 3). The number of MTAs ranged from 41 in chromosome 4B to 472 in chromosome 2A, with an average of 146 MTAs/chromosome.

**Table 4:** Number of MTAs per trait and year.

**Trait**	**2013**	**2014**	**2015**	**Total**
Yield	25 (2)	17 (0)	19 (1)	61 (3)
NS	25 (3)	11 (0)	16 (1)	52 (4)
NGS	81 (14)	50 (23)	39 (14)	170 (51)
W	96 (39)	61 (29)	77 (33)	234 (101)
HI	11 (0)	15 (1)	30 (0)	56 (1)
D_EH_	24 (0)	32 (7)	62 (7)	118 (14)
D_HA_	20 (1)	29 (1)	29 (0)	78 (2)
D_AM_	32 (0)	22 (1)	28 (2)	82 (3)
GFR	97 (28)	55 (0)	83 (29)	235 (57)
PL	33 (0)	27 (2)	52 (3)	112 (5)
PH	30 (3)	60 (3)	86 (7)	176 (13)
DM_A_	21 (0)	9 (0)	32 (2)	62 (2)
DM_M_	55 (1)	14 (0)	20 (1)	89 (2)
CTD_A_	15 (0)	9 (0)	29 (2)	53 (2)
CTD_Mi_	15 (0)	55 (7)	20 (1)	90 (8)
cPAR_EH_	21 (0)	15 (0)	26 (0)	62 (0)
cPAR_HA_	25 (0)	21 (0)	9 (0)	55 (0)
cPAR_AM_	19 (0)	45 (0)	40 (0)	104 (0)
RUE_EA_	10 (0)	13 (0)	6 (0)	29 (0)
RUE_AM_	48 (2)	7 (0)	9 (1)	64 (3)
GA_90d_	26 (1)	12 (1)	26 (0)	64 (2)

*In parenthesis are indicated the MTAs above the FDR threshold. NS, number of spikes m^−2^; NGS, number of grains spike^−1^; W, grain weight; HI, harvest index; DEH, days from emergence to heading; DHA, days from heading to anthesis; DAM, days from anthesis to maturity; GFR, grain filling rate; PL, peduncle length; PH, plant height; DM_A_, dry matter at anthesis; DM_M_, dry matter at maturity; CTD_A_, canopy temperature depression at anthesis; CTD_Mi_, canopy temperature depression at milky-dough grain stage; cPAR_EH_, absorbed radiation from emergence to heading; cPAR_HA_, absorbed radiation from heading to anthesis; cPAR_AM_, absorbed radiation from anthesis to maturity; RUE_EA_, radiation use efficiency from emergence to anthesis; RUE_AM_, radiation use efficiency from anthesis to maturity; GA_90d_, green area accumulated at 90 days from emergence*.

To simplify the MTAs information, QTL hotspots were defined based on the LD decay at 1cM reported by Soriano et al. (2021). A hotspot was defined when at least two MTAs from different years were included within the same LD block. A total of 144 QTL hotspots were identified integrating 1927 MTAs (Supplementary Table 4). The number of MTAs per QTL hotspot ranged from 2 to 427 in the QTL hotspot 2A.2, covering a region of ~20 cM on the distal part of the short arm of chromosome 2A. This QTL hotspot integrates 177 from 273 MTAs over the FDR threshold (65%).

**Figure 4:**
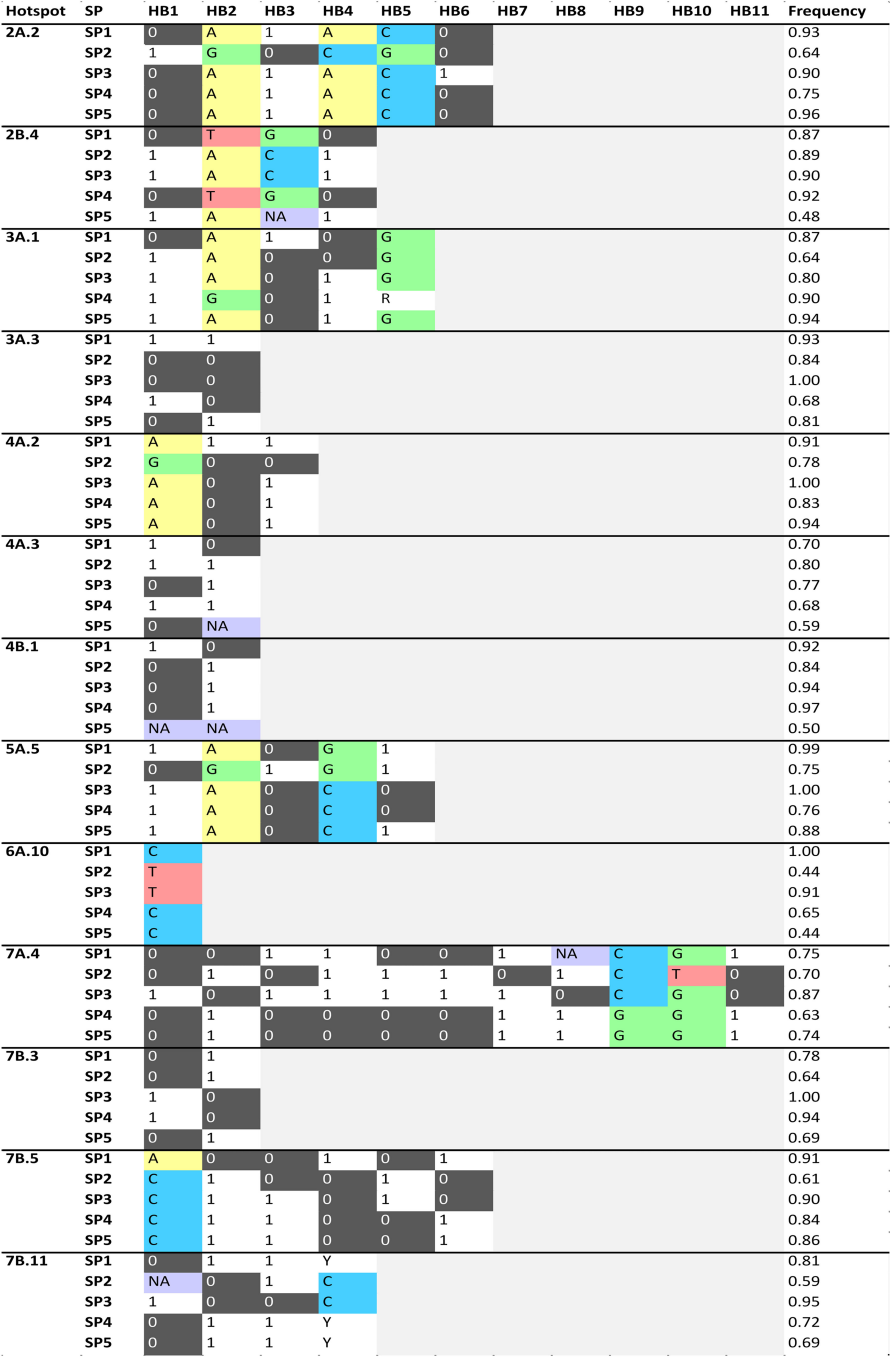
Haplotype blocks (HBs) for each one of the QTL hotspots identified among 120 durum wheat modern and landrace genotypes. The frequency corresponds to the mean of the frequency of the major allele for each marker within each SP. NA: Frequency of the major allele lower than 60%. PAVs are codified as 1 (presence) or 0 (absence), and SNPs following the IUPAC codes.

Haplotype (allele) differences among SPs within the QTL hotspots for the analyzed traits was carried out defining HBs when at least one SP have a different haplotype with a frequency higher than 60% than the other SPs (Figure 4). A total of 53 HBs were identified within 13 QTL hotspots. The number of HB blocks ranged from 1 in QTL hotspot 6A.10 to 11 in QTL hotspot 7A.4.

**Figure 5:**
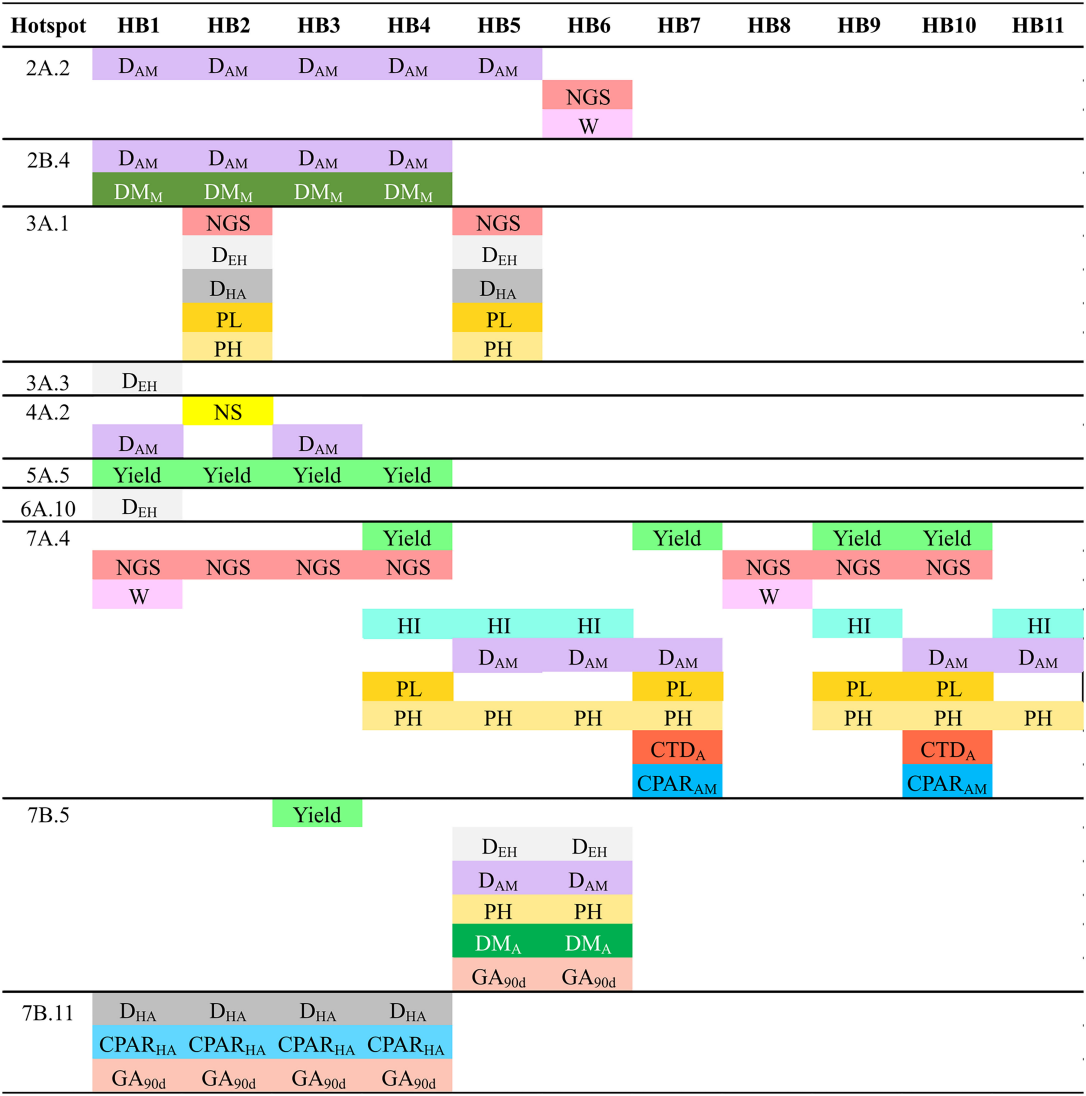
Allelic differences among SPs in each one of the QTL hotspots as reported in Figure 4 and associated agronomic traits. NS, number of spikes/m^2^; NGS, number of grains/spike; W, grain weight; HI, harvest index; DEH, days from emergence to heading; DHA, days from heading to anthesis; DAM, days from anthesis to maturity; PL, peduncle length; PH, plant height; DM_A_, dry matter at anthesis; DM_M_, dry matter at maturity; CTD_A_, canopy temperature depression at anthesis; cPAR_HA_, absorbed radiation from heading to anthesis; cPAR_AM_, absorbed radiation from anthesis to maturity; GA_90d_, green area accumulated at 90 days from emergence.

Figure 5 represents the traits showing haplotype differences for each SP. The analysis identified haplotype differences between SPs for all the QTL hotspot except 4A.3, 4B.1 and 7B.3. As an example, QTL hotspot 2A.2 with 5 HBs (HB1-5) for SP2 and one HB (HB6) for SP3 (Figure 4), showed that differences in HB1-5 are related to D_AM_, whereas the HB6 is related to NGS and W. In total we found 39 HBs associated with 16 agronomic traits. Among the different traits, D_AM_ was associated with variation in 18 HBs whereas NS was linked to only one. Six traits were not associated with haplotype differences among SPs: CPAR_EH_, GFR, CTD_Mi_, RUE_EA_, and RUE_AM_.

## Discussion

A 3-years field experiment was conducted to evaluate a panel of 172 ancestral landraces from 21 Mediterranean countries and 200 modern cultivars of diverse origins. The 21 traits determined included detailed phenological indicators, relevant physiological parameters, yield and all its components. Given the substantial differences in phenology, height and general plant type between landraces and most modern cultivars, comparing their agronomic performance in the same experiment is not straightforward and cannot be reliably done at any location and under any conditions. This is also the case when comparing performance of modern cultivars with photoperiod sensitivity (Upper Midwest of USA and Canada) to those with none (most CIMMYT/ICARDA-derived germplasm). In order to adequately assess their performance potential, landraces need to be grown under weather conditions that accommodate their general lateness and long pre-heading growth periods (extended cool temperatures during the fall and winter) while allowing for grain-fill to occur under relatively optimal conditions (no excessive heat or water stress at the end of the season). Because of their considerable height, they need to be evaluated under conditions that prevent excessive lodging, including low-to-moderate water input and avoiding excess fertilization during plot management. At the same time, the experimental conditions need to also allow for the proper expression of performance potential of the generally shorter, earlier modern cultivars in order for the comparison between the two groups to be fair and reliable. The testing site used in the present study, namely the north-eastern Spanish location of Lleida, satisfies all these prerequisites, in addition to being highly representative of rainfed Mediterranean environments (Ammar et al., 2008). In fact, during the 3 years of the field experiment, all genotypes were able to develop and mature properly and their final performance was not affected to any significant extent by either lodging or disease. Even though 2013 and 2014 were dry years, average experiment yields were higher than what one would expect given the water input. This is due to the shallow level of the water table (70 cm below the soil surface) at the Lleida experimental site, which contributes to store whatever water is absorbed by the soil at a depth that makes it available to roots for long periods of time. Taken together, these considerations strongly support the reliability and relevance of the results from the present study with regards to comparing the performance of ancestral landraces from all around the Mediterranean Basin (including those not endemic to the Iberian Peninsula) to that of modern cultivars from most spring durum breeding programs, including those producing photoperiod sensitive types. The fact that a large and balanced number of representatives from each group was included in the study enhances the fairness and robustness of the comparison between the performance of landraces and that of modern cultivars.

The large differences between the agronomic performance of durum wheat landraces and modern cultivars identified in the current study agree with the contrasting genetic background of both germplasm types reported in previous studies (Soriano et al., 2016; Mazzucotelli et al., 2020). Group mean analysis, observation of frequency distributions for each group and the multivariate analysis (PCA for traits) indicated that the significant yield advantage of modern cultivars was associated with greater number of grains per spike and a drastically improved harvest index but not with any difference in kernel weight, confirming results from previous studies (Gale and Youssefian, 1985; De Vita et al., 2007; Royo et al., 2007, 2008; Fayaz et al., 2013). Under the conditions of the present study, of all the traits evaluated, the frequency distributions for harvest index exhibited the least overlap between the two groups. Phenological differences between landraces and modern cultivars, with the latter showing a shorter average time to anthesis and longer average grain filling period, is consistent with and would provide an opportunity to produce a higher harvest index. The fact that harvest index is the most differentiating yield component (and trait in general) between landraces and modern cultivars is consistent with the extensive literature attributing an increase in harvest index as the main basis for the agronomic superiority of the semi-dwarf wheat types from the “Green Revolution” and their descendants compared to ancestral, tall landraces. While the modern cultivars included in this study were, on average and as expected, much shorter than the landraces, it is important to note that not all modern cultivars were semi-dwarfs, hence the significant overlap in frequency distribution for height between the two groups. A small minority of these are non-semi-dwarf types, including most of the representatives from the US upper Midwest and Canada, a few of the ICARDA-derived genotypes and one cultivar from Ethiopia. While these exhibited a significant improvement in performance compared to landraces, their superiority should be largely unrelated to dwarfing genes and the “Green Revolution” events.

In the current study, there was no significant difference in the average number of spikes per unit area between landraces and modern cultivars, with close to complete overlap in the frequency distributions of the two groups. This is in apparent contrast with results reported in previous studies analyzing historical series of durum cultivars from Italy and Spain where the breeding-mediated increase in spikes per unit area was a significant factor contributing to yield progress from landraces to modern cultivars (Royo et al., 2007; Subira et al., 2015). This is in part due to the much lower number of genotypes used in those two studies and their highly localized origin compared to the large number of genotypes spanning the entire Mediterranean Basin used in the present study. This apparent contradiction disappears when considering the significantly lower average number of spikes per unit area recorded in landraces from northern and western Mediterranean countries in comparison to modern cultivars (Table 3). Including in the comparison with modern cultivars landraces from the eastern Mediterranean, with their very high number of spikes per unit area, makes the difference between the two groups non-significant and may be considered in breeding programs to increase the spike number, a trait associated to adaptation to drought environments (Royo et al., 2014; Soriano et al., 2018). Previous investigations have reported a shorter time to anthesis achieved through breeding during the last century (Motzo and Giunta, 2007; Royo et al., 2008; Isidro et al., 2011). While results of the present study coincide with these findings, the more detailed phenological analysis done herein by splitting time to anthesis in two sub-periods, allowed us to discover that modern cultivars have a shorter average time to heading than landraces but a significantly larger period from heading to anthesis. Given that most floret mortality in wheat occurs from booting to anthesis (Kirby, 1988) and that floral abortion in durum wheat is negatively associated with the length of this period (Isidro et al., 2011), an increase in NGS would be reasonably expected from a prolonged heading to anthesis time, which is what is observed in the current study. In this regard eastern Mediterranean landraces may be particularly interesting for breeding purposes. The modern cultivars appeared to convert photosynthetically active radiation (PAR) up to anthesis into aboveground dry matter more efficiently than landraces. Siddique et al. (1989) obtained similar results when comparing bread wheat cultivars grown in Australia in different historical periods. However, in the current study this trend was reversed during grain filling, with landraces showing higher RUE than modern cultivars, likely because they remain green longer (due to their general lateness) and are more equipped to cope with mild moisture stress late in the season which is consistent with the lower average canopy temperature of the landraces.

The bi-dimensional cluster obtained from agronomical data (Figure 1) was useful to visualize in a structured way the variability existing within the panel and to identify phenotypical similarities between genotypes. In general, landraces and modern cultivars clustered separately with PH, PL, D_EH_, cPAR_EH_ and DM_A_ being higher in landraces and yield, HI, D_HA_, D_AM_, cPAR_HA_, cPAR_AM_, and RUE_EA_ being clearly superior in modern cultivars. However, for the remaining traits a range of variation existed within both types of germplasm, which could be a possible basis for selecting landraces carrying interesting traits expressed at high enough levels to justify their use as “donor” for such traits in breeding programs. For example, the Spanish “Enano de Andujar” and the Greek “Greece 14” and “Greece 23” could be used in breeding programs to increase grain weight, while the Tunisian “Hamira” and the Spanish “Colorado de Jerez” could be putatively used as source to enhance CTD (Supplementary Table 1). The bi-dimensional cluster also allowed the identification of genotypes showing close similarities based on the phenotypical traits analyzed. Although a trend to separate genetic subpopulations according to phenotypic data was observed in this cluster, there was not a perfect matching between the genetic structure of the population and its agronomic performance. In several cases landraces and modern cultivars with very high similarity in their agronomic performance were not classified in the same genetic subpopulation (see below) as defined by clustering based on markers, suggesting a weak relationship between overall agronomic performance and marker-based genetic similarities. Previous studies have reported that the association between genetic and agronomical or morpho-physiological traits was generally weak in durum wheat (Annicchiarico et al., 2009; Royo et al., 2010; Marzario et al., 2018; Sareen et al., 2020). However, exceptions to this general trend can be found. The phenotypic similarity between the old Italian founder cultivar “Capeiti” and the modern Syrian cultivar “Omrabi 3” (both tall types) supports the results of a previous study showing both genetic and phenotypic closeness between them (Royo et al., 2010). The phenotypic similarity between the Canadian modern cultivar “Macoun” and the Spanish landrace “Entrelargo de Montijo” suggests that this landrace could be one of the Mediterranean wheats introduced in USA at the end of the nineteen-century, likely contributing to the pedigree of “Mindum” (Royo et al., 2009), potentially being a “Macoun” ancestor. Phenotypical similarities can be a way to document the use of Mediterranean landraces by the North-American durum wheat breeding programs.

The analysis of the correlation coefficients between traits showed a similarity in landraces and modern cultivars regarding the negative effect of late heading in HI, as reported in previous studies (Villegas et al., 2016). This suggests that late heading genotypes reduced the accumulation of photosynthates in the grain, probably due to the declining of current photosynthesis resulting for increasing drought and heat stress as reported for Mediterranean environments (Papakosta and Gagianas, 1991; Blum, 1998). The negative relationships between NGS and W and GFR and the dependence of W on GFR have been described previously in durum wheat (Motzo et al., 1996; Arjona et al., 2020). Grain weight (W) and GFR were the traits that mostly influenced the yield of landraces, likely due to compensate for their lower number of grains per unit area when compared with modern cultivars (Royo et al., 2007). On the contrary, the strong relationship between HI and yield in modern cultivars is in agreement with the largest sink capacity of semi-dwarf cultivars compared with the landraces and their superior capacity for partitioning assimilates to the grain (Álvaro et al., 2008; Addisu et al., 2010; Rebetzke et al., 2012). Late-heading modern cultivars increased CTD_A_ (i.e., reduced their canopy temperature) more than the early-heading ones, which could result from higher transpiration rates associated with a larger potential evapotranspiration (Royo et al., 2002). In contrast, canopy temperature of late-heading landraces was higher than in the early-heading ones, which could be related to their better adaptation to environments with high evapotranspiration before anthesis and their limited capacity to respond to water availability during grain filling (Subira et al., 2015). However, this hypothesis would need further investigation.

A more granular subdivision, beyond landraces *vs*. modern cultivars, was made by analyzing the genetic structure of the panel, using 1,695 SNP markers, all showing <25% missing data, all having a minor alleles frequency of at least 10% and showing a PIC value >0.3 (Soriano et al., 2021). The analysis resulted in the unambiguous classification of 120 genotypes from the original panel of 372 into 5 genetically distinct subpopulations (SP 1 to 5), with each genotype having a coefficient of membership (*q*) to a particular SP > 0.8. While the classification was, for the great majority of the 120 genotypes, consistent with their geographical or breeding origin, it yielded some apparent incongruences. The Italian landrace “Aziziah 17/45” was classified in the middle-eastern group SP1, instead of logically be included is SP3 with most of the Italian landraces. However, it was developed in Italy from an early maturing pure line selected from Syrio-Palestinian landraces (Scarascia Mugnozza, 2005), which accounts for its classification among eastern Mediterranean types. Information on “IG-83920,” the second Italian landrace contained in SP1 has not been found, but its genetic similarity with landraces of SP1 suggests that it may have retained part of the genetic background of ancestral wheats from the east of the Mediterranean Basin. The Canadian cultivar “Macoun,” which was included in SP3, was released in Canada in 1974. It derived from the cross RL3607/DT182 (De Pauw and Hunt, 2001), both having in their pedigree the old variety “Mindum” (Clarke, 2005) that was released in the US in 1917 as a descendent of the Mediterranean wheats introduced in the country by the USDA in 1850 (Royo et al., 2009). Given this historical information and the very high membership coefficient of “Macoun” to SP3 (*q* = 0.999), it is conceivable that it retained a large part of the genetic background from western Mediterranean landraces. Finally, the French cultivar “Auroc” was classified in SP5, along with the US and Canadian modern cultivars. No information could be obtained to clearly explain this apparent geographical discrepancy, but it is conceivable that it may have a north American parent in its ancestry. Some of these unexpected clustering could be also due to errors in accession identification and labeling.

This analysis further subdivided the landraces group into three SPs, with SP1 corresponding to landraces from the eastern Mediterranean, in or around durum wheat's center of origin, SP2 including landraces from the northern Mediterranean coast and SP3 representing the western Mediterranean region. These results agree with a recent study involving a worldwide durum wheat collection (Mazzucotelli et al., 2020). Using a Ward clustering of the landraces the authors found different groups from the Fertile Crescent: North of Africa and South of Europe. Other studies carried out in durum wheat with the same worldwide panel classified the Mediterranean landraces in two groups involving eastern and western Mediterranean landraces (Maccaferri et al., 2019). The modern cultivars were subdivided into two SPs, SP4 corresponding to the CIMMYT/ICARDA-derived germplasm, spanning multiple countries in four continents, and SP5 including the mostly tall, photoperiod sensitive cultivars from the US upper Midwest and Canada. Using the worldwide germplasm panel Kabbaj et al. (2017) were able to separate CIMMYT and ICARDA germplasm. The population structure of Mazzucotelli et al. (2020) separated North-American modern cultivars from those derived from CIMMYT that were in turn divided from the south European modern cultivars + ICARDA germplasm that constituted a third subpopulation within the modern germplasm.

The information obtained from the analysis of the relationship between the genetic structure of the sub-panel and its agronomic performance could be interpreted not only in biological, but also in evolutionary terms. The three genetic SPs including Mediterranean landraces nicely corresponded with the wheat domestication Middle eastern region (SP1) and with the regions surrounding the Mediterranean Basin that wheat colonized during its migration westward (SP2 and SP3). SP2 represent landraces established along the migration path that wheat took before entering the Iberian Peninsula from the north, about 7,000 years BP, after spreading through Turkey and the Balkan Peninsula (Feldman, 2001). A second migration route involves the introduction of wheat to the Maghreb countries from Italy (Feldman, 2001), from where it likely migrated to Spain from the south (Moragues et al., 2007), which is consistent with the inclusion of accessions from Italy, Tunisia, Algeria, Morocco, and Spain in SP3. The analysis of the agronomical differences between SP retaining ancient genetic background of eastern, northern, and western Mediterranean regions can be useful to identify adaptive changes during the migration of durum wheat from its domestication area to the west of the Mediterranean Basin. Comparing the overall performance of landraces from northern (SP2) and western (SP3) Mediterranean areas with that of SP1 from which they are likely to have evolved, the main differences consisted of a reduction in the grain-filling period, NS and HI, with a concomitant increase in D_EH_, GFR, GA_90d_, PH, PL and DM_A_. Given that the eastern zone is the warmest and driest within the Mediterranean Basin (Royo et al., 2014), these evolutionary changes may have resulted from the adaptation of wheat to colder and wetter environments during its migration process. Kato and Yokoyama (1991) related the heading time of landraces with their geographical origin. Previous studies have demonstrated that landraces from cold and wet areas have later heading, less spikes, heavier grains, higher GFR, taller plants and superior yields than those originating in arid areas (Royo et al., 2014), with some of these differences associated with contrasting allele frequencies for NS, NGS and D_AM_ (Soriano et al., 2018). The higher spike number and lighter grains of SP1 when compared with SP2 and SP3 is consistent with the reported influence of the environmental conditions on the contribution of each component to the final yield of durum wheat. It has been demonstrated that durum wheat yield under warm and dry Mediterranean environments is achieved mostly by spike number, while grain weight predominantly influences yield in cool and wet environments (García del Moral et al., 2003; Royo et al., 2006). The more aggressive early soil coverage observed in landraces from SP2 and SP3, revealed by their higher GA_90d_ in the present study, may have also resulted from adaptation to colder and wetter environments and is consistent with their greater aboveground biomass at anthesis compared to landraces from SP1. Early soil coverage is considered to contribute to increase wheat yield in drought-prone environments because it minimizes evaporation of moisture from the soil surface (Botwright et al., 2002), maximizes the soil water available for transpiration and growth (López-Castañeda and Richards, 1994), reduces weeds competition (Coleman et al., 2001) and increases early root growth (Liao et al., 2006). For this reason, northern and western Mediterranean landraces may have breeding interest. A previous study comparing morpho-physiological traits of Tunisian and Algerian landraces with landraces from Syria and Jordan found that the latter group showed greater earliness, longer grain filling period, shorter stature and lower early growth vigor (Annicchiarico and Pecetti, 1995), in agreement with the results of the current study. The superior yield, CTD_A_, earlier flowering and shorter plants of landraces endemic of western Mediterranean environments compared to those originated in the northern countries may be signs of adaptation to the higher temperatures and radiation and lower rainfall pattern characteristics of this area of adaptation (Royo et al., 2014). The heavier grains of landraces from western Mediterranean countries, contributing to their higher yields could also be mediated by local human selection.

The two SPs involving modern cultivars (SP4 and SP5) include two of the most widely and currently grown genetic pools of spring durum wheat (Royo et al., 2009). While these germplasm pools are too recent to be discussed in evolutionary terms, comparing the agronomic performance of improved cultivars provides relevant information on the history and impact of durum wheat breeding. This study showed that the North-American germplasm pool (SP5) was genetically and agronomically close to eastern Mediterranean landraces (SP1). This finding agrees with the high gene-flow recently found between these seemingly unrelated SPs (Soriano et al., 2021) and the large influence of a relatively small number of plant introductions from Mediterranean countries and Russia on the genetic base present in modern US and Canadian cultivars (Joppa and Williams, 1988). Differences in the agronomic performance of modern North-American (SP5) and the CIMMYT/ICARDA germplasm pools (SP4) may be related to their contrasting pattern of adaptation. Whereas the CIMMYT/ICARDA breeding programs focus on developing germplasm with wide adaptation, the North-American germplasm is more specifically adapted to the lower temperature regimes and the very short growing seasons of the main wheat growing areas in northern latitudes (De Pauw and Hunt, 2001). In addition, while the CIMMYT/ICARDA programs tend to exclude strong or any level of photoperiod sensitivity, the North-America programs by enlarge keep high levels of sensitivity as it gives them an adaptive advantage at high latitudes. These different breeding strategies are apparent in the present study based on the longer time to heading and to anthesis characterizing SP5 compared to SP4, which is probably related to their different levels of photoperiod sensitivity (Royo et al., 2020). SP5 showed a different yield formation strategy than SP4, which led to a lower average yield under the conditions of the present study. The high number of grains per spike has been reported to be the main driver of yield increases in Canadian durum wheats (McCaig and Clarke, 1995). In the current study, the average NGS for SP5 was indeed higher than that of SP4 but the lower average grain weight and harvest index characterizing SP5 down-compensated for NGS, ultimately resulting a lower average yield compared to SP4.

The large number of accessions that classified within the CIMMYT/ICARDA genetic background (SP4) was not unexpected given the massive adoption of the germplasm developed in these international centers worldwide. Pfeiffer and Payne (2005) reported that more than 90% of durum wheat varieties released in developing countries during the 1990's had CIMMYT genetic background. More recently, Lantican et al. (2016) reported that between 65 and 94% (depending on the region) of the durum cultivars released worldwide between 1994 and 2014 were either CIMMYT/ICARDA straight introductions or lines from crosses made locally using CIMMYT/ICARDA parents. The enormous impact in Spain of CIMMYT durum wheat germplasm starting in the 1970s (Royo and Briceño-Félix, 2011) supports the inclusion of many Spanish modern cultivars in this SP. Modern germplasm from Morocco and Tunisia is also largely represented in SP4, reflecting the substantial impact of CIMMYT-related genetics, derived from the joined CIMMYT/ICARDA breeding program for the North African countries since the mid-1980's (Belaid, 2000). The contribution of CIMMYT germplasm to other countries such Argentina, Italy and France has been also documented (Maccaferri et al., 2005; Roncallo et al., 2019). Modern cultivars retaining CIMMYT/ICARDA genetic background were the most agronomically distant from landraces, which is consistent with the low gene-flow detected between these two germplasm pools (Soriano et al., 2021) and the genetic distance existing between CIMMYT-derived founders and the old Mediterranean landraces (Maccaferri et al., 2003). The agronomic characteristics observed in SP4 in the current study are in general agreement with those reported previously. The high yield, NGS, HI and shorter plant characteristics of CIMMYT germplasm have been related to the introgression of the *Rht-B1b* dwarfing gene in the early days of the breeding program (Pfeiffer and Payne, 2005). Grain yield progress in CIMMYT germplasm has been attributed to a linear increase in the number of grains per unit area, via an augmented number of spikes per unit area and/or grains per spike (Waddington et al., 1987; Pfeiffer et al., 2000). We have not found any previous study underlining the high RUE before anthesis of the CIMMYT/ICARDA genetic pool. Our results suggest that even though the early anthesis of this germplasm pool constrained the amount of light absorbed by the crop canopy prior to anthesis, it was efficiently used to produce biomass which, in turn, was highly efficient in converting assimilates into grain.

A GWAS allowed the identification of molecular markers associated with the agronomic traits analyzed in the current study. As previously reported by Soriano et al. (2021) LD decay was used to integrate all the MTAs into QTL hotspots. Sixty-four out of 144 QTL hotspots shared genetic positions with selective sweeps detected by Soriano et al. (2021) by eigenGWAS. They included nine of the hotspots showing haplotype differences among SPs, which agrees with the selection of these genomic regions during durum wheat breeding (Supplementary Table 4). According to the genomic location defined by Liu et al. (2019) some important genes are known to co-locate within the QTL hotspots identified in the present study. These include the photoperiod sensitivity genes *Ppd*-A1 and *Ppd*-B1, the vernalization response genes *Vrn*-A1 and *Vrn*-B3, and the dwarfing genes *Rht*-B1, *Rht12* and *Rht25*. The incorporation of favorable alleles at these loci by breeding programs shortened the cycle length of durum wheat (Royo et al., 2020). The incorporation of dwarfing genes in durum wheat was associated with yield increases up to 35% (Royo et al., 2009). Four genes included in hotspots on chromosomes 2A, 2B and 7A were involved in grain weight: *TaSus2*-2A (Jiang et al., 2015), *TaSST-A2* (Dong et al., 2016) and *TaTEF*-7A (Zheng et al., 2014), and size *TaGS2*-B1 (Zhang et al., 2014). Other genes with implication on yield were *TaALP*-4A (Chen et al., 2016), involved in preharvest sprouting tolerance, found in the hotspot 4A.9. The gene *TaCOMT*-3B (Fu et al., 2018) is involved in lignin content and contributes to lodging tolerance, and the gene *1-FEH-w3* (Zhang et al., 2015) was found to participate in water soluble carbohydrates mobilization under drought. Other genes found within QTL hotspots were involved in grain quality. Among these genes, *Glu*-A1 was located in hotspot 1A.6. According to Subirà et al. (2014) the improvement of pasta making quality in modern durum wheat cultivars was caused by the introgression of favorable alleles for HMW and LMW glutenin subunits. Other important genes for quality within QTL hotspots were related to polyphenol oxidase (PPO) activity, genes *Ppo*-A1 (chromosome 2A) and *Ppo*-B2 (chromosome 2B); peroxidase activity, the *Pod*-A1 gene on chromosome 3A; grain protein content (*GPC*-B1) on chromosome 6B; Finally, the phytoene synthase gene *Psy-*B1 located in QTL hotspot 7B.11 involved in the biosynthesis of carotenoid pigments, the biochemical basis of yellow color in durum wheat. The identification of functional genes incorporated in the modern durum wheat cultivars suggest these QTL hotspots were target genomic regions during selection in modern breeding programs.

Moreover, within the QTL hotspots, HBs were defined to search for haplotype patterns differentiating the SPs. The use of specific haplotypes affecting agronomic performance will allow the selection of the most favorable allelic combinations for durum wheat breeding in Mediterranean environments. Landraces showed haplotypes on chromosomes 5A and 7A, associated with decreased yield. The different haplotype variants identified in HB4 and HB9 in hotspot 7A.4 could be associated with the greater NGS of modern cultivars compared to that of the landraces. HB2 and HB5 in hotspot 3A.1 could be associated with the greater values for NGS and the lowest values for D_EH_, D_HA_, PL and PH found in cultivars developed from CIMMYT/ICARDA. Among the landrace SPs, the Western Mediterranean landraces (SP3) showed haplotypes associated with higher grain weight in HB6 hotspot 2A.2 and HB1 and HB2 in hotspot 7A.4, where known loci increasing grain weight were found, namely, *TaSus2*-2A and *TaTEF*-7A, respectively. A previous study reported higher grain weight in western durum wheat Mediterranean landraces (Soriano et al., 2018). Finally, alleles linked to higher HI were found in SP4 and SP5 and differences were identified in HB4 and HB9 in hotspot 7A.4. The late heading and short grain-filling period characteristic of landraces of SP2 and SP3 could be related to HB5 and HB6 in hotspot 7B.5. Western Mediterranean landraces showed haplotypes increasing plant height and peduncle length, whereas east Mediterranean landraces and modern cultivars seem to have haplotypes associated with shorter plant and peduncles. These height-related haplotype differences were from QTL hot spots 7A.4 and 3A.1, therefore unrelated to the known dwarfing gene *Rht-B1* located on chromosome 4B and representing alternative genetic basis underlying variability in height and peduncle length. Western Mediterranean landraces had haplotypes associated with enhanced early growth (hotspots 3A.1, 3A.3, 4A.3, 6A.10 and 7B.5) and increased biomass at maturity (2B.4, 4A.2 and 5A.5). Western Mediterranean landraces showed haplotypes associated with lower cPAR from heading to anthesis in hotspots 7B.3 and 7B.11, whereas northern landraces had haplotypes decreasing cPAR at maturity both eastern and western Mediterranean landraces harbored alleles increasing the latter parameter (hotspots 3A.1, 7A.4 and 7B.11). Among these hotspots affecting PAR, known loci for grain weight (*TaTEF*-7A) and vernalization requirements (*Vrn*-B3) were found in 7A.4 and 7B.11, respectively.

## Conclusions

The conjunction of a profuse molecular analysis with a comprehensive phenotyping of the germplasm panel allowed a reliable distinction of genetic groups and the identification of phenotypic traits differentiating landraces from modern cultivars and among genetic subpopulations. Except for the number of spikes and grain weight, the remainder 19 phenotypic traits assessed in the current study properly differentiated old germplasm from the improved one. Landraces from different Mediterranean geographical regions could be distinguished by their different NS, GFR, PH and DM_A_, traits different than the ones separating the two subpopulations involving modern cultivars: yield, HI, grain weight, phenology, PL, PH, CTD_A_, and CPAR_EH_. Landraces from the east of the Mediterranean Basin were the most phenotypically similar to modern cultivars, particularly the North-American ones, but yield and days to heading allowed distinguishing both subpopulations.

At molecular level, the identification of different haplotypes among SPs affecting agronomic performance within QTL hotspots will be of special interest for designing future breeding lines through MAS including germplasm from different Mediterranean regions.

## Data Availability Statement

The original contributions presented in the study are included in the article/Supplementary Material, further inquiries can be directed to the corresponding author/s.

## Author Contributions

CR and JS conceived the project and carried out the statistical analyses and outlined the manuscript. CR, DV, and KA assembled and purified the germplasm collection. CR and DV performed field evaluations. JS performed molecular analyses. All authors wrote the manuscript and approved the final version.

## Conflict of Interest

The authors declare that the research was conducted in the absence of any commercial or financial relationships that could be construed as a potential conflict of interest.

## Supplementary Material

Supplementary Figure 1 Monthly water input and maximum (red line), mean (green line) and minimum (orange line) temperatures during the growth cycle of each crop season. The lowest figures indicate the water soil content at three depths (0–40, 10–40, and 25–40 cm) for each year.

Supplementary Figure 2 Number of landraces (green) and modern cultivars (red) in each range of values for the agronomic traits analyzed.

Supplementary Figure 3 Correlations between the 21 phenotypic traits in landraces (*n* = 172) and modern cultivars (*n* = 200). Positive and negative correlation coefficients (r) are indicated in red and blue colors, respectively, with the color intensity associated to the values. *P* < 0.05 for 0.14 > *r* < 0.19; *P* < 0.01 for 0.19 > *r* < 0.24; *P* < 0.001 for *r* > 0.24.

Supplementary Table 1 Genotypes included in the study with their genetic structure coefficients (*q*).

Supplementary Table 2 Mean values across years of the studied traits for each genotype.

Supplementary Table 3 GWAS results.

Supplementary Table 4 QTL hotspots.
