# Supplementary figures and images for: Agronomic, Physiological and Genetic Changes Associated With Evolution, Migration and Modern Breeding in Durum Wheat
Source: Front Plant Sci. 2021 Jul 8;12:674470. doi: 10.3389/fpls.2021.674470 (PMC8296143; doi:10.3389/fpls.2021.674470)

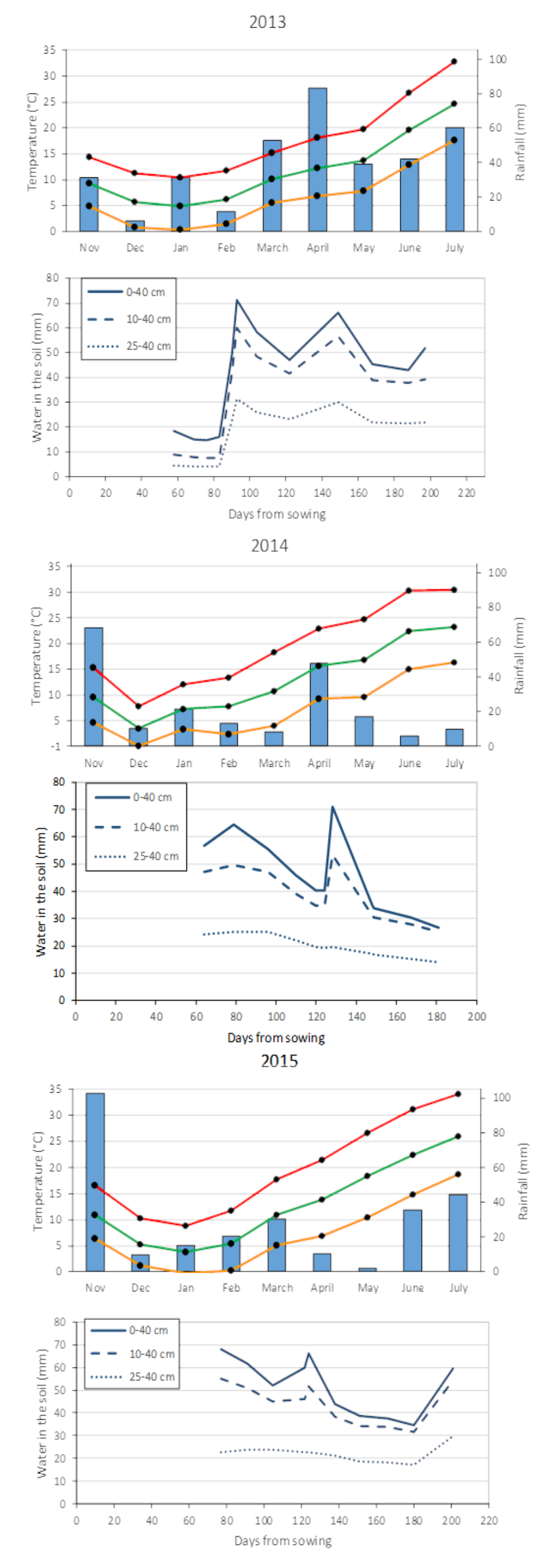

Supplement: Supplementary Figure 1 — Monthly water input and maximum (red line), mean (green line) and minimum (orange line) temperatures during the growth cycle of each crop season. The lowest figures indicate the water soil content at three depths (0–40, 10–40, and 25–40 cm) for each year. [file Image_1.TIF]

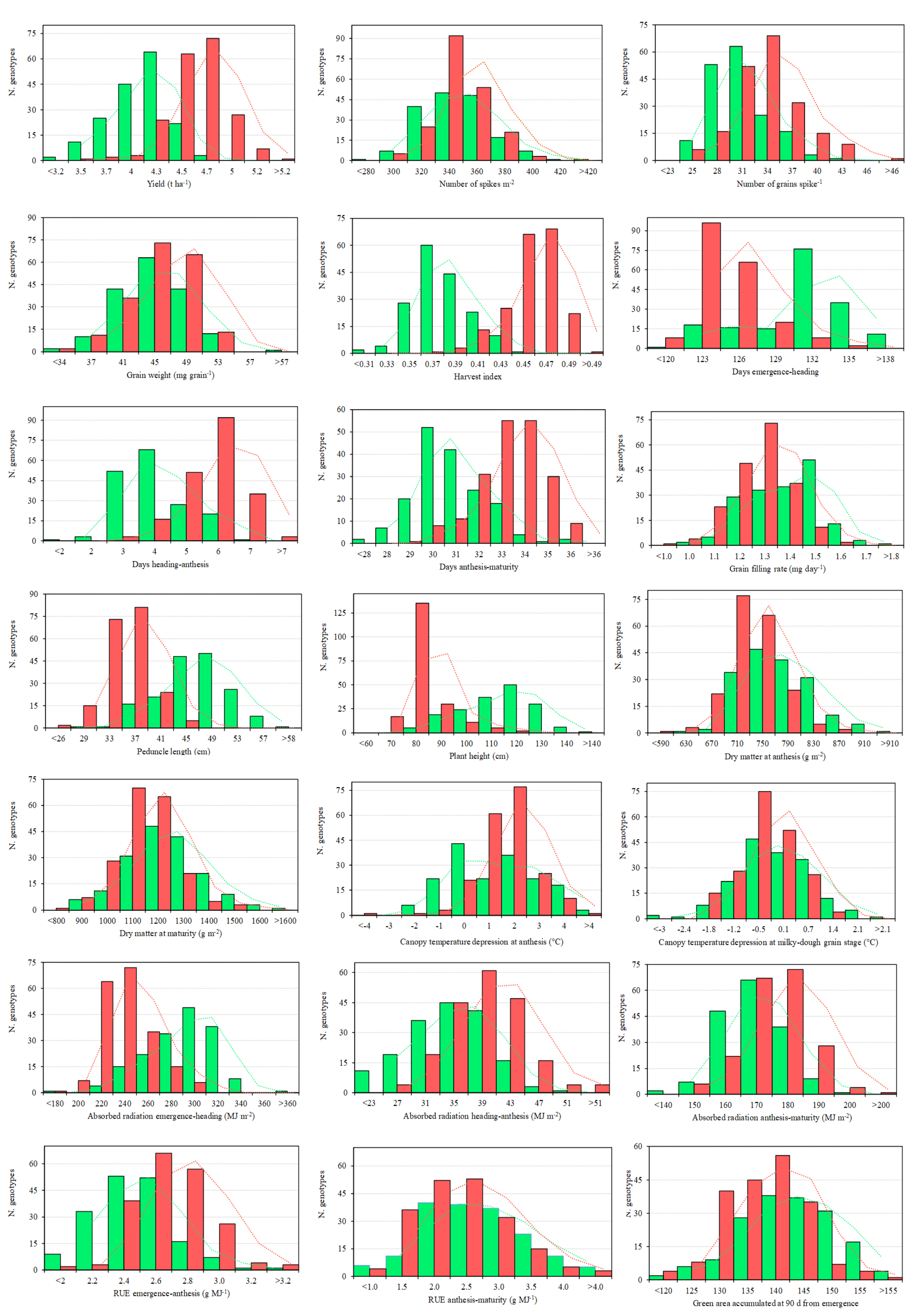

Supplement: Supplementary Figure 2 — Number of landraces (green) and modern cultivars (red) in each range of values for the agronomic traits analyzed. [file Image_2.tif]

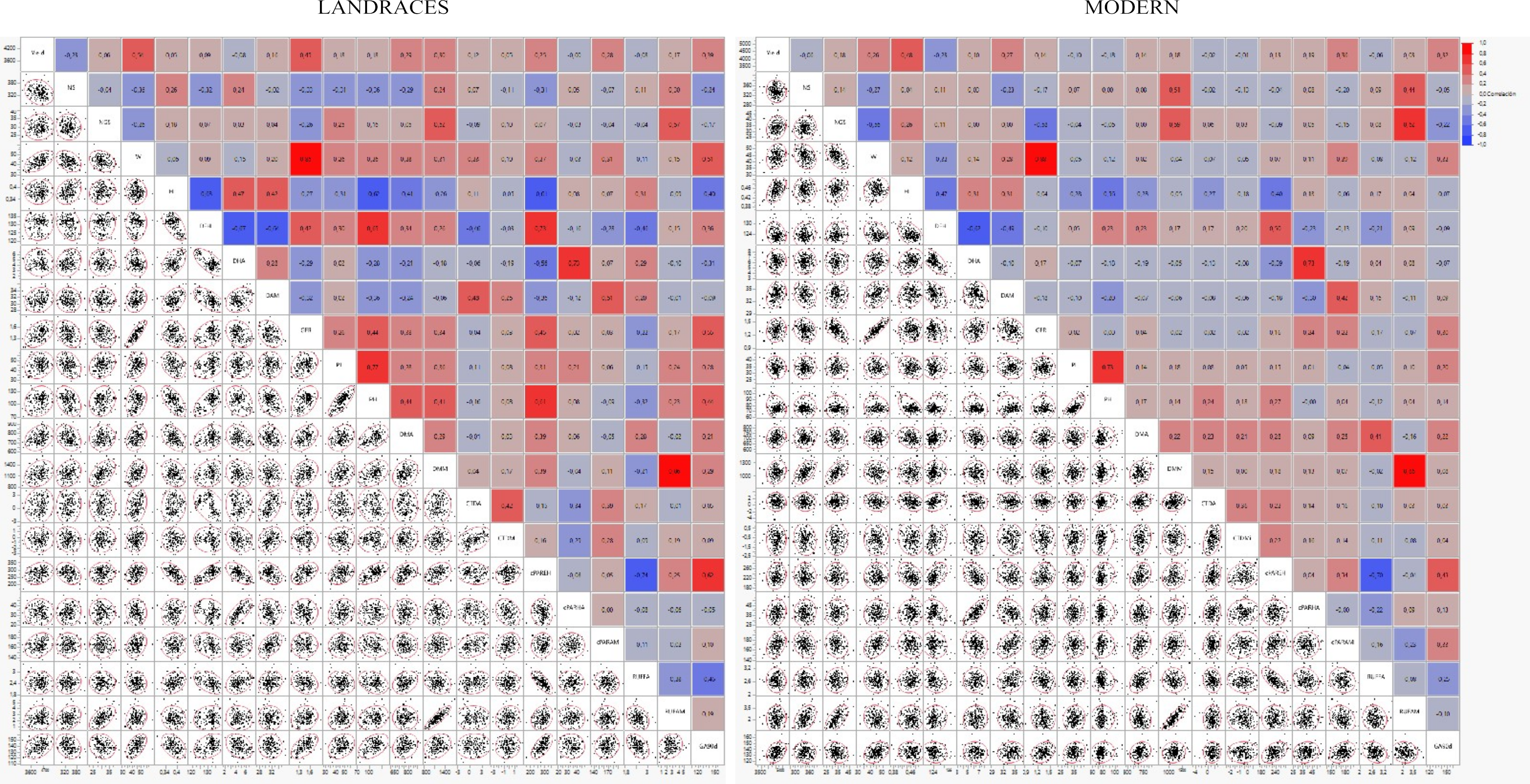

Supplement: Supplementary Figure 3 — Correlations between the 21 phenotypic traits in landraces (n = 172) and modern cultivars (n = 200). Positive and negative correlation coefficients (r) are indicated in red and blue colors, respectively, with the color intensity associated to the values. P < 0.05 for 0.14 > r < 0.19; P < 0.01 for 0.19 > r < 0.24; P < 0.001 for r > 0.24. [file Image_3.TIF]
