# Supplementary material for: Agronomic, Physiological and Genetic Changes Associated With Evolution, Migration and Modern Breeding in Durum Wheat
Source: Front Plant Sci. 2021 Jul 8;12:674470. doi: 10.3389/fpls.2021.674470 (PMC8296143; doi:10.3389/fpls.2021.674470)
Supplement: Supplementary Table 1 — Genotypes included in the study with their genetic structure coefficients (q). [file Data_Sheet_1.docx]

**Supplementary TABLE S1 ǀ** Genotypes included in the study with their genetic structure coefficients (*q*).

| **Cultivar** | **Type** | **Country** | **Code** | ***q1*** | ***q2*** | ***q3*** | ***q4*** | ***q5*** |
| --- | --- | --- | --- | --- | --- | --- | --- | --- |
| Dur de Medeah | Landrace | Algeria | BGE020915 | 0.730 | 0.001 | 0.002 | 0.006 | 0.261 |
| IG-94009 | Landrace | Algeria |  | 0.646 | 0.001 | 0.004 | 0.057 | 0.293 |
| IG-92895 | Landrace | Algeria |  | 0.466 | 0.002 | 0.056 | 0.004 | 0.473 |
| IG-92967 | Landrace | Algeria |  | 0.443 | 0.003 | 0.001 | 0.016 | 0.537 |
| IG-93621 | Landrace | Algeria |  | 0.310 | 0.002 | 0.002 | 0.028 | 0.658 |
| IG-93030 | Landrace | Algeria |  | 0.005 | 0.002 | 0.001 | 0.001 | 0.991 |
| Tchirpan | Landrace | Bulgaria | IPGR | 0.586 | 0.018 | 0.053 | 0.266 | 0.077 |
| Zagorka | Landrace | Bulgaria |  | 0.556 | 0.039 | 0.008 | 0.297 | 0.100 |
| Lozen 76 | Landrace | Bulgaria | IPGR | 0.403 | 0.002 | 0.095 | 0.090 | 0.410 |
| IG-96851 | Landrace | Crete |  | 0.626 | 0.014 | 0.020 | 0.220 | 0.120 |
| IG-96802 | Landrace | Crete |  | 0.246 | 0.005 | 0.004 | 0.112 | 0.633 |
| 440-IX/96 | Landrace | Croatia | PI-345441 | 0.895 | 0.010 | 0.001 | 0.001 | 0.093 |
| 441-IX/97 | Landrace | Croatia | PI-345442 | 0.883 | 0.005 | 0.000 | 0.001 | 0.111 |
| Dalmatia 3 | Landrace | Croatia | Citr-11246 | 0.840 | 0.015 | 0.001 | 0.029 | 0.115 |
| Dalmatia 1 | Landrace | Croatia | Citr-11245 | 0.805 | 0.057 | 0.001 | 0.065 | 0.072 |
| Mishriki | Landrace | Egypt | PI-7016 | 0.631 | 0.002 | 0.127 | 0.225 | 0.015 |
| Milagro | Landrace | Egypt | BGE000168 | 0.582 | 0.043 | 0.003 | 0.002 | 0.371 |
| Reading | Landrace | Egypt | BGE012451 | 0.541 | 0.000 | 0.016 | 0.128 | 0.314 |
| 1P1 | Landrace | Egypt | PI-113397 | 0.534 | 0.098 | 0.017 | 0.350 | 0.001 |
| 5P4 | Landrace | Egypt | PI-113395 | 0.519 | 0.045 | 0.069 | 0.344 | 0.024 |
| Girgeh | Landrace | Egypt | PI-7422 | 0.497 | 0.002 | 0.103 | 0.397 | 0.001 |
| 28 | Landrace | Egypt | PI-60726 | 0.477 | 0.152 | 0.088 | 0.280 | 0.003 |
| MG 26429 | Landrace | Egypt | PI-576803 | 0.475 | 0.111 | 0.017 | 0.396 | 0.001 |
| 2751 | Landrace | Egypt | PI-559973 | 0.471 | 0.038 | 0.062 | 0.428 | 0.001 |
| D-2 | Landrace | Egypt | PI-366109 | 0.468 | 0.055 | 0.044 | 0.432 | 0.001 |
| 31 | Landrace | Egypt | PI-60727 | 0.459 | 0.115 | 0.198 | 0.206 | 0.022 |
| Giza 2 | Landrace | Egypt | PI-434894 | 0.449 | 0.002 | 0.096 | 0.369 | 0.085 |
| Sinai No.8 | Landrace | Egypt | PI-60742 | 0.318 | 0.001 | 0.003 | 0.672 | 0.007 |
| Beladi Rouge | Landrace | France | PI-174599 | 0.750 | 0.005 | 0.065 | 0.105 | 0.075 |
| De Santa Marta | Landrace | France | PI-174620 | 0.730 | 0.001 | 0.061 | 0.009 | 0.198 |
| Rubio enlargado d’Atlemteje | Landrace | France | PI-48212 | 0.695 | 0.001 | 0.002 | 0.039 | 0.264 |
| Tounse | Landrace | France | PI-174697 | 0.625 | 0.014 | 0.006 | 0.165 | 0.189 |
| Iumillo | Landrace | France | PI-174662 | 0.574 | 0.001 | 0.207 | 0.215 | 0.002 |
| Trigo Glutinoso | Landrace | France | PI-174699 | 0.546 | 0.078 | 0.005 | 0.147 | 0.224 |
| Mavraani | Landrace | Greece | PI-43245 | 0.755 | 0.002 | 0.147 | 0.091 | 0.005 |
| Greece 24 | Landrace | Greece |  | 0.748 | 0.003 | 0.004 | 0.150 | 0.095 |
| Rapsani | Landrace | Greece | PI-43246 | 0.686 | 0.001 | 0.024 | 0.109 | 0.180 |
| Greece 14 | Landrace | Greece |  | 0.596 | 0.001 | 0.001 | 0.004 | 0.398 |
| Greece 23 | Landrace | Greece |  | 0.590 | 0.001 | 0.001 | 0.004 | 0.404 |
| Juljulith | Landrace | Israel | PI-292034 | 0.601 | 0.001 | 0.101 | 0.294 | 0.003 |
| Abu Fashit | Landrace | Israel | PI-384037 | 0.549 | 0.002 | 0.028 | 0.420 | 0.002 |
| JM-3989 | Landrace | Israel | PI-572903 | 0.496 | 0.001 | 0.048 | 0.455 | 0.001 |
| JM-3987 | Landrace | Israel | PI-572901 | 0.476 | 0.003 | 0.080 | 0.440 | 0.001 |
| Hati | Landrace | Israel | PI-52503 | 0.159 | 0.009 | 0.046 | 0.784 | 0.001 |
| Etith | Landrace | Israel | PI-292032 | 0.001 | 0.001 | 0.000 | 0.998 | 0.000 |
| Razza 181 | Landrace | Italy | BGE012498 | 0.747 | 0.001 | 0.001 | 0.002 | 0.250 |
| Cicirelo | Landrace | Italy | IG-82555 | 0.738 | 0.001 | 0.120 | 0.140 | 0.001 |
| Balilla Falso | Landrace | Italy | BGE012340 | 0.697 | 0.006 | 0.005 | 0.233 | 0.059 |
| Milazzo | Landrace | Italy | BGE012487 | 0.639 | 0.001 | 0.002 | 0.001 | 0.357 |
| IG-83905 | Landrace | Italy |  | 0.605 | 0.007 | 0.181 | 0.194 | 0.014 |
| Carlantino | Landrace | Italy | IG-82551 | 0.522 | 0.003 | 0.005 | 0.005 | 0.464 |
| IG-83920 | Landrace | Italy |  | 0.021 | 0.001 | 0.071 | 0.907 | 0.000 |
| Hymera | Landrace | Italy | BGE002876 | 0.003 | 0.001 | 0.000 | 0.407 | 0.589 |
| Capeiti 8 | Landrace | Italy |  | 0.001 | 0.013 | 0.001 | 0.401 | 0.584 |
| Trinakria | Landrace | Italy | BGE002893 | 0.001 | 0.003 | 0.001 | 0.285 | 0.710 |
| Carlo jucci | Landrace | Italy | BGE000097 | 0.001 | 0.000 | 0.000 | 0.206 | 0.792 |
| Razza 208 | Landrace | Italy | BGE012330 | 0.001 | 0.000 | 0.000 | 0.006 | 0.992 |
| Razza 96 | Landrace | Italy | BGE020912 | 0.001 | 0.000 | 0.000 | 0.000 | 0.999 |
| Senatore Capelli | Landrace | Italy | BGE000119 | 0.000 | 0.000 | 0.000 | 0.000 | 0.999 |
| Capeiti | Landrace | Italy | BGE020911 | 0.000 | 0.000 | 0.000 | 0.453 | 0.546 |
| Aziziah 17/45 | Landrace | Italy/Syria | BGE012328 | 0.000 | 0.000 | 0.000 | 0.999 | 0.000 |
| Salti na Zinia | Landrace | Jordan | PI-223169 | 0.587 | 0.002 | 0.018 | 0.338 | 0.055 |
| Safra Jerash | Landrace | Jordan | PI-210885 | 0.213 | 0.045 | 0.020 | 0.677 | 0.046 |
| 26 | Landrace | Jordan | PI-420946 | 0.060 | 0.001 | 0.004 | 0.934 | 0.002 |
| Zoghbiyeh Safra | Landrace | Jordan | PI-283155 | 0.020 | 0.001 | 0.002 | 0.977 | 0.001 |
| Horani Howawi | Landrace | Jordan | PI-234382 | 0.001 | 0.001 | 0.000 | 0.997 | 0.001 |
| Zugbieh Sutra | Landrace | Jordan | PI-234388 | 0.001 | 0.000 | 0.000 | 0.998 | 0.000 |
| Safra Maan | Landrace | Jordan | PI-371833 | 0.001 | 0.000 | 0.000 | 0.998 | 0.000 |
| Harani Auttma | Landrace | Jordan | PI-223156 | 0.000 | 0.000 | 0.000 | 0.999 | 0.000 |
| 9918 | Landrace | Lebanon | PI-182666 | 0.493 | 0.001 | 0.041 | 0.435 | 0.029 |
| Reyati | Landrace | Lebanon | PI-51211 | 0.391 | 0.002 | 0.003 | 0.004 | 0.600 |
| IG-84856 | Landrace | Lebanon | IG-84856 | 0.199 | 0.010 | 0.034 | 0.305 | 0.452 |
| 9935 | Landrace | Lebanon | PI-182671 | 0.085 | 0.001 | 0.001 | 0.908 | 0.005 |
| Hourah | Landrace | Lebanon | PI-182674 | 0.015 | 0.000 | 0.004 | 0.981 | 0.000 |
| 9923 | Landrace | Lebanon | PI-182667 | 0.007 | 0.008 | 0.002 | 0.966 | 0.016 |
| 9929 | Landrace | Lebanon | PI-182669 | 0.000 | 0.000 | 0.000 | 0.999 | 0.000 |
| Tripshiro | Landrace | Libya | PI-54432 | 0.233 | 0.001 | 0.002 | 0.735 | 0.029 |
| 259-VII/12 | Landrace | Macedonia | PI-345260 | 0.806 | 0.001 | 0.124 | 0.068 | 0.001 |
| VII/13-X11 | Landrace | Macedonia | PI-362629 | 0.801 | 0.001 | 0.123 | 0.075 | 0.000 |
| II/4 | Landrace | Macedonia | PI-405908 | 0.800 | 0.001 | 0.101 | 0.098 | 0.000 |
| 196/71 | Landrace | Macedonia | PI-374658 | 0.795 | 0.001 | 0.136 | 0.068 | 0.000 |
| 248-VII/7 | Landrace | Macedonia | PI-345249 | 0.763 | 0.001 | 0.060 | 0.175 | 0.001 |
| 1640 | Landrace | Macedonia |  | 0.733 | 0.001 | 0.153 | 0.112 | 0.000 |
| II/10 | Landrace | Macedonia |  | 0.273 | 0.038 | 0.006 | 0.001 | 0.682 |
| 42 | Landrace | Montenegro | PI-435043 | 0.892 | 0.005 | 0.001 | 0.001 | 0.102 |
| 37 | Landrace | Montenegro | PI-435038 | 0.886 | 0.002 | 0.001 | 0.001 | 0.110 |
| 23 | Landrace | Montenegro | PI-435024 | 0.883 | 0.003 | 0.001 | 0.000 | 0.113 |
| 356-I/9 | Landrace | Montenegro | PI-345357 | 0.861 | 0.017 | 0.001 | 0.001 | 0.119 |
| 33 | Landrace | Montenegro | PI-435034 | 0.856 | 0.052 | 0.001 | 0.001 | 0.090 |
| Douro Boukowo | Landrace | Morocco |  | 0.862 | 0.001 | 0.014 | 0.122 | 0.001 |
| Cobros | Landrace | Morocco | PI-410918 | 0.683 | 0.001 | 0.002 | 0.179 | 0.136 |
| Zoco Yebel Hebil | Landrace | Morocco | BGE020916 | 0.599 | 0.009 | 0.007 | 0.190 | 0.196 |
| Maghoussa Amizmiz | Landrace | Morocco | PI-192639 | 0.532 | 0.001 | 0.001 | 0.092 | 0.375 |
| Du Maroc Battandier | Landrace | Morocco |  | 0.435 | 0.001 | 0.001 | 0.001 | 0.563 |
| Maghoussa | Landrace | Morocco | PI-192638 | 0.426 | 0.001 | 0.001 | 0.059 | 0.513 |
| Ble Dur 250 | Landrace | Morocco | PI-191029 | 0.402 | 0.001 | 0.002 | 0.001 | 0.594 |
| Merzaga | Landrace | Morocco | PI-192487 | 0.274 | 0.001 | 0.001 | 0.002 | 0.722 |
| Morocco | Landrace | Morocco | citr-6878 | 0.259 | 0.000 | 0.001 | 0.003 | 0.737 |
| Saffi | Landrace | Morocco | citr-6879 | 0.250 | 0.001 | 0.001 | 0.003 | 0.746 |
| Red Beard | Landrace | Morocco | PI-410922 | 0.051 | 0.001 | 0.000 | 0.025 | 0.923 |
| Oned Zenati | Landrace | Morocco | PI-192483 | 0.005 | 0.001 | 0.001 | 0.001 | 0.992 |
| Mahmoudi C | Landrace | Morocco | PI-192492 | 0.001 | 0.000 | 0.000 | 0.000 | 0.998 |
| Alentejo | Landrace | Portugal | BGE013723 | 0.840 | 0.044 | 0.000 | 0.001 | 0.115 |
| Dezassete | Landrace | Portugal | BGE012566 | 0.802 | 0.081 | 0.001 | 0.004 | 0.112 |
| Anafil | Landrace | Portugal | BGE012500 | 0.801 | 0.001 | 0.002 | 0.002 | 0.194 |
| Raposinho | Landrace | Portugal | BGE012247 | 0.732 | 0.000 | 0.001 | 0.034 | 0.232 |
| Lobeiro de grao escuro | Landrace | Portugal | BGE020909 | 0.687 | 0.001 | 0.001 | 0.077 | 0.235 |
| Durazio Rijo | Landrace | Portugal | BGE012485 | 0.675 | 0.001 | 0.001 | 0.016 | 0.307 |
| Tremes rijo | Landrace | Portugal | BGE019279 | 0.645 | 0.001 | 0.001 | 0.163 | 0.191 |
| Espanhol | Landrace | Portugal | BGE012522 | 0.575 | 0.001 | 0.000 | 0.036 | 0.388 |
| Amarelo Barba Preta | Landrace | Portugal | BGE013694 | 0.573 | 0.013 | 0.004 | 0.015 | 0.395 |
| Raspinegro | Landrace | Portugal | BGE012497 | 0.555 | 0.003 | 0.001 | 0.199 | 0.242 |
| Marques | Landrace | Portugal | BGE012379 | 0.548 | 0.001 | 0.002 | 0.138 | 0.311 |
| Caxudo de sete espigas | Landrace | Portugal | BGE013737 | 0.525 | 0.005 | 0.001 | 0.002 | 0.467 |
| Durazio Rijo Glabro | Landrace | Portugal | BGE013658 | 0.519 | 0.005 | 0.072 | 0.401 | 0.003 |
| Belgrade 9 | Landrace | Serbia | PI-278600 | 0.852 | 0.001 | 0.021 | 0.127 | 0.000 |
| 1575 | Landrace | Serbia | PI-378303 | 0.774 | 0.001 | 0.084 | 0.140 | 0.001 |
| 18/71 | Landrace | Serbia | PI-585195 | 0.648 | 0.001 | 0.007 | 0.249 | 0.095 |
| Gros de Cerdaña | Landrace | Spain | BGE019289 | 0.869 | 0.080 | 0.001 | 0.001 | 0.049 |
| Blanquillo | Landrace | Spain | BGE013729 | 0.868 | 0.083 | 0.001 | 0.001 | 0.047 |
| Griego de Baleares | Landrace | Spain | BGE018619 | 0.867 | 0.071 | 0.001 | 0.001 | 0.061 |
| Blanco de Corella | Landrace | Spain | BGE012534 | 0.858 | 0.016 | 0.001 | 0.003 | 0.122 |
| Pisana cañihueca | Landrace | Spain | BGE018621 | 0.854 | 0.084 | 0.001 | 0.001 | 0.060 |
| Heraldo del Rhin | Landrace | Spain | BGE013103 | 0.833 | 0.032 | 0.001 | 0.001 | 0.133 |
| Blanquillón de Boñar | Landrace | Spain | BGE018599 | 0.804 | 0.029 | 0.001 | 0.002 | 0.164 |
| Claro de Balazote | Landrace | Spain | BGE019281 | 0.784 | 0.001 | 0.002 | 0.001 | 0.212 |
| Rubio de Belalcázar | Landrace | Spain |  | 0.774 | 0.001 | 0.001 | 0.001 | 0.223 |
| Farto cañifino | Landrace | Spain | BGE013064 | 0.768 | 0.002 | 0.005 | 0.009 | 0.216 |
| Clarofino | Landrace | Spain |  | 0.762 | 0.002 | 0.002 | 0.001 | 0.233 |
| Basto Duro | Landrace | Spain | BGE013687 | 0.738 | 0.003 | 0.019 | 0.003 | 0.238 |
| Entrelargo de Montijo | Landrace | Spain | BGE013053 | 0.738 | 0.001 | 0.001 | 0.036 | 0.223 |
| Raspinegro Canario | Landrace | Spain | BGE012346 | 0.720 | 0.000 | 0.002 | 0.006 | 0.272 |
| Rubio de Montijo | Landrace | Spain | BGE018274 | 0.717 | 0.001 | 0.002 | 0.063 | 0.217 |
| Colorado de Jerez | Landrace | Spain | BGE018304 | 0.712 | 0.006 | 0.015 | 0.054 | 0.213 |
| Azulejo de Villa del Río | Landrace | Spain | BGE018605 | 0.692 | 0.000 | 0.002 | 0.026 | 0.280 |
| Raspinegro de Alcalá Guadaira | Landrace | Spain | BGE018337 | 0.661 | 0.001 | 0.001 | 0.001 | 0.337 |
| Pinet | Landrace | Spain | BGE018282 | 0.649 | 0.001 | 0.027 | 0.006 | 0.317 |
| Verdial | Landrace | Spain | BGE012381 | 0.644 | 0.002 | 0.010 | 0.053 | 0.290 |
| Semental | Landrace | Spain | BGE013617 | 0.592 | 0.001 | 0.002 | 0.053 | 0.351 |
| Blanco Verdeal | Landrace | Spain |  | 0.582 | 0.002 | 0.002 | 0.003 | 0.411 |
| Candeal de Salamanca | Landrace | Spain | BGE012392 | 0.543 | 0.001 | 0.455 | 0.001 | 0.000 |
| Alonso | Landrace | Spain | BGE013041 | 0.518 | 0.001 | 0.001 | 0.002 | 0.478 |
| Andalucía 344 | Landrace | Spain | BGE013056 | 0.518 | 0.001 | 0.001 | 0.004 | 0.477 |
| Fartó | Landrace | Spain | BGE013699 | 0.485 | 0.001 | 0.001 | 0.143 | 0.370 |
| Enano de Andújar | Landrace | Spain | BGE018298 | 0.452 | 0.005 | 0.005 | 0.002 | 0.536 |
| Arisnegro de Tenerife | Landrace | Spain | BGE018651 | 0.399 | 0.061 | 0.011 | 0.084 | 0.445 |
| Recio de Cañete | Landrace | Spain | BGE013611 | 0.229 | 0.001 | 0.001 | 0.002 | 0.768 |
| Blancal | Landrace | Spain | BGE018598 | 0.193 | 0.002 | 0.002 | 0.002 | 0.801 |
| Recio de Almería | Landrace | Spain | BGE013610 | 0.069 | 0.015 | 0.118 | 0.001 | 0.798 |
| Haurani 79-b | Landrace | Syria |  | 0.587 | 0.001 | 0.001 | 0.003 | 0.409 |
| IG-95841 | Landrace | Syria |  | 0.501 | 0.001 | 0.051 | 0.363 | 0.085 |
| IG-95931 | Landrace | Syria |  | 0.431 | 0.001 | 0.060 | 0.499 | 0.009 |
| IG-95812 | Landrace | Syria |  | 0.003 | 0.001 | 0.002 | 0.993 | 0.002 |
| IG-95847 | Landrace | Syria |  | 0.000 | 0.000 | 0.000 | 0.999 | 0.000 |
| Louri AP 5 | Landrace | Tunisia | Citr-3225 | 0.725 | 0.001 | 0.108 | 0.004 | 0.161 |
| Jennah Khetifa Rp4 | Landrace | Tunisia |  | 0.697 | 0.001 | 0.083 | 0.001 | 0.217 |
| Souri | Landrace | Tunisia | Citr-6870 | 0.650 | 0.001 | 0.013 | 0.085 | 0.250 |
| Hamira | Landrace | Tunisia | Citr-6874 | 0.510 | 0.002 | 0.003 | 0.074 | 0.411 |
| Realforte | Landrace | Tunisia | Citr-6871 | 0.268 | 0.030 | 0.007 | 0.063 | 0.632 |
| Biskri | Landrace | Tunisia | Citr-6872 | 0.005 | 0.001 | 0.000 | 0.000 | 0.994 |
| BGE-019263 | Landrace | Turkey |  | 0.803 | 0.001 | 0.051 | 0.143 | 0.002 |
| BGE019266 | Landrace | Turkey |  | 0.791 | 0.001 | 0.007 | 0.199 | 0.001 |
| BGE-018354 | Landrace | Turkey |  | 0.766 | 0.021 | 0.034 | 0.115 | 0.064 |
| BGE019265 | Landrace | Turkey |  | 0.760 | 0.001 | 0.002 | 0.237 | 0.000 |
| BGE019264 | Landrace | Turkey |  | 0.744 | 0.001 | 0.020 | 0.234 | 0.000 |
| BGE019262 | Landrace | Turkey |  | 0.742 | 0.001 | 0.009 | 0.249 | 0.000 |
| BGE-018192 | Landrace | Turkey |  | 0.739 | 0.001 | 0.024 | 0.236 | 0.001 |
| BGE018353 | Landrace | Turkey |  | 0.671 | 0.001 | 0.013 | 0.314 | 0.001 |
| BGE018351 | Landrace | Turkey |  | 0.595 | 0.001 | 0.001 | 0.004 | 0.400 |
| Mindium | Landrace | Turkey | BGE012407 | 0.583 | 0.002 | 0.210 | 0.202 | 0.003 |
| BGE-019270 | Landrace | Turkey |  | 0.550 | 0.070 | 0.095 | 0.277 | 0.008 |
| Sahel 77 | Modern | Algeria |  | 0.140 | 0.599 | 0.259 | 0.000 | 0.002 |
| Cham-1 | Modern | Algeria |  | 0.110 | 0.477 | 0.308 | 0.043 | 0.062 |
| Waha | Modern | Algeria |  | 0.077 | 0.499 | 0.300 | 0.018 | 0.106 |
| Buck Candisur | Modern | Argentina |  | 0.051 | 0.528 | 0.183 | 0.001 | 0.236 |
| Bonaerense Valverde | Modern | Argentina |  | 0.047 | 0.936 | 0.014 | 0.002 | 0.001 |
| Buck Cristal | Modern | Argentina |  | 0.001 | 0.984 | 0.010 | 0.001 | 0.003 |
| Bellaroi | Modern | Australia |  | 0.206 | 0.475 | 0.307 | 0.009 | 0.003 |
| Arivato | Modern | Australia |  | 0.113 | 0.421 | 0.382 | 0.000 | 0.083 |
| Kalka | Modern | Australia |  | 0.104 | 0.398 | 0.332 | 0.162 | 0.004 |
| Saintly | Modern | Australia |  | 0.068 | 0.385 | 0.358 | 0.176 | 0.013 |
| Tamaroi | Modern | Australia |  | 0.005 | 0.587 | 0.339 | 0.017 | 0.053 |
| AC Avonlea | Modern | Canada |  | 0.127 | 0.021 | 0.843 | 0.001 | 0.008 |
| Wakooma | Modern | Canada |  | 0.104 | 0.001 | 0.881 | 0.014 | 0.000 |
| Waskana | Modern | Canada |  | 0.050 | 0.001 | 0.929 | 0.019 | 0.001 |
| Strongfield | Modern | Canada |  | 0.027 | 0.311 | 0.641 | 0.009 | 0.012 |
| AC Pathfinder | Modern | Canada |  | 0.022 | 0.001 | 0.951 | 0.001 | 0.025 |
| AC Navigator | Modern | Canada |  | 0.015 | 0.001 | 0.978 | 0.002 | 0.004 |
| Commander | Modern | Canada |  | 0.005 | 0.001 | 0.981 | 0.001 | 0.012 |
| AC Morse | Modern | Canada |  | 0.001 | 0.001 | 0.993 | 0.005 | 0.000 |
| Macoun | Modern | Canada |  | 0.000 | 0.000 | 0.000 | 0.000 | 0.999 |
| Ucaro 1 | Modern | Chile |  | 0.022 | 0.754 | 0.221 | 0.002 | 0.001 |
| Chonta INIA | Modern | Chile |  | 0.020 | 0.755 | 0.220 | 0.004 | 0.002 |
| Guayacan INIA | Modern | Chile |  | 0.018 | 0.725 | 0.213 | 0.001 | 0.043 |
| Quilafen | Modern | Chile |  | 0.003 | 0.691 | 0.086 | 0.016 | 0.205 |
| Chagual INIA | Modern | Chile |  | 0.000 | 0.999 | 0.000 | 0.000 | 0.000 |
| Sula | Modern | Mexico |  | 0.001 | 0.998 | 0.000 | 0.000 | 0.000 |
| Porto 5 | Modern | Mexico |  | 0.001 | 0.997 | 0.000 | 0.000 | 0.002 |
| Somat | Modern | Mexico |  | 0.001 | 0.844 | 0.014 | 0.001 | 0.140 |
| Plata 16 | Modern | Mexico |  | 0.000 | 0.999 | 0.000 | 0.000 | 0.000 |
| Vitron | Modern | Mexico |  | 0.000 | 0.998 | 0.001 | 0.001 | 0.000 |
| Mesaoria | Modern | Cyprus |  | 0.188 | 0.602 | 0.184 | 0.001 | 0.025 |
| Aronas | Modern | Cyprus |  | 0.095 | 0.447 | 0.441 | 0.012 | 0.005 |
| Arendeto | Modern | Ethiopia |  | 0.185 | 0.618 | 0.181 | 0.001 | 0.015 |
| Boohai | Modern | Ethiopia |  | 0.141 | 0.203 | 0.152 | 0.002 | 0.502 |
| Hora | Modern | Ethiopia |  | 0.012 | 0.808 | 0.166 | 0.005 | 0.010 |
| Marou | Modern | Ethiopia |  | 0.002 | 0.920 | 0.001 | 0.001 | 0.075 |
| Flodur | Modern | France |  | 0.239 | 0.005 | 0.606 | 0.008 | 0.143 |
| Ardente | Modern | France |  | 0.214 | 0.188 | 0.159 | 0.063 | 0.377 |
| Arcodur | Modern | France |  | 0.147 | 0.016 | 0.721 | 0.009 | 0.107 |
| Ambral | Modern | France |  | 0.091 | 0.106 | 0.676 | 0.006 | 0.121 |
| Artimon | Modern | France |  | 0.032 | 0.841 | 0.045 | 0.002 | 0.080 |
| Attila | Modern | France |  | 0.014 | 0.590 | 0.126 | 0.010 | 0.260 |
| Arment | Modern | France |  | 0.012 | 0.115 | 0.276 | 0.130 | 0.467 |
| Imhotep | Modern | France |  | 0.011 | 0.335 | 0.497 | 0.007 | 0.150 |
| Epidur | Modern | France |  | 0.001 | 0.397 | 0.512 | 0.000 | 0.090 |
| Arcalis | Modern | France |  | 0.001 | 0.316 | 0.469 | 0.001 | 0.214 |
| Aronde | Modern | France |  | 0.001 | 0.079 | 0.231 | 0.078 | 0.610 |
| Auroc | Modern | France |  | 0.000 | 0.004 | 0.949 | 0.000 | 0.046 |
| Burgos | Modern | Germany |  | 0.108 | 0.598 | 0.287 | 0.002 | 0.005 |
| Bonitec | Modern | Germany |  | 0.000 | 0.999 | 0.000 | 0.000 | 0.000 |
| Raj 1555 | Modern | India |  | 0.121 | 0.728 | 0.148 | 0.002 | 0.002 |
| Malvaraj | Modern | India |  | 0.039 | 0.646 | 0.000 | 0.003 | 0.312 |
| WH 896 | Modern | India |  | 0.006 | 0.913 | 0.075 | 0.004 | 0.001 |
| Malavika | Modern | India |  | 0.003 | 0.776 | 0.221 | 0.001 | 0.001 |
| Narbada 215 | Modern | India |  | 0.003 | 0.755 | 0.240 | 0.000 | 0.001 |
| Oscar | Modern | Iran |  | 0.005 | 0.151 | 0.451 | 0.002 | 0.392 |
| Hazera | Modern | Israel |  | 0.067 | 0.795 | 0.137 | 0.001 | 0.000 |
| Creso | Modern | Italy |  | 0.780 | 0.003 | 0.002 | 0.001 | 0.214 |
| Claudio | Modern | Italy |  | 0.105 | 0.616 | 0.199 | 0.064 | 0.015 |
| Flavio | Modern | Italy |  | 0.099 | 0.189 | 0.243 | 0.005 | 0.464 |
| Svevo | Modern | Italy |  | 0.078 | 0.789 | 0.131 | 0.000 | 0.001 |
| Anento | Modern | Italy |  | 0.012 | 0.161 | 0.161 | 0.027 | 0.640 |
| Grecale | Modern | Italy |  | 0.002 | 0.436 | 0.471 | 0.004 | 0.087 |
| Meridiano | Modern | Italy |  | 0.001 | 0.817 | 0.111 | 0.001 | 0.070 |
| Fenice | Modern | Italy |  | 0.001 | 0.751 | 0.146 | 0.001 | 0.101 |
| Adamello | Modern | Italy |  | 0.001 | 0.295 | 0.450 | 0.000 | 0.254 |
| Cirillo | Modern | Italy |  | 0.001 | 0.132 | 0.665 | 0.001 | 0.202 |
| Simeto | Modern | Italy |  | 0.001 | 0.058 | 0.150 | 0.167 | 0.624 |
| Fortore | Modern | Italy |  | 0.001 | 0.011 | 0.232 | 0.324 | 0.433 |
| Casiello | Modern | Italy |  | 0.001 | 0.004 | 0.131 | 0.298 | 0.566 |
| Appulo | Modern | Italy |  | 0.001 | 0.000 | 0.000 | 0.482 | 0.517 |
| Sarif | Modern | Morocco |  | 0.180 | 0.683 | 0.027 | 0.005 | 0.106 |
| 1804 | Modern | Morocco |  | 0.116 | 0.622 | 0.103 | 0.004 | 0.156 |
| 1807 | Modern | Morocco |  | 0.108 | 0.680 | 0.087 | 0.003 | 0.122 |
| Tassaout | Modern | Morocco |  | 0.065 | 0.685 | 0.082 | 0.001 | 0.167 |
| 1808 | Modern | Morocco |  | 0.058 | 0.665 | 0.170 | 0.012 | 0.095 |
| Yasmine | Modern | Morocco |  | 0.044 | 0.697 | 0.095 | 0.002 | 0.161 |
| 1805 | Modern | Morocco |  | 0.040 | 0.849 | 0.002 | 0.108 | 0.002 |
| 1809 | Modern | Morocco |  | 0.037 | 0.898 | 0.001 | 0.063 | 0.001 |
| Massa | Modern | Morocco |  | 0.001 | 0.950 | 0.048 | 0.001 | 0.000 |
| Annouar | Modern | Morocco |  | 0.000 | 0.999 | 0.000 | 0.000 | 0.000 |
| Karim | Modern | Morocco |  | 0.000 | 0.999 | 0.000 | 0.000 | 0.000 |
| Ouedezena | Modern | Morocco |  | 0.000 | 0.999 | 0.000 | 0.000 | 0.000 |
| Enduro | Modern | Netherland |  | 0.046 | 0.550 | 0.215 | 0.001 | 0.189 |
| Wadhanak 85 | Modern | Pakistan |  | 0.004 | 0.904 | 0.002 | 0.013 | 0.077 |
| Alcamin | Modern | Portugal |  | 0.001 | 0.124 | 0.221 | 0.336 | 0.318 |
| Bakht | Modern | Russia |  | 0.075 | 0.383 | 0.424 | 0.116 | 0.001 |
| Selinogradskaja | Modern | Russia |  | 0.014 | 0.110 | 0.373 | 0.047 | 0.456 |
| Anibal | Modern | Spain |  | 0.502 | 0.180 | 0.172 | 0.010 | 0.136 |
| Camacho | Modern | Spain |  | 0.296 | 0.144 | 0.001 | 0.001 | 0.558 |
| Jaguar | Modern | Spain |  | 0.287 | 0.543 | 0.083 | 0.002 | 0.084 |
| Jiloca | Modern | Spain |  | 0.283 | 0.130 | 0.024 | 0.009 | 0.554 |
| Debano | Modern | Spain |  | 0.164 | 0.267 | 0.352 | 0.001 | 0.214 |
| Pingüino | Modern | Spain |  | 0.160 | 0.538 | 0.027 | 0.001 | 0.275 |
| Valira | Modern | Spain |  | 0.152 | 0.728 | 0.001 | 0.004 | 0.115 |
| Randur | Modern | Spain |  | 0.139 | 0.072 | 0.521 | 0.065 | 0.203 |
| Excalibur | Modern | Spain |  | 0.128 | 0.022 | 0.508 | 0.055 | 0.287 |
| Tejón | Modern | Spain |  | 0.127 | 0.531 | 0.334 | 0.003 | 0.005 |
| Euroduro | Modern | Spain |  | 0.125 | 0.871 | 0.002 | 0.001 | 0.001 |
| Belladur | Modern | Spain |  | 0.103 | 0.598 | 0.285 | 0.002 | 0.012 |
| Ponferrada | Modern | Spain |  | 0.098 | 0.634 | 0.176 | 0.069 | 0.023 |
| Ariesol | Modern | Spain |  | 0.093 | 0.323 | 0.580 | 0.001 | 0.004 |
| Jabato | Modern | Spain |  | 0.086 | 0.487 | 0.298 | 0.039 | 0.090 |
| Kidur | Modern | Spain |  | 0.085 | 0.021 | 0.744 | 0.006 | 0.144 |
| Mexidur | Modern | Spain |  | 0.081 | 0.166 | 0.425 | 0.006 | 0.321 |
| Boabdil | Modern | Spain |  | 0.080 | 0.726 | 0.163 | 0.002 | 0.028 |
| Donduro | Modern | Spain |  | 0.078 | 0.809 | 0.111 | 0.001 | 0.001 |
| Bonzo | Modern | Spain |  | 0.076 | 0.404 | 0.346 | 0.001 | 0.174 |
| Semolero | Modern | Spain |  | 0.073 | 0.784 | 0.099 | 0.001 | 0.044 |
| Asdrúbal | Modern | Spain |  | 0.065 | 0.003 | 0.783 | 0.057 | 0.093 |
| Bolido | Modern | Spain |  | 0.051 | 0.623 | 0.175 | 0.001 | 0.150 |
| Mellaria | Modern | Spain |  | 0.045 | 0.498 | 0.454 | 0.003 | 0.001 |
| Santadur | Modern | Spain |  | 0.037 | 0.783 | 0.171 | 0.001 | 0.007 |
| Safari | Modern | Spain |  | 0.037 | 0.136 | 0.678 | 0.148 | 0.001 |
| Severo | Modern | Spain |  | 0.030 | 0.922 | 0.003 | 0.020 | 0.025 |
| Esquilache | Modern | Spain |  | 0.024 | 0.291 | 0.456 | 0.055 | 0.173 |
| Prospero | Modern | Spain |  | 0.022 | 0.738 | 0.219 | 0.013 | 0.008 |
| Ramirez | Modern | Spain |  | 0.018 | 0.781 | 0.139 | 0.011 | 0.051 |
| Taranto | Modern | Spain |  | 0.015 | 0.960 | 0.023 | 0.001 | 0.000 |
| Bolo | Modern | Spain |  | 0.006 | 0.702 | 0.060 | 0.037 | 0.196 |
| Hispasano | Modern | Spain |  | 0.005 | 0.892 | 0.005 | 0.001 | 0.097 |
| Astigi | Modern | Spain |  | 0.005 | 0.789 | 0.198 | 0.001 | 0.007 |
| Carpio | Modern | Spain |  | 0.004 | 0.544 | 0.342 | 0.001 | 0.110 |
| Borgia | Modern | Spain |  | 0.004 | 0.403 | 0.008 | 0.050 | 0.535 |
| Valgera | Modern | Spain |  | 0.004 | 0.125 | 0.380 | 0.034 | 0.457 |
| Jupare | Modern | Spain |  | 0.003 | 0.917 | 0.001 | 0.002 | 0.077 |
| Boreal | Modern | Spain |  | 0.003 | 0.699 | 0.059 | 0.040 | 0.199 |
| Duradero | Modern | Spain |  | 0.002 | 0.991 | 0.003 | 0.001 | 0.002 |
| Gallareta | Modern | Spain |  | 0.002 | 0.973 | 0.001 | 0.010 | 0.013 |
| Vitronero | Modern | Spain |  | 0.002 | 0.537 | 0.456 | 0.002 | 0.003 |
| Mexa | Modern | Spain |  | 0.002 | 0.284 | 0.534 | 0.002 | 0.178 |
| Amilcar | Modern | Spain |  | 0.001 | 0.997 | 0.001 | 0.001 | 0.001 |
| Ancalei | Modern | Spain |  | 0.001 | 0.951 | 0.010 | 0.038 | 0.001 |
| Senadur | Modern | Spain |  | 0.001 | 0.383 | 0.584 | 0.001 | 0.031 |
| Tetradur | Modern | Spain |  | 0.001 | 0.171 | 0.793 | 0.012 | 0.024 |
| Bidi 17 | Modern | Spain |  | 0.000 | 0.000 | 0.000 | 0.000 | 0.999 |
| Omrabi 5 | Modern | Syria |  | 0.245 | 0.168 | 0.184 | 0.337 | 0.066 |
| Stojocri-2 | Modern | Syria |  | 0.171 | 0.567 | 0.126 | 0.006 | 0.129 |
| Stojocri-3 | Modern | Syria |  | 0.160 | 0.571 | 0.126 | 0.002 | 0.141 |
| Sebah | Modern | Syria |  | 0.156 | 0.738 | 0.055 | 0.003 | 0.048 |
| ORT-1 | Modern | Syria |  | 0.125 | 0.695 | 0.091 | 0.084 | 0.005 |
| Chanst | Modern | Syria |  | 0.071 | 0.855 | 0.001 | 0.001 | 0.072 |
| Khabur-1 | Modern | Syria |  | 0.058 | 0.893 | 0.001 | 0.001 | 0.048 |
| Awali-1 | Modern | Syria |  | 0.054 | 0.718 | 0.052 | 0.001 | 0.176 |
| Guerou-1 | Modern | Syria |  | 0.044 | 0.950 | 0.003 | 0.000 | 0.002 |
| Marsyr-1 | Modern | Syria |  | 0.041 | 0.841 | 0.005 | 0.058 | 0.055 |
| Aghrass-1 | Modern | Syria |  | 0.041 | 0.701 | 0.013 | 0.077 | 0.169 |
| Moulsabil 2 | Modern | Syria |  | 0.032 | 0.850 | 0.003 | 0.001 | 0.113 |
| Fardes | Modern | Syria |  | 0.028 | 0.888 | 0.034 | 0.003 | 0.047 |
| Derra | Modern | Syria |  | 0.020 | 0.615 | 0.316 | 0.021 | 0.028 |
| Chahba 88 | Modern | Syria |  | 0.007 | 0.931 | 0.036 | 0.024 | 0.002 |
| Quabrach-1 | Modern | Syria |  | 0.007 | 0.828 | 0.001 | 0.006 | 0.157 |
| Arislahn-5 | Modern | Syria |  | 0.007 | 0.768 | 0.113 | 0.002 | 0.110 |
| Chacan | Modern | Syria |  | 0.006 | 0.793 | 0.088 | 0.016 | 0.097 |
| Zeina 1 | Modern | Syria |  | 0.004 | 0.678 | 0.081 | 0.020 | 0.218 |
| Moosabil-1 | Modern | Syria |  | 0.004 | 0.352 | 0.429 | 0.087 | 0.128 |
| Stork | Modern | Syria |  | 0.003 | 0.270 | 0.552 | 0.002 | 0.173 |
| Lahn | Modern | Syria |  | 0.002 | 0.991 | 0.000 | 0.004 | 0.002 |
| Loukos-1 | Modern | Syria |  | 0.002 | 0.957 | 0.038 | 0.002 | 0.000 |
| Wadalmez-1 | Modern | Syria |  | 0.002 | 0.774 | 0.097 | 0.051 | 0.076 |
| Ouaserl-1 | Modern | Syria |  | 0.002 | 0.679 | 0.101 | 0.073 | 0.146 |
| Sabil 1 | Modern | Syria |  | 0.001 | 0.998 | 0.000 | 0.000 | 0.000 |
| Omruf-2 | Modern | Syria |  | 0.001 | 0.958 | 0.000 | 0.000 | 0.040 |
| Ammar-1 | Modern | Syria |  | 0.001 | 0.936 | 0.001 | 0.020 | 0.042 |
| Ouasloukos-1 | Modern | Syria |  | 0.001 | 0.916 | 0.075 | 0.002 | 0.006 |
| Brachoua | Modern | Syria |  | 0.001 | 0.897 | 0.005 | 0.000 | 0.098 |
| Maamouri-1 | Modern | Syria |  | 0.001 | 0.849 | 0.048 | 0.017 | 0.084 |
| Terbol97-3 | Modern | Syria |  | 0.001 | 0.779 | 0.015 | 0.111 | 0.095 |
| Murlagost-1 | Modern | Syria |  | 0.001 | 0.778 | 0.135 | 0.001 | 0.086 |
| Lagost 3 | Modern | Syria |  | 0.001 | 0.739 | 0.116 | 0.005 | 0.138 |
| Lagonil-2 | Modern | Syria |  | 0.001 | 0.718 | 0.093 | 0.069 | 0.118 |
| Omgenil-3 | Modern | Syria |  | 0.001 | 0.712 | 0.001 | 0.185 | 0.101 |
| Kabir1 | Modern | Syria |  | 0.001 | 0.673 | 0.026 | 0.073 | 0.226 |
| Massara-1 | Modern | Syria |  | 0.001 | 0.464 | 0.068 | 0.284 | 0.183 |
| Omrabi 3 | Modern | Syria |  | 0.001 | 0.447 | 0.056 | 0.282 | 0.214 |
| Awalbit-7 | Modern | Syria |  | 0.001 | 0.331 | 0.075 | 0.332 | 0.262 |
| Bicre | Modern | Syria |  | 0.000 | 0.985 | 0.000 | 0.000 | 0.014 |
| Mâali | Modern | Tunisia |  | 0.097 | 0.858 | 0.007 | 0.001 | 0.037 |
| Nasr 99 | Modern | Tunisia |  | 0.002 | 0.694 | 0.088 | 0.003 | 0.213 |
| Khiar 92 | Modern | Tunisia |  | 0.001 | 0.997 | 0.001 | 0.000 | 0.001 |
| Razzak 87 | Modern | Tunisia |  | 0.001 | 0.844 | 0.155 | 0.001 | 0.000 |
| Karim 80 | Modern | Tunisia |  | 0.000 | 0.999 | 0.001 | 0.000 | 0.000 |
| Amanos 97.3.1 | Modern | Turkey |  | 0.009 | 0.691 | 0.280 | 0.017 | 0.003 |
| Modoc | Modern | USA |  | 0.308 | 0.086 | 0.458 | 0.011 | 0.137 |
| Lakota | Modern | USA |  | 0.247 | 0.001 | 0.615 | 0.136 | 0.000 |
| Duraking | Modern | USA |  | 0.078 | 0.679 | 0.242 | 0.001 | 0.001 |
| Cortez | Modern | USA |  | 0.064 | 0.437 | 0.323 | 0.040 | 0.135 |
| Orita | Modern | USA |  | 0.002 | 0.328 | 0.587 | 0.001 | 0.082 |
| West Bred Laker | Modern | USA |  | 0.002 | 0.235 | 0.581 | 0.003 | 0.179 |
| West Bred Turbo | Modern | USA |  | 0.001 | 0.681 | 0.205 | 0.109 | 0.004 |
| Kronos | Modern | USA |  | 0.001 | 0.374 | 0.533 | 0.000 | 0.091 |
| Lloyd | Modern | USA |  | 0.001 | 0.112 | 0.884 | 0.000 | 0.003 |
| Durex | Modern | USA |  | 0.001 | 0.101 | 0.772 | 0.001 | 0.126 |
| Ocotillo | Modern | USA |  | 0.001 | 0.031 | 0.872 | 0.001 | 0.094 |
| Vic | Modern | USA |  | 0.001 | 0.003 | 0.941 | 0.001 | 0.055 |
| Monroe | Modern | USA |  | 0.001 | 0.001 | 0.997 | 0.001 | 0.000 |
| Colorado | Modern | USA |  | 0.001 | 0.001 | 0.862 | 0.000 | 0.136 |
| Medora | Modern | USA |  | 0.001 | 0.000 | 0.998 | 0.001 | 0.000 |
| Ward | Modern | USA |  | 0.001 | 0.000 | 0.998 | 0.001 | 0.000 |
| Fjord | Modern | USA |  | 0.000 | 0.002 | 0.996 | 0.001 | 0.000 |
| Matt | Modern | USA |  | 0.000 | 0.001 | 0.795 | 0.000 | 0.203 |

**Supplementary TABLE S2 ǀ** Mean values across years of the studied traits for each genotype.*NS, number of spikes m^-2^; NGS, number of grains spike^-1^; W, grain weight; HI, harvest index; DEH, days from emergence to heading; DHA, days from heading to anthesis; DAM, days from anthesis to maturity; GFR, grain filling rate; PL, peduncle length; PH, plant height; DM_A_, dry matter at anthesis; DM_M_, dry matter at maturity; CTD_A_, canopy temperature depression at anthesis; CTD_Mi_, canopy temperature depression at milky-dough grain stage; cPAR_EH_, absorbed radiation from emergence to heading; cPAR_HA_, absorbed radiation from heading to anthesis; cPAR_AM_, absorbed radiation from anthesis to maturity; RUE_EA_, radiation use efficiency from emergence to anthesis; RUE_AM_, radiation use efficiency from anthesis to maturity; GA_90d_, green area accumulated at 90 days from emergence.*

| **Cultivar** | **Yield** (kg ha^-1^) | **NS** | **NGS** | **W**  (mg grain^-1^) | **HI** | **DEH** | **DHA** | **DAM** | **GFR**  (mg day^-1^) | **PL** (cm) | **PH**  (cm) | **DM_A_**  (g m^-2^) | **DM_M_** (g m^-2^) | **CTDA** (°C) | **CTDMi** (°C) | **cPAR_EH_** (MJ m^-2^) | **cPAR_HA_**  (MJ m^-2^) | **cPAR_AM_**  (MJ m^-2^) | **RUE_EA_**  (g MJ^-1^) | **RUE_AM_** (g MJ^-1^) | **GA_90d_** |
| --- | --- | --- | --- | --- | --- | --- | --- | --- | --- | --- | --- | --- | --- | --- | --- | --- | --- | --- | --- | --- | --- |
| 1575 | 4104 | 327 | 29.4 | 49.1 | 0.39 | 132.7 | 5.1 | 30.2 | 1.69 | 57.0 | 125.2 | 759 | 1202 | -0.92 | -1.26 | 263 | 38.3 | 166.4 | 2.5 | 2.9 | 135.3 |
| 1640 | 3875 | 345 | 34.1 | 47.9 | 0.36 | 129.2 | 4.3 | 32.3 | 1.49 | 48.9 | 118.0 | 672 | 1555 | 2.15 | -0.71 | 232 | 30.4 | 180.9 | 2.7 | 2.8 | 141.5 |
| 18/71 | 4445 | 350 | 35.2 | 45.4 | 0.44 | 121.9 | 7.0 | 33.4 | 1.37 | 48.2 | 103.5 | 765 | 1266 | 1.63 | 0.05 | 243 | 38.5 | 175.1 | 2.8 | 2.6 | 144.6 |
| 1804 | 4363 | 329 | 32.2 | 42.7 | 0.47 | 121.9 | 6.5 | 35.1 | 1.22 | 35.7 | 77.6 | 662 | 954 | 0.36 | -1.57 | 199 | 32.5 | 162.1 | 2.9 | 1.8 | 134.9 |
| 1805 | 4092 | 356 | 35.8 | 43.3 | 0.45 | 123.7 | 6.6 | 33.7 | 1.30 | 31.9 | 71.4 | 697 | 1223 | 0.86 | -0.08 | 223 | 36.6 | 168.4 | 2.7 | 3.5 | 127.0 |
| 1807 | 4068 | 341 | 33.5 | 42.7 | 0.47 | 122.6 | 7.7 | 32.1 | 1.35 | 36.9 | 74.0 | 655 | 1046 | 0.95 | -0.27 | 206 | 41.0 | 157.2 | 2.7 | 2.4 | 130.3 |
| 1808 | 4456 | 336 | 31.3 | 48.4 | 0.44 | 122.6 | 6.7 | 34.7 | 1.39 | 32.4 | 74.2 | 686 | 1128 | 1.36 | 0.28 | 248 | 39.5 | 185.4 | 2.4 | 2.5 | 126.6 |
| 1809 | 4293 | 313 | 31.5 | 45.8 | 0.45 | 124.0 | 7.8 | 32.5 | 1.41 | 29.7 | 72.5 | 668 | 974 | -0.76 | -0.48 | 192 | 40.1 | 154.3 | 2.9 | 2.3 | 119.2 |
| 196/71 | 4122 | 361 | 28.9 | 43.9 | 0.36 | 132.9 | 5.0 | 30.5 | 1.49 | 52.2 | 124.3 | 925 | 1255 | -0.15 | -1.60 | 273 | 39.2 | 158.0 | 3.0 | 2.2 | 146.5 |
| 1P1 | 3988 | 323 | 26.6 | 39.3 | 0.40 | 126.5 | 4.7 | 32.4 | 1.22 | 39.8 | 90.1 | 832 | 848 | 1.68 | -0.33 | 260 | 36.4 | 166.4 | 2.8 | 0.8 | 122.2 |
| 23 | 3901 | 313 | 27.0 | 47.1 | 0.35 | 131.8 | 6.5 | 27.9 | 1.73 | 47.6 | 126.5 | 781 | 1108 | -0.14 | -1.84 | 293 | 49.9 | 154.7 | 2.4 | 2.1 | 156.1 |
| 248-VII/7 | 3667 | 357 | 30.0 | 41.0 | 0.36 | 132.1 | 4.3 | 29.2 | 1.43 | 42.9 | 116.1 | 837 | 1205 | 0.90 | -0.05 | 294 | 36.5 | 157.2 | 2.6 | 2.4 | 144.1 |
| 259-VII/12 | 4164 | 361 | 27.5 | 46.7 | 0.38 | 134.7 | 4.1 | 30.5 | 1.56 | 53.5 | 121.9 | 783 | 1271 | -0.79 | -2.35 | 287 | 36.8 | 178.9 | 2.5 | 2.0 | 145.2 |
| 26 | 4079 | 356 | 32.0 | 45.0 | 0.41 | 126.9 | 5.2 | 32.7 | 1.38 | 45.6 | 94.0 | 678 | 1258 | 2.15 | -0.73 | 252 | 35.8 | 163.6 | 2.4 | 3.3 | 132.7 |
| 2751 | 3536 | 368 | 30.3 | 38.0 | 0.42 | 126.9 | 3.9 | 30.7 | 1.26 | 36.9 | 86.3 | 711 | 1018 | 1.74 | -0.41 | 259 | 33.2 | 170.7 | 2.5 | 1.7 | 134.9 |
| 28 | 3717 | 374 | 32.6 | 41.7 | 0.37 | 127.9 | 5.0 | 31.3 | 1.23 | 44.6 | 113.3 | 756 | 1274 | 3.66 | -0.65 | 273 | 36.0 | 169.2 | 2.5 | 2.4 | 131.4 |
| 31 | 3324 | 424 | 35.3 | 32.5 | 0.39 | 132.2 | 3.2 | 28.3 | 1.17 | 35.8 | 96.8 | 715 | 1236 | 4.11 | 0.55 | 239 | 23.0 | 158.3 | 2.7 | 3.3 | 131.2 |
| 33 | 3763 | 303 | 28.5 | 41.2 | 0.35 | 136.1 | 4.3 | 30.7 | 1.38 | 47.5 | 123.1 | 704 | 1002 | -1.07 | 0.51 | 308 | 35.4 | 168.5 | 2.1 | 1.6 | 137.3 |
| 356-I/9 | 3953 | 365 | 29.1 | 43.1 | 0.36 | 132.2 | 3.9 | 31.2 | 1.40 | 48.5 | 122.7 | 796 | 1268 | 0.89 | -0.97 | 297 | 28.5 | 167.3 | 2.5 | 2.5 | 143.2 |
| 37 | 3408 | 347 | 32.3 | 40.5 | 0.36 | 136.3 | 4.7 | 30.7 | 1.36 | 45.5 | 117.9 | 724 | 1239 | -0.85 | 1.05 | 319 | 38.2 | 169.5 | 2.0 | 3.0 | 137.3 |
| 42 | 3820 | 319 | 26.9 | 41.4 | 0.37 | 136.6 | 3.5 | 29.8 | 1.41 | 43.8 | 113.4 | 719 | 950 | -0.35 | -0.82 | 308 | 31.7 | 161.0 | 2.2 | 1.5 | 147.5 |
| 440-IX/96 | 4029 | 296 | 27.7 | 47.5 | 0.37 | 134.8 | 4.2 | 31.8 | 1.56 | 53.0 | 115.0 | 709 | 1005 | -0.05 | -0.95 | 287 | 33.6 | 166.7 | 2.4 | 1.8 | 146.9 |
| 441-IX/97 | 3970 | 345 | 26.7 | 45.3 | 0.38 | 135.7 | 4.5 | 28.5 | 1.62 | 44.1 | 114.0 | 692 | 1125 | -1.34 | -0.19 | 282 | 35.1 | 159.6 | 2.2 | 2.0 | 139.0 |
| 5P4 | 3417 | 321 | 33.9 | 38.2 | 0.37 | 131.9 | 3.8 | 31.6 | 1.21 | 39.0 | 107.8 | 720 | 1096 | -0.13 | -0.14 | 289 | 28.7 | 168.8 | 2.3 | 2.2 | 127.9 |
| 9918 | 3708 | 343 | 30.5 | 40.0 | 0.41 | 132.7 | 5.0 | 28.9 | 1.42 | 40.1 | 96.7 | 761 | 1005 | -2.23 | -1.09 | 247 | 33.0 | 151.5 | 2.7 | 1.5 | 119.8 |
| 9923 | 4083 | 398 | 29.3 | 45.1 | 0.42 | 124.7 | 6.6 | 33.6 | 1.36 | 46.1 | 96.2 | 607 | 1255 | 2.12 | -0.10 | 251 | 39.4 | 177.2 | 2.1 | 3.3 | 139.2 |
| 9929 | 3612 | 321 | 32.0 | 42.5 | 0.41 | 123.8 | 6.6 | 33.6 | 1.30 | 45.7 | 87.0 | 682 | 1045 | 1.01 | -1.19 | 252 | 39.2 | 181.9 | 2.4 | 2.2 | 133.9 |
| 9935 | 4122 | 354 | 29.7 | 41.8 | 0.39 | 125.0 | 5.9 | 33.6 | 1.26 | 43.5 | 93.1 | 726 | 1127 | 2.50 | 0.17 | 263 | 44.1 | 182.8 | 2.4 | 2.7 | 129.0 |
| Abu Fashit | 3211 | 396 | 29.4 | 35.2 | 0.44 | 121.8 | 5.2 | 32.5 | 1.10 | 34.1 | 74.0 | 654 | 944 | 0.14 | -1.20 | 175 | 22.9 | 153.2 | 3.4 | 1.8 | 122.3 |
| AC Avonlea | 4017 | 334 | 32.6 | 42.1 | 0.41 | 129.3 | 5.2 | 30.6 | 1.39 | 39.0 | 97.5 | 772 | 1118 | 3.30 | 0.22 | 246 | 33.3 | 167.8 | 2.7 | 1.9 | 129.4 |
| AC Morse | 4070 | 353 | 29.6 | 43.4 | 0.43 | 125.2 | 5.4 | 33.9 | 1.29 | 33.5 | 80.5 | 684 | 1064 | 2.52 | -0.64 | 233 | 35.5 | 168.9 | 2.6 | 2.1 | 135.9 |
| AC Navigator | 4529 | 344 | 30.8 | 42.7 | 0.44 | 125.4 | 4.0 | 34.2 | 1.26 | 35.8 | 76.4 | 733 | 1063 | 1.76 | -1.08 | 258 | 31.3 | 182.1 | 2.6 | 1.7 | 139.3 |
| AC Pathfinder | 4405 | 342 | 33.8 | 42.6 | 0.42 | 123.6 | 6.1 | 32.6 | 1.32 | 45.1 | 97.7 | 720 | 1177 | 1.73 | -0.33 | 230 | 38.9 | 165.5 | 2.6 | 2.8 | 140.0 |
| Adamello | 4612 | 311 | 24.7 | 52.4 | 0.44 | 121.9 | 6.4 | 35.6 | 1.48 | 29.3 | 73.3 | 744 | 895 | 0.23 | -0.63 | 208 | 35.4 | 184.0 | 3.1 | 0.9 | 132.3 |
| Aghrass-1 | 4853 | 325 | 34.0 | 43.9 | 0.47 | 125.0 | 6.0 | 33.3 | 1.32 | 38.2 | 81.2 | 720 | 1030 | 3.14 | -0.51 | 228 | 39.7 | 171.2 | 2.8 | 1.9 | 136.2 |
| Alcamin | 4071 | 326 | 26.0 | 42.5 | 0.41 | 125.9 | 5.3 | 34.1 | 1.27 | 39.8 | 95.0 | 678 | 1027 | 2.80 | 0.20 | 240 | 35.8 | 154.0 | 2.5 | 2.8 | 139.7 |
| Alentejo | 4019 | 307 | 33.5 | 46.7 | 0.38 | 134.9 | 4.5 | 30.7 | 1.57 | 48.8 | 114.8 | 781 | 1233 | -0.73 | -0.83 | 324 | 36.9 | 152.7 | 2.2 | 3.3 | 154.1 |
| Alonso | 4140 | 346 | 30.0 | 44.6 | 0.37 | 132.1 | 5.5 | 29.2 | 1.54 | 41.8 | 112.6 | 869 | 1283 | -1.00 | -1.55 | 282 | 41.0 | 161.2 | 2.7 | 2.2 | 142.5 |
| Amanos 97.3.1 | 4459 | 369 | 28.0 | 47.2 | 0.44 | 124.7 | 5.8 | 32.5 | 1.47 | 31.2 | 74.4 | 762 | 1085 | 1.50 | -0.88 | 241 | 37.2 | 172.7 | 2.8 | 1.7 | 145.9 |
| Amarelo Barba Preta | 4067 | 311 | 30.6 | 44.8 | 0.35 | 130.9 | 4.1 | 31.8 | 1.42 | 40.7 | 113.7 | 825 | 1206 | 2.87 | 1.57 | 314 | 29.5 | 171.6 | 2.5 | 2.5 | 150.2 |
| Ambral | 4639 | 331 | 34.2 | 41.9 | 0.45 | 127.3 | 5.9 | 34.1 | 1.24 | 32.1 | 72.7 | 719 | 1063 | 1.68 | -0.88 | 253 | 42.1 | 178.3 | 2.4 | 1.7 | 136.2 |
| Amilcar | 4342 | 321 | 37.6 | 45.3 | 0.50 | 122.5 | 6.8 | 33.6 | 1.34 | 33.9 | 69.5 | 731 | 1073 | 0.33 | -1.97 | 211 | 39.1 | 168.4 | 3.0 | 2.1 | 137.6 |
| Ammar-1 | 4709 | 328 | 32.0 | 45.9 | 0.47 | 123.4 | 6.6 | 34.0 | 1.35 | 34.9 | 76.0 | 702 | 1020 | -0.75 | -1.99 | 211 | 39.9 | 152.5 | 3.0 | 1.9 | 135.0 |
| Anafil | 3722 | 326 | 40.7 | 38.3 | 0.37 | 135.4 | 3.8 | 29.3 | 1.34 | 55.4 | 132.8 | 741 | 1371 | -1.23 | 1.56 | 290 | 29.1 | 156.7 | 2.4 | 4.7 | 133.8 |
| Ancalei | 4219 | 339 | 35.3 | 41.5 | 0.44 | 123.0 | 6.8 | 33.2 | 1.23 | 30.0 | 71.5 | 703 | 1077 | 1.90 | 0.46 | 219 | 39.4 | 161.9 | 2.8 | 2.4 | 127.9 |
| Andalucía 344 | 4137 | 357 | 29.4 | 43.9 | 0.37 | 132.0 | 4.0 | 30.0 | 1.50 | 48.4 | 113.6 | 800 | 1249 | 1.23 | -1.01 | 282 | 32.4 | 137.1 | 2.6 | 3.2 | 144.9 |
| Anento | 4435 | 314 | 29.5 | 46.5 | 0.44 | 122.0 | 6.1 | 36.3 | 1.28 | 32.5 | 74.8 | 683 | 964 | 0.73 | -0.20 | 226 | 31.8 | 162.0 | 2.6 | 1.6 | 126.8 |
| Anibal | 4591 | 348 | 31.4 | 43.7 | 0.44 | 130.0 | 4.2 | 32.3 | 1.38 | 33.2 | 79.0 | 726 | 1089 | 2.54 | -0.38 | 270 | 30.2 | 167.1 | 2.5 | 2.0 | 133.6 |
| Annouar | 4566 | 400 | 27.1 | 46.6 | 0.45 | 122.7 | 6.3 | 34.1 | 1.36 | 32.2 | 71.7 | 727 | 1117 | 0.28 | -1.53 | 235 | 43.1 | 181.1 | 2.7 | 1.9 | 139.2 |
| Appulo | 4430 | 378 | 32.5 | 43.2 | 0.45 | 121.1 | 5.6 | 36.2 | 1.20 | 39.1 | 81.0 | 698 | 1179 | -1.01 | -2.14 | 208 | 27.0 | 170.8 | 3.0 | 2.6 | 136.6 |
| Arcalis | 4546 | 347 | 30.5 | 49.1 | 0.45 | 129.3 | 4.6 | 35.3 | 1.40 | 30.7 | 73.7 | 736 | 1146 | 4.06 | -1.44 | 264 | 35.9 | 188.8 | 2.5 | 2.3 | 140.7 |
| Arcodur | 4455 | 360 | 29.5 | 41.3 | 0.44 | 133.1 | 3.4 | 32.5 | 1.29 | 30.2 | 71.2 | 775 | 1036 | -0.20 | -1.07 | 269 | 29.9 | 181.6 | 2.6 | 1.5 | 129.4 |
| Ardente | 4363 | 321 | 33.4 | 47.4 | 0.47 | 126.0 | 5.6 | 33.3 | 1.44 | 36.2 | 72.9 | 694 | 1073 | 0.60 | -0.94 | 220 | 33.4 | 175.2 | 2.7 | 2.3 | 122.6 |
| Arendeto | 4418 | 339 | 34.5 | 43.6 | 0.46 | 123.8 | 6.9 | 33.5 | 1.31 | 36.8 | 75.4 | 743 | 1147 | 0.84 | -0.73 | 232 | 41.8 | 177.4 | 2.8 | 2.3 | 144.3 |
| Ariesol | 5111 | 349 | 31.0 | 45.6 | 0.47 | 121.0 | 7.5 | 35.6 | 1.28 | 31.4 | 76.1 | 692 | 1054 | 0.11 | -0.90 | 239 | 42.0 | 184.4 | 2.5 | 1.7 | 133.6 |
| Arislahn-5 | 4449 | 318 | 34.8 | 44.0 | 0.46 | 122.8 | 7.3 | 33.6 | 1.32 | 29.8 | 70.8 | 658 | 1051 | 0.06 | -1.61 | 202 | 39.2 | 165.1 | 2.7 | 2.2 | 130.1 |
| Arisnegro de Tenerife | 4207 | 356 | 29.1 | 48.7 | 0.39 | 130.0 | 4.4 | 31.6 | 1.57 | 43.1 | 112.0 | 792 | 1349 | 3.72 | 0.23 | 280 | 29.7 | 171.9 | 2.6 | 2.7 | 131.9 |
| Arivato | 4507 | 325 | 32.0 | 42.8 | 0.45 | 124.7 | 6.4 | 32.9 | 1.32 | 39.7 | 77.2 | 666 | 1035 | 1.52 | -1.56 | 211 | 37.9 | 163.7 | 2.6 | 2.1 | 136.6 |
| Arment | 5085 | 334 | 33.4 | 41.1 | 0.46 | 123.5 | 6.7 | 35.2 | 1.17 | 29.5 | 71.1 | 756 | 986 | -0.54 | 0.31 | 212 | 32.7 | 179.9 | 3.1 | 1.2 | 142.4 |
| Aronas | 4543 | 325 | 33.5 | 46.1 | 0.45 | 124.2 | 6.5 | 34.1 | 1.35 | 37.1 | 79.1 | 717 | 1128 | 1.70 | -0.90 | 239 | 42.2 | 175.4 | 2.5 | 2.3 | 128.0 |
| Aronde | 4638 | 313 | 39.1 | 41.0 | 0.47 | 124.2 | 6.0 | 35.9 | 1.14 | 27.4 | 71.9 | 783 | 1028 | 2.17 | -0.26 | 247 | 39.4 | 187.5 | 2.8 | 1.4 | 130.6 |
| Artimon | 4880 | 343 | 36.3 | 44.5 | 0.47 | 121.1 | 6.3 | 34.5 | 1.28 | 33.9 | 70.3 | 704 | 1140 | -0.71 | -1.66 | 233 | 33.5 | 175.3 | 2.6 | 2.8 | 137.3 |
| Asdrúbal | 4092 | 347 | 39.2 | 35.1 | 0.47 | 127.1 | 5.4 | 32.1 | 1.10 | 28.3 | 66.7 | 657 | 1010 | 2.62 | -0.51 | 221 | 34.2 | 165.3 | 2.6 | 1.9 | 116.4 |
| Astigi | 4884 | 316 | 40.0 | 37.0 | 0.45 | 124.3 | 7.4 | 34.3 | 1.10 | 37.4 | 80.8 | 709 | 1026 | 2.51 | -0.08 | 239 | 50.9 | 177.3 | 2.5 | 2.0 | 126.3 |
| Attila | 4849 | 299 | 34.0 | 53.3 | 0.46 | 122.5 | 6.0 | 35.1 | 1.52 | 34.9 | 81.3 | 722 | 1095 | 0.88 | -0.99 | 234 | 34.3 | 188.3 | 2.7 | 2.2 | 136.6 |
| Auroc | 4305 | 364 | 31.0 | 43.4 | 0.46 | 130.8 | 4.3 | 31.6 | 1.37 | 37.3 | 75.7 | 708 | 1064 | 1.16 | -0.50 | 230 | 30.6 | 157.5 | 2.7 | 2.1 | 135.0 |
| Awalbit-7 | 4239 | 338 | 32.6 | 42.7 | 0.44 | 124.1 | 6.4 | 35.6 | 1.22 | 38.1 | 79.0 | 703 | 1073 | 1.44 | -0.70 | 223 | 36.0 | 165.7 | 2.7 | 2.6 | 131.4 |
| Awali-1 | 4608 | 335 | 30.6 | 49.4 | 0.46 | 122.1 | 6.2 | 34.5 | 1.43 | 35.6 | 79.9 | 721 | 1090 | 0.96 | -0.42 | 243 | 34.9 | 181.0 | 2.6 | 2.1 | 151.6 |
| Aziziah 17/45 | 3558 | 349 | 29.9 | 40.4 | 0.40 | 122.9 | 5.8 | 34.9 | 1.16 | 43.9 | 87.2 | 710 | 1027 | 2.34 | 0.04 | 244 | 33.9 | 175.3 | 2.7 | 2.0 | 132.2 |
| Azulejo de Villa del Río | 4103 | 324 | 31.2 | 44.9 | 0.38 | 127.0 | 5.4 | 31.9 | 1.42 | 48.9 | 111.9 | 806 | 1157 | 3.01 | 1.25 | 282 | 34.3 | 174.5 | 2.6 | 2.2 | 139.9 |
| Bakht | 4575 | 334 | 29.9 | 45.3 | 0.44 | 127.5 | 5.1 | 35.1 | 1.30 | 34.2 | 76.3 | 739 | 1037 | 3.29 | -0.23 | 259 | 33.7 | 175.6 | 2.6 | 1.4 | 149.6 |
| Balilla Falso | 4094 | 353 | 26.3 | 44.6 | 0.42 | 123.1 | 6.3 | 33.5 | 1.35 | 44.8 | 103.6 | 715 | 965 | 1.33 | -0.04 | 232 | 35.8 | 156.1 | 2.7 | 1.5 | 135.8 |
| Basto Duro | 4374 | 304 | 30.7 | 48.2 | 0.40 | 133.1 | 3.8 | 31.2 | 1.58 | 43.8 | 112.4 | 796 | 1119 | 1.21 | 0.74 | 304 | 28.4 | 153.7 | 2.4 | 2.1 | 143.2 |
| Beladi Rouge | 3586 | 365 | 29.0 | 42.5 | 0.36 | 128.6 | 4.2 | 29.5 | 1.66 | 44.3 | 109.7 | 739 | 1228 | 2.25 | -1.05 | 291 | 27.6 | 153.6 | 2.3 | 4.0 | 146.3 |
| Belgrade 9 | 3713 | 354 | 30.1 | 44.7 | 0.38 | 133.7 | 4.7 | 28.3 | 1.62 | 49.8 | 115.5 | 792 | 1269 | -0.50 | -0.15 | 287 | 37.0 | 153.0 | 2.5 | 2.6 | 138.2 |
| Belladur | 4769 | 350 | 28.1 | 45.2 | 0.44 | 125.9 | 6.3 | 31.4 | 1.47 | 32.4 | 74.7 | 730 | 1012 | 3.38 | -0.80 | 235 | 45.2 | 182.5 | 2.6 | 1.4 | 136.8 |
| Bellaroi | 4430 | 331 | 30.6 | 46.7 | 0.43 | 126.7 | 4.7 | 35.1 | 1.36 | 31.4 | 72.1 | 754 | 1092 | 1.61 | -0.62 | 248 | 32.2 | 177.1 | 2.7 | 1.9 | 135.2 |
| BGE-018192 | 4125 | 310 | 28.5 | 43.0 | 0.38 | 133.1 | 4.8 | 28.8 | 1.53 | 39.6 | 104.7 | 781 | 1003 | -0.55 | -1.59 | 271 | 36.2 | 158.8 | 2.6 | 2.0 | 138.9 |
| BGE018351 | 4257 | 321 | 31.5 | 50.8 | 0.35 | 132.9 | 3.6 | 31.2 | 1.63 | 49.3 | 119.0 | 827 | 1472 | 1.89 | -0.45 | 316 | 33.8 | 175.7 | 2.4 | 3.2 | 141.9 |
| BGE018353 | 3991 | 330 | 31.0 | 45.0 | 0.36 | 135.9 | 4.3 | 28.2 | 1.64 | 51.9 | 119.5 | 809 | 1292 | -2.36 | 0.40 | 284 | 36.1 | 149.8 | 2.5 | 3.4 | 141.7 |
| BGE-018354 | 3840 | 345 | 31.3 | 40.5 | 0.37 | 130.9 | 4.3 | 31.2 | 1.32 | 41.6 | 112.7 | 702 | 1181 | 2.35 | 0.83 | 256 | 28.7 | 161.6 | 2.6 | 2.5 | 137.5 |
| BGE019262 | 4398 | 334 | 32.9 | 46.9 | 0.40 | 132.6 | 5.0 | 31.3 | 1.53 | 45.0 | 109.5 | 766 | 1251 | -0.11 | -0.56 | 273 | 38.3 | 159.4 | 2.5 | 3.0 | 141.8 |
| BGE-019263 | 3508 | 371 | 34.0 | 42.5 | 0.37 | 137.5 | 3.4 | 30.1 | 1.44 | 46.1 | 106.6 | 748 | 1462 | -2.68 | 0.94 | 295 | 31.4 | 158.1 | 2.4 | 4.1 | 134.9 |
| BGE019264 | 3828 | 335 | 29.1 | 46.4 | 0.39 | 133.1 | 4.5 | 29.8 | 1.59 | 43.1 | 103.3 | 708 | 1153 | -0.13 | -0.53 | 264 | 36.5 | 164.0 | 2.4 | 2.8 | 131.5 |
| BGE019265 | 4090 | 344 | 36.1 | 44.1 | 0.40 | 133.3 | 5.5 | 29.1 | 1.55 | 43.6 | 107.1 | 832 | 1371 | -0.38 | -2.28 | 284 | 42.5 | 161.6 | 2.6 | 3.2 | 137.1 |
| BGE019266 | 4117 | 323 | 28.8 | 46.0 | 0.37 | 132.9 | 4.4 | 30.6 | 1.57 | 45.0 | 108.9 | 825 | 1129 | -0.27 | -1.67 | 292 | 33.3 | 168.5 | 2.5 | 1.8 | 139.3 |
| BGE-019270 | 4024 | 330 | 30.4 | 45.2 | 0.39 | 132.9 | 5.1 | 30.2 | 1.50 | 42.6 | 110.8 | 803 | 1146 | -0.99 | -1.68 | 276 | 37.6 | 161.9 | 2.6 | 2.3 | 137.6 |
| Bicre | 4622 | 340 | 33.7 | 48.3 | 0.48 | 124.6 | 6.2 | 34.3 | 1.40 | 31.6 | 72.0 | 699 | 1158 | 1.32 | -1.18 | 245 | 39.9 | 178.7 | 2.5 | 2.5 | 143.7 |
| Bidi 17 | 4318 | 350 | 33.0 | 47.8 | 0.39 | 130.0 | 4.2 | 32.6 | 1.49 | 42.8 | 101.2 | 855 | 1431 | 2.41 | 0.54 | 258 | 28.0 | 174.3 | 3.0 | 3.5 | 143.9 |
| Biskri | 4225 | 316 | 28.7 | 43.8 | 0.37 | 131.2 | 4.5 | 30.9 | 1.43 | 44.4 | 113.0 | 745 | 1043 | 0.80 | -0.72 | 279 | 31.6 | 158.4 | 2.5 | 1.7 | 146.6 |
| Blancal | 4138 | 328 | 30.7 | 51.2 | 0.38 | 130.4 | 3.9 | 33.0 | 1.58 | 49.5 | 114.9 | 756 | 1347 | 2.95 | 0.92 | 304 | 31.2 | 181.9 | 2.3 | 3.5 | 160.6 |
| Blanco de Corella | 4199 | 348 | 29.4 | 43.1 | 0.41 | 133.3 | 3.7 | 31.8 | 1.42 | 37.9 | 90.1 | 716 | 1054 | 1.26 | -0.03 | 304 | 26.8 | 171.1 | 2.2 | 1.7 | 153.4 |
| Blanco Verdeal | 4282 | 295 | 33.0 | 46.6 | 0.36 | 130.1 | 4.1 | 32.1 | 1.47 | 45.9 | 124.9 | 777 | 1240 | 3.96 | 0.39 | 289 | 31.8 | 187.5 | 2.4 | 2.3 | 144.9 |
| Blanquillo | 3969 | 331 | 31.9 | 40.1 | 0.39 | 133.5 | 4.6 | 31.5 | 1.31 | 43.5 | 119.6 | 768 | 1126 | -1.21 | -3.09 | 250 | 33.5 | 160.0 | 2.7 | 1.9 | 135.0 |
| Blanquillón de Boñar | 3622 | 319 | 34.4 | 41.9 | 0.35 | 133.5 | 4.8 | 32.0 | 1.33 | 57.8 | 134.5 | 820 | 1310 | -0.20 | -0.84 | 290 | 37.2 | 154.1 | 2.6 | 3.0 | 137.5 |
| Ble Dur 250 | 4155 | 308 | 28.1 | 45.3 | 0.38 | 132.9 | 3.9 | 30.9 | 1.51 | 51.0 | 121.1 | 754 | 1034 | -0.09 | -0.96 | 307 | 31.9 | 164.7 | 2.3 | 1.5 | 149.5 |
| Boabdil | 4991 | 335 | 31.4 | 48.3 | 0.45 | 123.6 | 7.1 | 32.7 | 1.47 | 33.4 | 72.9 | 762 | 1105 | 1.34 | -1.04 | 217 | 37.0 | 174.0 | 3.0 | 2.4 | 130.3 |
| Bolido | 4524 | 364 | 40.3 | 39.1 | 0.47 | 124.2 | 5.7 | 34.1 | 1.14 | 32.7 | 70.0 | 762 | 1205 | 1.49 | -0.38 | 238 | 35.3 | 164.1 | 2.8 | 2.7 | 129.3 |
| Bolo | 4549 | 363 | 34.2 | 47.8 | 0.46 | 124.4 | 6.9 | 33.7 | 1.41 | 31.3 | 76.7 | 684 | 1263 | 0.23 | -1.15 | 230 | 42.4 | 178.5 | 2.5 | 3.3 | 139.1 |
| Bonaerense Valverde | 4266 | 349 | 37.7 | 35.6 | 0.46 | 124.4 | 6.8 | 30.2 | 1.20 | 35.2 | 68.1 | 704 | 1091 | 1.43 | -0.35 | 212 | 39.6 | 163.5 | 2.8 | 2.3 | 133.7 |
| Bonitec | 4773 | 370 | 43.2 | 41.6 | 0.49 | 124.5 | 6.5 | 33.7 | 1.24 | 34.9 | 72.0 | 648 | 1353 | 1.08 | -0.27 | 204 | 36.7 | 150.9 | 2.7 | 4.8 | 127.2 |
| Bonzo | 4215 | 338 | 37.8 | 32.5 | 0.42 | 127.2 | 4.3 | 35.6 | 0.91 | 37.4 | 94.5 | 712 | 1000 | 2.46 | 0.11 | 243 | 26.6 | 179.1 | 2.7 | 1.5 | 128.5 |
| Boohai | 4150 | 337 | 29.1 | 44.5 | 0.41 | 124.9 | 5.8 | 32.1 | 1.39 | 38.3 | 103.4 | 684 | 1060 | 1.73 | -0.99 | 286 | 38.9 | 168.6 | 2.2 | 2.1 | 142.2 |
| Boreal | 4487 | 389 | 31.4 | 48.5 | 0.45 | 125.2 | 5.7 | 35.1 | 1.38 | 34.3 | 76.2 | 741 | 1308 | 3.27 | -0.69 | 235 | 42.8 | 177.7 | 2.7 | 3.1 | 144.9 |
| Borgia | 4475 | 325 | 41.8 | 39.7 | 0.46 | 127.6 | 4.7 | 34.6 | 1.16 | 32.0 | 71.7 | 684 | 1168 | 2.68 | -0.92 | 234 | 30.6 | 168.8 | 2.6 | 2.7 | 135.5 |
| Brachoua | 4488 | 341 | 34.9 | 46.4 | 0.48 | 124.1 | 6.1 | 34.1 | 1.36 | 35.5 | 80.6 | 700 | 1159 | 1.01 | -1.36 | 236 | 38.4 | 168.8 | 2.6 | 2.4 | 129.4 |
| Buck Candisur | 4678 | 339 | 35.2 | 46.1 | 0.46 | 126.9 | 5.8 | 31.8 | 1.44 | 37.9 | 78.9 | 733 | 1166 | 1.29 | -0.49 | 281 | 38.3 | 175.7 | 2.4 | 2.5 | 141.5 |
| Buck Cristal | 4532 | 338 | 33.4 | 44.5 | 0.47 | 123.9 | 5.2 | 34.4 | 1.30 | 41.5 | 86.1 | 747 | 1100 | 1.55 | -0.43 | 233 | 35.4 | 170.8 | 2.8 | 2.0 | 134.5 |
| Burgos | 4806 | 339 | 30.0 | 49.7 | 0.45 | 125.4 | 6.7 | 30.6 | 1.61 | 34.9 | 76.3 | 679 | 1087 | 2.15 | -1.04 | 236 | 45.4 | 171.8 | 2.4 | 2.6 | 136.4 |
| Camacho | 4736 | 320 | 29.8 | 51.2 | 0.44 | 128.2 | 5.4 | 34.2 | 1.50 | 36.2 | 80.8 | 713 | 1121 | 1.70 | 0.56 | 271 | 42.4 | 179.5 | 2.3 | 1.9 | 149.4 |
| Candeal de Salamanca | 3775 | 313 | 27.3 | 37.3 | 0.34 | 131.8 | 4.9 | 29.6 | 1.28 | 52.3 | 119.5 | 776 | 913 | -0.07 | 0.34 | 304 | 42.2 | 169.8 | 2.3 | 0.9 | 143.9 |
| Capeiti | 4281 | 368 | 38.4 | 44.1 | 0.44 | 121.5 | 6.7 | 36.0 | 1.25 | 47.0 | 88.5 | 780 | 1415 | 1.13 | -0.68 | 259 | 38.8 | 178.3 | 2.7 | 3.8 | 136.5 |
| Capeiti 8 | 4077 | 333 | 30.2 | 43.8 | 0.43 | 122.1 | 6.0 | 36.1 | 1.23 | 46.8 | 84.7 | 703 | 1020 | 1.40 | -0.75 | 234 | 35.6 | 175.6 | 2.7 | 1.9 | 126.6 |
| Carlantino | 4085 | 323 | 31.5 | 49.1 | 0.40 | 132.3 | 3.5 | 31.6 | 1.57 | 49.7 | 122.2 | 795 | 1216 | 0.31 | -1.09 | 290 | 30.4 | 157.9 | 2.5 | 2.7 | 147.8 |
| Carlo jucci | 4306 | 337 | 32.8 | 44.3 | 0.40 | 124.1 | 5.3 | 35.4 | 1.27 | 47.4 | 96.2 | 731 | 1197 | 1.43 | -0.53 | 262 | 33.2 | 183.5 | 2.5 | 2.7 | 148.4 |
| Carpio | 4799 | 337 | 33.3 | 48.1 | 0.47 | 121.7 | 6.2 | 35.5 | 1.36 | 33.0 | 74.0 | 721 | 1129 | -0.48 | -2.03 | 208 | 35.6 | 180.0 | 3.0 | 2.4 | 142.5 |
| Casiello | 4734 | 299 | 32.7 | 47.9 | 0.48 | 126.2 | 6.0 | 34.0 | 1.42 | 36.4 | 71.1 | 689 | 947 | 1.65 | -0.37 | 268 | 39.5 | 179.6 | 2.2 | 1.3 | 141.9 |
| Caxudo de sete espigas | 3801 | 294 | 39.1 | 40.9 | 0.37 | 133.4 | 3.8 | 31.2 | 1.36 | 48.7 | 116.0 | 807 | 1172 | 1.30 | -2.12 | 285 | 30.8 | 154.1 | 2.6 | 2.3 | 142.2 |
| Chacan | 4227 | 341 | 28.4 | 42.8 | 0.45 | 123.6 | 6.8 | 34.0 | 1.26 | 34.0 | 77.1 | 724 | 921 | 0.29 | -0.69 | 221 | 42.2 | 167.6 | 2.8 | 1.3 | 141.4 |
| Chagual INIA | 4633 | 328 | 30.0 | 44.7 | 0.47 | 123.5 | 6.6 | 33.6 | 1.33 | 35.4 | 71.7 | 722 | 989 | 1.33 | -0.16 | 217 | 38.7 | 169.8 | 2.8 | 1.5 | 135.0 |
| Chahba 88 | 4558 | 339 | 33.9 | 46.9 | 0.45 | 122.8 | 5.6 | 35.4 | 1.32 | 35.8 | 81.6 | 785 | 1174 | -0.05 | -1.13 | 226 | 33.9 | 176.3 | 3.1 | 2.2 | 136.8 |
| Cham-1 | 4416 | 335 | 37.5 | 39.2 | 0.47 | 125.5 | 5.5 | 33.5 | 1.17 | 33.2 | 77.0 | 729 | 1032 | 3.22 | -1.82 | 193 | 36.3 | 162.4 | 3.3 | 1.9 | 126.2 |
| Chanst | 4890 | 336 | 30.8 | 48.9 | 0.46 | 123.2 | 6.5 | 34.4 | 1.41 | 37.8 | 83.9 | 698 | 1066 | 1.29 | -0.43 | 233 | 41.2 | 183.9 | 2.6 | 2.0 | 133.5 |
| Chonta INIA | 4665 | 354 | 37.7 | 41.8 | 0.44 | 123.9 | 6.8 | 32.6 | 1.29 | 34.7 | 79.2 | 746 | 1275 | 2.14 | -0.52 | 270 | 42.0 | 175.4 | 2.4 | 3.1 | 138.0 |
| Cicirelo | 3919 | 360 | 27.7 | 48.6 | 0.36 | 130.7 | 4.9 | 30.9 | 1.60 | 51.7 | 122.5 | 775 | 1336 | 1.04 | -0.01 | 285 | 43.7 | 166.5 | 2.4 | 3.2 | 148.7 |
| Cirillo | 4459 | 325 | 32.2 | 44.4 | 0.44 | 124.1 | 5.6 | 35.6 | 1.26 | 31.1 | 74.9 | 711 | 1043 | 1.37 | -0.20 | 246 | 34.9 | 179.7 | 2.6 | 1.8 | 145.4 |
| Claro de Balazote | 4156 | 351 | 33.1 | 43.2 | 0.33 | 136.5 | 2.9 | 29.8 | 1.48 | 49.0 | 128.1 | 820 | 1654 | -1.74 | 0.23 | 310 | 29.2 | 162.2 | 2.4 | 5.6 | 145.3 |
| Clarofino | 4304 | 353 | 35.8 | 42.4 | 0.37 | 133.4 | 4.1 | 30.3 | 1.44 | 51.0 | 121.9 | 741 | 1430 | -0.68 | -0.68 | 319 | 36.2 | 167.6 | 2.1 | 4.1 | 138.2 |
| Claudio | 5035 | 353 | 35.6 | 44.3 | 0.45 | 124.3 | 6.3 | 33.8 | 1.30 | 35.3 | 82.1 | 709 | 1199 | 1.45 | -0.18 | 251 | 41.7 | 179.1 | 2.5 | 2.6 | 142.8 |
| Cobros | 4093 | 319 | 34.0 | 40.0 | 0.38 | 124.6 | 5.1 | 33.4 | 1.20 | 42.1 | 93.0 | 723 | 1104 | 1.31 | -0.08 | 253 | 37.2 | 172.2 | 2.5 | 2.7 | 134.6 |
| Colorado | 4114 | 340 | 31.3 | 42.1 | 0.45 | 126.5 | 5.1 | 34.0 | 1.25 | 32.5 | 71.9 | 693 | 997 | 0.91 | -1.65 | 250 | 33.5 | 173.1 | 2.5 | 1.6 | 136.9 |
| Colorado de Jerez | 4327 | 327 | 31.2 | 48.9 | 0.39 | 130.0 | 4.2 | 33.5 | 1.49 | 52.1 | 121.8 | 794 | 1272 | 4.22 | 0.20 | 324 | 28.5 | 179.3 | 2.3 | 2.9 | 144.3 |
| Commander | 4763 | 340 | 29.0 | 45.5 | 0.47 | 125.0 | 5.5 | 33.0 | 1.38 | 34.2 | 78.5 | 745 | 1002 | 1.10 | -0.54 | 251 | 36.5 | 178.8 | 2.6 | 1.6 | 139.6 |
| Cortez | 4470 | 355 | 32.6 | 45.4 | 0.46 | 122.2 | 7.3 | 34.0 | 1.33 | 31.6 | 69.7 | 684 | 1122 | 1.31 | -1.02 | 210 | 42.7 | 169.1 | 2.9 | 2.3 | 132.8 |
| Creso | 4288 | 336 | 34.4 | 43.8 | 0.41 | 128.5 | 4.9 | 33.1 | 1.32 | 40.3 | 91.9 | 709 | 1227 | 1.76 | 0.28 | 262 | 30.9 | 177.5 | 2.5 | 3.1 | 129.5 |
| D-2 | 3291 | 371 | 35.6 | 34.2 | 0.40 | 128.7 | 3.5 | 32.4 | 1.09 | 38.9 | 88.0 | 687 | 1124 | 1.28 | 0.02 | 265 | 24.4 | 154.8 | 2.4 | 3.0 | 123.1 |
| Dalmatia 1 | 3398 | 320 | 27.1 | 45.8 | 0.37 | 133.1 | 5.1 | 29.9 | 1.55 | 44.7 | 114.2 | 678 | 1038 | -0.54 | -1.86 | 279 | 44.8 | 146.3 | 2.2 | 2.2 | 148.5 |
| Dalmatia 3 | 4577 | 335 | 28.7 | 46.0 | 0.37 | 136.2 | 3.5 | 30.8 | 1.54 | 46.7 | 126.1 | 782 | 1176 | -1.27 | 0.54 | 287 | 31.9 | 157.5 | 2.6 | 2.5 | 149.1 |
| De Santa Marta | 3565 | 344 | 32.8 | 40.5 | 0.38 | 136.9 | 4.2 | 29.1 | 1.41 | 51.3 | 123.8 | 760 | 1302 | -1.61 | 0.41 | 300 | 30.0 | 160.9 | 2.4 | 2.9 | 151.0 |
| Debano | 4466 | 337 | 32.6 | 42.8 | 0.47 | 121.0 | 6.3 | 36.7 | 1.16 | 33.6 | 71.8 | 663 | 983 | 0.48 | -1.07 | 210 | 30.1 | 181.9 | 2.8 | 1.7 | 130.7 |
| Derra | 4355 | 315 | 27.8 | 50.1 | 0.47 | 124.0 | 6.0 | 36.1 | 1.38 | 34.8 | 77.9 | 686 | 919 | 1.06 | -0.20 | 213 | 34.7 | 177.0 | 2.8 | 1.2 | 141.8 |
| Dezassete | 3482 | 366 | 26.9 | 44.1 | 0.30 | 132.2 | 5.4 | 31.8 | 1.43 | 47.8 | 120.2 | 762 | 1461 | -0.17 | -0.58 | 320 | 40.4 | 175.6 | 2.1 | 3.7 | 157.2 |
| Donduro | 4312 | 336 | 28.7 | 47.1 | 0.44 | 123.6 | 7.5 | 32.2 | 1.46 | 34.7 | 74.6 | 723 | 988 | 1.43 | -0.39 | 213 | 42.1 | 178.9 | 2.8 | 1.4 | 130.3 |
| Douro Boukowo | 3770 | 342 | 28.9 | 44.3 | 0.36 | 130.1 | 4.3 | 30.1 | 1.50 | 49.2 | 121.0 | 813 | 1207 | 3.09 | 0.99 | 276 | 31.6 | 157.3 | 2.7 | 2.4 | 144.5 |
| Du Maroc Battandier | 3852 | 358 | 37.7 | 46.3 | 0.40 | 133.7 | 4.0 | 30.9 | 1.52 | 49.1 | 114.8 | 770 | 1578 | -0.69 | -0.67 | 296 | 34.5 | 160.7 | 2.4 | 5.2 | 138.6 |
| Dur de Medeah | 4167 | 331 | 35.4 | 39.5 | 0.36 | 133.3 | 5.2 | 31.2 | 1.28 | 52.7 | 128.5 | 725 | 1267 | 0.26 | 1.38 | 329 | 39.3 | 174.7 | 2.0 | 3.1 | 145.8 |
| Duradero | 4548 | 355 | 43.4 | 38.3 | 0.48 | 123.7 | 7.2 | 33.8 | 1.13 | 36.2 | 77.3 | 826 | 1278 | 1.99 | -0.02 | 207 | 42.2 | 151.7 | 3.3 | 3.0 | 129.2 |
| Duraking | 4480 | 318 | 37.4 | 42.8 | 0.43 | 122.7 | 7.5 | 32.3 | 1.31 | 33.1 | 76.7 | 703 | 1136 | 1.44 | -1.42 | 224 | 41.7 | 174.5 | 2.7 | 2.7 | 132.8 |
| Durazio Rijo | 3995 | 320 | 27.9 | 45.1 | 0.37 | 128.3 | 4.4 | 31.0 | 1.51 | 46.3 | 117.1 | 721 | 1061 | 3.11 | -0.81 | 262 | 26.2 | 150.5 | 2.5 | 2.0 | 138.1 |
| Durazio Rijo Glabro | 3513 | 347 | 29.1 | 36.7 | 0.39 | 128.2 | 3.3 | 30.1 | 1.24 | 35.3 | 84.8 | 696 | 941 | 3.29 | -0.50 | 279 | 27.9 | 163.4 | 2.3 | 1.4 | 129.9 |
| Durex | 4524 | 338 | 32.7 | 44.2 | 0.46 | 120.4 | 6.5 | 36.0 | 1.23 | 32.1 | 76.5 | 743 | 1036 | 1.12 | -1.60 | 211 | 32.7 | 185.2 | 3.1 | 1.5 | 142.7 |
| Enano de Andújar | 3349 |  | 25.6 | 60.4 | 0.37 | 132.6 | 4.3 | 31.2 | 1.87 | 49.7 | 115.8 | 777 | 1141 | -0.63 | 0.73 | 283 | 37.9 | 167.6 | 2.5 | 2.2 | 136.1 |
| Enduro | 4373 | 349 | 32.6 | 42.2 | 0.45 | 124.8 | 7.0 | 31.8 | 1.33 | 35.1 | 79.5 | 700 | 1104 | 0.79 | -0.16 | 263 | 43.5 | 170.4 | 2.3 | 2.3 | 151.1 |
| Entrelargo de Montijo | 4105 | 322 | 26.5 | 49.9 | 0.38 | 131.3 | 3.2 | 32.1 | 1.58 | 44.4 | 100.6 | 709 | 1086 | 3.21 | 0.29 | 288 | 23.1 | 165.0 | 2.4 | 2.0 | 153.3 |
| Epidur | 4806 | 366 | 39.8 | 48.2 | 0.48 | 124.4 | 5.9 | 34.7 | 1.39 | 34.9 | 73.6 | 866 | 1465 | 1.62 | -1.45 | 215 | 36.4 | 162.8 | 3.5 | 4.5 | 136.1 |
| Espanhol | 4011 | 316 | 32.3 | 42.8 | 0.36 | 134.8 | 4.1 | 30.1 | 1.44 | 56.7 | 117.7 | 739 | 1180 | -1.15 | -1.43 | 294 | 34.5 | 164.7 | 2.3 | 2.6 | 138.3 |
| Esquilache | 4917 | 348 | 35.1 | 43.3 | 0.46 | 121.9 | 6.9 | 35.7 | 1.23 | 33.3 | 70.9 | 674 | 1130 | 0.95 | -0.74 | 239 | 37.0 | 179.8 | 2.4 | 2.4 | 137.2 |
| Etith | 3855 | 379 | 27.6 | 42.8 | 0.42 | 119.0 | 7.2 | 33.0 | 1.32 | 44.7 | 83.3 | 685 | 1065 | -0.71 | -2.07 | 216 | 40.0 | 184.6 | 2.7 | 1.8 | 131.8 |
| Euroduro | 4588 | 369 | 24.3 | 41.7 | 0.47 | 124.5 | 7.3 | 30.9 | 1.35 | 33.5 | 72.0 | 742 | 1005 | 2.12 | -0.90 | 227 | 45.1 | 168.6 | 2.7 | 1.7 | 138.9 |
| Excalibur | 4451 | 329 | 43.0 | 37.0 | 0.49 | 124.2 | 7.4 | 33.2 | 1.11 | 25.8 | 71.7 | 681 | 1062 | 1.34 | -0.81 | 204 | 44.9 | 167.0 | 2.8 | 2.6 | 122.1 |
| Fardes | 4441 | 347 | 28.4 | 47.2 | 0.49 | 123.1 | 6.8 | 33.8 | 1.40 | 33.6 | 72.8 | 760 | 939 | 0.80 | 0.62 | 218 | 43.9 | 163.8 | 2.9 | 1.3 | 134.8 |
| Fartó | 4273 | 302 | 34.6 | 45.0 | 0.39 | 132.1 | 3.5 | 30.5 | 1.50 | 50.7 | 125.1 | 788 | 1175 | 1.26 | -1.05 | 305 | 27.7 | 158.2 | 2.4 | 2.7 | 143.2 |
| Farto cañifino | 3999 | 308 | 36.3 | 41.7 | 0.37 | 134.1 | 4.1 | 30.2 | 1.41 | 48.1 | 112.3 | 810 | 1212 | -1.97 | -1.35 | 300 | 36.0 | 160.6 | 2.4 | 3.0 | 149.5 |
| Fenice | 4374 | 299 | 23.4 | 52.5 | 0.46 | 122.1 | 6.4 | 34.9 | 1.51 | 33.6 | 71.7 | 673 | 776 | 0.54 | -2.16 | 223 | 32.0 | 171.3 | 2.7 | 1.0 | 143.4 |
| Fjord | 4364 | 382 | 33.8 | 42.0 | 0.44 | 125.9 | 6.4 | 30.8 | 1.38 | 41.8 | 96.9 | 811 | 1282 | 3.25 | -0.73 | 207 | 42.8 | 174.5 | 3.3 | 2.4 | 139.3 |
| Flavio | 4293 | 287 | 30.4 | 45.4 | 0.45 | 127.4 | 4.2 | 34.0 | 1.34 | 22.8 | 61.2 | 665 | 842 | 1.10 | 0.62 | 245 | 32.7 | 176.4 | 2.4 | 1.0 | 130.5 |
| Flodur | 4387 | 345 | 37.3 | 37.0 | 0.45 | 135.0 | 4.2 | 32.1 | 1.16 | 30.9 | 61.0 | 729 | 1047 | -4.16 | -1.03 | 226 | 28.2 | 154.5 | 3.0 | 2.5 | 129.2 |
| Fortore | 4794 | 322 | 34.9 | 46.5 | 0.46 | 123.4 | 5.9 | 35.1 | 1.43 | 34.4 | 79.8 | 770 | 1091 | 1.48 | 0.52 | 255 | 41.2 | 204.1 | 2.6 | 1.3 | 141.9 |
| Gallareta | 4419 | 366 | 39.6 | 40.2 | 0.48 | 125.5 | 7.3 | 31.8 | 1.27 | 31.9 | 68.5 | 696 | 1236 | 2.74 | -1.26 | 226 | 44.5 | 144.1 | 2.6 | 3.4 | 126.8 |
| Girgeh | 3634 | 329 | 28.7 | 38.7 | 0.40 | 124.7 | 6.6 | 30.9 | 1.26 | 34.0 | 80.4 | 749 | 922 | 1.94 | -0.25 | 232 | 41.3 | 155.7 | 2.8 | 1.1 | 123.2 |
| Giza 2 | 3754 | 342 | 31.5 | 41.4 | 0.40 | 128.3 | 3.9 | 30.5 | 1.38 | 35.1 | 85.9 | 725 | 1151 | 2.09 | -0.13 | 264 | 30.8 | 176.4 | 2.5 | 1.9 | 123.0 |
| Grecale | 4426 | 325 | 24.7 | 48.1 | 0.44 | 122.3 | 6.1 | 34.0 | 1.42 | 34.4 | 76.1 | 703 | 863 | 1.48 | -0.29 | 223 | 38.0 | 169.6 | 2.7 | 1.0 | 139.8 |
| Greece 14 | 4629 | 329 | 26.1 | 53.4 | 0.40 | 132.6 | 4.8 | 30.7 | 1.75 | 45.5 | 113.9 | 673 | 1106 | -0.65 | -1.41 | 300 | 41.4 | 164.0 | 2.0 | 2.4 | 154.7 |
| Greece 23 | 4433 | 309 | 30.5 | 53.2 | 0.38 | 132.6 | 3.7 | 32.3 | 1.67 | 42.6 | 116.5 | 819 | 1286 | 2.57 | 0.83 | 311 | 28.6 | 197.5 | 2.4 | 3.1 | 142.2 |
| Greece 24 | 3525 | 306 | 27.4 | 47.8 | 0.35 | 130.5 | 3.6 | 32.3 | 1.53 | 55.2 | 121.1 | 787 | 1120 | 3.37 | 0.94 | 287 | 25.8 | 167.5 | 2.6 | 2.4 | 145.0 |
| Griego de Baleares | 3764 | 304 | 30.0 | 38.4 | 0.39 | 134.4 | 4.7 | 31.1 | 1.25 | 50.1 | 120.6 | 794 | 887 | -0.16 | 0.01 | 272 | 35.5 | 156.8 | 2.7 | 0.9 | 133.2 |
| Gros de Cerdaña | 3574 | 308 | 34.2 | 39.4 | 0.36 | 137.4 | 5.0 | 30.3 | 1.32 | 58.5 | 143.5 | 764 | 1156 | -1.01 | 1.68 | 320 | 36.6 | 151.1 | 2.2 | 2.6 | 141.7 |
| Guayacan INIA | 4545 | 324 | 31.4 | 45.2 | 0.45 | 122.5 | 7.4 | 34.0 | 1.34 | 37.0 | 80.7 | 699 | 1053 | 1.95 | -0.66 | 233 | 44.2 | 177.0 | 2.5 | 1.9 | 131.0 |
| Guerou-1 | 4559 | 352 | 30.9 | 48.4 | 0.46 | 122.6 | 8.5 | 34.5 | 1.40 | 32.0 | 73.9 | 702 | 1140 | 1.24 | -0.37 | 227 | 54.9 | 178.8 | 2.5 | 2.4 | 140.2 |
| Hamira | 4091 | 325 | 27.6 | 49.5 | 0.34 | 131.4 | 3.8 | 31.9 | 1.60 | 51.3 | 120.3 | 893 | 1268 | 4.09 | 0.60 | 318 | 27.9 | 169.1 | 2.6 | 2.5 | 149.0 |
| Harani Auttma | 4111 | 371 | 36.0 | 40.9 | 0.42 | 122.7 | 6.5 | 33.0 | 1.26 | 45.4 | 93.2 | 678 | 1298 | 1.74 | -1.17 | 244 | 39.8 | 171.1 | 2.4 | 3.6 | 138.0 |
| Hati | 3911 | 335 | 32.8 | 39.9 | 0.40 | 122.6 | 6.3 | 31.8 | 1.29 | 41.9 | 93.9 | 704 | 1110 | 0.90 | -0.34 | 211 | 33.1 | 169.0 | 2.9 | 2.7 | 131.9 |
| Haurani 79-b | 4340 | 305 | 27.9 | 51.9 | 0.38 | 132.3 | 4.6 | 30.4 | 1.73 | 46.8 | 117.3 | 739 | 1115 | -0.18 | -0.06 | 317 | 36.1 | 180.6 | 2.1 | 2.1 | 152.6 |
| Hazera | 4335 | 338 | 32.2 | 43.6 | 0.45 | 119.9 | 6.4 | 34.2 | 1.28 | 35.2 | 77.5 | 699 | 1087 | 1.27 | -0.71 | 209 | 38.8 | 171.6 | 2.7 | 2.2 | 143.6 |
| Heraldo del Rhin | 3615 | 282 | 34.7 | 37.2 | 0.35 | 134.7 | 3.6 | 30.2 | 1.26 | 53.2 | 138.5 | 709 | 991 | -0.89 | -1.71 | 294 | 30.6 | 149.3 | 2.3 | 2.1 | 138.7 |
| Hispasano | 4552 | 320 | 29.6 | 50.4 | 0.44 | 122.7 | 5.6 | 35.2 | 1.43 | 32.6 | 74.3 | 722 | 1103 | 0.49 | -1.25 | 225 | 32.1 | 185.8 | 2.8 | 1.6 | 138.3 |
| Hora | 4223 | 346 | 34.3 | 39.1 | 0.42 | 123.1 | 6.1 | 33.6 | 1.18 | 36.5 | 78.2 | 682 | 1114 | 1.87 | -0.67 | 210 | 37.0 | 164.5 | 2.7 | 2.7 | 144.0 |
| Horani Howawi | 3144 | 347 | 24.6 | 44.0 | 0.42 | 123.4 | 5.3 | 33.8 | 1.31 | 43.7 | 92.6 | 713 | 843 | 1.39 | -0.57 | 252 | 33.2 | 166.1 | 2.6 | 1.0 | 151.3 |
| Hourah | 3935 | 347 | 31.6 | 39.7 | 0.41 | 126.3 | 5.5 | 33.4 | 1.23 | 40.1 | 88.8 | 701 | 1059 | 0.78 | 0.42 | 252 | 32.1 | 174.0 | 2.5 | 2.4 | 129.2 |
| Hymera | 4291 | 316 | 36.4 | 43.6 | 0.42 | 121.8 | 6.7 | 34.2 | 1.30 | 42.0 | 84.5 | 698 | 1191 | 1.16 | -0.91 | 226 | 32.6 | 164.4 | 2.7 | 3.4 | 136.9 |
| IG-83905 | 3571 | 341 | 30.4 | 36.7 | 0.35 | 136.6 | 3.4 | 30.2 | 1.25 | 45.2 | 106.9 | 755 | 1078 | -1.28 | -0.67 | 306 | 28.1 | 168.8 | 2.3 | 1.8 | 142.0 |
| IG-83920 | 3843 | 378 | 25.0 | 39.5 | 0.40 | 131.4 | 4.6 | 30.8 | 1.31 | 36.9 | 88.4 | 657 | 949 | -1.66 | -2.48 | 239 | 24.9 | 145.4 | 2.6 | 1.9 | 136.8 |
| IG-84856 | 4383 | 306 | 26.9 | 51.6 | 0.39 | 129.1 | 4.7 | 34.0 | 1.55 | 47.0 | 113.2 | 745 | 1064 | 3.50 | 0.62 | 288 | 35.5 | 185.4 | 2.4 | 1.6 | 155.8 |
| IG-92895 | 3993 | 308 | 34.0 | 50.3 | 0.37 | 131.1 | 3.4 | 33.5 | 1.52 | 47.3 | 111.0 | 796 | 1380 | 3.49 | 1.61 | 285 | 21.3 | 167.1 | 2.6 | 3.5 | 139.3 |
| IG-92967 | 4157 | 332 | 36.7 | 42.6 | 0.39 | 134.7 | 3.6 | 30.4 | 1.44 | 47.7 | 109.3 | 741 | 1362 | -1.30 | -1.14 | 304 | 30.4 | 156.4 | 2.3 | 4.9 | 141.6 |
| IG-93030 | 4079 | 306 | 29.4 | 49.7 | 0.36 | 132.9 | 3.5 | 31.3 | 1.61 | 46.3 | 103.0 | 769 | 1253 | 1.15 | -0.29 | 307 | 32.5 | 171.4 | 2.3 | 2.7 | 154.2 |
| IG-93621 | 4105 | 345 | 24.9 | 47.6 | 0.37 | 132.7 | 1.9 | 31.7 | 1.52 | 39.4 | 96.3 | 737 | 1102 | 3.25 | 0.52 | 314 | 22.4 | 179.1 | 2.2 | 2.1 | 155.0 |
| IG-94009 | 4154 | 330 | 29.1 | 44.1 | 0.34 | 132.9 | 4.4 | 30.5 | 1.45 | 42.5 | 105.1 | 823 | 1251 | -0.05 | -0.39 | 315 | 38.5 | 164.9 | 2.4 | 2.3 | 149.1 |
| IG-95812 | 3754 | 335 | 28.6 | 44.4 | 0.42 | 122.8 | 6.9 | 32.8 | 1.37 | 46.7 | 94.0 | 709 | 1024 | -0.33 | -1.28 | 225 | 41.3 | 168.5 | 2.7 | 1.8 | 138.8 |
| IG-95841 | 4229 | 341 | 30.1 | 45.9 | 0.40 | 134.5 | 3.9 | 30.5 | 1.53 | 40.5 | 99.7 | 731 | 1177 | -0.78 | -0.41 | 286 | 33.0 | 163.3 | 2.3 | 3.0 | 144.1 |
| IG-95847 | 3987 | 327 | 31.0 | 43.1 | 0.41 | 123.1 | 6.0 | 34.4 | 1.27 | 42.5 | 90.7 | 696 | 1039 | 0.42 | -0.70 | 268 | 36.0 | 174.2 | 2.3 | 2.0 | 143.1 |
| IG-95931 | 4417 | 388 | 29.3 | 51.4 | 0.43 | 124.5 | 6.3 | 33.0 | 1.56 | 44.3 | 88.8 | 795 | 1335 | 1.63 | -0.16 | 267 | 43.8 | 176.7 | 2.6 | 3.5 | 136.3 |
| IG-96802 | 4399 | 309 | 30.0 | 48.8 | 0.36 | 130.4 | 3.9 | 32.7 | 1.51 | 45.8 | 109.1 | 810 | 1197 | 2.58 | 0.77 | 269 | 24.7 | 175.9 | 2.8 | 1.9 | 144.7 |
| IG-96851 | 4001 | 365 | 32.0 | 44.0 | 0.40 | 132.3 | 4.5 | 29.2 | 1.54 | 38.9 | 107.5 | 689 | 1277 | -0.75 | -1.43 | 272 | 34.7 | 157.9 | 2.3 | 3.5 | 138.5 |
| II/10 | 3584 | 312 | 31.7 | 40.7 | 0.38 | 132.2 | 3.1 | 30.3 | 1.37 | 37.3 | 87.6 | 750 | 1047 | -1.18 | -0.94 | 277 | 23.4 | 161.9 | 2.5 | 1.7 | 131.5 |
| II/4 | 4252 | 321 | 27.8 | 45.3 | 0.35 | 132.0 | 5.4 | 29.7 | 1.55 | 54.7 | 123.9 | 885 | 1135 | 0.11 | -1.15 | 265 | 39.6 | 174.2 | 3.1 | 1.6 | 137.0 |
| Imhotep | 4298 | 354 | 31.8 | 42.4 | 0.47 | 122.1 | 7.3 | 33.0 | 1.28 | 29.9 | 72.6 | 693 | 998 | -0.78 | -0.17 | 171 | 36.7 | 152.3 | 3.5 | 2.3 | 115.7 |
| Iumillo | 3470 | 330 | 31.5 | 33.2 | 0.36 | 133.3 | 5.4 | 30.2 | 1.11 | 53.8 | 115.1 | 747 | 955 | -1.41 | -2.04 | 274 | 40.6 | 166.0 | 2.5 | 1.1 | 131.6 |
| Jabato | 4561 | 374 | 37.6 | 42.9 | 0.49 | 123.5 | 6.7 | 34.9 | 1.22 | 34.3 | 73.0 | 674 | 1217 | 2.50 | 0.10 | 221 | 37.8 | 145.1 | 2.6 | 3.7 | 125.0 |
| Jaguar | 4360 | 341 | 33.0 | 50.0 | 0.45 | 125.9 | 6.3 | 32.3 | 1.58 | 29.7 | 69.7 | 719 | 1223 | 1.00 | -0.12 | 225 | 39.8 | 169.6 | 2.8 | 3.4 | 138.3 |
| Jennah Khetifa Rp4 | 4249 | 334 | 33.2 | 43.1 | 0.36 | 131.9 | 4.2 | 31.5 | 1.42 | 52.5 | 130.9 | 797 | 1312 | 2.16 | 0.64 | 331 | 31.8 | 176.3 | 2.2 | 2.8 | 146.7 |
| Jiloca | 4910 | 343 | 33.7 | 52.7 | 0.44 | 124.6 | 6.3 | 35.7 | 1.47 | 38.4 | 85.2 | 734 | 1374 | 1.27 | 0.72 | 282 | 45.0 | 199.7 | 2.3 | 3.1 | 151.4 |
| JM-3987 | 3587 | 396 | 30.5 | 39.6 | 0.40 | 121.5 | 5.8 | 31.8 | 1.25 | 35.1 | 76.0 | 749 | 1204 | 1.19 | -0.71 | 210 | 32.5 | 165.6 | 3.2 | 2.7 | 130.8 |
| JM-3989 | 3466 | 386 | 28.3 | 40.7 | 0.41 | 121.8 | 5.9 | 31.4 | 1.30 | 35.0 | 78.7 | 679 | 1081 | 0.43 | -0.17 | 247 | 34.7 | 169.1 | 2.4 | 2.6 | 134.6 |
| Juljulith | 4010 | 367 | 24.3 | 45.1 | 0.40 | 125.3 | 6.2 | 28.2 | 1.62 | 36.5 | 90.4 | 766 | 1015 | -0.02 | -0.90 | 250 | 45.2 | 157.5 | 2.6 | 1.6 | 130.4 |
| Jupare | 4835 | 360 | 41.3 | 42.4 | 0.48 | 123.9 | 7.1 | 32.4 | 1.32 | 34.9 | 74.4 | 726 | 1342 | 0.65 | -1.00 | 218 | 45.5 | 167.6 | 2.8 | 3.5 | 130.6 |
| Kabir1 | 4693 | 305 | 30.5 | 49.9 | 0.42 | 124.6 | 6.1 | 35.3 | 1.42 | 35.6 | 80.3 | 799 | 1059 | 1.20 | -0.05 | 236 | 38.1 | 176.9 | 2.9 | 1.8 | 145.4 |
| Kalka | 4160 | 341 | 35.8 | 41.8 | 0.43 | 127.5 | 5.0 | 33.6 | 1.26 | 35.9 | 78.9 | 712 | 1193 | 1.19 | -0.11 | 268 | 31.8 | 178.6 | 2.4 | 2.8 | 140.7 |
| Karim | 4197 | 324 | 29.9 | 48.1 | 0.48 | 122.2 | 6.9 | 33.3 | 1.46 | 33.1 | 68.8 | 670 | 955 | 1.14 | 0.12 | 201 | 39.6 | 160.9 | 2.8 | 2.0 | 131.2 |
| Karim 80 | 4535 | 343 | 30.6 | 47.4 | 0.48 | 121.5 | 5.3 | 33.8 | 1.41 | 36.2 | 72.9 | 735 | 1064 | -0.91 | -0.79 | 230 | 36.9 | 171.0 | 2.7 | 1.8 | 144.1 |
| Khabur-1 | 4756 | 327 | 27.7 | 48.0 | 0.47 | 122.6 | 7.1 | 33.2 | 1.44 | 41.1 | 82.7 | 780 | 915 | -0.48 | -0.01 | 252 | 47.9 | 184.0 | 2.6 | 1.1 | 155.1 |
| Khiar 92 | 4805 | 331 | 37.5 | 40.5 | 0.48 | 122.5 | 5.2 | 36.0 | 1.14 | 37.7 | 76.3 | 698 | 1117 | 1.72 | -0.48 | 194 | 36.1 | 178.0 | 3.0 | 2.3 | 138.4 |
| Kidur | 4409 | 361 | 32.1 | 40.8 | 0.41 | 127.9 | 4.6 | 33.7 | 1.22 | 32.6 | 74.4 | 837 | 1172 | 3.18 | 0.03 | 285 | 38.5 | 197.4 | 2.6 | 1.3 | 141.9 |
| Kronos | 4751 | 340 | 39.0 | 38.7 | 0.49 | 119.5 | 7.4 | 34.7 | 1.28 | 33.0 | 73.5 | 653 | 1153 | 0.87 | -1.42 | 210 | 36.0 | 168.3 | 2.7 | 3.1 | 134.6 |
| Lagonil-2 | 4659 | 303 | 32.9 | 48.4 | 0.47 | 124.3 | 5.9 | 34.2 | 1.41 | 34.7 | 78.8 | 661 | 985 | -0.19 | -0.79 | 231 | 34.3 | 167.2 | 2.5 | 1.8 | 136.9 |
| Lagost 3 | 4463 | 324 | 29.3 | 54.0 | 0.47 | 122.8 | 6.4 | 34.5 | 1.56 | 29.5 | 72.3 | 720 | 1063 | 0.69 | -0.26 | 239 | 36.1 | 179.7 | 2.6 | 1.9 | 133.5 |
| Lahn | 4744 | 331 | 36.5 | 53.4 | 0.46 | 125.3 | 6.4 | 32.9 | 1.61 | 36.1 | 77.3 | 745 | 1401 | 2.44 | -1.37 | 252 | 47.8 | 169.2 | 2.5 | 4.0 | 143.3 |
| Lakota | 3664 | 355 | 40.8 | 32.5 | 0.41 | 130.9 | 6.2 | 30.5 | 1.08 | 41.8 | 98.5 | 722 | 1158 | -0.94 | -0.11 | 242 | 40.7 | 151.8 | 2.6 | 2.6 | 130.0 |
| Lloyd | 4706 | 337 | 33.8 | 44.3 | 0.45 | 129.1 | 4.0 | 34.8 | 1.28 | 31.1 | 71.9 | 773 | 1109 | 2.96 | -0.68 | 238 | 26.0 | 177.6 | 3.0 | 1.8 | 128.1 |
| Lobeiro de grao escuro | 3469 | 348 | 28.7 | 43.0 | 0.37 | 133.5 | 4.2 | 30.8 | 1.42 | 39.8 | 98.2 | 676 | 1173 | -0.66 | -0.54 | 322 | 36.4 | 166.2 | 1.9 | 2.9 | 151.4 |
| Loukos-1 | 4256 | 314 | 29.8 | 49.5 | 0.44 | 123.0 | 7.6 | 33.1 | 1.50 | 35.8 | 72.2 | 644 | 1037 | -0.38 | -0.88 | 213 | 46.5 | 154.8 | 2.5 | 2.7 | 131.0 |
| Louri AP 5 | 3895 | 354 | 24.3 | 46.1 | 0.35 | 132.5 | 4.3 | 29.9 | 1.57 | 50.4 | 126.7 | 713 | 1135 | 1.48 | -2.17 | 294 | 32.3 | 154.8 | 2.3 | 2.4 | 155.2 |
| Lozen 76 | 4112 | 349 | 31.4 | 43.5 | 0.39 | 133.2 | 3.2 | 31.4 | 1.42 | 29.4 | 81.4 | 692 | 1225 | -0.26 | 0.14 | 279 | 21.2 | 151.3 | 2.3 | 3.8 | 145.0 |
| Mâali | 4939 | 327 | 38.0 | 37.6 | 0.48 | 121.1 | 6.6 | 35.6 | 1.08 | 40.0 | 86.1 | 745 | 1054 | 0.82 | -0.05 | 207 | 41.1 | 176.8 | 2.9 | 1.6 | 139.6 |
| Maamouri-1 | 4598 | 333 | 30.0 | 48.2 | 0.45 | 121.9 | 7.6 | 34.2 | 1.42 | 36.9 | 80.1 | 649 | 1061 | 0.96 | -1.73 | 222 | 41.9 | 164.3 | 2.5 | 2.1 | 140.1 |
| Macoun | 4205 | 321 | 28.4 | 48.6 | 0.37 | 130.1 | 4.3 | 32.1 | 1.54 | 44.7 | 112.5 | 752 | 1164 | 2.71 | 0.41 | 270 | 31.0 | 168.1 | 2.5 | 2.6 | 148.0 |
| Maghoussa | 4075 | 334 | 27.0 | 43.2 | 0.33 | 130.9 | 3.5 | 32.4 | 1.35 | 42.5 | 102.8 | 715 | 1172 | 3.35 | -0.23 | 245 | 21.9 | 180.5 | 2.7 | 2.8 | 139.4 |
| Maghoussa Amizmiz | 3767 | 337 | 27.6 | 47.1 | 0.38 | 128.4 | 3.9 | 32.5 | 1.49 | 47.4 | 110.2 | 779 | 1118 | 1.22 | 0.69 | 245 | 23.4 | 170.0 | 3.0 | 1.9 | 131.3 |
| Mahmoudi C | 4216 | 340 | 27.3 | 47.9 | 0.36 | 131.9 | 3.3 | 31.4 | 1.55 | 43.8 | 105.1 | 706 | 1214 | 1.70 | -0.03 | 287 | 26.7 | 179.7 | 2.3 | 2.6 | 152.9 |
| Malavika | 5178 | 348 | 35.2 | 47.1 | 0.49 | 124.1 | 5.6 | 33.9 | 1.40 | 35.9 | 71.4 | 711 | 1196 | 0.09 | -0.53 | 246 | 37.1 | 183.3 | 2.6 | 2.8 | 150.6 |
| Malvaraj | 4275 | 344 | 32.1 | 47.1 | 0.44 | 124.8 | 7.8 | 32.3 | 1.45 | 34.5 | 64.2 | 728 | 1168 | 0.56 | -0.85 | 238 | 45.4 | 164.0 | 2.6 | 2.7 | 137.7 |
| Marou | 4658 | 335 | 28.8 | 52.1 | 0.46 | 123.8 | 5.9 | 34.5 | 1.53 | 36.4 | 80.7 | 700 | 1021 | 1.99 | -0.53 | 228 | 38.9 | 175.3 | 2.6 | 1.6 | 145.1 |
| Marques | 4091 | 319 | 27.3 | 47.5 | 0.36 | 132.8 | 3.8 | 32.2 | 1.48 | 43.7 | 106.5 | 790 | 1123 | 0.70 | 0.16 | 320 | 33.4 | 162.9 | 2.3 | 1.8 | 150.6 |
| Marsyr-1 | 3608 | 332 | 25.7 | 41.7 | 0.41 | 121.4 | 7.9 | 32.8 | 1.27 | 30.9 | 67.2 | 668 | 843 | 1.13 | -0.80 | 212 | 37.9 | 145.9 | 2.7 | 1.5 | 137.8 |
| Massa | 3895 | 323 | 24.6 | 49.4 | 0.44 | 122.7 | 7.2 | 33.0 | 1.50 | 35.7 | 71.7 | 687 | 885 | 0.57 | -0.39 | 216 | 41.7 | 172.4 | 2.7 | 1.3 | 127.0 |
| Massara-1 | 4612 | 341 | 32.5 | 42.8 | 0.49 | 121.1 | 6.5 | 36.8 | 1.17 | 39.3 | 77.4 | 661 | 979 | 1.03 | -1.79 | 202 | 29.8 | 175.0 | 2.8 | 1.6 | 120.1 |
| Matt | 4518 | 334 | 32.9 | 43.9 | 0.48 | 121.5 | 6.0 | 34.3 | 1.27 | 34.4 | 73.0 | 712 | 1026 | 0.13 | -1.81 | 211 | 29.7 | 173.7 | 3.0 | 1.7 | 136.4 |
| Mavraani | 3740 | 331 | 27.6 | 45.7 | 0.35 | 130.8 | 4.8 | 29.7 | 1.54 | 48.9 | 124.2 | 813 | 1186 | 1.34 | 0.84 | 288 | 37.0 | 159.5 | 2.5 | 2.0 | 135.5 |
| Medora | 4195 | 310 | 31.7 | 40.4 | 0.40 | 127.3 | 5.2 | 33.0 | 1.24 | 42.5 | 103.7 | 744 | 993 | 1.52 | -0.71 | 245 | 38.0 | 174.8 | 2.6 | 1.5 | 129.9 |
| Mellaria | 4923 | 342 | 38.2 | 40.6 | 0.48 | 125.0 | 6.7 | 33.5 | 1.21 | 34.9 | 73.4 | 710 | 1091 | -0.15 | -0.86 | 219 | 43.6 | 158.3 | 2.8 | 2.3 | 146.2 |
| Meridiano | 4971 | 349 | 32.1 | 48.9 | 0.47 | 123.1 | 6.3 | 34.2 | 1.42 | 34.6 | 75.8 | 722 | 1137 | 1.69 | -0.22 | 205 | 36.6 | 172.1 | 3.0 | 2.3 | 141.6 |
| Merzaga | 4623 | 321 | 32.2 | 50.4 | 0.39 | 131.4 | 2.8 | 31.8 | 1.61 | 41.7 | 106.1 | 776 | 1321 | 2.98 | 0.71 | 327 | 21.7 | 168.9 | 2.3 | 3.0 | 154.9 |
| Mesaoria | 4583 | 331 | 33.7 | 44.1 | 0.46 | 124.6 | 6.0 | 35.2 | 1.27 | 40.2 | 78.2 | 748 | 1110 | 1.53 | -0.27 | 259 | 39.8 | 180.3 | 2.5 | 2.0 | 135.4 |
| Mexa | 4720 | 333 | 34.9 | 45.8 | 0.48 | 121.0 | 6.7 | 35.0 | 1.30 | 39.4 | 81.6 | 705 | 1092 | 1.20 | -0.84 | 197 | 33.8 | 163.8 | 3.1 | 2.5 | 139.3 |
| Mexidur | 4292 | 372 | 33.9 | 41.2 | 0.46 | 132.5 | 5.4 | 31.8 | 1.31 | 29.9 | 65.4 | 681 | 1143 | -2.89 | -1.12 | 244 | 33.9 | 144.5 | 2.5 | 2.9 | 126.0 |
| MG 26429 | 3445 | 352 | 28.8 | 35.1 | 0.39 | 128.0 | 3.2 | 31.4 | 1.12 | 33.4 | 76.9 | 736 | 927 | 1.93 | -0.14 | 229 | 22.7 | 159.8 | 3.0 | 1.2 | 124.3 |
| Milagro | 3968 | 337 | 35.2 | 39.3 | 0.40 | 130.1 | 6.5 | 30.7 | 1.30 | 49.9 | 112.7 | 727 | 1180 | -1.52 | -0.83 | 224 | 37.6 | 140.5 | 2.8 | 2.9 | 128.8 |
| Milazzo | 4146 | 324 | 29.5 | 47.2 | 0.35 | 135.3 | 2.9 | 30.5 | 1.57 | 45.0 | 101.2 | 738 | 1300 | -2.68 | 0.18 | 319 | 28.3 | 151.5 | 2.2 | 3.3 | 149.4 |
| Mindium | 4158 | 334 | 28.1 | 42.0 | 0.36 | 133.0 | 4.7 | 30.9 | 1.38 | 41.6 | 105.9 | 775 | 1092 | -0.94 | -0.69 | 297 | 38.6 | 158.3 | 2.4 | 2.0 | 133.9 |
| Mishriki | 3964 | 370 | 31.3 | 37.1 | 0.37 | 127.6 | 4.5 | 30.5 | 1.24 | 45.8 | 111.3 | 763 | 1175 | 3.34 | -0.62 | 281 | 31.2 | 155.1 | 2.5 | 2.4 | 134.9 |
| Modoc | 5241 | 317 | 39.4 | 41.7 | 0.46 | 125.7 | 6.2 | 33.6 | 1.23 | 32.8 | 78.3 | 680 | 1109 | 0.82 | 0.30 | 232 | 37.7 | 168.5 | 2.6 | 2.3 | 140.0 |
| Monroe | 4234 | 290 | 31.9 | 43.9 | 0.43 | 123.5 | 7.2 | 32.5 | 1.35 | 40.4 | 100.2 | 735 | 889 | 2.28 | -0.89 | 228 | 45.4 | 160.0 | 2.7 | 1.1 | 141.1 |
| Moosabil-1 | 4297 | 323 | 31.8 | 45.3 | 0.46 | 122.5 | 6.4 | 34.5 | 1.35 | 37.3 | 93.1 | 625 | 998 | -0.69 | -1.64 | 223 | 32.8 | 185.6 | 2.4 | 2.1 | 132.8 |
| Morocco | 3863 | 364 | 27.2 | 46.0 | 0.34 | 130.4 | 4.3 | 31.1 | 1.49 | 45.6 | 110.8 | 790 | 1352 | 3.57 | 0.21 | 307 | 30.1 | 170.9 | 2.4 | 3.5 | 155.8 |
| Moulsabil 2 | 4359 | 330 | 32.2 | 44.5 | 0.49 | 122.1 | 6.1 | 35.7 | 1.24 | 33.7 | 71.9 | 676 | 974 | 0.87 | -0.25 | 206 | 34.5 | 165.9 | 2.9 | 1.7 | 124.8 |
| Murlagost-1 | 4400 | 340 | 30.7 | 48.4 | 0.47 | 123.5 | 6.8 | 34.9 | 1.38 | 33.6 | 77.5 | 727 | 1078 | 1.12 | 0.26 | 222 | 41.5 | 176.4 | 2.8 | 1.7 | 140.1 |
| Narbada 215 | 4518 | 327 | 31.9 | 46.5 | 0.50 | 126.9 | 5.1 | 33.4 | 1.39 | 33.5 | 69.9 | 711 | 1032 | 0.94 | -1.02 | 263 | 33.7 | 170.7 | 2.4 | 1.7 | 144.7 |
| Nasr 99 | 4914 | 341 | 34.2 | 45.0 | 0.47 | 123.5 | 5.6 | 34.7 | 1.31 | 36.2 | 85.2 | 730 | 1148 | -0.26 | 0.14 | 273 | 38.8 | 182.9 | 2.5 | 2.3 | 166.5 |
| Ocotillo | 4389 | 335 | 30.5 | 44.4 | 0.43 | 121.8 | 6.3 | 34.7 | 1.29 | 30.1 | 77.1 | 692 | 1046 | 0.23 | -1.32 | 243 | 41.1 | 192.7 | 2.4 | 2.0 | 138.0 |
| Omgenil-3 | 4575 | 376 | 29.4 | 44.0 | 0.48 | 121.4 | 8.5 | 32.5 | 1.37 | 30.8 | 71.3 | 721 | 1018 | 1.99 | -0.41 | 221 | 52.8 | 162.1 | 2.6 | 1.8 | 133.3 |
| Omrabi 3 | 4460 | 339 | 39.6 | 46.5 | 0.47 | 120.9 | 6.4 | 36.9 | 1.25 | 36.9 | 77.6 | 678 | 1274 | 1.30 | -1.09 | 228 | 33.6 | 184.2 | 2.6 | 4.2 | 138.7 |
| Omrabi 5 | 4586 | 366 | 34.6 | 42.2 | 0.46 | 125.7 | 6.7 | 33.4 | 1.31 | 41.2 | 92.0 | 688 | 1187 | 1.22 | 0.45 | 230 | 40.9 | 180.7 | 2.6 | 2.2 | 134.4 |
| Omruf-2 | 4342 | 355 | 33.2 | 47.4 | 0.45 | 123.1 | 6.9 | 33.0 | 1.41 | 36.1 | 76.6 | 753 | 1190 | -0.21 | -1.34 | 233 | 50.6 | 189.0 | 2.8 | 2.6 | 143.1 |
| Oned Zenati | 4207 | 329 | 29.2 | 46.8 | 0.38 | 132.7 | 3.7 | 30.8 | 1.54 | 39.5 | 105.0 | 854 | 1186 | 0.97 | -0.64 | 305 | 30.9 | 165.5 | 2.6 | 2.2 | 149.1 |
| Orita | 4731 | 327 | 32.1 | 47.3 | 0.43 | 120.5 | 6.2 | 34.2 | 1.40 | 36.3 | 72.6 | 737 | 1132 | 0.54 | -1.48 | 225 | 32.5 | 182.1 | 2.9 | 2.4 | 138.0 |
| ORT-1 | 4371 | 324 | 29.9 | 51.7 | 0.45 | 123.2 | 7.3 | 33.4 | 1.54 | 35.2 | 76.4 | 716 | 1115 | 2.46 | 0.19 | 230 | 41.0 | 168.7 | 2.8 | 2.2 | 135.2 |
| Oscar | 4640 | 341 | 34.5 | 42.5 | 0.44 | 127.8 | 5.3 | 34.0 | 1.27 | 34.0 | 76.2 | 752 | 1149 | 0.02 | -0.69 | 273 | 32.6 | 170.9 | 2.5 | 2.3 | 131.7 |
| Ouaserl-1 | 4677 | 377 | 35.8 | 45.1 | 0.48 | 121.3 | 6.9 | 34.5 | 1.33 | 38.3 | 75.3 | 685 | 1264 | 0.25 | -2.06 | 208 | 34.7 | 167.4 | 2.9 | 3.3 | 133.7 |
| Ouasloukos-1 | 4536 | 348 | 37.3 | 45.0 | 0.48 | 123.8 | 6.2 | 34.8 | 1.29 | 36.4 | 77.9 | 664 | 1209 | 0.23 | -0.43 | 201 | 36.9 | 157.7 | 2.8 | 3.3 | 124.4 |
| Ouedezena | 4432 | 371 | 32.2 | 48.2 | 0.48 | 122.7 | 5.7 | 34.4 | 1.41 | 34.7 | 72.6 | 674 | 1195 | -1.85 | -2.11 | 208 | 29.0 | 163.1 | 2.9 | 2.9 | 139.4 |
| Pinet | 4019 | 326 | 30.6 | 45.3 | 0.36 | 132.5 | 4.9 | 30.0 | 1.53 | 46.0 | 120.9 | 789 | 1249 | 0.27 | -0.53 | 332 | 41.4 | 165.6 | 2.1 | 2.6 | 155.3 |
| Pingüino | 4418 | 307 | 37.0 | 49.4 | 0.48 | 123.8 | 6.0 | 35.5 | 1.37 | 32.8 | 72.9 | 622 | 1137 | 0.63 | -0.98 | 224 | 37.5 | 184.0 | 2.4 | 2.9 | 127.3 |
| Pisana cañihueca | 3816 | 331 | 25.7 | 43.5 | 0.33 | 136.3 | 4.2 | 31.5 | 1.40 | 51.9 | 131.6 | 733 | 1102 | -2.82 | -1.30 | 319 | 35.9 | 176.7 | 2.1 | 1.8 | 142.9 |
| Plata 16 | 4703 | 328 | 35.2 | 39.2 | 0.49 | 124.4 | 6.7 | 33.2 | 1.18 | 34.3 | 77.3 | 773 | 923 | -0.05 | -0.99 | 224 | 41.7 | 154.8 | 3.0 | 1.1 | 133.4 |
| Ponferrada | 4715 | 335 | 34.3 | 45.9 | 0.44 | 124.8 | 6.5 | 34.5 | 1.34 | 34.8 | 79.0 | 802 | 1179 | 1.85 | -0.28 | 226 | 40.9 | 180.4 | 3.1 | 2.0 | 133.0 |
| Porto 5 | 4710 | 331 | 41.9 | 38.9 | 0.46 | 124.2 | 7.1 | 33.2 | 1.16 | 34.6 | 70.6 | 724 | 1125 | 0.79 | -0.97 | 244 | 43.7 | 158.1 | 2.5 | 2.7 | 134.9 |
| Prospero | 4748 | 321 | 29.6 | 48.2 | 0.46 | 122.8 | 5.8 | 35.0 | 1.39 | 32.8 | 76.5 | 686 | 985 | 0.46 | -0.80 | 213 | 31.9 | 186.4 | 2.9 | 1.4 | 137.1 |
| Quabrach-1 | 4529 | 373 | 26.9 | 45.6 | 0.47 | 122.0 | 6.0 | 34.8 | 1.32 | 34.3 | 77.0 | 646 | 964 | 0.46 | -1.69 | 224 | 32.1 | 154.0 | 2.5 | 1.9 | 135.2 |
| Quilafen | 4727 | 331 | 32.3 | 45.1 | 0.47 | 125.6 | 5.6 | 34.8 | 1.31 | 34.1 | 74.4 | 708 | 1072 | -0.48 | -0.44 | 264 | 37.1 | 171.5 | 2.4 | 2.0 | 143.8 |
| Raj 1555 | 4482 | 332 | 28.9 | 46.7 | 0.46 | 120.6 | 6.6 | 34.9 | 1.35 | 39.9 | 79.7 | 697 | 1005 | 0.49 | -1.20 | 255 | 42.7 | 172.3 | 2.4 | 1.6 | 141.6 |
| Ramirez | 4624 | 338 | 35.1 | 41.3 | 0.47 | 126.6 | 6.0 | 32.8 | 1.25 | 32.7 | 77.9 | 741 | 1004 | 0.90 | 0.14 | 230 | 38.0 | 168.0 | 2.8 | 1.6 | 126.1 |
| Randur | 4340 | 331 | 39.0 | 36.4 | 0.41 | 131.2 | 4.2 | 31.9 | 1.16 | 33.2 | 91.4 | 718 | 1147 | 2.94 | 0.89 | 244 | 29.8 | 167.1 | 2.7 | 2.7 | 133.8 |
| Raposinho | 4165 | 308 | 35.1 | 49.7 | 0.40 | 131.3 | 3.6 | 33.4 | 1.51 | 56.3 | 125.8 | 751 | 1342 | 3.14 | 2.40 | 305 | 27.1 | 178.4 | 2.3 | 3.8 | 146.9 |
| Rapsani | 3769 | 344 | 33.9 | 38.1 | 0.36 | 133.4 | 4.7 | 29.3 | 1.33 | 47.5 | 119.7 | 746 | 1247 | -0.65 | -0.80 | 316 | 39.7 | 166.4 | 2.1 | 3.3 | 151.1 |
| Raspinegro | 4186 | 332 | 28.4 | 44.6 | 0.38 | 132.1 | 4.3 | 31.9 | 1.41 | 48.2 | 116.4 | 828 | 1110 | -0.74 | 2.00 | 283 | 32.6 | 161.6 | 2.7 | 1.9 | 146.2 |
| Raspinegro Canario | 4414 | 300 | 28.5 | 48.5 | 0.39 | 131.7 | 3.1 | 32.6 | 1.51 | 49.1 | 120.8 | 758 | 1048 | 2.82 | 0.64 | 269 | 24.9 | 179.2 | 2.6 | 1.6 | 147.6 |
| Raspinegro de Alcalá Guadaira | 4120 | 280 | 35.0 | 48.5 | 0.35 | 132.5 | 3.9 | 31.1 | 1.58 | 46.9 | 119.3 | 893 | 1246 | 1.58 | -0.73 | 308 | 33.0 | 166.9 | 2.6 | 2.1 | 139.5 |
| Razza 181 | 3878 | 321 | 29.9 | 46.3 | 0.34 | 132.4 | 3.9 | 30.8 | 1.52 | 48.7 | 123.4 | 856 | 1267 | 0.76 | -0.31 | 300 | 35.0 | 156.6 | 2.6 | 2.6 | 143.5 |
| Razza 208 | 4038 | 339 | 27.4 | 48.4 | 0.38 | 131.9 | 3.6 | 30.6 | 1.58 | 42.8 | 110.2 | 789 | 1180 | 0.84 | -1.03 | 305 | 31.0 | 173.4 | 2.4 | 2.2 | 151.0 |
| Razza 96 | 4090 | 311 | 28.3 | 50.8 | 0.37 | 131.9 | 3.6 | 31.1 | 1.65 | 46.7 | 107.4 | 909 | 1173 | 0.11 | -0.66 | 281 | 27.0 | 145.9 | 3.0 | 1.7 | 148.8 |
| Razzak 87 | 4694 | 324 | 32.1 | 43.0 | 0.47 | 122.7 | 5.5 | 33.7 | 1.29 | 28.8 | 69.6 | 732 | 1010 | 1.39 | -0.24 | 234 | 37.1 | 174.9 | 2.7 | 1.4 | 135.3 |
| Reading | 4159 | 349 | 29.4 | 45.3 | 0.39 | 131.9 | 4.6 | 30.7 | 1.50 | 42.5 | 104.3 | 751 | 1174 | 0.35 | 0.57 | 291 | 35.6 | 172.4 | 2.4 | 2.5 | 145.5 |
| Realforte | 4373 | 327 | 29.7 | 48.7 | 0.38 | 130.8 | 3.2 | 31.6 | 1.55 | 48.3 | 101.6 | 737 | 1236 | 2.95 | 0.96 | 328 | 26.2 | 183.2 | 2.1 | 2.4 | 158.0 |
| Recio de Almería | 4190 | 313 | 30.3 | 51.3 | 0.37 | 130.8 | 4.1 | 32.5 | 1.63 | 46.3 | 115.9 | 750 | 1310 | 2.57 | 0.70 | 304 | 32.7 | 182.3 | 2.2 | 3.1 | 146.1 |
| Recio de Cañete | 4198 | 295 | 31.3 | 49.4 | 0.35 | 130.9 | 4.7 | 31.5 | 1.59 | 50.4 | 118.1 | 734 | 1213 | 0.12 | -0.41 | 266 | 33.3 | 165.6 | 2.5 | 3.0 | 142.0 |
| Red Beard | 4339 | 312 | 23.2 | 48.8 | 0.36 | 132.1 | 3.5 | 31.0 | 1.60 | 37.5 | 97.6 | 871 | 944 | 0.80 | -1.37 | 289 | 30.3 | 167.6 | 2.7 | 0.6 | 140.8 |
| Reyati | 4227 | 358 | 30.3 | 46.1 | 0.38 | 131.4 | 5.9 | 29.8 | 1.56 | 48.8 | 119.9 | 826 | 1312 | -1.02 | -1.25 | 302 | 42.4 | 168.3 | 2.4 | 3.0 | 144.8 |
| Rubio de Belalcázar | 4185 | 365 | 35.1 | 41.9 | 0.37 | 137.2 | 3.5 | 27.6 | 1.54 | 43.6 | 125.6 | 764 | 1504 | -1.60 | -0.63 | 365 | 30.8 | 157.8 | 1.9 | 4.1 | 151.0 |
| Rubio de Montijo | 4048 | 384 | 30.1 | 40.1 | 0.35 | 131.3 | 5.0 | 29.9 | 1.38 | 50.9 | 118.7 | 841 | 1334 | 0.77 | -0.67 | 288 | 38.2 | 162.7 | 2.6 | 2.6 | 137.8 |
| Rubio enlargado d’Atlemteje | 3911 | 344 | 30.9 | 40.7 | 0.32 | 134.0 | 4.4 | 32.4 | 1.28 | 53.3 | 131.0 | 846 | 1374 | -0.53 | -1.74 | 313 | 37.5 | 158.8 | 2.5 | 3.1 | 141.9 |
| Sabil 1 | 4262 | 358 | 29.1 | 48.2 | 0.46 | 122.1 | 8.6 | 32.2 | 1.49 | 32.3 | 70.9 | 679 | 1108 | 1.18 | -1.10 | 242 | 50.2 | 163.2 | 2.3 | 2.2 | 131.1 |
| Safari | 4225 | 356 | 42.9 | 36.0 | 0.43 | 128.6 | 5.0 | 35.3 | 1.02 | 26.6 | 66.6 | 688 | 1261 | 3.12 | -0.28 | 218 | 31.9 | 173.2 | 2.8 | 3.7 | 132.4 |
| Saffi | 3705 | 333 | 25.8 | 48.3 | 0.36 | 129.2 | 4.3 | 31.5 | 1.55 | 39.9 | 115.3 | 718 | 1147 | 2.12 | -0.02 | 303 | 28.5 | 168.5 | 2.2 | 2.6 | 152.7 |
| Safra Jerash | 3939 | 349 | 31.0 | 44.9 | 0.40 | 124.2 | 6.4 | 33.9 | 1.32 | 49.4 | 100.6 | 727 | 1184 | 1.30 | 0.69 | 249 | 40.7 | 167.9 | 2.6 | 2.6 | 136.6 |
| Safra Maan | 3700 | 336 | 27.1 | 45.8 | 0.41 | 125.4 | 5.1 | 33.9 | 1.36 | 45.1 | 93.4 | 741 | 1006 | 2.88 | 0.17 | 241 | 34.5 | 158.1 | 2.7 | 1.6 | 135.6 |
| Sahel 77 | 4557 | 348 | 32.7 | 43.7 | 0.46 | 125.1 | 5.5 | 33.6 | 1.31 | 39.2 | 75.6 | 668 | 1117 | 1.92 | -0.30 | 243 | 35.8 | 178.6 | 2.4 | 2.5 | 140.2 |
| Saintly | 4388 | 351 | 42.2 | 39.9 | 0.48 | 120.7 | 6.5 | 35.1 | 1.16 | 37.9 | 77.8 | 729 | 1284 | 1.45 | -0.67 | 216 | 38.0 | 170.8 | 2.8 | 3.6 | 143.2 |
| Salti na Zinia | 3589 | 369 | 29.4 | 41.1 | 0.38 | 123.6 | 6.9 | 30.4 | 1.35 | 43.0 | 103.2 | 721 | 1153 | 1.54 | 0.44 | 256 | 40.1 | 161.1 | 2.5 | 3.0 | 131.5 |
| Santadur | 4645 | 340 | 47.9 | 36.7 | 0.47 | 123.8 | 6.4 | 33.0 | 1.11 | 33.0 | 77.7 | 653 | 1259 | 0.72 | 0.01 | 209 | 41.0 | 160.1 | 2.6 | 3.7 | 131.3 |
| Sarif | 4515 | 328 | 31.3 | 47.3 | 0.45 | 124.8 | 5.9 | 36.0 | 1.32 | 37.9 | 80.6 | 783 | 1084 | 1.46 | -0.04 | 222 | 38.3 | 188.5 | 3.0 | 1.5 | 129.8 |
| Sebah | 4545 | 351 | 35.3 | 46.2 | 0.48 | 123.5 | 4.6 | 35.0 | 1.34 | 32.8 | 72.6 | 590 | 1183 | 0.44 | -0.83 | 220 | 27.1 | 184.5 | 2.4 | 3.0 | 137.9 |
| Selinogradskaja | 4602 | 314 | 37.1 | 47.7 | 0.43 | 124.5 | 6.1 | 34.8 | 1.38 | 30.5 | 77.3 | 694 | 1254 | 1.83 | -1.35 | 243 | 42.2 | 190.1 | 2.4 | 3.3 | 134.8 |
| Semental | 3943 | 322 | 28.2 | 42.0 | 0.34 | 130.5 | 5.3 | 30.7 | 1.39 | 41.1 | 107.7 | 825 | 1080 | -1.94 | -0.42 | 296 | 41.2 | 178.5 | 2.4 | 1.7 | 146.3 |
| Semolero | 4779 | 321 | 34.4 | 47.6 | 0.47 | 124.2 | 6.3 | 33.2 | 1.42 | 32.2 | 75.1 | 686 | 1075 | 1.33 | -0.80 | 220 | 39.8 | 178.1 | 2.7 | 2.5 | 131.8 |
| Senadur | 5012 | 355 | 35.1 | 47.9 | 0.50 | 121.6 | 6.8 | 35.0 | 1.37 | 33.5 | 74.9 | 703 | 1192 | 0.30 | -1.16 | 225 | 33.7 | 175.2 | 2.7 | 2.5 | 141.7 |
| Senatore Capelli | 4195 | 302 | 33.6 | 50.8 | 0.36 | 130.2 | 4.6 | 32.3 | 1.60 | 52.3 | 119.6 | 897 | 1404 | 3.29 | 1.06 | 282 | 33.2 | 179.0 | 2.8 | 3.7 | 146.2 |
| Severo | 4446 | 365 | 42.9 | 37.1 | 0.48 | 125.2 | 6.3 | 33.7 | 1.11 | 32.1 | 74.1 | 689 | 1209 | 1.51 | -1.22 | 212 | 41.1 | 167.2 | 2.8 | 3.1 | 122.1 |
| Simeto | 4610 | 319 | 28.5 | 48.2 | 0.45 | 123.7 | 6.0 | 34.2 | 1.41 | 33.2 | 75.0 | 740 | 988 | 1.41 | -1.01 | 231 | 34.5 | 179.6 | 2.8 | 1.2 | 138.1 |
| Sinai No.8 | 3527 | 342 | 25.3 | 37.1 | 0.39 | 127.6 | 4.4 | 30.4 | 1.24 | 39.5 | 86.7 | 719 | 826 | 1.48 | -0.39 | 234 | 33.1 | 161.3 | 2.8 | 0.8 | 128.2 |
| Somat | 4543 | 336 | 34.8 | 51.2 | 0.45 | 124.7 | 5.5 | 34.3 | 1.48 | 31.1 | 72.9 | 770 | 1296 | 2.57 | 0.50 | 223 | 34.3 | 194.2 | 3.0 | 3.0 | 139.2 |
| Souri | 3880 | 352 | 30.4 | 44.2 | 0.38 | 128.9 | 4.0 | 33.1 | 1.35 | 47.8 | 113.8 | 705 | 1233 | 3.05 | -0.04 | 281 | 26.6 | 165.6 | 2.3 | 3.0 | 138.0 |
| Stojocri-2 | 4309 | 344 | 29.7 | 47.1 | 0.44 | 121.8 | 6.9 | 35.1 | 1.35 | 36.1 | 84.2 | 684 | 1088 | 0.34 | -0.50 | 220 | 36.3 | 172.3 | 2.7 | 2.1 | 138.5 |
| Stojocri-3 | 4666 | 315 | 33.6 | 46.0 | 0.47 | 121.5 | 6.5 | 35.3 | 1.31 | 35.6 | 85.0 | 680 | 1014 | 0.69 | -2.04 | 234 | 36.5 | 165.5 | 2.5 | 2.1 | 133.8 |
| Stork | 4423 | 312 | 30.8 | 47.0 | 0.48 | 121.8 | 6.3 | 35.0 | 1.34 | 37.3 | 84.8 | 753 | 927 | 0.70 | 0.01 | 215 | 38.9 | 177.5 | 3.0 | 0.9 | 130.8 |
| Strongfield | 4145 | 353 | 34.4 | 39.5 | 0.41 | 132.9 | 6.1 | 29.5 | 1.35 | 35.9 | 88.1 | 708 | 1163 | -0.39 | -2.35 | 260 | 35.6 | 154.9 | 2.5 | 3.0 | 136.6 |
| Sula | 4466 | 330 | 39.9 | 38.7 | 0.48 | 123.9 | 6.2 | 32.9 | 1.18 | 33.0 | 66.3 | 686 | 1046 | 0.47 | -1.10 | 245 | 39.6 | 148.0 | 2.4 | 2.0 | 143.2 |
| Svevo | 4608 | 350 | 29.6 | 46.3 | 0.47 | 121.4 | 6.1 | 34.4 | 1.34 | 36.0 | 75.9 | 652 | 1005 | -1.72 | -1.77 | 218 | 33.7 | 175.9 | 2.5 | 1.7 | 136.5 |
| Tamaroi | 4236 | 332 | 35.6 | 36.9 | 0.42 | 124.8 | 6.4 | 33.5 | 1.12 | 37.3 | 80.2 | 761 | 1067 | 1.33 | -0.85 | 239 | 39.3 | 171.7 | 2.8 | 1.7 | 144.0 |
| Taranto | 4573 | 327 | 31.6 | 50.7 | 0.46 | 123.1 | 7.1 | 32.9 | 1.53 | 28.9 | 70.5 | 696 | 1121 | -0.63 | -2.24 | 209 | 41.9 | 160.5 | 2.9 | 2.5 | 145.5 |
| Tassaout | 4964 | 322 | 36.8 | 46.1 | 0.46 | 126.3 | 6.4 | 32.4 | 1.43 | 35.8 | 78.0 | 720 | 1169 | 2.49 | 0.19 | 261 | 42.8 | 169.9 | 2.4 | 2.7 | 131.6 |
| Tchirpan | 3854 | 302 | 27.5 | 39.7 | 0.40 | 132.6 | 4.3 | 31.3 | 1.30 | 36.7 | 77.4 | 755 | 822 | -1.63 | -0.96 | 270 | 32.4 | 180.5 | 2.5 | 0.7 | 114.7 |
| Tejón | 3979 | 348 | 33.9 | 44.6 | 0.45 | 122.6 | 7.4 | 34.5 | 1.30 | 33.5 | 77.0 | 700 | 1146 | 2.12 | 0.79 | 199 | 44.6 | 163.6 | 2.9 | 3.0 | 130.5 |
| Terbol97-3 | 4554 | 330 | 35.0 | 45.0 | 0.46 | 122.7 | 6.8 | 34.8 | 1.30 | 36.9 | 81.6 | 694 | 1117 | 1.46 | -0.44 | 214 | 41.9 | 163.7 | 2.7 | 2.6 | 139.3 |
| Tetradur | 4233 | 351 | 33.7 | 39.2 | 0.44 | 129.8 | 4.2 | 33.2 | 1.18 | 33.8 | 71.5 | 685 | 1074 | 1.37 | -1.79 | 211 | 28.8 | 160.1 | 2.9 | 2.1 | 125.3 |
| Tounse | 4266 | 308 | 28.8 | 44.2 | 0.36 | 135.6 | 3.2 | 30.7 | 1.49 | 50.2 | 114.4 | 767 | 1039 | -1.08 | -1.51 | 284 | 26.8 | 166.0 | 2.5 | 1.6 | 146.9 |
| Tremes rijo | 3867 | 387 | 26.0 | 47.3 | 0.34 | 132.0 | 3.3 | 32.3 | 1.49 | 44.1 | 106.1 | 741 | 1379 | 2.13 | -0.29 | 281 | 24.6 | 162.9 | 2.4 | 3.8 | 139.7 |
| Trigo Glutinoso | 3806 | 356 | 37.7 | 39.0 | 0.39 | 132.8 | 4.8 | 30.3 | 1.32 | 54.8 | 116.9 | 750 | 1328 | -0.63 | -0.30 | 289 | 34.1 | 173.2 | 2.3 | 2.9 | 134.1 |
| Trinakria | 4116 | 339 | 31.2 | 48.1 | 0.43 | 123.7 | 6.1 | 33.6 | 1.45 | 48.3 | 92.6 | 679 | 1182 | 1.85 | 0.66 | 221 | 35.1 | 165.3 | 2.7 | 3.2 | 136.5 |
| Tripshiro | 3898 | 351 | 25.2 | 36.4 | 0.36 | 125.2 | 5.9 | 31.1 | 1.20 | 36.8 | 89.2 | 691 | 896 | 1.68 | -0.44 | 234 | 37.2 | 159.6 | 2.6 | 1.8 | 143.2 |
| Ucaro 1 | 4382 | 343 | 38.9 | 41.1 | 0.45 | 122.9 | 7.4 | 33.8 | 1.23 | 33.2 | 78.3 | 732 | 1237 | 1.59 | -0.88 | 220 | 44.0 | 167.9 | 2.8 | 3.1 | 138.7 |
| Valgera | 4552 | 331 | 36.0 | 47.7 | 0.46 | 123.9 | 6.2 | 34.9 | 1.37 | 30.2 | 75.8 | 710 | 1229 | 1.51 | -0.41 | 249 | 40.0 | 185.0 | 2.5 | 3.1 | 130.5 |
| Valira | 5104 | 365 | 36.4 | 46.4 | 0.47 | 124.1 | 7.6 | 33.3 | 1.39 | 38.8 | 82.4 | 809 | 1308 | 0.79 | -0.16 | 235 | 54.1 | 180.5 | 2.9 | 2.4 | 136.9 |
| Verdial | 4203 | 339 | 39.1 | 41.1 | 0.39 | 134.2 | 4.5 | 30.7 | 1.38 | 47.6 | 114.7 | 817 | 1410 | -0.30 | -0.24 | 301 | 33.1 | 156.8 | 2.5 | 3.3 | 137.4 |
| Vic | 4163 | 358 | 33.0 | 41.0 | 0.40 | 128.3 | 5.6 | 31.4 | 1.33 | 39.9 | 102.3 | 711 | 1195 | 3.05 | 0.82 | 232 | 40.8 | 170.4 | 2.7 | 3.0 | 132.9 |
| VII/13-X11 | 4135 | 360 | 28.7 | 45.8 | 0.38 | 132.7 | 4.9 | 29.8 | 1.57 | 51.7 | 118.3 | 802 | 1262 | -0.71 | -3.09 | 280 | 36.1 | 159.4 | 2.6 | 2.6 | 144.7 |
| Vitron | 4506 | 311 | 34.1 | 46.3 | 0.50 | 123.3 | 7.0 | 33.8 | 1.37 | 33.4 | 70.7 | 683 | 974 | 0.93 | 0.86 | 214 | 43.6 | 172.6 | 2.7 | 1.8 | 138.7 |
| Vitronero | 4653 | 339 | 29.7 | 43.7 | 0.48 | 122.8 | 7.6 | 32.0 | 1.36 | 28.3 | 76.4 | 712 | 905 | 2.18 | 0.22 | 227 | 47.9 | 168.3 | 2.6 | 1.2 | 139.4 |
| Wadalmez-1 | 3895 | 356 | 28.2 | 44.4 | 0.43 | 123.4 | 5.8 | 33.3 | 1.35 | 34.8 | 78.4 | 728 | 1001 | 0.57 | 0.10 | 211 | 35.9 | 164.6 | 3.0 | 1.5 | 128.7 |
| Wadhanak 85 | 4607 | 341 | 35.9 | 43.6 | 0.47 | 123.7 | 5.0 | 34.3 | 1.28 | 32.1 | 83.0 | 753 | 1175 | 0.15 | -0.80 | 209 | 36.2 | 169.9 | 3.0 | 2.5 | 130.3 |
| Waha | 4798 | 360 | 37.0 | 42.5 | 0.48 | 123.3 | 7.5 | 33.7 | 1.26 | 34.1 | 74.5 | 597 | 1155 | 0.21 | -0.07 | 237 | 47.1 | 172.9 | 2.1 | 3.4 | 133.5 |
| Wakooma | 3396 | 356 | 32.3 | 39.8 | 0.39 | 129.9 | 5.4 | 31.2 | 1.30 | 42.1 | 96.7 | 727 | 1187 | 0.45 | -1.10 | 239 | 32.4 | 162.9 | 2.7 | 2.4 | 123.7 |
| Ward | 4300 | 368 | 30.5 | 39.7 | 0.44 | 126.7 | 6.1 | 31.0 | 1.31 | 37.0 | 100.9 | 644 | 1018 | 1.16 | -1.60 | 204 | 36.1 | 153.7 | 2.7 | 2.6 | 129.0 |
| Waskana | 4373 | 376 | 34.8 | 41.1 | 0.38 | 130.6 | 5.5 | 31.3 | 1.32 | 45.4 | 112.8 | 774 | 1428 | 1.68 | -0.62 | 293 | 43.5 | 168.5 | 2.3 | 3.7 | 141.1 |
| West Bred Laker | 4742 | 310 | 32.0 | 44.8 | 0.41 | 130.0 | 3.9 | 33.1 | 1.35 | 35.9 | 87.0 | 764 | 1058 | 2.76 | 0.84 | 297 | 29.9 | 177.9 | 2.4 | 1.5 | 148.7 |
| West Bred Turbo | 4738 | 332 | 34.9 | 47.3 | 0.49 | 122.6 | 7.7 | 33.0 | 1.44 | 30.7 | 72.8 | 685 | 1102 | 0.57 | -0.86 | 216 | 49.8 | 185.8 | 2.6 | 2.2 | 133.3 |
| WH 896 | 4794 | 336 | 32.3 | 43.3 | 0.45 | 124.6 | 7.5 | 30.6 | 1.42 | 30.6 | 72.7 | 720 | 1081 | 0.62 | -0.62 | 259 | 43.1 | 165.2 | 2.4 | 2.1 | 137.5 |
| Yasmine | 5326 | 339 | 34.1 | 46.3 | 0.46 | 125.0 | 7.0 | 32.4 | 1.42 | 31.8 | 79.5 | 686 | 1140 | 2.57 | -0.53 | 274 | 53.6 | 177.2 | 2.1 | 2.4 | 149.5 |
| Zagorka | 3990 | 333 | 28.7 | 40.6 | 0.36 | 133.0 | 4.5 | 29.3 | 1.37 | 36.5 | 83.6 | 772 | 1073 | -0.33 | -1.49 | 300 | 37.9 | 141.2 | 2.4 | 2.0 | 149.8 |
| Zeina 1 | 4176 | 327 | 28.4 | 43.7 | 0.46 | 124.8 | 6.5 | 33.3 | 1.33 | 32.7 | 77.4 | 697 | 870 | 1.39 | -0.12 | 217 | 37.7 | 167.4 | 2.7 | 1.2 | 126.5 |
| Zoco Yebel Hebil | 4214 | 417 | 28.8 | 40.6 | 0.34 | 133.3 | 3.2 | 31.8 | 1.31 | 43.7 | 99.5 | 693 | 1443 | 1.29 | -0.33 | 313 | 29.1 | 167.8 | 2.0 | 4.2 | 136.5 |
| Zoghbiyeh Safra | 3910 | 357 | 29.4 | 45.1 | 0.41 | 124.6 | 6.1 | 33.5 | 1.36 | 46.4 | 93.2 | 728 | 1141 | 2.93 | -0.69 | 211 | 38.7 | 170.8 | 2.9 | 2.6 | 134.4 |
| Zugbieh Sutra | 4210 | 343 | 31.5 | 43.7 | 0.42 | 123.1 | 6.8 | 33.8 | 1.30 | 50.8 | 93.7 | 674 | 1097 | 0.90 | 0.48 | 222 | 39.8 | 170.6 | 2.7 | 2.6 | 139.6 |

**Supplementary TABLE S3 ǀ** GWAS results.

| **Year** | **Trait** | **Marker** | **Chromosome** | **Position (cM)** | **PVE** | **-log*P*** |
| --- | --- | --- | --- | --- | --- | --- |
| 2014 | CTD_Mi_ | 4008064_PAV | 1A | 6.16 | 0.04 | 3.80 |
| 2015 | PL | 1020433_SNP | 1A | 7.22 | 0.07 | 3.75 |
| 2013 | NS | 3949529_PAV | 1A | 7.22 | 0.04 | 3.62 |
| 2013 | DM_M_ | 3949529_PAV | 1A | 7.22 | 0.03 | 3.33 |
| 2013 | RUE_AM_ | 3949529_PAV | 1A | 7.22 | 0.03 | 3.29 |
| 2013 | D_AM_ | 4406014_PAV | 1A | 7.22 | 0.03 | 3.80 |
| 2013 | RUE_AM_ | 1128039_SNP | 1A | 7.59 | 0.06 | 3.73 |
| 2013 | NS | 1128039_SNP | 1A | 7.59 | 0.06 | 3.45 |
| 2013 | DM_M_ | 1128039_SNP | 1A | 7.59 | 0.05 | 3.26 |
| 2014 | D_AM_ | 1384324_PAV | 1A | 8.4 | 0.04 | 3.58 |
| 2013 | cPAR_EH_ | 1112159_PAV | 1A | 16.09 | 0.03 | 3.31 |
| 2015 | RUE_AM_ | 3534358_PAV | 1A | 25.31 | 0.04 | 3.37 |
| 2014 | D_AM_ | 3573015_PAV | 1A | 25.31 | 0.04 | 3.38 |
| 2015 | RUE_AM_ | 3573015_PAV | 1A | 25.31 | 0.04 | 3.22 |
| 2014 | PH | 2277788_SNP | 1A | 41.23 | 0.04 | 3.33 |
| 2013 | NGS | 1133526_PAV | 1A | 58.72 | 0.04 | 3.74 |
| 2013 | CTD_Mi_ | 2277328_SNP | 1A | 62.16 | 0.05 | 3.70 |
| 2014 | RUE_AM_ | 4398298_PAV | 1A | 62.93 | 0.03 | 3.07 |
| 2014 | DM_M_ | 4398298_PAV | 1A | 62.93 | 0.03 | 3.00 |
| 2015 | CTD_Mi_ | 1101419_PAV | 1A | 64.83 | 0.05 | 4.21 |
| 2014 | CTD_Mi_ | 994367_SNP | 1A | 67.69 | 0.04 | 3.45 |
| 2013 | CTD_Mi_ | 994367_SNP | 1A | 67.69 | 0.04 | 3.23 |
| 2015 | CTD_Mi_ | 4410544_PAV | 1A | 79.27 | 0.03 | 3.22 |
| 2015 | DM_A_ | 4410341_PAV | 1A | 80.23 | 0.04 | 3.61 |
| 2015 | DM_M_ | 1121826_PAV | 1A | 80.82 | 0.03 | 3.17 |
| 2015 | PH | 1051568_SNP | 1A | 80.93 | 0.04 | 3.29 |
| 2015 | PH | 1090724_PAV | 1A | 81.32 | 0.04 | 3.51 |
| 2014 | CTD_Mi_ | 1094246_SNP | 1A | 81.63 | 0.05 | 4.00 |
| 2015 | CTD_Mi_ | 1082675_SNP | 1A | 82.87 | 0.04 | 3.07 |
| 2015 | DM_A_ | 3951863_PAV | 1A | 83.23 | 0.04 | 3.57 |
| 2013 | GFR | 1265462_PAV | 1A | 85.46 | 0.03 | 3.29 |
| 2013 | CTD_Mi_ | 3022070_PAV | 1A | 86.78 | 0.04 | 3.34 |
| 2014 | PL | 3022070_PAV | 1A | 86.78 | 0.03 | 3.27 |
| 2015 | D_EH_ | 1724079_PAV | 1A | 91.11 | 0.00 | 3.63 |
| 2015 | DM_M_ | 3222545_SNP | 1A | 91.11 | 0.05 | 3.67 |
| 2015 | PH | 3939206_PAV | 1A | 93.41 | 0.03 | 3.44 |
| 2014 | PH | 2262966_SNP | 1A | 99.62 | 0.06 | 3.87 |
| 2013 | D_AM_ | 1063183_PAV | 1A | 102.19 | 0.02 | 3.14 |
| 2014 | D_EH_ | 3064923_SNP | 1A | 103.83 | 0.01 | 3.74 |
| 2015 | D_EH_ | 3064923_SNP | 1A | 103.83 | 0.00 | 3.26 |
| 2015 | PH | 1137101_PAV | 1A | 108.61 | 0.04 | 3.79 |
| 2013 | GFR | 2303774_PAV | 1A | 117.13 | 0.03 | 3.10 |
| 2013 | D_EH_ | 4397580_PAV | 1A | 117.17 | 0.01 | 3.12 |
| 2015 | D_EH_ | 1002571_SNP | 1A | 117.54 | 0.01 | 7.13 |
| 2014 | D_EH_ | 1002571_SNP | 1A | 117.54 | 0.01 | 4.79 |
| 2013 | cPAR_EH_ | 1002571_SNP | 1A | 117.54 | 0.04 | 4.04 |
| 2014 | NGS | 4004173_PAV | 1A | 117.7 | 0.03 | 3.05 |
| 2014 | D_EH_ | 1210578_SNP | 1A | 117.77 | 0.01 | 4.24 |
| 2014 | CTD_Mi_ | 2362198_SNP | 1A | 118.26 | 0.04 | 3.00 |
| 2014 | CTD_Mi_ | 1090978_PAV | 1A | 131.88 | 0.04 | 3.42 |
| 2013 | D_HA_ | 1090978_PAV | 1A | 131.88 | 0.03 | 3.11 |
| 2014 | PH | 1028725_SNP | 1A | 133.6 | 0.04 | 3.53 |
| 2014 | W | 1104662_SNP | 1A | 150.35 | 0.06 | 3.28 |
| 2013 | D_HA_ | 1085538_PAV | 1A | 152.2 | 0.02 | 3.02 |
| 2014 | D_HA_ | 4409218_PAV | 1A | 160.43 | 0.03 | 3.21 |
| 2014 | CTD_Mi_ | 2306419_PAV | 1A | 160.56 | 0.05 | 4.60 |
| 2014 | CTD_Mi_ | 992431_SNP | 1A | 160.89 | 0.04 | 3.13 |
| 2014 | CTD_Mi_ | 1217099_SNP | 1A | 166.52 | 0.04 | 3.04 |
| 2014 | PH | 1123074_SNP | 1A | 167.06 | 0.06 | 4.81 |
| 2014 | CTD_Mi_ | 1036806_SNP | 1A | 171.85 | 0.05 | 3.68 |
| 2014 | CTD_Mi_ | 1094451_SNP | 1A | 173.55 | 0.06 | 4.71 |
| 2014 | CTD_Mi_ | 1030952_SNP | 1A | 174.33 | 0.04 | 3.06 |
| 2013 | RUE_AM_ | 3028636_PAV | 1A | 180.11 | 0.03 | 3.13 |
| 2013 | NGS | 3028636_PAV | 1A | 180.11 | 0.03 | 3.02 |
| 2013 | D_AM_ | 1124216_PAV | 1A | 187.97 | 0.03 | 3.09 |
| 2014 | Yield | 4406210_PAV | 1A | 191.82 | 0.03 | 3.44 |
| 2014 | PH | 4406210_PAV | 1A | 191.82 | 0.03 | 3.28 |
| 2013 | PH | 4406210_PAV | 1A | 191.82 | 0.03 | 3.26 |
| 2015 | PL | 1126137_PAV | 1A | 224.36 | 0.03 | 3.06 |
| 2015 | GFR | 1008576_SNP | 1A | 232.39 | 0.04 | 3.26 |
| 2013 | NGS | 1377838_PAV | 1A | 234.44 | 0.04 | 4.14 |
| 2015 | PL | 4404731_PAV | 1A | 236.8 | 0.04 | 3.68 |
| 2015 | PL | 4405166_PAV | 1A | 236.8 | 0.04 | 3.68 |
| 2015 | PL | 4408684_PAV | 1A | 236.8 | 0.04 | 3.68 |
| 2015 | PL | 3028294_PAV | 1A | 237.22 | 0.03 | 3.14 |
| 2015 | PL | 1019773_PAV | 1A | 237.88 | 0.04 | 4.03 |
| 2015 | PL | 3384831_PAV | 1A | 238.01 | 0.03 | 3.33 |
| 2015 | PL | 1129758_PAV | 1A | 238.06 | 0.03 | 3.24 |
| 2015 | PL | 3026199_PAV | 1A | 238.4 | 0.04 | 3.57 |
| 2015 | PL | 1220050_PAV | 1A | 240.07 | 0.04 | 3.83 |
| 2013 | cPAR_EH_ | 2301074_PAV | 1A | 245.81 | 0.02 | 3.22 |
| 2013 | cPAR_EH_ | 1086532_PAV | 1A | 250.14 | 0.02 | 3.05 |
| 2014 | DM_M_ | 1104221_PAV | 1A | 250.14 | 0.03 | 3.04 |
| 2015 | GFR | 990592_SNP | 1A | 250.14 | 0.04 | 3.06 |
| 2015 | GFR | 3955480_SNP | 1A | 251.18 | 0.05 | 3.84 |
| 2013 | cPAR_EH_ | 4393842_PAV | 1A | 251.28 | 0.03 | 3.41 |
| 2015 | PH | 1119717_PAV | 1B | 3.58 | 0.03 | 3.38 |
| 2015 | PH | 1089620_PAV | 1B | 4.19 | 0.04 | 3.90 |
| 2015 | PL | 1089620_PAV | 1B | 4.19 | 0.03 | 3.27 |
| 2014 | CTD_Mi_ | 1163555_PAV | 1B | 4.19 | 0.03 | 3.22 |
| 2015 | PH | 1120343_PAV | 1B | 4.39 | 0.03 | 3.26 |
| 2015 | PH | 1130108_PAV | 1B | 4.46 | 0.03 | 3.05 |
| 2015 | GFR | 997207_SNP | 1B | 4.71 | 0.04 | 3.03 |
| 2015 | PH | 2303647_PAV | 1B | 6.26 | 0.04 | 3.84 |
| 2014 | CTD_Mi_ | 3952045_PAV | 1B | 6.84 | 0.03 | 3.27 |
| 2015 | PH | 1259845_PAV | 1B | 7.16 | 0.03 | 3.29 |
| 2015 | PH | 1279427_PAV | 1B | 7.79 | 0.04 | 3.71 |
| 2015 | PH | 3022546_PAV | 1B | 7.79 | 0.03 | 3.01 |
| 2014 | GFR | 1229677_PAV | 1B | 8.1 | 0.03 | 3.02 |
| 2015 | PH | 2276990_PAV | 1B | 8.3 | 0.03 | 3.10 |
| 2015 | PH | 1234475_PAV | 1B | 8.41 | 0.03 | 3.42 |
| 2014 | GFR | 1242215_PAV | 1B | 8.41 | 0.04 | 3.72 |
| 2015 | PH | 2277173_PAV | 1B | 8.41 | 0.04 | 4.06 |
| 2015 | PH | 1279388_PAV | 1B | 8.95 | 0.06 | 5.23 |
| 2015 | PH | 1267298_PAV | 1B | 9 | 0.03 | 3.41 |
| 2015 | PH | 1279324_PAV | 1B | 9 | 0.05 | 5.14 |
| 2015 | PL | 1279324_PAV | 1B | 9 | 0.03 | 3.32 |
| 2015 | PH | 1018209_PAV | 1B | 9.3 | 0.03 | 3.23 |
| 2015 | PH | 1103430_PAV | 1B | 9.3 | 0.04 | 3.84 |
| 2014 | HI | 1124281_PAV | 1B | 9.3 | 0.04 | 3.90 |
| 2015 | PH | 1238673_PAV | 1B | 9.3 | 0.03 | 3.54 |
| 2015 | PH | 1267412_PAV | 1B | 9.3 | 0.05 | 4.24 |
| 2015 | PH | 1267670_PAV | 1B | 9.3 | 0.04 | 4.04 |
| 2014 | GFR | 1268362_PAV | 1B | 9.3 | 0.03 | 3.12 |
| 2015 | PH | 1274218_PAV | 1B | 9.3 | 0.03 | 3.25 |
| 2015 | PH | 1279790_PAV | 1B | 9.3 | 0.03 | 3.61 |
| 2015 | PH | 1062346_PAV | 1B | 9.59 | 0.04 | 3.87 |
| 2014 | PH | 1062346_PAV | 1B | 9.59 | 0.04 | 3.66 |
| 2014 | CTD_Mi_ | 1120149_PAV | 1B | 9.59 | 0.03 | 3.34 |
| 2014 | CTD_Mi_ | 2276956_SNP | 1B | 15.67 | 0.05 | 4.03 |
| 2015 | PH | 1278292_PAV | 1B | 17.79 | 0.04 | 3.82 |
| 2014 | D_EH_ | 2275986_PAV | 1B | 20.41 | 0.00 | 3.04 |
| 2015 | PH | 1012381_PAV | 1B | 27.22 | 0.05 | 4.69 |
| 2014 | RUE_EA_ | 1151931_PAV | 1B | 32.69 | 0.03 | 3.03 |
| 2015 | HI | 4405057_PAV | 1B | 37.69 | 0.03 | 3.21 |
| 2015 | HI | 4410652_PAV | 1B | 37.69 | 0.03 | 3.21 |
| 2014 | CTD_Mi_ | 1081768_SNP | 1B | 42.86 | 0.05 | 4.05 |
| 2014 | NS | 1121425_PAV | 1B | 44.77 | 0.04 | 3.97 |
| 2015 | PH | 4261156_PAV | 1B | 48.8 | 0.03 | 3.13 |
| 2014 | CTD_Mi_ | 1163852_SNP | 1B | 49.2 | 0.05 | 3.71 |
| 2015 | Yield | 1695856_SNP | 1B | 51.29 | 0.04 | 3.63 |
| 2014 | PH | 2300797_PAV | 1B | 51.29 | 0.03 | 3.09 |
| 2015 | PH | 3942840_PAV | 1B | 51.29 | 0.03 | 3.35 |
| 2015 | NGS | 4261691_PAV | 1B | 51.29 | 0.03 | 3.03 |
| 2014 | NGS | 4394833_PAV | 1B | 51.29 | 0.05 | 4.72 |
| 2015 | PH | 4396510_PAV | 1B | 51.29 | 0.03 | 3.04 |
| 2015 | cPAR_AM_ | 4409100_PAV | 1B | 51.29 | 0.03 | 3.24 |
| 2015 | D_EH_ | 4410695_PAV | 1B | 51.29 | 0.00 | 3.92 |
| 2015 | cPAR_AM_ | 4411408_PAV | 1B | 51.29 | 0.03 | 3.24 |
| 2013 | CTD_A_ | 997598_SNP | 1B | 51.58 | 0.05 | 3.50 |
| 2015 | HI | 1114624_SNP | 1B | 54.02 | 0.04 | 3.59 |
| 2015 | cPAR_AM_ | 1104653_PAV | 1B | 63.91 | 0.03 | 3.04 |
| 2015 | cPAR_AM_ | 2279800_PAV | 1B | 63.91 | 0.03 | 3.23 |
| 2015 | cPAR_AM_ | 3946314_PAV | 1B | 63.91 | 0.03 | 3.17 |
| 2015 | cPAR_AM_ | 4409866_PAV | 1B | 63.91 | 0.03 | 3.22 |
| 2014 | CTD_Mi_ | 1119193_SNP | 1B | 73.14 | 0.04 | 3.51 |
| 2015 | PH | 3949371_PAV | 1B | 76.4 | 0.03 | 3.01 |
| 2014 | NGS | 1089943_PAV | 1B | 80.42 | 0.04 | 3.76 |
| 2015 | CTD_Mi_ | 3020845_PAV | 1B | 81.58 | 0.03 | 3.22 |
| 2013 | D_EH_ | 4007994_PAV | 1B | 82.16 | 0.01 | 3.35 |
| 2015 | GA_90d_ | 1194221_SNP | 1B | 82.75 | 0.04 | 3.22 |
| 2014 | D_EH_ | 4410576_PAV | 1B | 83.3 | 0.00 | 3.42 |
| 2013 | D_HA_ | 2318542_PAV | 1B | 85.8 | 0.03 | 3.42 |
| 2013 | RUE_AM_ | 2277673_PAV | 1B | 86.38 | 0.03 | 3.09 |
| 2014 | NGS | 4409348_PAV | 1B | 90.31 | 0.04 | 3.74 |
| 2014 | NGS | 1091443_PAV | 1B | 90.87 | 0.04 | 3.83 |
| 2013 | PL | 3946834_PAV | 1B | 94.95 | 0.03 | 3.50 |
| 2015 | HI | 1307796_SNP | 1B | 96.05 | 0.03 | 3.06 |
| 2013 | CTD_A_ | 1201029_SNP | 1B | 98.59 | 0.04 | 3.20 |
| 2014 | RUE_EA_ | 4412117_PAV | 1B | 99.36 | 0.03 | 3.50 |
| 2015 | D_EH_ | 1090571_SNP | 1B | 100.89 | 0.00 | 3.14 |
| 2013 | D_HA_ | 3948018_SNP | 1B | 102.79 | 0.03 | 3.54 |
| 2014 | Yield | 4410528_PAV | 1B | 102.79 | 0.03 | 3.11 |
| 2014 | RUE_EA_ | 4411452_PAV | 1B | 103.29 | 0.03 | 3.50 |
| 2015 | cPAR_AM_ | 3938778_PAV | 1B | 109.71 | 0.03 | 3.15 |
| 2014 | RUE_EA_ | 3957258_PAV | 1B | 111.63 | 0.03 | 3.52 |
| 2015 | cPAR_AM_ | 3934520_PAV | 1B | 114.18 | 0.03 | 3.15 |
| 2015 | cPAR_AM_ | 3946072_PAV | 1B | 114.18 | 0.03 | 3.15 |
| 2015 | cPAR_AM_ | 3948254_PAV | 1B | 114.18 | 0.03 | 3.15 |
| 2014 | HI | 4008958_PAV | 1B | 114.18 | 0.03 | 3.18 |
| 2015 | CTD_A_ | 1215945_SNP | 1B | 116.58 | 0.04 | 3.46 |
| 2014 | PH | 1215945_SNP | 1B | 116.58 | 0.04 | 3.08 |
| 2015 | HI | 2266225_SNP | 1B | 120.02 | 0.05 | 4.30 |
| 2013 | PH | 3570025_SNP | 1B | 123.55 | 0.05 | 3.32 |
| 2015 | cPAR_AM_ | 1164132_SNP | 1B | 123.64 | 0.04 | 3.18 |
| 2015 | W | 1213417_SNP | 1B | 123.64 | 0.05 | 3.46 |
| 2015 | GFR | 1213417_SNP | 1B | 123.64 | 0.04 | 3.38 |
| 2015 | RUE_EA_ | 983716_PAV | 1B | 133.86 | 0.04 | 3.66 |
| 2014 | CTD_Mi_ | 995057_SNP | 1B | 133.87 | 0.05 | 3.87 |
| 2013 | GFR | 995057_SNP | 1B | 133.87 | 0.04 | 3.00 |
| 2014 | CTD_Mi_ | 1218788_SNP | 1B | 142.15 | 0.05 | 3.48 |
| 2013 | DM_A_ | 1668347_SNP | 1B | 142.15 | 0.05 | 3.49 |
| 2013 | GA_90d_ | 1212317_PAV | 1B | 142.57 | 0.04 | 3.93 |
| 2013 | GA_90d_ | 2277204_PAV | 1B | 142.57 | 0.05 | 3.99 |
| 2015 | cPAR_AM_ | 3026450_PAV | 1B | 147.3 | 0.04 | 4.20 |
| 2013 | GA_90d_ | 2323501_PAV | 1B | 148.22 | 0.05 | 4.16 |
| 2015 | D_EH_ | 1092776_PAV | 1B | 148.71 | 0.00 | 3.32 |
| 2015 | cPAR_AM_ | 1003008_SNP | 1B | 149.56 | 0.04 | 3.32 |
| 2015 | cPAR_AM_ | 3026386_PAV | 1B | 150.14 | 0.04 | 4.12 |
| 2014 | PH | 2332066_SNP | 1B | 152.02 | 0.04 | 3.06 |
| 2015 | cPAR_AM_ | 2332066_SNP | 1B | 152.02 | 0.04 | 3.06 |
| 2013 | Yield | 3023424_SNP | 1B | 156.1 | 0.08 | 4.58 |
| 2013 | Yield | 1091858_SNP | 1B | 159.81 | 0.06 | 4.69 |
| 2013 | Yield | 978671_SNP | 1B | 160.01 | 0.05 | 4.04 |
| 2013 | Yield | 1128834_PAV | 1B | 160.45 | 0.03 | 3.00 |
| 2013 | Yield | 1138583_PAV | 1B | 160.45 | 0.04 | 3.84 |
| 2013 | RUE_EA_ | 1238842_SNP | 1B | 160.7 | 0.04 | 3.10 |
| 2014 | PH | 4411722_PAV | 1B | 160.7 | 0.05 | 5.00 |
| 2013 | DM_M_ | 3222477_SNP | 1B | 161.64 | 0.05 | 3.76 |
| 2013 | RUE_AM_ | 3222477_SNP | 1B | 161.64 | 0.05 | 3.64 |
| 2013 | DM_M_ | 2282538_SNP | 1B | 162.4 | 0.05 | 4.02 |
| 2013 | RUE_AM_ | 2282538_SNP | 1B | 162.4 | 0.05 | 3.91 |
| 2014 | PH | 2282538_SNP | 1B | 162.4 | 0.04 | 3.10 |
| 2013 | DM_M_ | 3022822_PAV | 1B | 165.41 | 0.03 | 3.23 |
| 2013 | DM_M_ | 1382807_PAV | 1B | 167.68 | 0.05 | 4.27 |
| 2013 | RUE_AM_ | 1382807_PAV | 1B | 167.68 | 0.04 | 3.73 |
| 2013 | DM_M_ | 3027436_SNP | 1B | 167.68 | 0.04 | 3.22 |
| 2014 | PH | 1112822_SNP | 1B | 171.5 | 0.05 | 3.64 |
| 2014 | GA_90d_ | 1401481_PAV | 1B | 173.39 | 0.03 | 3.00 |
| 2014 | PH | 3026994_SNP | 1B | 174.61 | 0.04 | 3.32 |
| 2015 | DM_M_ | 4406174_PAV | 1B | 194.78 | 0.03 | 3.08 |
| 2015 | HI | 1104353_SNP | 1B | 196.97 | 0.04 | 3.64 |
| 2013 | Yield | 1287515_PAV | 1B | 197.08 | 0.03 | 3.42 |
| 2014 | W | 4404267_PAV | 1B | 197.23 | 0.08 | 6.81 |
| 2013 | GFR | 4404267_PAV | 1B | 197.23 | 0.05 | 4.53 |
| 2015 | W | 4404267_PAV | 1B | 197.23 | 0.05 | 4.46 |
| 2013 | W | 4404267_PAV | 1B | 197.23 | 0.05 | 4.36 |
| 2015 | GFR | 4404267_PAV | 1B | 197.23 | 0.04 | 3.70 |
| 2014 | GFR | 4404267_PAV | 1B | 197.23 | 0.04 | 3.61 |
| 2014 | NGS | 4404267_PAV | 1B | 197.23 | 0.04 | 3.58 |
| 2015 | D_HA_ | 1034271_SNP | 1B | 199.39 | 0.03 | 3.05 |
| 2013 | Yield | 1073456_PAV | 1B | 201.65 | 0.03 | 3.23 |
| 2014 | PH | 3935151_PAV | 1B | 203.41 | 0.03 | 3.10 |
| 2013 | GA_90d_ | 3533994_PAV | 1B | 208.96 | 0.05 | 4.16 |
| 2015 | cPAR_HA_ | 1253592_PAV | 1B | 221.4 | 0.03 | 3.39 |
| 2015 | D_HA_ | 1253592_PAV | 1B | 221.4 | 0.03 | 3.00 |
| 2014 | D_HA_ | 1022777_PAV | 1B | 223.47 | 0.02 | 3.02 |
| 2014 | PH | 2277906_PAV | 1B | 223.47 | 0.04 | 3.96 |
| 2014 | D_HA_ | 2277906_PAV | 1B | 223.47 | 0.03 | 3.67 |
| 2014 | D_HA_ | 3025726_PAV | 1B | 223.47 | 0.03 | 3.73 |
| 2014 | cPAR_HA_ | 3025726_PAV | 1B | 223.47 | 0.03 | 3.16 |
| 2014 | D_HA_ | 3940397_PAV | 1B | 223.47 | 0.02 | 3.13 |
| 2014 | D_HA_ | 3950074_PAV | 1B | 223.47 | 0.02 | 3.00 |
| 2014 | D_HA_ | 3960756_PAV | 1B | 223.47 | 0.02 | 3.13 |
| 2014 | D_HA_ | 1273584_PAV | 1B | 223.51 | 0.03 | 3.38 |
| 2015 | cPAR_HA_ | 1261486_SNP | 1B | 223.52 | 0.05 | 3.37 |
| 2015 | D_HA_ | 1261486_SNP | 1B | 223.52 | 0.04 | 3.03 |
| 2015 | NGS | 1136579_PAV | 1B | 241.91 | 0.03 | 3.32 |
| 2015 | DM_M_ | 1136579_PAV | 1B | 241.91 | 0.04 | 3.24 |
| 2015 | D_HA_ | 1082696_SNP | 1B | 274.71 | 0.04 | 3.00 |
| 2014 | RUE_AM_ | 996082_SNP | 1B | 279.65 | 0.03 | 3.23 |
| 2014 | CTD_Mi_ | 2277520_SNP | 2A | 8.29 | 0.06 | 4.18 |
| 2014 | PL | 1128568_SNP | 2A | 8.84 | 0.05 | 3.31 |
| 2015 | cPAR_HA_ | 1108319_PAV | 2A | 10.5 | 0.03 | 3.09 |
| 2015 | PH | 1072779_PAV | 2A | 11.57 | 0.03 | 3.10 |
| 2015 | GFR | 1087720_PAV | 2A | 11.57 | 0.05 | 4.97 |
| 2015 | W | 1087720_PAV | 2A | 11.57 | 0.04 | 4.18 |
| 2015 | Yield | 4394481_PAV | 2A | 13.34 | 0.04 | 3.33 |
| 2015 | GFR | 994759_PAV | 2A | 33.8 | 0.03 | 3.18 |
| 2015 | GA_90d_ | 4409299_PAV | 2A | 54.75 | 0.04 | 3.75 |
| 2014 | cPAR_AM_ | 991483_PAV | 2A | 58.21 | 0.03 | 3.16 |
| 2013 | DM_A_ | 4408728_PAV | 2A | 58.86 | 0.03 | 3.11 |
| 2013 | D_HA_ | 4009679_PAV | 2A | 59.1 | 0.03 | 3.65 |
| 2013 | NGS | 1098792_SNP | 2A | 59.3 | 0.05 | 3.60 |
| 2014 | HI | 1098792_SNP | 2A | 59.3 | 0.04 | 3.33 |
| 2013 | NGS | 4004922_PAV | 2A | 59.82 | 0.03 | 3.12 |
| 2014 | HI | 4004922_PAV | 2A | 59.82 | 0.03 | 3.01 |
| 2014 | PH | 1228155_PAV | 2A | 60.52 | 0.03 | 3.12 |
| 2014 | HI | 3034586_PAV | 2A | 60.52 | 0.03 | 3.08 |
| 2013 | W | 1092173_PAV | 2A | 62.4 | 0.06 | 4.67 |
| 2013 | GFR | 1092173_PAV | 2A | 62.4 | 0.05 | 3.99 |
| 2015 | W | 1092173_PAV | 2A | 62.4 | 0.04 | 3.66 |
| 2015 | GFR | 1092173_PAV | 2A | 62.4 | 0.04 | 3.35 |
| 2013 | NGS | 3938659_PAV | 2A | 62.6 | 0.05 | 4.89 |
| 2013 | GFR | 3938659_PAV | 2A | 62.6 | 0.05 | 4.58 |
| 2013 | W | 3938659_PAV | 2A | 62.6 | 0.05 | 4.45 |
| 2015 | W | 3938659_PAV | 2A | 62.6 | 0.04 | 3.47 |
| 2015 | GFR | 3938659_PAV | 2A | 62.6 | 0.03 | 3.35 |
| 2013 | GFR | 1111962_SNP | 2A | 62.93 | 0.04 | 3.16 |
| 2013 | GFR | 1137470_PAV | 2A | 62.93 | 0.04 | 3.46 |
| 2013 | GFR | 1140854_PAV | 2A | 62.93 | 0.04 | 3.68 |
| 2013 | W | 1166053_SNP | 2A | 62.93 | 0.06 | 4.99 |
| 2013 | GFR | 1166053_SNP | 2A | 62.93 | 0.06 | 4.80 |
| 2015 | W | 1166053_SNP | 2A | 62.93 | 0.04 | 3.37 |
| 2013 | W | 1166101_SNP | 2A | 62.93 | 0.05 | 3.74 |
| 2013 | GFR | 1166101_SNP | 2A | 62.93 | 0.05 | 3.66 |
| 2014 | W | 1166101_SNP | 2A | 62.93 | 0.05 | 3.36 |
| 2015 | GFR | 3025548_SNP | 2A | 62.93 | 0.04 | 3.15 |
| 2015 | W | 3025548_SNP | 2A | 62.93 | 0.04 | 3.12 |
| 2013 | W | 3025548_SNP | 2A | 62.93 | 0.04 | 3.03 |
| 2014 | W | 3031798_PAV | 2A | 62.93 | 0.04 | 3.37 |
| 2013 | GFR | 3031798_PAV | 2A | 62.93 | 0.03 | 3.16 |
| 2015 | W | 3031798_PAV | 2A | 62.93 | 0.04 | 3.15 |
| 2013 | W | 3031798_PAV | 2A | 62.93 | 0.03 | 3.05 |
| 2013 | W | 982312_PAV | 2A | 62.93 | 0.07 | 6.61 |
| 2013 | GFR | 982312_PAV | 2A | 62.93 | 0.07 | 6.34 |
| 2015 | W | 982312_PAV | 2A | 62.93 | 0.05 | 4.74 |
| 2015 | GFR | 982312_PAV | 2A | 62.93 | 0.04 | 3.99 |
| 2014 | W | 982312_PAV | 2A | 62.93 | 0.03 | 3.11 |
| 2013 | W | 3941903_PAV | 2A | 63.31 | 0.06 | 5.47 |
| 2013 | GFR | 3941903_PAV | 2A | 63.31 | 0.05 | 4.76 |
| 2015 | W | 3941903_PAV | 2A | 63.31 | 0.05 | 4.75 |
| 2015 | GFR | 3941903_PAV | 2A | 63.31 | 0.05 | 4.62 |
| 2013 | W | 3943192_PAV | 2A | 63.31 | 0.06 | 5.47 |
| 2013 | GFR | 3943192_PAV | 2A | 63.31 | 0.05 | 4.76 |
| 2015 | W | 3943192_PAV | 2A | 63.31 | 0.05 | 4.75 |
| 2015 | GFR | 3943192_PAV | 2A | 63.31 | 0.05 | 4.62 |
| 2013 | W | 979718_SNP | 2A | 63.5 | 0.04 | 3.46 |
| 2013 | GFR | 979718_SNP | 2A | 63.5 | 0.04 | 3.18 |
| 2014 | W | 4408487_PAV | 2A | 63.56 | 0.03 | 3.00 |
| 2013 | W | 1049695_PAV | 2A | 63.69 | 0.06 | 5.68 |
| 2013 | GFR | 1049695_PAV | 2A | 63.69 | 0.06 | 5.43 |
| 2015 | W | 1049695_PAV | 2A | 63.69 | 0.04 | 3.96 |
| 2015 | GFR | 1049695_PAV | 2A | 63.69 | 0.04 | 3.94 |
| 2013 | GFR | 1208262_PAV | 2A | 63.69 | 0.05 | 4.68 |
| 2013 | W | 1208262_PAV | 2A | 63.69 | 0.05 | 4.57 |
| 2015 | GFR | 1208262_PAV | 2A | 63.69 | 0.04 | 3.51 |
| 2015 | W | 1208262_PAV | 2A | 63.69 | 0.04 | 3.40 |
| 2013 | W | 979688_SNP | 2A | 64.05 | 0.05 | 3.90 |
| 2013 | GFR | 979688_SNP | 2A | 64.05 | 0.05 | 3.73 |
| 2013 | W | 1082459_PAV | 2A | 64.27 | 0.05 | 4.82 |
| 2013 | GFR | 1082459_PAV | 2A | 64.27 | 0.05 | 4.81 |
| 2013 | W | 3944635_PAV | 2A | 64.27 | 0.05 | 4.44 |
| 2013 | GFR | 3944635_PAV | 2A | 64.27 | 0.05 | 4.32 |
| 2014 | W | 3944635_PAV | 2A | 64.27 | 0.03 | 3.22 |
| 2013 | GFR | 4005236_PAV | 2A | 64.27 | 0.04 | 3.64 |
| 2013 | GFR | 4018220_PAV | 2A | 64.27 | 0.04 | 3.42 |
| 2014 | W | 4406134_PAV | 2A | 64.27 | 0.08 | 6.23 |
| 2013 | GFR | 4406134_PAV | 2A | 64.27 | 0.06 | 4.96 |
| 2015 | W | 4406134_PAV | 2A | 64.27 | 0.06 | 4.95 |
| 2015 | GFR | 4406134_PAV | 2A | 64.27 | 0.05 | 4.58 |
| 2013 | W | 4406134_PAV | 2A | 64.27 | 0.05 | 4.42 |
| 2014 | NGS | 4406134_PAV | 2A | 64.27 | 0.05 | 4.34 |
| 2015 | NGS | 4406134_PAV | 2A | 64.27 | 0.04 | 3.28 |
| 2013 | W | 4409935_PAV | 2A | 64.27 | 0.05 | 4.29 |
| 2013 | GFR | 4409935_PAV | 2A | 64.27 | 0.05 | 3.80 |
| 2013 | W | 3937645_PAV | 2A | 64.37 | 0.04 | 3.81 |
| 2015 | W | 3937645_PAV | 2A | 64.37 | 0.04 | 3.65 |
| 2015 | GFR | 3937645_PAV | 2A | 64.37 | 0.03 | 3.37 |
| 2013 | GFR | 3937645_PAV | 2A | 64.37 | 0.03 | 3.19 |
| 2014 | W | 3937645_PAV | 2A | 64.37 | 0.03 | 3.08 |
| 2015 | W | 3951022_PAV | 2A | 64.37 | 0.03 | 3.42 |
| 2015 | GFR | 3951022_PAV | 2A | 64.37 | 0.03 | 3.38 |
| 2014 | W | 3961370_PAV | 2A | 64.37 | 0.03 | 3.29 |
| 2014 | W | 4404812_PAV | 2A | 64.37 | 0.03 | 3.00 |
| 2015 | W | 3955811_PAV | 2A | 64.4 | 0.03 | 3.42 |
| 2015 | GFR | 3955811_PAV | 2A | 64.4 | 0.03 | 3.38 |
| 2013 | W | 2339487_PAV | 2A | 64.49 | 0.04 | 4.01 |
| 2013 | GFR | 2339487_PAV | 2A | 64.49 | 0.04 | 3.46 |
| 2015 | GFR | 2339487_PAV | 2A | 64.49 | 0.03 | 3.05 |
| 2013 | W | 3958547_PAV | 2A | 64.49 | 0.07 | 6.31 |
| 2013 | GFR | 3958547_PAV | 2A | 64.49 | 0.06 | 5.58 |
| 2015 | W | 3958547_PAV | 2A | 64.49 | 0.06 | 5.35 |
| 2015 | GFR | 3958547_PAV | 2A | 64.49 | 0.05 | 5.23 |
| 2013 | GFR | 1131599_SNP | 2A | 64.67 | 0.06 | 4.06 |
| 2013 | W | 1131599_SNP | 2A | 64.67 | 0.05 | 3.87 |
| 2013 | W | 1258244_PAV | 2A | 65.11 | 0.06 | 5.47 |
| 2015 | W | 1258244_PAV | 2A | 65.11 | 0.05 | 4.94 |
| 2015 | GFR | 1258244_PAV | 2A | 65.11 | 0.05 | 4.69 |
| 2013 | GFR | 1258244_PAV | 2A | 65.11 | 0.05 | 4.53 |
| 2013 | W | 3937685_PAV | 2A | 65.11 | 0.06 | 5.47 |
| 2013 | GFR | 3937685_PAV | 2A | 65.11 | 0.05 | 4.76 |
| 2015 | W | 3937685_PAV | 2A | 65.11 | 0.05 | 4.75 |
| 2015 | GFR | 3937685_PAV | 2A | 65.11 | 0.05 | 4.62 |
| 2013 | W | 4261365_PAV | 2A | 65.11 | 0.03 | 3.29 |
| 2015 | NGS | 4261365_PAV | 2A | 65.11 | 0.03 | 3.19 |
| 2014 | W | 1041769_SNP | 2A | 65.7 | 0.11 | 8.27 |
| 2013 | GFR | 1041769_SNP | 2A | 65.7 | 0.06 | 5.00 |
| 2013 | W | 1041769_SNP | 2A | 65.7 | 0.06 | 4.86 |
| 2015 | W | 1041769_SNP | 2A | 65.7 | 0.06 | 4.83 |
| 2015 | GFR | 1041769_SNP | 2A | 65.7 | 0.06 | 4.67 |
| 2014 | NGS | 1041769_SNP | 2A | 65.7 | 0.05 | 4.16 |
| 2013 | NGS | 1041769_SNP | 2A | 65.7 | 0.05 | 3.73 |
| 2014 | GFR | 1041769_SNP | 2A | 65.7 | 0.05 | 3.66 |
| 2013 | W | 1052193_PAV | 2A | 65.7 | 0.04 | 3.94 |
| 2013 | GFR | 1052193_PAV | 2A | 65.7 | 0.04 | 3.65 |
| 2013 | W | 1159200_SNP | 2A | 65.7 | 0.05 | 4.04 |
| 2013 | GFR | 1159200_SNP | 2A | 65.7 | 0.05 | 3.94 |
| 2013 | W | 1163763_PAV | 2A | 65.7 | 0.04 | 3.80 |
| 2013 | GFR | 1163763_PAV | 2A | 65.7 | 0.04 | 3.58 |
| 2014 | W | 1163763_PAV | 2A | 65.7 | 0.04 | 3.41 |
| 2013 | DM_A_ | 1194498_SNP | 2A | 65.7 | 0.04 | 3.38 |
| 2014 | W | 1260329_SNP | 2A | 65.7 | 0.10 | 7.54 |
| 2013 | NGS | 1260329_SNP | 2A | 65.7 | 0.07 | 5.36 |
| 2014 | NGS | 1260329_SNP | 2A | 65.7 | 0.06 | 4.67 |
| 2015 | W | 1260329_SNP | 2A | 65.7 | 0.05 | 4.32 |
| 2015 | GFR | 1260329_SNP | 2A | 65.7 | 0.05 | 4.01 |
| 2014 | GFR | 1260329_SNP | 2A | 65.7 | 0.05 | 3.84 |
| 2013 | GFR | 1260329_SNP | 2A | 65.7 | 0.04 | 3.47 |
| 2013 | W | 1260329_SNP | 2A | 65.7 | 0.04 | 3.31 |
| 2014 | W | 1268930_PAV | 2A | 65.7 | 0.07 | 5.95 |
| 2013 | GFR | 1268930_PAV | 2A | 65.7 | 0.05 | 4.41 |
| 2013 | W | 1268930_PAV | 2A | 65.7 | 0.05 | 4.09 |
| 2015 | W | 1268930_PAV | 2A | 65.7 | 0.05 | 3.85 |
| 2015 | GFR | 1268930_PAV | 2A | 65.7 | 0.04 | 3.43 |
| 2015 | NGS | 1268930_PAV | 2A | 65.7 | 0.04 | 3.32 |
| 2014 | NGS | 1268930_PAV | 2A | 65.7 | 0.04 | 3.24 |
| 2014 | W | 2256769_PAV | 2A | 65.7 | 0.05 | 4.32 |
| 2013 | W | 2256769_PAV | 2A | 65.7 | 0.04 | 4.25 |
| 2013 | GFR | 2256769_PAV | 2A | 65.7 | 0.04 | 4.21 |
| 2015 | W | 2256769_PAV | 2A | 65.7 | 0.03 | 3.16 |
| 2015 | GFR | 2256769_PAV | 2A | 65.7 | 0.03 | 3.09 |
| 2014 | GFR | 2256769_PAV | 2A | 65.7 | 0.03 | 3.00 |
| 2013 | W | 2260686_SNP | 2A | 65.7 | 0.04 | 3.05 |
| 2015 | GFR | 2260959_SNP | 2A | 65.7 | 0.06 | 4.47 |
| 2015 | W | 2260959_SNP | 2A | 65.7 | 0.06 | 4.40 |
| 2014 | W | 2260959_SNP | 2A | 65.7 | 0.06 | 4.39 |
| 2013 | W | 2260959_SNP | 2A | 65.7 | 0.04 | 3.19 |
| 2014 | W | 2289770_PAV | 2A | 65.7 | 0.11 | 9.32 |
| 2013 | GFR | 2289770_PAV | 2A | 65.7 | 0.08 | 6.34 |
| 2013 | W | 2289770_PAV | 2A | 65.7 | 0.07 | 6.05 |
| 2015 | W | 2289770_PAV | 2A | 65.7 | 0.07 | 5.69 |
| 2014 | NGS | 2289770_PAV | 2A | 65.7 | 0.06 | 5.62 |
| 2015 | GFR | 2289770_PAV | 2A | 65.7 | 0.06 | 5.41 |
| 2014 | GFR | 2289770_PAV | 2A | 65.7 | 0.05 | 4.39 |
| 2013 | NGS | 2289770_PAV | 2A | 65.7 | 0.04 | 3.99 |
| 2015 | NGS | 2289770_PAV | 2A | 65.7 | 0.04 | 3.84 |
| 2015 | GFR | 2322321_PAV | 2A | 65.7 | 0.03 | 3.46 |
| 2015 | W | 2322321_PAV | 2A | 65.7 | 0.03 | 3.17 |
| 2013 | GA_90d_ | 2322321_PAV | 2A | 65.7 | 0.03 | 3.09 |
| 2013 | W | 3027761_PAV | 2A | 65.7 | 0.03 | 3.15 |
| 2013 | GFR | 3027761_PAV | 2A | 65.7 | 0.03 | 3.06 |
| 2013 | W | 4008067_PAV | 2A | 65.7 | 0.05 | 4.86 |
| 2013 | GFR | 4008067_PAV | 2A | 65.7 | 0.05 | 4.57 |
| 2015 | GFR | 4008067_PAV | 2A | 65.7 | 0.04 | 3.98 |
| 2015 | W | 4008067_PAV | 2A | 65.7 | 0.05 | 3.95 |
| 2014 | W | 4008067_PAV | 2A | 65.7 | 0.04 | 3.27 |
| 2013 | W | 1000828_PAV | 2A | 65.76 | 0.05 | 4.90 |
| 2013 | GFR | 1000828_PAV | 2A | 65.76 | 0.05 | 4.39 |
| 2015 | W | 1000828_PAV | 2A | 65.76 | 0.04 | 3.65 |
| 2015 | GFR | 1000828_PAV | 2A | 65.76 | 0.03 | 3.52 |
| 2014 | NGS | 1264542_PAV | 2A | 65.76 | 0.05 | 4.05 |
| 2014 | W | 1264542_PAV | 2A | 65.76 | 0.04 | 3.09 |
| 2014 | GFR | 1264542_PAV | 2A | 65.76 | 0.03 | 3.00 |
| 2013 | W | 3940754_PAV | 2A | 65.76 | 0.04 | 4.14 |
| 2013 | GFR | 3940754_PAV | 2A | 65.76 | 0.04 | 3.78 |
| 2013 | GA_90d_ | 3940754_PAV | 2A | 65.76 | 0.04 | 3.61 |
| 2015 | D_EH_ | 4406006_PAV | 2A | 65.86 | 0.01 | 3.87 |
| 2014 | D_EH_ | 4409291_PAV | 2A | 65.86 | 0.00 | 3.72 |
| 2015 | D_EH_ | 4410089_PAV | 2A | 65.86 | 0.01 | 3.87 |
| 2013 | W | 1670626_PAV | 2A | 65.96 | 0.04 | 3.92 |
| 2013 | GFR | 1670626_PAV | 2A | 65.96 | 0.04 | 3.86 |
| 2014 | W | 1670626_PAV | 2A | 65.96 | 0.03 | 3.12 |
| 2013 | W | 3946241_PAV | 2A | 65.96 | 0.05 | 4.73 |
| 2013 | GFR | 3946241_PAV | 2A | 65.96 | 0.05 | 4.45 |
| 2015 | W | 3946241_PAV | 2A | 65.96 | 0.03 | 3.22 |
| 2015 | GFR | 3946241_PAV | 2A | 65.96 | 0.03 | 3.11 |
| 2015 | GFR | 1106212_SNP | 2A | 66.06 | 0.08 | 5.62 |
| 2015 | W | 1106212_SNP | 2A | 66.06 | 0.08 | 5.32 |
| 2014 | W | 1106212_SNP | 2A | 66.06 | 0.08 | 5.01 |
| 2013 | GFR | 1106212_SNP | 2A | 66.06 | 0.06 | 4.23 |
| 2013 | W | 1106212_SNP | 2A | 66.06 | 0.06 | 3.93 |
| 2013 | NGS | 1106212_SNP | 2A | 66.06 | 0.06 | 3.86 |
| 2014 | W | 4004953_PAV | 2A | 66.68 | 0.04 | 3.50 |
| 2014 | W | 1077708_SNP | 2A | 66.9 | 0.11 | 7.94 |
| 2015 | GFR | 1077708_SNP | 2A | 66.9 | 0.10 | 7.78 |
| 2015 | W | 1077708_SNP | 2A | 66.9 | 0.10 | 7.71 |
| 2014 | NGS | 1077708_SNP | 2A | 66.9 | 0.07 | 4.93 |
| 2013 | W | 1077708_SNP | 2A | 66.9 | 0.06 | 4.77 |
| 2015 | NGS | 1077708_SNP | 2A | 66.9 | 0.06 | 4.58 |
| 2013 | GFR | 1077708_SNP | 2A | 66.9 | 0.06 | 4.31 |
| 2013 | NGS | 1077708_SNP | 2A | 66.9 | 0.05 | 3.80 |
| 2014 | GFR | 1077708_SNP | 2A | 66.9 | 0.05 | 3.63 |
| 2015 | W | 1091747_SNP | 2A | 66.9 | 0.10 | 7.62 |
| 2015 | GFR | 1091747_SNP | 2A | 66.9 | 0.09 | 6.85 |
| 2014 | W | 1091747_SNP | 2A | 66.9 | 0.08 | 6.44 |
| 2015 | NGS | 1091747_SNP | 2A | 66.9 | 0.07 | 5.09 |
| 2014 | NGS | 1091747_SNP | 2A | 66.9 | 0.06 | 4.90 |
| 2013 | NGS | 1091747_SNP | 2A | 66.9 | 0.06 | 4.83 |
| 2013 | W | 1091747_SNP | 2A | 66.9 | 0.06 | 4.69 |
| 2013 | GFR | 1091747_SNP | 2A | 66.9 | 0.05 | 4.30 |
| 2014 | GFR | 1091747_SNP | 2A | 66.9 | 0.05 | 3.66 |
| 2015 | GFR | 1150369_SNP | 2A | 66.9 | 0.08 | 7.32 |
| 2014 | W | 1150369_SNP | 2A | 66.9 | 0.09 | 7.27 |
| 2015 | W | 1150369_SNP | 2A | 66.9 | 0.08 | 7.04 |
| 2015 | NGS | 1150369_SNP | 2A | 66.9 | 0.07 | 5.77 |
| 2014 | NGS | 1150369_SNP | 2A | 66.9 | 0.06 | 5.43 |
| 2013 | W | 1150369_SNP | 2A | 66.9 | 0.06 | 5.36 |
| 2013 | GFR | 1150369_SNP | 2A | 66.9 | 0.05 | 4.98 |
| 2013 | NGS | 1150369_SNP | 2A | 66.9 | 0.05 | 4.57 |
| 2014 | GFR | 1150369_SNP | 2A | 66.9 | 0.04 | 3.70 |
| 2014 | W | 1672979_PAV | 2A | 66.9 | 0.10 | 8.56 |
| 2015 | GFR | 1672979_PAV | 2A | 66.9 | 0.09 | 8.04 |
| 2015 | W | 1672979_PAV | 2A | 66.9 | 0.09 | 7.92 |
| 2014 | NGS | 1672979_PAV | 2A | 66.9 | 0.07 | 6.23 |
| 2015 | NGS | 1672979_PAV | 2A | 66.9 | 0.07 | 6.22 |
| 2013 | W | 1672979_PAV | 2A | 66.9 | 0.06 | 6.00 |
| 2013 | GFR | 1672979_PAV | 2A | 66.9 | 0.06 | 5.53 |
| 2013 | NGS | 1672979_PAV | 2A | 66.9 | 0.06 | 5.19 |
| 2014 | GFR | 1672979_PAV | 2A | 66.9 | 0.04 | 4.10 |
| 2014 | W | 2276567_SNP | 2A | 66.9 | 0.06 | 4.49 |
| 2015 | GFR | 2276567_SNP | 2A | 66.9 | 0.04 | 3.63 |
| 2014 | NGS | 2276567_SNP | 2A | 66.9 | 0.04 | 3.49 |
| 2015 | W | 2276567_SNP | 2A | 66.9 | 0.04 | 3.20 |
| 2014 | W | 2293689_PAV | 2A | 66.9 | 0.10 | 8.81 |
| 2015 | GFR | 2293689_PAV | 2A | 66.9 | 0.09 | 8.48 |
| 2015 | W | 2293689_PAV | 2A | 66.9 | 0.10 | 8.33 |
| 2014 | NGS | 2293689_PAV | 2A | 66.9 | 0.06 | 5.76 |
| 2013 | W | 2293689_PAV | 2A | 66.9 | 0.06 | 5.52 |
| 2015 | NGS | 2293689_PAV | 2A | 66.9 | 0.06 | 5.31 |
| 2013 | GFR | 2293689_PAV | 2A | 66.9 | 0.06 | 5.08 |
| 2013 | NGS | 2293689_PAV | 2A | 66.9 | 0.05 | 4.46 |
| 2014 | GFR | 2293689_PAV | 2A | 66.9 | 0.05 | 4.38 |
| 2015 | W | 991737_SNP | 2A | 66.9 | 0.05 | 3.86 |
| 2015 | GFR | 991737_SNP | 2A | 66.9 | 0.04 | 3.23 |
| 2015 | GFR | 1053788_SNP | 2A | 66.91 | 0.09 | 7.04 |
| 2015 | W | 1053788_SNP | 2A | 66.91 | 0.09 | 6.87 |
| 2014 | W | 1053788_SNP | 2A | 66.91 | 0.09 | 6.60 |
| 2015 | NGS | 1053788_SNP | 2A | 66.91 | 0.08 | 6.33 |
| 2013 | NGS | 1053788_SNP | 2A | 66.91 | 0.07 | 5.14 |
| 2013 | W | 1053788_SNP | 2A | 66.91 | 0.06 | 4.95 |
| 2013 | GFR | 1053788_SNP | 2A | 66.91 | 0.06 | 4.84 |
| 2014 | GFR | 1053788_SNP | 2A | 66.91 | 0.05 | 4.27 |
| 2014 | NGS | 1053788_SNP | 2A | 66.91 | 0.05 | 3.99 |
| 2014 | W | 3944344_PAV | 2A | 66.94 | 0.10 | 8.61 |
| 2015 | GFR | 3944344_PAV | 2A | 66.94 | 0.09 | 8.05 |
| 2015 | W | 3944344_PAV | 2A | 66.94 | 0.09 | 7.90 |
| 2014 | NGS | 3944344_PAV | 2A | 66.94 | 0.07 | 6.27 |
| 2015 | NGS | 3944344_PAV | 2A | 66.94 | 0.07 | 6.25 |
| 2013 | W | 3944344_PAV | 2A | 66.94 | 0.06 | 5.97 |
| 2013 | GFR | 3944344_PAV | 2A | 66.94 | 0.06 | 5.53 |
| 2013 | NGS | 3944344_PAV | 2A | 66.94 | 0.06 | 5.21 |
| 2014 | GFR | 3944344_PAV | 2A | 66.94 | 0.04 | 4.15 |
| 2014 | W | 4394232_PAV | 2A | 66.94 | 0.10 | 8.36 |
| 2015 | GFR | 4394232_PAV | 2A | 66.94 | 0.09 | 7.96 |
| 2015 | W | 4394232_PAV | 2A | 66.94 | 0.09 | 7.45 |
| 2014 | NGS | 4394232_PAV | 2A | 66.94 | 0.07 | 6.42 |
| 2015 | NGS | 4394232_PAV | 2A | 66.94 | 0.07 | 6.06 |
| 2013 | W | 4394232_PAV | 2A | 66.94 | 0.06 | 5.84 |
| 2013 | GFR | 4394232_PAV | 2A | 66.94 | 0.07 | 5.81 |
| 2013 | NGS | 4394232_PAV | 2A | 66.94 | 0.05 | 4.75 |
| 2014 | GFR | 4394232_PAV | 2A | 66.94 | 0.04 | 4.14 |
| 2014 | W | 4394461_PAV | 2A | 66.94 | 0.07 | 6.30 |
| 2013 | GFR | 4394461_PAV | 2A | 66.94 | 0.06 | 5.24 |
| 2013 | W | 4394461_PAV | 2A | 66.94 | 0.05 | 5.09 |
| 2015 | W | 4394461_PAV | 2A | 66.94 | 0.05 | 4.43 |
| 2015 | NGS | 4394461_PAV | 2A | 66.94 | 0.05 | 4.42 |
| 2015 | GFR | 4394461_PAV | 2A | 66.94 | 0.04 | 4.33 |
| 2014 | GFR | 4394461_PAV | 2A | 66.94 | 0.04 | 4.07 |
| 2014 | NGS | 4394461_PAV | 2A | 66.94 | 0.04 | 3.58 |
| 2013 | NGS | 4394461_PAV | 2A | 66.94 | 0.03 | 3.36 |
| 2013 | NGS | 1038970_PAV | 2A | 67.25 | 0.03 | 3.08 |
| 2015 | W | 1140354_SNP | 2A | 67.31 | 0.09 | 6.79 |
| 2015 | GFR | 1140354_SNP | 2A | 67.31 | 0.09 | 6.63 |
| 2014 | W | 1140354_SNP | 2A | 67.31 | 0.09 | 6.24 |
| 2015 | NGS | 1140354_SNP | 2A | 67.31 | 0.07 | 5.48 |
| 2013 | W | 1140354_SNP | 2A | 67.31 | 0.06 | 4.89 |
| 2014 | NGS | 1140354_SNP | 2A | 67.31 | 0.06 | 4.55 |
| 2013 | GFR | 1140354_SNP | 2A | 67.31 | 0.06 | 4.24 |
| 2013 | NGS | 1140354_SNP | 2A | 67.31 | 0.05 | 3.72 |
| 2014 | GFR | 1140354_SNP | 2A | 67.31 | 0.04 | 3.21 |
| 2014 | W | 1698984_PAV | 2A | 67.35 | 0.10 | 8.62 |
| 2015 | GFR | 1698984_PAV | 2A | 67.35 | 0.09 | 8.08 |
| 2015 | W | 1698984_PAV | 2A | 67.35 | 0.09 | 7.96 |
| 2014 | NGS | 1698984_PAV | 2A | 67.35 | 0.07 | 6.31 |
| 2015 | NGS | 1698984_PAV | 2A | 67.35 | 0.07 | 6.27 |
| 2013 | W | 1698984_PAV | 2A | 67.35 | 0.06 | 5.95 |
| 2013 | GFR | 1698984_PAV | 2A | 67.35 | 0.06 | 5.52 |
| 2013 | NGS | 1698984_PAV | 2A | 67.35 | 0.06 | 5.21 |
| 2014 | GFR | 1698984_PAV | 2A | 67.35 | 0.04 | 4.16 |
| 2015 | GFR | 2322963_PAV | 2A | 67.35 | 0.06 | 5.37 |
| 2014 | W | 2322963_PAV | 2A | 67.35 | 0.06 | 5.08 |
| 2015 | W | 2322963_PAV | 2A | 67.35 | 0.06 | 4.72 |
| 2015 | PH | 2275863_PAV | 2A | 67.47 | 0.03 | 3.06 |
| 2014 | PH | 2275863_PAV | 2A | 67.47 | 0.03 | 3.00 |
| 2013 | W | 3951735_PAV | 2A | 67.55 | 0.05 | 4.58 |
| 2013 | GFR | 3951735_PAV | 2A | 67.55 | 0.05 | 4.50 |
| 2015 | W | 3951735_PAV | 2A | 67.55 | 0.03 | 3.12 |
| 2015 | GFR | 3951735_PAV | 2A | 67.55 | 0.03 | 3.12 |
| 2013 | CTD_Mi_ | 4005021_PAV | 2A | 67.55 | 0.04 | 3.30 |
| 2014 | W | 4410530_PAV | 2A | 67.76 | 0.10 | 8.33 |
| 2015 | GFR | 4410530_PAV | 2A | 67.76 | 0.08 | 7.62 |
| 2015 | W | 4410530_PAV | 2A | 67.76 | 0.08 | 7.35 |
| 2014 | NGS | 4410530_PAV | 2A | 67.76 | 0.06 | 5.51 |
| 2015 | NGS | 4410530_PAV | 2A | 67.76 | 0.05 | 4.72 |
| 2013 | W | 4410530_PAV | 2A | 67.76 | 0.05 | 4.71 |
| 2013 | GFR | 4410530_PAV | 2A | 67.76 | 0.05 | 4.70 |
| 2013 | NGS | 4410530_PAV | 2A | 67.76 | 0.05 | 4.29 |
| 2014 | GFR | 4410530_PAV | 2A | 67.76 | 0.04 | 4.24 |
| 2015 | cPAR_EH_ | 4410530_PAV | 2A | 67.76 | 0.03 | 3.36 |
| 2014 | W | 1164339_PAV | 2A | 67.8 | 0.10 | 8.61 |
| 2015 | GFR | 1164339_PAV | 2A | 67.8 | 0.09 | 8.05 |
| 2015 | W | 1164339_PAV | 2A | 67.8 | 0.09 | 7.89 |
| 2015 | NGS | 1164339_PAV | 2A | 67.8 | 0.07 | 6.28 |
| 2014 | NGS | 1164339_PAV | 2A | 67.8 | 0.07 | 6.27 |
| 2013 | W | 1164339_PAV | 2A | 67.8 | 0.06 | 5.98 |
| 2013 | GFR | 1164339_PAV | 2A | 67.8 | 0.06 | 5.53 |
| 2013 | NGS | 1164339_PAV | 2A | 67.8 | 0.06 | 5.21 |
| 2014 | GFR | 1164339_PAV | 2A | 67.8 | 0.04 | 4.14 |
| 2014 | W | 1378550_PAV | 2A | 67.8 | 0.10 | 7.87 |
| 2015 | W | 1378550_PAV | 2A | 67.8 | 0.06 | 4.64 |
| 2013 | NGS | 1378550_PAV | 2A | 67.8 | 0.05 | 4.24 |
| 2014 | NGS | 1378550_PAV | 2A | 67.8 | 0.05 | 3.97 |
| 2013 | W | 1378550_PAV | 2A | 67.8 | 0.04 | 3.82 |
| 2015 | GFR | 1378550_PAV | 2A | 67.8 | 0.04 | 3.77 |
| 2013 | GFR | 1378550_PAV | 2A | 67.8 | 0.04 | 3.59 |
| 2014 | GFR | 1378550_PAV | 2A | 67.8 | 0.04 | 3.22 |
| 2013 | W | 1115897_PAV | 2A | 67.81 | 0.06 | 4.71 |
| 2013 | GFR | 1115897_PAV | 2A | 67.81 | 0.05 | 4.29 |
| 2015 | W | 1115897_PAV | 2A | 67.81 | 0.04 | 3.21 |
| 2015 | GFR | 1115897_PAV | 2A | 67.81 | 0.03 | 3.21 |
| 2013 | W | 2275484_PAV | 2A | 67.81 | 0.06 | 4.95 |
| 2013 | GFR | 2275484_PAV | 2A | 67.81 | 0.05 | 4.40 |
| 2015 | W | 2275484_PAV | 2A | 67.81 | 0.04 | 3.59 |
| 2015 | GFR | 2275484_PAV | 2A | 67.81 | 0.03 | 3.23 |
| 2015 | W | 2277559_PAV | 2A | 68.03 | 0.10 | 7.28 |
| 2015 | GFR | 2277559_PAV | 2A | 68.03 | 0.09 | 7.11 |
| 2014 | W | 2277559_PAV | 2A | 68.03 | 0.09 | 6.85 |
| 2015 | NGS | 2277559_PAV | 2A | 68.03 | 0.07 | 5.91 |
| 2014 | NGS | 2277559_PAV | 2A | 68.03 | 0.07 | 5.75 |
| 2013 | W | 2277559_PAV | 2A | 68.03 | 0.06 | 5.09 |
| 2013 | GFR | 2277559_PAV | 2A | 68.03 | 0.06 | 4.64 |
| 2013 | NGS | 2277559_PAV | 2A | 68.03 | 0.06 | 4.63 |
| 2014 | GFR | 2277559_PAV | 2A | 68.03 | 0.04 | 3.20 |
| 2013 | NGS | 1039194_PAV | 2A | 68.04 | 0.03 | 3.06 |
| 2014 | W | 2275846_PAV | 2A | 68.04 | 0.04 | 3.16 |
| 2014 | NGS | 4405819_PAV | 2A | 68.06 | 0.04 | 3.78 |
| 2014 | GFR | 3532728_PAV | 2A | 68.39 | 0.04 | 4.05 |
| 2014 | W | 3532728_PAV | 2A | 68.39 | 0.04 | 3.89 |
| 2015 | D_HA_ | 4411119_PAV | 2A | 68.39 | 0.03 | 3.04 |
| 2013 | W | 1261415_PAV | 2A | 68.49 | 0.06 | 5.71 |
| 2013 | GFR | 1261415_PAV | 2A | 68.49 | 0.05 | 5.04 |
| 2015 | W | 1261415_PAV | 2A | 68.49 | 0.05 | 4.63 |
| 2015 | GFR | 1261415_PAV | 2A | 68.49 | 0.05 | 4.56 |
| 2014 | W | 3575239_PAV | 2A | 68.5 | 0.05 | 4.41 |
| 2014 | GFR | 3575239_PAV | 2A | 68.5 | 0.04 | 3.47 |
| 2014 | CTD_A_ | 1025969_SNP | 2A | 68.57 | 0.04 | 3.11 |
| 2015 | CTD_Mi_ | 1135504_PAV | 2A | 68.57 | 0.03 | 3.03 |
| 2014 | W | 1235569_SNP | 2A | 68.57 | 0.10 | 7.52 |
| 2015 | W | 1235569_SNP | 2A | 68.57 | 0.09 | 7.37 |
| 2015 | GFR | 1235569_SNP | 2A | 68.57 | 0.09 | 7.04 |
| 2014 | NGS | 1235569_SNP | 2A | 68.57 | 0.08 | 5.84 |
| 2015 | NGS | 1235569_SNP | 2A | 68.57 | 0.06 | 4.39 |
| 2013 | W | 1235569_SNP | 2A | 68.57 | 0.05 | 4.30 |
| 2013 | GFR | 1235569_SNP | 2A | 68.57 | 0.05 | 4.30 |
| 2014 | GFR | 1235569_SNP | 2A | 68.57 | 0.05 | 4.18 |
| 2013 | NGS | 1235569_SNP | 2A | 68.57 | 0.04 | 3.19 |
| 2013 | NGS | 983804_PAV | 2A | 68.57 | 0.03 | 3.20 |
| 2013 | cPAR_AM_ | 985267_SNP | 2A | 68.57 | 0.04 | 3.07 |
| 2014 | DM_A_ | 992650_PAV | 2A | 68.57 | 0.04 | 3.21 |
| 2014 | RUE_EA_ | 2276438_PAV | 2A | 68.75 | 0.03 | 3.38 |
| 2014 | W | 1083407_SNP | 2A | 68.85 | 0.10 | 7.25 |
| 2015 | GFR | 1083407_SNP | 2A | 68.85 | 0.09 | 6.82 |
| 2014 | NGS | 1083407_SNP | 2A | 68.85 | 0.10 | 6.80 |
| 2015 | W | 1083407_SNP | 2A | 68.85 | 0.09 | 6.74 |
| 2015 | NGS | 1083407_SNP | 2A | 68.85 | 0.07 | 5.18 |
| 2013 | NGS | 1083407_SNP | 2A | 68.85 | 0.05 | 4.75 |
| 2013 | W | 1083407_SNP | 2A | 68.85 | 0.06 | 4.65 |
| 2013 | GFR | 1083407_SNP | 2A | 68.85 | 0.05 | 3.86 |
| 2013 | RUE_EA_ | 1083407_SNP | 2A | 68.85 | 0.05 | 3.64 |
| 2014 | GFR | 1083407_SNP | 2A | 68.85 | 0.05 | 3.52 |
| 2014 | NGS | 979169_SNP | 2A | 69.12 | 0.06 | 4.70 |
| 2014 | W | 979169_SNP | 2A | 69.12 | 0.04 | 3.21 |
| 2013 | PL | 979169_SNP | 2A | 69.12 | 0.04 | 3.08 |
| 2014 | GFR | 3222159_SNP | 2A | 69.27 | 0.05 | 4.02 |
| 2014 | D_AM_ | 3222159_SNP | 2A | 69.27 | 0.04 | 3.07 |
| 2014 | W | 1097891_SNP | 2A | 69.4 | 0.10 | 7.98 |
| 2013 | W | 1097891_SNP | 2A | 69.4 | 0.05 | 4.00 |
| 2015 | GFR | 1097891_SNP | 2A | 69.4 | 0.05 | 3.92 |
| 2013 | GFR | 1097891_SNP | 2A | 69.4 | 0.05 | 3.87 |
| 2015 | W | 1097891_SNP | 2A | 69.4 | 0.05 | 3.71 |
| 2014 | NGS | 2257424_SNP | 2A | 69.42 | 0.05 | 3.65 |
| 2014 | GFR | 1202353_PAV | 2A | 69.5 | 0.05 | 4.18 |
| 2015 | CTD_Mi_ | 1220072_PAV | 2A | 69.5 | 0.03 | 3.19 |
| 2014 | NGS | 984222_SNP | 2A | 69.66 | 0.08 | 6.16 |
| 2014 | GFR | 984222_SNP | 2A | 69.66 | 0.04 | 3.21 |
| 2014 | CTD_Mi_ | 1063031_SNP | 2A | 69.95 | 0.04 | 3.21 |
| 2014 | NGS | 3026593_SNP | 2A | 70.25 | 0.07 | 5.11 |
| 2014 | GFR | 1143754_PAV | 2A | 70.31 | 0.04 | 3.40 |
| 2014 | GFR | 3024502_PAV | 2A | 70.54 | 0.05 | 4.26 |
| 2014 | GFR | 1281681_PAV | 2A | 71.38 | 0.03 | 3.22 |
| 2014 | GFR | 1074137_PAV | 2A | 71.95 | 0.04 | 3.61 |
| 2014 | GFR | 1105928_PAV | 2A | 72.36 | 0.04 | 4.05 |
| 2013 | PH | 986598_PAV | 2A | 72.77 | 0.03 | 3.06 |
| 2015 | W | 1155046_PAV | 2A | 72.86 | 0.03 | 3.34 |
| 2015 | NGS | 979281_SNP | 2A | 72.86 | 0.05 | 4.07 |
| 2013 | GFR | 979281_SNP | 2A | 72.86 | 0.04 | 3.47 |
| 2015 | W | 979281_SNP | 2A | 72.86 | 0.04 | 3.47 |
| 2013 | W | 979281_SNP | 2A | 72.86 | 0.04 | 3.06 |
| 2014 | PL | 995187_SNP | 2A | 73.11 | 0.04 | 3.06 |
| 2013 | DM_A_ | 3937376_PAV | 2A | 73.96 | 0.03 | 3.31 |
| 2014 | PH | 985039_SNP | 2A | 74.3 | 0.05 | 3.34 |
| 2015 | D_EH_ | 1724214_PAV | 2A | 76.68 | 0.01 | 4.69 |
| 2013 | D_AM_ | 1093442_PAV | 2A | 77.3 | 0.03 | 3.17 |
| 2015 | RUE_EA_ | 4407769_PAV | 2A | 77.31 | 0.04 | 3.58 |
| 2015 | cPAR_EH_ | 4407769_PAV | 2A | 77.31 | 0.03 | 3.15 |
| 2013 | Yield | 2322447_PAV | 2A | 77.93 | 0.03 | 3.14 |
| 2015 | cPAR_HA_ | 1072243_SNP | 2A | 83.55 | 0.03 | 3.02 |
| 2015 | Yield | 3022273_SNP | 2A | 87.88 | 0.08 | 6.20 |
| 2015 | cPAR_AM_ | 3022273_SNP | 2A | 87.88 | 0.04 | 3.20 |
| 2014 | GFR | 3064509_SNP | 2A | 87.88 | 0.05 | 4.27 |
| 2014 | PL | 4008069_PAV | 2A | 90.88 | 0.03 | 3.12 |
| 2015 | CTD_A_ | 1092204_SNP | 2A | 95.59 | 0.04 | 3.17 |
| 2014 | D_EH_ | 4410202_PAV | 2A | 104.08 | 0.00 | 3.34 |
| 2013 | PH | 4404699_PAV | 2A | 109.77 | 0.03 | 3.00 |
| 2015 | PL | 1053545_SNP | 2A | 113.38 | 0.04 | 3.26 |
| 2014 | PH | 1053545_SNP | 2A | 113.38 | 0.04 | 3.05 |
| 2015 | GA_90d_ | 1161059_SNP | 2A | 114.61 | 0.04 | 3.10 |
| 2014 | cPAR_HA_ | 993009_PAV | 2A | 117.05 | 0.03 | 3.23 |
| 2013 | PL | 1699476_PAV | 2A | 120.2 | 0.03 | 3.28 |
| 2015 | HI | 3064546_SNP | 2A | 120.67 | 0.04 | 3.75 |
| 2013 | CTD_A_ | 1197618_SNP | 2A | 120.68 | 0.05 | 3.28 |
| 2013 | DM_M_ | 1671937_PAV | 2A | 120.7 | 0.03 | 3.04 |
| 2014 | cPAR_HA_ | 1040655_PAV | 2A | 120.72 | 0.03 | 3.32 |
| 2013 | PL | 1062662_PAV | 2A | 120.72 | 0.03 | 3.32 |
| 2013 | GA_90d_ | 1089389_PAV | 2A | 120.72 | 0.03 | 3.06 |
| 2014 | CTD_Mi_ | 1090922_SNP | 2A | 120.72 | 0.07 | 5.59 |
| 2013 | NGS | 2277232_SNP | 2A | 120.72 | 0.04 | 3.19 |
| 2013 | GA_90d_ | 2322279_PAV | 2A | 120.72 | 0.03 | 3.37 |
| 2013 | NGS | 2322279_PAV | 2A | 120.72 | 0.03 | 3.04 |
| 2013 | PL | 3027058_PAV | 2A | 120.72 | 0.03 | 3.34 |
| 2013 | GA_90d_ | 3940359_PAV | 2A | 121.12 | 0.03 | 3.05 |
| 2013 | NGS | 989009_PAV | 2A | 121.31 | 0.03 | 3.08 |
| 2013 | PL | 1017924_SNP | 2A | 123.66 | 0.04 | 3.11 |
| 2014 | CTD_Mi_ | 1068080_SNP | 2A | 123.66 | 0.05 | 3.87 |
| 2014 | D_HA_ | 1211034_PAV | 2A | 123.66 | 0.03 | 3.37 |
| 2014 | D_HA_ | 2276769_PAV | 2A | 123.66 | 0.03 | 3.25 |
| 2014 | D_HA_ | 2340355_PAV | 2A | 123.66 | 0.03 | 3.31 |
| 2014 | cPAR_EH_ | 4412102_PAV | 2A | 123.66 | 0.02 | 3.10 |
| 2014 | D_HA_ | 983560_PAV | 2A | 123.66 | 0.02 | 3.04 |
| 2014 | D_HA_ | 3533429_PAV | 2A | 123.76 | 0.03 | 3.33 |
| 2015 | CTD_A_ | 1021328_SNP | 2A | 124.28 | 0.04 | 3.39 |
| 2015 | D_AM_ | 4411287_PAV | 2A | 125.1 | 0.03 | 3.42 |
| 2013 | RUE_EA_ | 978665_PAV | 2B | 0.92 | 0.04 | 3.75 |
| 2015 | PL | 4404840_PAV | 2B | 0.98 | 0.03 | 3.06 |
| 2013 | RUE_EA_ | 4407701_PAV | 2B | 1.14 | 0.03 | 3.20 |
| 2014 | CTD_Mi_ | 2302674_PAV | 2B | 2.05 | 0.04 | 3.97 |
| 2014 | D_AM_ | 2302674_PAV | 2B | 2.05 | 0.03 | 3.18 |
| 2015 | PH | 4407811_PAV | 2B | 6.7 | 0.04 | 3.90 |
| 2015 | DM_M_ | 1241439_PAV | 2B | 10.64 | 0.03 | 3.25 |
| 2015 | HI | 3385538_PAV | 2B | 11.94 | 0.03 | 3.07 |
| 2014 | D_AM_ | 1108304_PAV | 2B | 17.84 | 0.03 | 3.29 |
| 2015 | NS | 2294554_PAV | 2B | 17.84 | 0.03 | 3.00 |
| 2015 | NS | 3025247_PAV | 2B | 17.84 | 0.04 | 3.95 |
| 2015 | DM_M_ | 3025247_PAV | 2B | 17.84 | 0.03 | 3.17 |
| 2015 | RUE_AM_ | 3025247_PAV | 2B | 17.84 | 0.03 | 3.16 |
| 2014 | D_AM_ | 3942520_PAV | 2B | 17.84 | 0.03 | 3.19 |
| 2013 | NGS | 1004475_SNP | 2B | 21.44 | 0.05 | 3.37 |
| 2015 | W | 4004030_PAV | 2B | 23.11 | 0.06 | 5.25 |
| 2015 | GFR | 4004030_PAV | 2B | 23.11 | 0.04 | 4.29 |
| 2015 | NGS | 4407578_PAV | 2B | 32.76 | 0.04 | 3.68 |
| 2013 | DM_M_ | 1016366_PAV | 2B | 37.15 | 0.04 | 3.80 |
| 2013 | DM_M_ | 1020538_PAV | 2B | 37.15 | 0.04 | 3.82 |
| 2013 | DM_M_ | 1028653_PAV | 2B | 37.15 | 0.05 | 5.00 |
| 2013 | RUE_AM_ | 1028653_PAV | 2B | 37.15 | 0.04 | 3.82 |
| 2013 | DM_M_ | 1072035_PAV | 2B | 37.15 | 0.04 | 3.80 |
| 2013 | RUE_AM_ | 1072035_PAV | 2B | 37.15 | 0.03 | 3.20 |
| 2013 | DM_M_ | 2275497_SNP | 2B | 37.15 | 0.04 | 3.20 |
| 2013 | RUE_AM_ | 2275497_SNP | 2B | 37.15 | 0.04 | 3.05 |
| 2015 | W | 1115679_SNP | 2B | 37.55 | 0.05 | 3.16 |
| 2015 | GFR | 1115679_SNP | 2B | 37.55 | 0.05 | 3.08 |
| 2013 | DM_M_ | 1066619_PAV | 2B | 37.61 | 0.03 | 3.34 |
| 2013 | RUE_AM_ | 1066619_PAV | 2B | 37.61 | 0.03 | 3.01 |
| 2013 | DM_M_ | 3944857_PAV | 2B | 40.46 | 0.05 | 4.45 |
| 2013 | RUE_AM_ | 3944857_PAV | 2B | 40.46 | 0.04 | 3.63 |
| 2013 | DM_M_ | 3948003_PAV | 2B | 40.46 | 0.05 | 4.45 |
| 2013 | RUE_AM_ | 3948003_PAV | 2B | 40.46 | 0.04 | 3.63 |
| 2013 | DM_M_ | 4410212_PAV | 2B | 40.46 | 0.04 | 3.50 |
| 2013 | RUE_AM_ | 4410212_PAV | 2B | 40.46 | 0.03 | 3.08 |
| 2014 | cPAR_AM_ | 1254442_PAV | 2B | 43.8 | 0.04 | 3.58 |
| 2014 | D_HA_ | 4406239_PAV | 2B | 44.08 | 0.03 | 3.16 |
| 2014 | D_HA_ | 4408311_PAV | 2B | 44.08 | 0.03 | 3.09 |
| 2014 | D_HA_ | 4409782_PAV | 2B | 44.08 | 0.03 | 3.09 |
| 2014 | D_HA_ | 4410188_PAV | 2B | 44.08 | 0.03 | 3.09 |
| 2015 | HI | 994059_PAV | 2B | 48.35 | 0.04 | 4.20 |
| 2014 | PH | 994059_PAV | 2B | 48.35 | 0.03 | 3.27 |
| 2014 | cPAR_AM_ | 1255293_PAV | 2B | 49.31 | 0.03 | 3.02 |
| 2014 | cPAR_AM_ | 2258320_PAV | 2B | 49.31 | 0.04 | 3.38 |
| 2014 | cPAR_AM_ | 2292730_PAV | 2B | 49.31 | 0.03 | 3.30 |
| 2014 | cPAR_AM_ | 3022079_PAV | 2B | 49.31 | 0.03 | 3.03 |
| 2014 | cPAR_AM_ | 3022763_PAV | 2B | 49.31 | 0.04 | 3.48 |
| 2014 | cPAR_AM_ | 3025672_PAV | 2B | 49.31 | 0.03 | 3.27 |
| 2014 | cPAR_AM_ | 3025766_PAV | 2B | 49.31 | 0.03 | 3.06 |
| 2014 | cPAR_AM_ | 3953184_PAV | 2B | 49.31 | 0.03 | 3.19 |
| 2014 | cPAR_AM_ | 4004509_PAV | 2B | 49.31 | 0.04 | 3.55 |
| 2014 | cPAR_AM_ | 4398305_PAV | 2B | 49.31 | 0.04 | 3.52 |
| 2014 | cPAR_AM_ | 4404543_PAV | 2B | 49.31 | 0.03 | 3.02 |
| 2014 | cPAR_AM_ | 985214_PAV | 2B | 49.31 | 0.04 | 3.46 |
| 2014 | cPAR_AM_ | 1049675_PAV | 2B | 49.59 | 0.03 | 3.32 |
| 2014 | cPAR_AM_ | 1103012_PAV | 2B | 49.59 | 0.03 | 3.13 |
| 2014 | cPAR_AM_ | 1103827_PAV | 2B | 49.59 | 0.03 | 3.10 |
| 2014 | cPAR_AM_ | 1111225_PAV | 2B | 49.59 | 0.04 | 3.60 |
| 2014 | cPAR_AM_ | 1117897_PAV | 2B | 49.59 | 0.04 | 3.63 |
| 2014 | cPAR_AM_ | 1126937_PAV | 2B | 49.59 | 0.04 | 3.56 |
| 2013 | W | 1204183_SNP | 2B | 49.59 | 0.04 | 3.16 |
| 2014 | cPAR_AM_ | 1216744_PAV | 2B | 49.59 | 0.04 | 3.31 |
| 2014 | cPAR_AM_ | 998387_PAV | 2B | 49.59 | 0.04 | 4.02 |
| 2014 | DM_M_ | 998387_PAV | 2B | 49.59 | 0.03 | 3.11 |
| 2014 | cPAR_AM_ | 1237184_PAV | 2B | 49.74 | 0.03 | 3.28 |
| 2014 | cPAR_AM_ | 2276270_PAV | 2B | 49.89 | 0.04 | 3.35 |
| 2014 | DM_M_ | 2277515_PAV | 2B | 49.89 | 0.04 | 3.53 |
| 2014 | cPAR_AM_ | 2277515_PAV | 2B | 49.89 | 0.04 | 3.36 |
| 2014 | cPAR_AM_ | 3028308_PAV | 2B | 49.89 | 0.04 | 3.52 |
| 2014 | DM_M_ | 3028308_PAV | 2B | 49.89 | 0.03 | 3.07 |
| 2014 | cPAR_AM_ | 4003771_PAV | 2B | 49.89 | 0.03 | 3.13 |
| 2014 | cPAR_AM_ | 4404376_PAV | 2B | 49.94 | 0.04 | 3.80 |
| 2014 | cPAR_AM_ | 4407354_PAV | 2B | 49.94 | 0.04 | 3.80 |
| 2014 | cPAR_AM_ | 3947942_PAV | 2B | 50.07 | 0.04 | 3.37 |
| 2014 | cPAR_AM_ | 1013114_PAV | 2B | 50.2 | 0.04 | 3.65 |
| 2014 | cPAR_AM_ | 2276848_PAV | 2B | 52.14 | 0.05 | 4.13 |
| 2014 | cPAR_AM_ | 1221101_PAV | 2B | 52.86 | 0.03 | 3.15 |
| 2014 | D_AM_ | 1056644_PAV | 2B | 54.33 | 0.03 | 3.04 |
| 2014 | CTD_Mi_ | 1208611_SNP | 2B | 54.33 | 0.05 | 3.65 |
| 2014 | cPAR_AM_ | 977989_PAV | 2B | 54.33 | 0.04 | 3.68 |
| 2014 | cPAR_AM_ | 1210486_SNP | 2B | 55.09 | 0.04 | 3.29 |
| 2015 | cPAR_EH_ | 1103326_PAV | 2B | 57.38 | 0.03 | 3.50 |
| 2015 | D_EH_ | 4404339_PAV | 2B | 58.37 | 0.00 | 3.12 |
| 2015 | D_EH_ | 4409764_PAV | 2B | 58.37 | 0.00 | 3.12 |
| 2014 | HI | 1052289_PAV | 2B | 59.6 | 0.04 | 3.84 |
| 2014 | HI | 1319458_PAV | 2B | 59.6 | 0.03 | 3.41 |
| 2014 | cPAR_EH_ | 4397540_PAV | 2B | 60.08 | 0.03 | 3.45 |
| 2014 | cPAR_AM_ | 2366453_PAV | 2B | 60.85 | 0.03 | 3.12 |
| 2014 | D_AM_ | 4407375_PAV | 2B | 62.95 | 0.04 | 3.95 |
| 2014 | cPAR_AM_ | 4407375_PAV | 2B | 62.95 | 0.04 | 3.39 |
| 2014 | D_AM_ | 4409589_PAV | 2B | 62.95 | 0.04 | 3.95 |
| 2014 | cPAR_AM_ | 4409589_PAV | 2B | 62.95 | 0.04 | 3.39 |
| 2014 | GFR | 2303461_PAV | 2B | 63.41 | 0.03 | 3.04 |
| 2014 | GFR | 3938783_PAV | 2B | 63.41 | 0.03 | 3.32 |
| 2015 | DM_A_ | 4406929_PAV | 2B | 63.41 | 0.04 | 3.34 |
| 2013 | cPAR_EH_ | 4406727_PAV | 2B | 63.46 | 0.03 | 3.20 |
| 2013 | RUE_AM_ | 1115571_PAV | 2B | 63.73 | 0.03 | 3.13 |
| 2013 | W | 4405658_PAV | 2B | 65.75 | 0.06 | 5.17 |
| 2013 | GFR | 4405658_PAV | 2B | 65.75 | 0.05 | 4.54 |
| 2015 | W | 4405658_PAV | 2B | 65.75 | 0.04 | 3.81 |
| 2015 | GFR | 4405658_PAV | 2B | 65.75 | 0.04 | 3.66 |
| 2013 | W | 4409205_PAV | 2B | 65.75 | 0.06 | 5.17 |
| 2013 | GFR | 4409205_PAV | 2B | 65.75 | 0.05 | 4.54 |
| 2015 | W | 4409205_PAV | 2B | 65.75 | 0.04 | 3.81 |
| 2015 | GFR | 4409205_PAV | 2B | 65.75 | 0.04 | 3.66 |
| 2014 | Yield | 1065080_PAV | 2B | 66.52 | 0.03 | 3.34 |
| 2013 | RUE_EA_ | 1055145_PAV | 2B | 68.25 | 0.04 | 3.40 |
| 2013 | D_HA_ | 2279916_SNP | 2B | 69.03 | 0.04 | 4.05 |
| 2013 | cPAR_HA_ | 2279916_SNP | 2B | 69.03 | 0.04 | 3.53 |
| 2013 | D_EH_ | 3940384_PAV | 2B | 69.53 | 0.01 | 3.15 |
| 2013 | DM_A_ | 1023993_SNP | 2B | 70.11 | 0.05 | 3.96 |
| 2014 | Yield | 4407393_PAV | 2B | 70.99 | 0.03 | 3.07 |
| 2014 | NGS | 981336_SNP | 2B | 71.77 | 0.07 | 5.09 |
| 2014 | DM_A_ | 1151413_PAV | 2B | 72.01 | 0.03 | 3.07 |
| 2014 | DM_A_ | 1767690_PAV | 2B | 72.26 | 0.04 | 4.17 |
| 2014 | DM_M_ | 4009949_PAV | 2B | 72.98 | 0.03 | 3.26 |
| 2014 | RUE_AM_ | 1116943_PAV | 2B | 73.02 | 0.03 | 3.03 |
| 2013 | NS | 4406296_PAV | 2B | 73.23 | 0.04 | 3.71 |
| 2013 | D_AM_ | 4394034_SNP | 2B | 73.43 | 0.04 | 3.29 |
| 2015 | HI | 4404537_PAV | 2B | 73.5 | 0.03 | 3.17 |
| 2015 | HI | 4410657_PAV | 2B | 73.5 | 0.03 | 3.17 |
| 2015 | PL | 1221313_SNP | 2B | 73.8 | 0.04 | 3.18 |
| 2013 | D_EH_ | 4004281_PAV | 2B | 73.91 | 0.01 | 3.37 |
| 2015 | D_AM_ | 1114208_PAV | 2B | 75.09 | 0.03 | 3.54 |
| 2014 | PH | 1114208_PAV | 2B | 75.09 | 0.03 | 3.18 |
| 2014 | cPAR_AM_ | 1221359_PAV | 2B | 75.09 | 0.04 | 3.67 |
| 2014 | CTD_Mi_ | 4010065_PAV | 2B | 75.09 | 0.05 | 4.27 |
| 2013 | NS | 4010065_PAV | 2B | 75.09 | 0.03 | 3.40 |
| 2015 | DM_A_ | 1059326_SNP | 2B | 75.11 | 0.06 | 3.91 |
| 2013 | PH | 1081466_SNP | 2B | 75.13 | 0.09 | 6.24 |
| 2013 | PL | 1081466_SNP | 2B | 75.13 | 0.06 | 4.12 |
| 2015 | PL | 1081466_SNP | 2B | 75.13 | 0.05 | 3.34 |
| 2015 | PH | 1081466_SNP | 2B | 75.13 | 0.05 | 3.19 |
| 2015 | NS | 1141542_PAV | 2B | 75.13 | 0.04 | 3.60 |
| 2013 | NS | 4410129_PAV | 2B | 75.9 | 0.04 | 3.71 |
| 2013 | NS | 4410163_PAV | 2B | 75.9 | 0.04 | 3.71 |
| 2013 | NS | 4410262_PAV | 2B | 75.9 | 0.04 | 3.71 |
| 2014 | GFR | 2281496_SNP | 2B | 75.94 | 0.04 | 3.05 |
| 2013 | NGS | 3939199_PAV | 2B | 76.02 | 0.04 | 3.58 |
| 2014 | NGS | 1694745_SNP | 2B | 76.17 | 0.04 | 3.00 |
| 2013 | cPAR_AM_ | 1115164_PAV | 2B | 76.69 | 0.04 | 3.58 |
| 2014 | D_AM_ | 3958899_PAV | 2B | 76.69 | 0.03 | 3.05 |
| 2013 | D_HA_ | 3958899_PAV | 2B | 76.69 | 0.02 | 3.03 |
| 2013 | cPAR_AM_ | 4008692_PAV | 2B | 76.69 | 0.03 | 3.25 |
| 2014 | D_EH_ | 4008692_PAV | 2B | 76.69 | 0.00 | 3.09 |
| 2013 | D_EH_ | 1116423_PAV | 2B | 77.19 | 0.01 | 3.09 |
| 2015 | DM_M_ | 1170459_PAV | 2B | 77.19 | 0.03 | 3.22 |
| 2014 | NGS | 2275728_PAV | 2B | 77.19 | 0.03 | 3.13 |
| 2015 | cPAR_HA_ | 1387071_PAV | 2B | 77.77 | 0.03 | 3.30 |
| 2013 | DM_A_ | 988074_SNP | 2B | 78.04 | 0.04 | 3.71 |
| 2014 | W | 3959914_PAV | 2B | 78.17 | 0.04 | 3.26 |
| 2015 | PH | 1114138_SNP | 2B | 78.31 | 0.06 | 4.19 |
| 2013 | NGS | 1204842_SNP | 2B | 78.31 | 0.05 | 3.48 |
| 2013 | D_EH_ | 4411733_PAV | 2B | 78.71 | 0.01 | 3.11 |
| 2013 | D_EH_ | 4406927_PAV | 2B | 78.74 | 0.01 | 3.11 |
| 2015 | W | 3942732_SNP | 2B | 78.87 | 0.05 | 3.65 |
| 2015 | GFR | 3942732_SNP | 2B | 78.87 | 0.04 | 3.50 |
| 2013 | cPAR_AM_ | 1075921_SNP | 2B | 79.08 | 0.05 | 4.21 |
| 2015 | W | 3958748_PAV | 2B | 79.92 | 0.05 | 4.37 |
| 2015 | GFR | 3958748_PAV | 2B | 79.92 | 0.04 | 3.55 |
| 2015 | W | 4404928_PAV | 2B | 79.92 | 0.04 | 4.03 |
| 2015 | GFR | 4404928_PAV | 2B | 79.92 | 0.03 | 3.18 |
| 2013 | PH | 3938087_PAV | 2B | 80.44 | 0.03 | 3.29 |
| 2013 | NGS | 3064387_SNP | 2B | 80.62 | 0.04 | 3.04 |
| 2014 | cPAR_AM_ | 1064899_PAV | 2B | 80.82 | 0.04 | 3.98 |
| 2013 | cPAR_AM_ | 1113038_PAV | 2B | 82.14 | 0.04 | 3.31 |
| 2014 | CTD_A_ | 3533636_PAV | 2B | 82.2 | 0.03 | 3.05 |
| 2014 | D_EH_ | 1134469_PAV | 2B | 82.4 | 0.00 | 4.29 |
| 2015 | PL | 1125933_SNP | 2B | 82.99 | 0.05 | 3.59 |
| 2013 | cPAR_AM_ | 2276054_PAV | 2B | 83.14 | 0.04 | 3.52 |
| 2013 | cPAR_AM_ | 2259692_SNP | 2B | 83.2 | 0.03 | 3.04 |
| 2014 | PH | 3961374_PAV | 2B | 83.28 | 0.03 | 3.19 |
| 2015 | GA_90d_ | 1125589_SNP | 2B | 83.54 | 0.04 | 3.14 |
| 2014 | cPAR_AM_ | 1252182_PAV | 2B | 83.82 | 0.04 | 4.16 |
| 2015 | D_AM_ | 3956975_PAV | 2B | 83.84 | 0.03 | 3.59 |
| 2013 | NS | 2278575_SNP | 2B | 83.87 | 0.04 | 3.29 |
| 2013 | Yield | 2257357_SNP | 2B | 83.92 | 0.06 | 3.59 |
| 2015 | D_EH_ | 3021181_PAV | 2B | 84.57 | 0.00 | 3.50 |
| 2014 | Yield | 2291195_PAV | 2B | 84.76 | 0.03 | 3.06 |
| 2015 | D_AM_ | 3935851_PAV | 2B | 85.08 | 0.02 | 3.06 |
| 2015 | D_AM_ | 3958645_PAV | 2B | 85.08 | 0.02 | 3.06 |
| 2015 | GA_90d_ | 1102666_PAV | 2B | 85.94 | 0.03 | 3.01 |
| 2014 | Yield | 3026115_PAV | 2B | 85.94 | 0.03 | 3.05 |
| 2013 | cPAR_EH_ | 3027068_PAV | 2B | 85.94 | 0.03 | 3.59 |
| 2014 | Yield | 4008982_PAV | 2B | 85.94 | 0.03 | 3.13 |
| 2014 | Yield | 4405730_PAV | 2B | 85.94 | 0.03 | 3.24 |
| 2014 | DM_M_ | 1198924_PAV | 2B | 86.51 | 0.04 | 3.75 |
| 2014 | RUE_AM_ | 1198924_PAV | 2B | 86.51 | 0.03 | 3.37 |
| 2013 | PL | 4409161_PAV | 2B | 86.99 | 0.03 | 3.02 |
| 2014 | Yield | 3944820_PAV | 2B | 87.85 | 0.03 | 3.01 |
| 2014 | DM_M_ | 3958206_PAV | 2B | 87.9 | 0.03 | 3.43 |
| 2014 | RUE_AM_ | 3958206_PAV | 2B | 87.9 | 0.03 | 3.13 |
| 2013 | PH | 3956384_PAV | 2B | 88.23 | 0.03 | 3.13 |
| 2015 | DM_M_ | 1145489_PAV | 2B | 88.44 | 0.03 | 3.11 |
| 2015 | DM_A_ | 1101902_PAV | 2B | 88.76 | 0.03 | 3.02 |
| 2015 | D_AM_ | 1139954_PAV | 2B | 88.84 | 0.02 | 3.20 |
| 2013 | cPAR_AM_ | 1130495_SNP | 2B | 88.87 | 0.04 | 3.30 |
| 2015 | NS | 1228146_PAV | 2B | 90.58 | 0.03 | 3.00 |
| 2014 | D_EH_ | 1100081_PAV | 2B | 94.68 | 0.00 | 3.45 |
| 2013 | PH | 1100081_PAV | 2B | 94.68 | 0.03 | 3.12 |
| 2015 | PL | 3957520_PAV | 2B | 94.83 | 0.04 | 3.55 |
| 2013 | GA_90d_ | 4404714_PAV | 2B | 104.91 | 0.03 | 3.12 |
| 2015 | NS | 1037077_SNP | 2B | 105.08 | 0.05 | 4.24 |
| 2014 | Yield | 1105968_SNP | 2B | 105.81 | 0.04 | 3.21 |
| 2014 | PL | 4407563_PAV | 2B | 105.81 | 0.04 | 3.53 |
| 2015 | cPAR_AM_ | 4004581_PAV | 2B | 106.22 | 0.03 | 3.09 |
| 2015 | cPAR_AM_ | 3935489_PAV | 2B | 106.27 | 0.04 | 3.64 |
| 2015 | DM_A_ | 1215290_PAV | 2B | 106.66 | 0.04 | 3.93 |
| 2014 | W | 1026348_PAV | 2B | 106.72 | 0.03 | 3.12 |
| 2014 | cPAR_AM_ | 1022883_PAV | 2B | 106.78 | 0.03 | 3.19 |
| 2014 | HI | 2277250_PAV | 2B | 106.86 | 0.03 | 3.13 |
| 2015 | CTD_A_ | 3939961_PAV | 2B | 106.98 | 0.04 | 3.73 |
| 2015 | D_EH_ | 4411482_PAV | 2B | 107 | 0.00 | 3.09 |
| 2013 | cPAR_EH_ | 1107610_PAV | 2B | 107.01 | 0.03 | 3.26 |
| 2013 | DM_M_ | 1107610_PAV | 2B | 107.01 | 0.03 | 3.16 |
| 2015 | cPAR_AM_ | 1318953_PAV | 2B | 107.01 | 0.04 | 3.91 |
| 2013 | PH | 3385353_PAV | 2B | 107.01 | 0.03 | 3.20 |
| 2013 | DM_M_ | 1036842_SNP | 2B | 107.03 | 0.04 | 3.07 |
| 2015 | cPAR_AM_ | 1136326_PAV | 2B | 107.03 | 0.04 | 3.65 |
| 2015 | DM_M_ | 1695461_PAV | 2B | 107.03 | 0.03 | 3.01 |
| 2015 | cPAR_EH_ | 976862_PAV | 2B | 107.03 | 0.03 | 3.17 |
| 2015 | GA_90d_ | 1084992_PAV | 2B | 107.75 | 0.03 | 3.11 |
| 2015 | Yield | 1157809_PAV | 2B | 109.09 | 0.04 | 3.38 |
| 2015 | DM_A_ | 4406483_PAV | 3A | 0.77 | 0.04 | 3.16 |
| 2014 | PL | 1156116_PAV | 3A | 3.58 | 0.06 | 4.99 |
| 2015 | D_HA_ | 2277193_SNP | 3A | 4.26 | 0.03 | 3.03 |
| 2013 | cPAR_AM_ | 4406668_PAV | 3A | 4.29 | 0.03 | 3.09 |
| 2014 | PL | 3384982_PAV | 3A | 4.59 | 0.06 | 5.09 |
| 2014 | PH | 3384982_PAV | 3A | 4.59 | 0.03 | 3.16 |
| 2013 | PH | 1092360_PAV | 3A | 4.62 | 0.03 | 3.26 |
| 2013 | NGS | 3064482_SNP | 3A | 4.63 | 0.05 | 4.02 |
| 2013 | GA_90d_ | 1106789_PAV | 3A | 4.65 | 0.04 | 3.40 |
| 2013 | PL | 4406539_PAV | 3A | 5.01 | 0.03 | 3.19 |
| 2013 | PL | 4407465_PAV | 3A | 5.01 | 0.03 | 3.19 |
| 2013 | PL | 4408994_PAV | 3A | 5.01 | 0.03 | 3.19 |
| 2013 | PL | 4410263_PAV | 3A | 5.01 | 0.03 | 3.19 |
| 2015 | D_EH_ | 3024772_SNP | 3A | 5.13 | 0.01 | 3.71 |
| 2015 | PL | 1100100_PAV | 3A | 8.9 | 0.05 | 4.57 |
| 2014 | CTD_Mi_ | 3064416_SNP | 3A | 8.9 | 0.05 | 3.39 |
| 2015 | PL | 1127382_SNP | 3A | 8.93 | 0.05 | 3.27 |
| 2014 | D_EH_ | 1094951_PAV | 3A | 13.11 | 0.01 | 4.78 |
| 2013 | W | 1210343_SNP | 3A | 13.11 | 0.04 | 3.11 |
| 2015 | NGS | 1767669_PAV | 3A | 13.2 | 0.03 | 3.21 |
| 2013 | PH | 1092230_PAV | 3A | 17.78 | 0.03 | 3.19 |
| 2014 | D_EH_ | 3029273_PAV | 3A | 18.52 | 0.01 | 4.27 |
| 2014 | CTD_Mi_ | 2276764_SNP | 3A | 20.28 | 0.07 | 4.32 |
| 2014 | D_EH_ | 2276764_SNP | 3A | 20.28 | 0.01 | 3.79 |
| 2013 | RUE_AM_ | 1115098_PAV | 3A | 23.16 | 0.04 | 3.55 |
| 2015 | PL | 1130605_PAV | 3A | 30.03 | 0.03 | 3.01 |
| 2013 | PL | 1164647_SNP | 3A | 30.03 | 0.05 | 3.83 |
| 2013 | DM_M_ | 1252770_SNP | 3A | 30.03 | 0.06 | 4.15 |
| 2013 | RUE_AM_ | 1252770_SNP | 3A | 30.03 | 0.05 | 3.50 |
| 2013 | NS | 1064832_PAV | 3A | 30.89 | 0.04 | 3.58 |
| 2013 | DM_M_ | 3021072_PAV | 3A | 30.89 | 0.04 | 3.94 |
| 2013 | RUE_AM_ | 3021072_PAV | 3A | 30.89 | 0.04 | 3.40 |
| 2013 | NS | 3022891_PAV | 3A | 30.89 | 0.04 | 3.25 |
| 2013 | DM_M_ | 3024251_PAV | 3A | 30.89 | 0.03 | 3.26 |
| 2013 | RUE_AM_ | 3024251_PAV | 3A | 30.89 | 0.03 | 3.02 |
| 2013 | PH | 4412072_PAV | 3A | 34.06 | 0.03 | 3.35 |
| 2013 | PH | 4412160_PAV | 3A | 34.06 | 0.03 | 3.35 |
| 2013 | PH | 1862879_SNP | 3A | 34.14 | 0.04 | 3.00 |
| 2013 | PH | 1025974_SNP | 3A | 36.24 | 0.04 | 4.13 |
| 2013 | PL | 1025974_SNP | 3A | 36.24 | 0.04 | 3.39 |
| 2013 | NGS | 1058280_PAV | 3A | 40.62 | 0.03 | 3.09 |
| 2015 | GA_90d_ | 1133972_PAV | 3A | 41.22 | 0.04 | 3.56 |
| 2015 | GA_90d_ | 1240205_PAV | 3A | 41.22 | 0.03 | 3.08 |
| 2014 | W | 2320230_PAV | 3A | 41.22 | 0.03 | 3.34 |
| 2013 | RUE_AM_ | 1090721_SNP | 3A | 46.75 | 0.04 | 3.06 |
| 2013 | HI | 1139799_SNP | 3A | 47.15 | 0.04 | 3.29 |
| 2014 | PH | 3064511_SNP | 3A | 48.17 | 0.07 | 4.12 |
| 2015 | PL | 3064511_SNP | 3A | 48.17 | 0.07 | 3.77 |
| 2015 | PH | 3064511_SNP | 3A | 48.17 | 0.06 | 3.57 |
| 2015 | PL | 3947101_PAV | 3A | 48.22 | 0.05 | 4.69 |
| 2015 | PH | 3947101_PAV | 3A | 48.22 | 0.03 | 3.08 |
| 2015 | PL | 3952904_PAV | 3A | 48.22 | 0.05 | 4.69 |
| 2015 | PH | 3952904_PAV | 3A | 48.22 | 0.03 | 3.08 |
| 2015 | D_HA_ | 1012487_SNP | 3A | 49.47 | 0.05 | 3.20 |
| 2013 | W | 1674219_PAV | 3A | 50.55 | 0.04 | 3.69 |
| 2013 | GFR | 1674219_PAV | 3A | 50.55 | 0.04 | 3.57 |
| 2013 | DM_M_ | 1220348_PAV | 3A | 51.77 | 0.04 | 3.39 |
| 2013 | RUE_AM_ | 1220348_PAV | 3A | 51.77 | 0.03 | 3.06 |
| 2015 | CTD_A_ | 3572862_PAV | 3A | 60.7 | 0.03 | 3.32 |
| 2013 | W | 3572862_PAV | 3A | 60.7 | 0.03 | 3.01 |
| 2014 | CTD_Mi_ | 1862576_SNP | 3A | 61.76 | 0.05 | 3.50 |
| 2013 | NGS | 2278246_SNP | 3A | 61.93 | 0.05 | 3.95 |
| 2014 | W | 1112863_SNP | 3A | 66.27 | 0.05 | 3.22 |
| 2014 | D_AM_ | 3949752_PAV | 3A | 66.57 | 0.04 | 3.50 |
| 2013 | D_HA_ | 1231255_PAV | 3A | 70.94 | 0.03 | 3.50 |
| 2013 | HI | 1153026_PAV | 3A | 72.37 | 0.03 | 3.18 |
| 2015 | RUE_EA_ | 3939308_PAV | 3A | 76.02 | 0.05 | 4.48 |
| 2014 | cPAR_EH_ | 1246633_PAV | 3A | 100.56 | 0.02 | 3.06 |
| 2015 | cPAR_EH_ | 1092726_SNP | 3A | 103.87 | 0.04 | 3.26 |
| 2015 | cPAR_EH_ | 1104893_SNP | 3A | 103.87 | 0.04 | 3.05 |
| 2015 | cPAR_EH_ | 1128265_SNP | 3A | 103.87 | 0.04 | 3.24 |
| 2015 | D_HA_ | 1258373_SNP | 3A | 106.87 | 0.04 | 3.07 |
| 2013 | PH | 1056379_SNP | 3A | 109.55 | 0.04 | 3.03 |
| 2015 | cPAR_AM_ | 4002919_PAV | 3A | 113.23 | 0.04 | 3.45 |
| 2015 | cPAR_EH_ | 2276788_PAV | 3A | 114.88 | 0.03 | 3.22 |
| 2014 | cPAR_HA_ | 1064764_SNP | 3A | 116.68 | 0.04 | 3.68 |
| 2014 | cPAR_HA_ | 1254900_PAV | 3A | 116.7 | 0.04 | 4.03 |
| 2015 | D_EH_ | 1262637_PAV | 3A | 118.46 | 0.00 | 3.20 |
| 2014 | CTD_A_ | 4410575_PAV | 3A | 125.21 | 0.04 | 4.16 |
| 2014 | W | 1092778_SNP | 3A | 126.45 | 0.04 | 3.02 |
| 2015 | PH | 1047772_PAV | 3A | 128.91 | 0.05 | 4.30 |
| 2015 | PL | 1047772_PAV | 3A | 128.91 | 0.05 | 4.01 |
| 2014 | PH | 1047772_PAV | 3A | 128.91 | 0.04 | 3.51 |
| 2015 | HI | 1047772_PAV | 3A | 128.91 | 0.03 | 3.43 |
| 2013 | Yield | 2265707_SNP | 3A | 133.67 | 0.04 | 3.24 |
| 2015 | D_EH_ | 1115450_PAV | 3A | 134.03 | 0.00 | 3.23 |
| 2015 | PH | 3955023_PAV | 3A | 134.03 | 0.03 | 3.00 |
| 2015 | PH | 980006_PAV | 3A | 137.71 | 0.03 | 3.55 |
| 2015 | GA_90d_ | 1109210_SNP | 3A | 144.85 | 0.04 | 3.40 |
| 2015 | PL | 1196031_SNP | 3A | 146.99 | 0.07 | 4.04 |
| 2014 | RUE_EA_ | 1118743_PAV | 3B | 3.48 | 0.03 | 3.35 |
| 2013 | GA_90d_ | 1118743_PAV | 3B | 3.48 | 0.03 | 3.03 |
| 2013 | D_AM_ | 1227454_PAV | 3B | 4.67 | 0.02 | 3.21 |
| 2015 | CTD_Mi_ | 4408654_PAV | 3B | 5.02 | 0.03 | 3.08 |
| 2015 | W | 1210301_PAV | 3B | 5.83 | 0.03 | 3.25 |
| 2014 | D_EH_ | 1211784_SNP | 3B | 8.48 | 0.00 | 3.25 |
| 2015 | GFR | 1261750_PAV | 3B | 8.9 | 0.03 | 3.50 |
| 2015 | W | 1261750_PAV | 3B | 8.9 | 0.03 | 3.43 |
| 2014 | cPAR_EH_ | 1213490_PAV | 3B | 9.02 | 0.02 | 3.21 |
| 2014 | RUE_EA_ | 1213490_PAV | 3B | 9.02 | 0.03 | 3.14 |
| 2015 | GA_90d_ | 3956601_PAV | 3B | 9.02 | 0.04 | 3.44 |
| 2013 | cPAR_AM_ | 4407645_PAV | 3B | 9.02 | 0.03 | 3.09 |
| 2013 | cPAR_AM_ | 4412154_PAV | 3B | 9.02 | 0.03 | 3.09 |
| 2015 | CTD_Mi_ | 3941921_PAV | 3B | 9.3 | 0.04 | 3.41 |
| 2015 | CTD_Mi_ | 3954483_PAV | 3B | 9.3 | 0.04 | 3.41 |
| 2014 | CTD_A_ | 1725192_SNP | 3B | 10.45 | 0.05 | 3.34 |
| 2013 | PL | 1767951_PAV | 3B | 11.56 | 0.03 | 3.25 |
| 2015 | DM_A_ | 1029913_PAV | 3B | 12.26 | 0.03 | 3.16 |
| 2015 | DM_A_ | 1343568_PAV | 3B | 12.37 | 0.05 | 3.86 |
| 2015 | DM_A_ | 1256609_PAV | 3B | 12.5 | 0.05 | 3.78 |
| 2013 | DM_M_ | 1157327_PAV | 3B | 12.62 | 0.03 | 3.39 |
| 2013 | DM_M_ | 1317305_PAV | 3B | 12.62 | 0.03 | 3.16 |
| 2013 | DM_M_ | 1862944_SNP | 3B | 12.62 | 0.05 | 3.64 |
| 2013 | RUE_AM_ | 1862944_SNP | 3B | 12.62 | 0.05 | 3.37 |
| 2014 | CTD_Mi_ | 1764978_SNP | 3B | 12.84 | 0.05 | 3.16 |
| 2013 | PL | 3956358_SNP | 3B | 13.38 | 0.05 | 3.65 |
| 2015 | GA_90d_ | 1216424_SNP | 3B | 16.86 | 0.05 | 3.28 |
| 2014 | GA_90d_ | 1380660_SNP | 3B | 17.37 | 0.05 | 3.58 |
| 2015 | PH | 1218956_SNP | 3B | 17.45 | 0.04 | 3.01 |
| 2013 | cPAR_AM_ | 3946590_PAV | 3B | 17.5 | 0.04 | 4.03 |
| 2014 | HI | 3946590_PAV | 3B | 17.5 | 0.03 | 3.11 |
| 2015 | GA_90d_ | 4004943_PAV | 3B | 17.58 | 0.03 | 3.09 |
| 2015 | GA_90d_ | 1140649_PAV | 3B | 17.82 | 0.04 | 3.41 |
| 2015 | GA_90d_ | 1268683_PAV | 3B | 17.82 | 0.04 | 4.07 |
| 2015 | DM_A_ | 1268386_SNP | 3B | 20.97 | 0.05 | 3.68 |
| 2015 | DM_A_ | 3934055_PAV | 3B | 21.64 | 0.04 | 3.54 |
| 2015 | RUE_EA_ | 3934055_PAV | 3B | 21.64 | 0.03 | 3.38 |
| 2015 | PH | 1039056_SNP | 3B | 23.59 | 0.05 | 3.82 |
| 2013 | CTD_A_ | 1234768_SNP | 3B | 24.86 | 0.05 | 3.16 |
| 2014 | NGS | 997369_PAV | 3B | 26.46 | 0.04 | 3.59 |
| 2013 | D_AM_ | 4262108_PAV | 3B | 26.79 | 0.03 | 3.25 |
| 2014 | CTD_Mi_ | 1102608_SNP | 3B | 28.17 | 0.05 | 3.30 |
| 2013 | DM_A_ | 4407876_PAV | 3B | 29.63 | 0.03 | 3.07 |
| 2013 | DM_A_ | 1078880_PAV | 3B | 29.75 | 0.03 | 3.14 |
| 2013 | DM_A_ | 1220223_PAV | 3B | 29.75 | 0.04 | 3.75 |
| 2014 | CTD_Mi_ | 2277821_SNP | 3B | 30.85 | 0.05 | 3.00 |
| 2013 | DM_A_ | 1101758_PAV | 3B | 32.46 | 0.03 | 3.19 |
| 2013 | DM_A_ | 2290379_PAV | 3B | 32.8 | 0.03 | 3.42 |
| 2015 | NS | 3029174_SNP | 3B | 35.54 | 0.05 | 3.87 |
| 2014 | Yield | 3029174_SNP | 3B | 35.54 | 0.04 | 3.46 |
| 2015 | HI | 1006009_PAV | 3B | 38 | 0.03 | 3.46 |
| 2014 | D_EH_ | 4405765_PAV | 3B | 41.92 | 0.00 | 3.34 |
| 2014 | PH | 1157912_SNP | 3B | 49.58 | 0.04 | 3.00 |
| 2013 | HI | 997119_SNP | 3B | 50.81 | 0.03 | 3.11 |
| 2014 | CTD_Mi_ | 3533521_PAV | 3B | 52.58 | 0.05 | 4.79 |
| 2013 | W | 1093129_SNP | 3B | 52.87 | 0.04 | 3.20 |
| 2015 | PH | 3946129_PAV | 3B | 53.13 | 0.03 | 3.00 |
| 2014 | CTD_Mi_ | 2258010_SNP | 3B | 53.31 | 0.04 | 3.10 |
| 2015 | cPAR_AM_ | 1146830_SNP | 3B | 53.89 | 0.05 | 3.52 |
| 2015 | NS | 1089859_PAV | 3B | 55.56 | 0.03 | 3.12 |
| 2013 | RUE_AM_ | 1083901_SNP | 3B | 56.13 | 0.05 | 4.06 |
| 2013 | NS | 1083901_SNP | 3B | 56.13 | 0.04 | 3.08 |
| 2013 | cPAR_EH_ | 1267475_PAV | 3B | 57.69 | 0.03 | 3.31 |
| 2014 | cPAR_AM_ | 3955435_PAV | 3B | 57.72 | 0.03 | 3.14 |
| 2014 | GFR | 3947989_PAV | 3B | 59.07 | 0.03 | 3.06 |
| 2014 | NGS | 2257120_SNP | 3B | 59.48 | 0.03 | 3.13 |
| 2015 | D_AM_ | 3949052_PAV | 3B | 60.53 | 0.05 | 5.34 |
| 2013 | DM_M_ | 1245174_PAV | 3B | 61 | 0.04 | 3.48 |
| 2013 | DM_A_ | 1120417_SNP | 3B | 61.8 | 0.04 | 3.06 |
| 2015 | cPAR_AM_ | 1133353_PAV | 3B | 61.89 | 0.03 | 3.22 |
| 2015 | cPAR_HA_ | 1410611_PAV | 3B | 64.05 | 0.04 | 3.78 |
| 2015 | D_HA_ | 1410611_PAV | 3B | 64.05 | 0.03 | 3.05 |
| 2013 | DM_M_ | 1410611_PAV | 3B | 64.05 | 0.03 | 3.00 |
| 2013 | DM_M_ | 1132326_SNP | 3B | 64.74 | 0.05 | 3.49 |
| 2013 | GFR | 1234572_PAV | 3B | 65.49 | 0.04 | 3.53 |
| 2013 | DM_M_ | 1201639_SNP | 3B | 65.59 | 0.05 | 3.95 |
| 2013 | NGS | 1201639_SNP | 3B | 65.59 | 0.04 | 3.31 |
| 2013 | RUE_AM_ | 1201639_SNP | 3B | 65.59 | 0.04 | 3.27 |
| 2013 | PH | 1863248_SNP | 3B | 66.63 | 0.04 | 3.95 |
| 2013 | NGS | 1047201_SNP | 3B | 68.36 | 0.04 | 3.35 |
| 2013 | NGS | 1091190_SNP | 3B | 68.36 | 0.05 | 3.35 |
| 2013 | Yield | 1109783_SNP | 3B | 68.36 | 0.05 | 3.48 |
| 2014 | W | 1055089_PAV | 3B | 68.64 | 0.03 | 3.09 |
| 2015 | DM_A_ | 4408340_PAV | 3B | 77.76 | 0.04 | 3.16 |
| 2014 | cPAR_EH_ | 4408243_PAV | 3B | 79.02 | 0.03 | 3.58 |
| 2014 | D_HA_ | 4404639_PAV | 3B | 79.91 | 0.03 | 3.03 |
| 2015 | GA_90d_ | 4003819_PAV | 3B | 80.69 | 0.04 | 3.97 |
| 2013 | CTD_A_ | 1028447_SNP | 3B | 82.11 | 0.06 | 4.06 |
| 2014 | D_HA_ | 3064935_SNP | 3B | 82.11 | 0.05 | 4.83 |
| 2013 | PL | 3938395_SNP | 3B | 82.85 | 0.05 | 3.85 |
| 2013 | Yield | 3938395_SNP | 3B | 82.85 | 0.04 | 3.37 |
| 2013 | NGS | 1109056_SNP | 3B | 83.45 | 0.05 | 3.94 |
| 2014 | GFR | 1039845_SNP | 3B | 84.64 | 0.05 | 4.17 |
| 2014 | cPAR_AM_ | 3533926_SNP | 3B | 90.55 | 0.04 | 3.04 |
| 2013 | cPAR_AM_ | 1087906_PAV | 3B | 91.62 | 0.03 | 3.06 |
| 2014 | D_EH_ | 4405300_PAV | 3B | 93.72 | 0.00 | 3.32 |
| 2014 | D_EH_ | 4411839_PAV | 3B | 93.72 | 0.00 | 3.34 |
| 2014 | PL | 2259950_SNP | 3B | 95.18 | 0.05 | 3.40 |
| 2015 | PH | 3575558_PAV | 3B | 100.09 | 0.04 | 3.81 |
| 2015 | PL | 3575558_PAV | 3B | 100.09 | 0.03 | 3.25 |
| 2014 | cPAR_HA_ | 1130599_SNP | 3B | 100.91 | 0.05 | 3.14 |
| 2014 | D_HA_ | 1130599_SNP | 3B | 100.91 | 0.04 | 3.11 |
| 2013 | W | 1398611_PAV | 3B | 112.86 | 0.03 | 3.31 |
| 2013 | GFR | 1398611_PAV | 3B | 112.86 | 0.03 | 3.15 |
| 2013 | W | 2307278_PAV | 3B | 112.86 | 0.03 | 3.30 |
| 2013 | W | 3936927_PAV | 3B | 112.86 | 0.04 | 4.01 |
| 2013 | GFR | 3936927_PAV | 3B | 112.86 | 0.04 | 3.58 |
| 2013 | W | 3937908_PAV | 3B | 112.86 | 0.04 | 4.01 |
| 2013 | GFR | 3937908_PAV | 3B | 112.86 | 0.04 | 3.58 |
| 2013 | W | 985734_PAV | 3B | 112.86 | 0.03 | 3.25 |
| 2013 | GFR | 985734_PAV | 3B | 112.86 | 0.03 | 3.08 |
| 2013 | W | 1088503_PAV | 3B | 113.52 | 0.03 | 3.00 |
| 2013 | W | 1126376_PAV | 3B | 114.62 | 0.04 | 3.94 |
| 2013 | GFR | 1126376_PAV | 3B | 114.62 | 0.04 | 3.59 |
| 2015 | RUE_EA_ | 1128206_SNP | 3B | 114.62 | 0.04 | 3.26 |
| 2013 | W | 3532801_PAV | 3B | 115.42 | 0.03 | 3.30 |
| 2013 | CTD_Mi_ | 4408225_PAV | 3B | 115.61 | 0.04 | 3.38 |
| 2014 | GFR | 4411597_PAV | 3B | 115.61 | 0.04 | 3.36 |
| 2013 | D_AM_ | 4411597_PAV | 3B | 115.61 | 0.03 | 3.24 |
| 2014 | GFR | 4412067_PAV | 3B | 115.61 | 0.04 | 3.36 |
| 2013 | D_AM_ | 4412067_PAV | 3B | 115.61 | 0.03 | 3.24 |
| 2013 | W | 991025_SNP | 3B | 116.84 | 0.04 | 3.41 |
| 2013 | W | 1068340_PAV | 3B | 116.95 | 0.04 | 3.63 |
| 2013 | GFR | 1068340_PAV | 3B | 116.95 | 0.04 | 3.30 |
| 2014 | CTD_Mi_ | 2254375_SNP | 3B | 121.02 | 0.06 | 4.71 |
| 2014 | GFR | 1143310_PAV | 3B | 123.48 | 0.04 | 3.36 |
| 2013 | D_AM_ | 1143310_PAV | 3B | 123.48 | 0.03 | 3.35 |
| 2013 | D_AM_ | 1094389_PAV | 3B | 124.12 | 0.03 | 3.54 |
| 2014 | GFR | 1094389_PAV | 3B | 124.12 | 0.04 | 3.38 |
| 2014 | PL | 1092828_PAV | 3B | 131.91 | 0.03 | 3.13 |
| 2013 | NS | 3955568_PAV | 3B | 137.99 | 0.03 | 3.01 |
| 2015 | DM_M_ | 1022298_PAV | 3B | 138.12 | 0.04 | 3.48 |
| 2015 | NGS | 1022298_PAV | 3B | 138.12 | 0.04 | 3.42 |
| 2015 | PH | 1022298_PAV | 3B | 138.12 | 0.03 | 3.24 |
| 2015 | HI | 1271275_PAV | 3B | 138.48 | 0.03 | 3.08 |
| 2013 | Yield | 1159999_SNP | 3B | 138.97 | 0.04 | 3.00 |
| 2013 | GA_90d_ | 1378260_PAV | 3B | 139.03 | 0.04 | 3.94 |
| 2015 | CTD_Mi_ | 1113201_PAV | 3B | 156.7 | 0.04 | 3.64 |
| 2014 | D_EH_ | 3027649_PAV | 3B | 156.7 | 0.00 | 3.38 |
| 2015 | NS | 1227999_SNP | 3B | 157.06 | 0.08 | 4.30 |
| 2015 | DM_M_ | 1227999_SNP | 3B | 157.06 | 0.07 | 3.58 |
| 2014 | D_AM_ | 1341461_SNP | 4A | 5.92 | 0.06 | 4.73 |
| 2014 | PL | 1229973_SNP | 4A | 19.84 | 0.05 | 3.87 |
| 2014 | PH | 1229973_SNP | 4A | 19.84 | 0.04 | 3.19 |
| 2013 | D_EH_ | 2288897_PAV | 4A | 19.84 | 0.01 | 3.03 |
| 2013 | D_EH_ | 2320838_PAV | 4A | 19.84 | 0.01 | 3.07 |
| 2013 | CTD_Mi_ | 1116119_PAV | 4A | 20.92 | 0.04 | 3.76 |
| 2014 | D_AM_ | 1272673_PAV | 4A | 21.81 | 0.05 | 4.15 |
| 2013 | D_EH_ | 3021742_PAV | 4A | 21.99 | 0.01 | 3.86 |
| 2013 | RUE_AM_ | 982577_SNP | 4A | 24.14 | 0.06 | 4.89 |
| 2013 | DM_M_ | 982577_SNP | 4A | 24.14 | 0.05 | 4.33 |
| 2014 | NS | 1719824_PAV | 4A | 24.73 | 0.03 | 3.13 |
| 2014 | cPAR_HA_ | 4405366_PAV | 4A | 24.73 | 0.03 | 3.02 |
| 2014 | PH | 994866_SNP | 4A | 25.7 | 0.04 | 3.23 |
| 2014 | NGS | 4009227_SNP | 4A | 26.03 | 0.04 | 3.12 |
| 2015 | W | 1263780_PAV | 4A | 26.08 | 0.06 | 4.78 |
| 2015 | GFR | 1263780_PAV | 4A | 26.08 | 0.05 | 4.72 |
| 2014 | W | 1263780_PAV | 4A | 26.08 | 0.05 | 4.32 |
| 2013 | W | 1263780_PAV | 4A | 26.08 | 0.04 | 3.79 |
| 2013 | GFR | 1263780_PAV | 4A | 26.08 | 0.04 | 3.39 |
| 2015 | D_HA_ | 1211780_SNP | 4A | 28.5 | 0.04 | 3.54 |
| 2013 | D_EH_ | 3030634_PAV | 4A | 28.5 | 0.01 | 3.14 |
| 2013 | D_EH_ | 3533011_PAV | 4A | 28.58 | 0.01 | 3.20 |
| 2015 | D_EH_ | 1084677_PAV | 4A | 29.14 | 0.01 | 5.03 |
| 2015 | Yield | 1396097_PAV | 4A | 29.37 | 0.03 | 3.11 |
| 2015 | NGS | 1293924_PAV | 4A | 30.81 | 0.04 | 3.45 |
| 2013 | D_EH_ | 1091178_SNP | 4A | 30.86 | 0.01 | 3.05 |
| 2013 | DM_M_ | 1076361_PAV | 4A | 46.97 | 0.03 | 3.34 |
| 2013 | RUE_AM_ | 1076361_PAV | 4A | 46.97 | 0.03 | 3.21 |
| 2015 | D_EH_ | 1129776_PAV | 4A | 47.12 | 0.01 | 5.36 |
| 2015 | GA_90d_ | 1091267_SNP | 4A | 57.47 | 0.04 | 3.14 |
| 2013 | D_EH_ | 1219379_PAV | 4A | 66.76 | 0.01 | 3.47 |
| 2013 | GFR | 1122769_PAV | 4A | 68.4 | 0.03 | 3.05 |
| 2013 | CTD_Mi_ | 1089707_PAV | 4A | 70.37 | 0.03 | 3.26 |
| 2013 | CTD_Mi_ | 994022_SNP | 4A | 70.37 | 0.04 | 3.20 |
| 2013 | DM_M_ | 1131791_SNP | 4A | 71.35 | 0.04 | 3.07 |
| 2013 | D_HA_ | 2254324_PAV | 4A | 72.6 | 0.03 | 3.61 |
| 2013 | NGS | 1015566_SNP | 4A | 85.41 | 0.07 | 5.68 |
| 2015 | PL | 3025184_PAV | 4A | 94.29 | 0.03 | 3.07 |
| 2015 | GA_90d_ | 1108372_PAV | 4A | 96.08 | 0.04 | 3.36 |
| 2013 | D_HA_ | 1128664_SNP | 4A | 96.08 | 0.04 | 3.12 |
| 2013 | DM_M_ | 1663802_PAV | 4A | 96.08 | 0.03 | 3.15 |
| 2015 | GFR | 4009056_PAV | 4A | 96.24 | 0.03 | 3.10 |
| 2015 | cPAR_HA_ | 3954323_PAV | 4A | 96.32 | 0.03 | 3.13 |
| 2015 | GA_90d_ | 1091143_PAV | 4A | 96.66 | 0.03 | 3.09 |
| 2015 | CTD_A_ | 4008221_PAV | 4A | 97.15 | 0.03 | 3.03 |
| 2013 | RUE_EA_ | 1005860_SNP | 4A | 103.69 | 0.05 | 3.71 |
| 2015 | D_AM_ | 3955368_SNP | 4A | 109.69 | 0.04 | 3.95 |
| 2015 | GFR | 3533037_PAV | 4A | 114.8 | 0.03 | 3.43 |
| 2015 | W | 3533037_PAV | 4A | 114.8 | 0.03 | 3.40 |
| 2014 | CTD_Mi_ | 1109730_SNP | 4A | 116.03 | 0.08 | 6.06 |
| 2015 | GFR | 1109730_SNP | 4A | 116.03 | 0.04 | 3.22 |
| 2013 | D_EH_ | 4005137_PAV | 4A | 116.17 | 0.01 | 4.06 |
| 2013 | D_HA_ | 4005137_PAV | 4A | 116.17 | 0.03 | 3.51 |
| 2014 | CTD_Mi_ | 1119457_SNP | 4A | 119.06 | 0.05 | 4.09 |
| 2013 | D_EH_ | 1330639_PAV | 4A | 122.06 | 0.01 | 3.00 |
| 2014 | D_EH_ | 1118128_PAV | 4A | 123.16 | 0.00 | 3.07 |
| 2015 | PL | 1235602_PAV | 4A | 123.16 | 0.03 | 3.13 |
| 2013 | PL | 3064477_SNP | 4A | 124.01 | 0.04 | 3.05 |
| 2015 | PL | 2276111_PAV | 4A | 124.37 | 0.04 | 3.53 |
| 2015 | PL | 1108449_PAV | 4A | 129.17 | 0.04 | 3.24 |
| 2014 | NS | 1268778_SNP | 4A | 129.7 | 0.05 | 3.04 |
| 2014 | DM_M_ | 1699386_PAV | 4A | 130.14 | 0.03 | 3.17 |
| 2014 | NS | 1117122_SNP | 4A | 130.21 | 0.05 | 3.02 |
| 2014 | NS | 1305526_PAV | 4A | 131.69 | 0.04 | 3.84 |
| 2013 | D_AM_ | 2275222_SNP | 4A | 132.76 | 0.03 | 3.09 |
| 2014 | GA_90d_ | 2277496_PAV | 4A | 134.5 | 0.03 | 3.21 |
| 2015 | PH | 4008687_PAV | 4B | 2.79 | 0.03 | 3.08 |
| 2015 | PH | 1121395_PAV | 4B | 17.44 | 0.06 | 5.67 |
| 2015 | PL | 1121395_PAV | 4B | 17.44 | 0.03 | 3.26 |
| 2014 | PH | 1121395_PAV | 4B | 17.44 | 0.03 | 3.09 |
| 2015 | PH | 1114067_PAV | 4B | 18.57 | 0.04 | 4.12 |
| 2015 | PH | 1267366_PAV | 4B | 18.57 | 0.05 | 4.65 |
| 2015 | PH | 1070513_PAV | 4B | 19.76 | 0.04 | 3.95 |
| 2013 | W | 998452_SNP | 4B | 19.76 | 0.05 | 3.43 |
| 2015 | PH | 1110644_PAV | 4B | 21.97 | 0.03 | 3.38 |
| 2015 | PH | 1686542_PAV | 4B | 23.86 | 0.03 | 3.29 |
| 2013 | cPAR_AM_ | 1048997_PAV | 4B | 31.24 | 0.04 | 3.79 |
| 2014 | PH | 1003062_SNP | 4B | 31.36 | 0.05 | 3.79 |
| 2013 | CTD_A_ | 1068469_SNP | 4B | 31.36 | 0.04 | 3.25 |
| 2013 | cPAR_AM_ | 980754_SNP | 4B | 31.36 | 0.05 | 3.02 |
| 2014 | cPAR_HA_ | 1409297_PAV | 4B | 31.47 | 0.03 | 3.14 |
| 2014 | Yield | 1282222_PAV | 4B | 32.58 | 0.03 | 3.17 |
| 2013 | NS | 1697863_PAV | 4B | 32.85 | 0.03 | 3.04 |
| 2014 | PH | 1671044_SNP | 4B | 33.01 | 0.04 | 3.65 |
| 2015 | GFR | 1235666_SNP | 4B | 34.46 | 0.04 | 3.19 |
| 2015 | CTD_Mi_ | 991821_SNP | 4B | 34.91 | 0.04 | 3.30 |
| 2015 | GFR | 1076176_PAV | 4B | 35.75 | 0.03 | 3.21 |
| 2015 | CTD_A_ | 1098002_PAV | 4B | 39.44 | 0.03 | 3.39 |
| 2015 | W | 1089380_SNP | 4B | 40.08 | 0.04 | 3.29 |
| 2015 | D_EH_ | 3935024_PAV | 4B | 41.97 | 0.00 | 3.14 |
| 2013 | HI | 1206598_PAV | 4B | 43.33 | 0.03 | 3.25 |
| 2015 | PH | 4407824_PAV | 4B | 44.26 | 0.03 | 3.07 |
| 2015 | PH | 4408111_PAV | 4B | 44.26 | 0.03 | 3.07 |
| 2015 | RUE_AM_ | 3022798_PAV | 4B | 47.48 | 0.03 | 3.09 |
| 2015 | D_HA_ | 1127388_SNP | 4B | 48.27 | 0.05 | 3.08 |
| 2015 | DM_M_ | 1122500_SNP | 4B | 49.62 | 0.05 | 3.54 |
| 2015 | RUE_AM_ | 1122500_SNP | 4B | 49.62 | 0.05 | 3.54 |
| 2015 | PH | 3025153_SNP | 4B | 52.42 | 0.04 | 3.54 |
| 2014 | PH | 3025153_SNP | 4B | 52.42 | 0.04 | 3.17 |
| 2015 | W | 3024231_PAV | 4B | 55.53 | 0.03 | 3.11 |
| 2013 | NGS | 3222471_SNP | 4B | 69.77 | 0.05 | 3.54 |
| 2013 | NGS | 1213366_PAV | 4B | 74.86 | 0.04 | 3.58 |
| 2015 | D_HA_ | 3941944_PAV | 4B | 75.67 | 0.04 | 3.60 |
| 2015 | D_HA_ | 3946905_PAV | 4B | 75.67 | 0.04 | 3.60 |
| 2015 | D_HA_ | 3951054_PAV | 4B | 75.67 | 0.04 | 3.60 |
| 2015 | PH | 1406855_PAV | 4B | 76.03 | 0.04 | 3.85 |
| 2013 | Yield | 1260853_PAV | 4B | 77.89 | 0.03 | 3.01 |
| 2013 | RUE_EA_ | 3942633_PAV | 5A | 8.82 | 0.04 | 3.46 |
| 2013 | W | 4410940_PAV | 5A | 8.82 | 0.04 | 3.41 |
| 2013 | GFR | 4410940_PAV | 5A | 8.82 | 0.03 | 3.20 |
| 2013 | PH | 1207000_SNP | 5A | 17.79 | 0.06 | 4.80 |
| 2013 | PL | 1207000_SNP | 5A | 17.79 | 0.04 | 3.40 |
| 2014 | cPAR_EH_ | 1402738_PAV | 5A | 18.2 | 0.03 | 3.82 |
| 2014 | cPAR_EH_ | 1009544_PAV | 5A | 20.01 | 0.02 | 3.26 |
| 2013 | DM_A_ | 4405813_PAV | 5A | 23.66 | 0.03 | 3.15 |
| 2013 | DM_A_ | 4407023_PAV | 5A | 23.66 | 0.03 | 3.15 |
| 2013 | DM_A_ | 1314856_PAV | 5A | 31.16 | 0.04 | 3.70 |
| 2014 | cPAR_HA_ | 1092381_SNP | 5A | 34.73 | 0.04 | 3.48 |
| 2014 | D_HA_ | 1092381_SNP | 5A | 34.73 | 0.03 | 3.48 |
| 2014 | GA_90d_ | 4411365_PAV | 5A | 35.22 | 0.03 | 3.16 |
| 2014 | GA_90d_ | 4412110_PAV | 5A | 35.22 | 0.03 | 3.16 |
| 2014 | GA_90d_ | 995872_SNP | 5A | 35.22 | 0.04 | 3.28 |
| 2014 | GA_90d_ | 1298772_PAV | 5A | 35.92 | 0.04 | 3.35 |
| 2014 | PH | 1103927_PAV | 5A | 36.95 | 0.03 | 3.33 |
| 2015 | D_EH_ | 4260954_SNP | 5A | 39.34 | 0.01 | 4.68 |
| 2013 | cPAR_EH_ | 4260954_SNP | 5A | 39.34 | 0.04 | 3.75 |
| 2014 | D_EH_ | 4260954_SNP | 5A | 39.34 | 0.01 | 3.69 |
| 2015 | HI | 4260954_SNP | 5A | 39.34 | 0.04 | 3.11 |
| 2015 | NS | 1126029_PAV | 5A | 43.37 | 0.04 | 3.90 |
| 2014 | PH | 1035170_SNP | 5A | 48.57 | 0.04 | 3.24 |
| 2014 | GA_90d_ | 980383_SNP | 5A | 48.57 | 0.04 | 3.54 |
| 2015 | cPAR_EH_ | 980597_PAV | 5A | 48.57 | 0.04 | 3.91 |
| 2015 | RUE_EA_ | 980597_PAV | 5A | 48.57 | 0.03 | 3.28 |
| 2013 | GA_90d_ | 4394579_PAV | 5A | 48.58 | 0.04 | 4.00 |
| 2013 | GA_90d_ | 992066_SNP | 5A | 48.58 | 0.04 | 3.05 |
| 2014 | PH | 1088744_PAV | 5A | 50.28 | 0.03 | 3.36 |
| 2014 | GFR | 3384945_PAV | 5A | 50.28 | 0.04 | 3.70 |
| 2014 | PH | 3384945_PAV | 5A | 50.28 | 0.03 | 3.54 |
| 2013 | GFR | 3384945_PAV | 5A | 50.28 | 0.04 | 3.30 |
| 2014 | PH | 1127707_SNP | 5A | 52.63 | 0.04 | 3.43 |
| 2015 | DM_M_ | 3945966_PAV | 5A | 53.65 | 0.04 | 3.38 |
| 2014 | DM_A_ | 1269476_PAV | 5A | 55.38 | 0.04 | 3.38 |
| 2013 | NS | 1265387_PAV | 5A | 58.39 | 0.04 | 3.78 |
| 2015 | D_AM_ | 1242564_SNP | 5A | 58.49 | 0.04 | 4.69 |
| 2015 | GFR | 1242564_SNP | 5A | 58.49 | 0.04 | 3.11 |
| 2013 | NGS | 3020494_PAV | 5A | 59.47 | 0.04 | 3.12 |
| 2013 | Yield | 3948413_PAV | 5A | 59.47 | 0.04 | 3.85 |
| 2015 | Yield | 3948413_PAV | 5A | 59.47 | 0.03 | 3.06 |
| 2015 | D_AM_ | 4003847_PAV | 5A | 59.47 | 0.02 | 3.19 |
| 2013 | NGS | 997467_SNP | 5A | 59.47 | 0.04 | 3.43 |
| 2013 | NGS | 1132848_PAV | 5A | 59.65 | 0.04 | 3.32 |
| 2013 | NGS | 3951746_PAV | 5A | 59.65 | 0.04 | 3.67 |
| 2015 | D_AM_ | 1063558_PAV | 5A | 60.39 | 0.02 | 3.03 |
| 2013 | Yield | 3571376_PAV | 5A | 60.98 | 0.04 | 3.77 |
| 2015 | D_AM_ | 1013062_PAV | 5A | 61.27 | 0.03 | 3.98 |
| 2013 | DM_M_ | 1090060_PAV | 5A | 61.27 | 0.03 | 3.17 |
| 2013 | NGS | 1218906_SNP | 5A | 61.27 | 0.06 | 4.90 |
| 2015 | DM_A_ | 1274441_PAV | 5A | 63.62 | 0.04 | 3.27 |
| 2014 | HI | 1108690_SNP | 5A | 65.91 | 0.05 | 3.02 |
| 2014 | PH | 1092507_SNP | 5A | 70.74 | 0.04 | 3.33 |
| 2014 | CTD_Mi_ | 1098012_PAV | 5A | 77.31 | 0.04 | 3.65 |
| 2013 | W | 4410488_PAV | 5A | 79.61 | 0.06 | 5.06 |
| 2013 | GFR | 4410488_PAV | 5A | 79.61 | 0.05 | 4.63 |
| 2015 | DM_A_ | 988625_SNP | 5A | 80.03 | 0.05 | 3.32 |
| 2014 | CTD_Mi_ | 994009_SNP | 5A | 80.32 | 0.06 | 4.51 |
| 2015 | PL | 994009_SNP | 5A | 80.32 | 0.04 | 3.44 |
| 2013 | DM_M_ | 993328_SNP | 5A | 80.76 | 0.04 | 3.09 |
| 2015 | CTD_A_ | 1109903_SNP | 5A | 81.53 | 0.04 | 3.08 |
| 2014 | CTD_Mi_ | 1220783_SNP | 5A | 81.58 | 0.06 | 4.21 |
| 2014 | PL | 3020998_PAV | 5A | 82.69 | 0.05 | 4.04 |
| 2015 | GFR | 3020998_PAV | 5A | 82.69 | 0.04 | 3.61 |
| 2015 | PL | 3020998_PAV | 5A | 82.69 | 0.04 | 3.36 |
| 2015 | PH | 3020998_PAV | 5A | 82.69 | 0.03 | 3.06 |
| 2015 | GA_90d_ | 1137911_SNP | 5A | 84.41 | 0.03 | 3.25 |
| 2014 | D_AM_ | 3940216_PAV | 5A | 84.51 | 0.03 | 3.09 |
| 2014 | D_AM_ | 3944867_PAV | 5A | 84.51 | 0.03 | 3.09 |
| 2014 | DM_M_ | 1089167_PAV | 5A | 84.94 | 0.03 | 3.18 |
| 2013 | GA_90d_ | 3222345_SNP | 5A | 86.69 | 0.07 | 5.15 |
| 2014 | D_EH_ | 3222345_SNP | 5A | 86.69 | 0.01 | 5.13 |
| 2015 | D_EH_ | 3222345_SNP | 5A | 86.69 | 0.01 | 3.56 |
| 2015 | CTD_A_ | 1215828_PAV | 5A | 89.64 | 0.03 | 3.26 |
| 2015 | cPAR_AM_ | 1215828_PAV | 5A | 89.64 | 0.03 | 3.10 |
| 2013 | NGS | 2277206_SNP | 5A | 89.79 | 0.04 | 3.19 |
| 2013 | RUE_AM_ | 3935199_PAV | 5A | 106.35 | 0.05 | 4.43 |
| 2013 | DM_M_ | 3935199_PAV | 5A | 106.35 | 0.04 | 3.46 |
| 2013 | RUE_AM_ | 3949660_PAV | 5A | 106.35 | 0.05 | 4.43 |
| 2013 | DM_M_ | 3949660_PAV | 5A | 106.35 | 0.04 | 3.46 |
| 2013 | RUE_AM_ | 3952123_PAV | 5A | 106.35 | 0.05 | 4.43 |
| 2013 | DM_M_ | 3952123_PAV | 5A | 106.35 | 0.04 | 3.46 |
| 2013 | RUE_AM_ | 1103307_PAV | 5A | 107.01 | 0.04 | 3.44 |
| 2013 | RUE_AM_ | 1259252_PAV | 5A | 107.01 | 0.03 | 3.22 |
| 2014 | DM_M_ | 2251786_PAV | 5A | 108.16 | 0.04 | 3.44 |
| 2014 | RUE_AM_ | 2251786_PAV | 5A | 108.16 | 0.04 | 3.21 |
| 2015 | PH | 1144964_SNP | 5A | 108.68 | 0.05 | 3.84 |
| 2015 | D_HA_ | 1234670_PAV | 5A | 111.35 | 0.03 | 3.50 |
| 2014 | W | 1724148_PAV | 5A | 112.36 | 0.03 | 3.28 |
| 2014 | RUE_EA_ | 1205387_PAV | 5A | 122.51 | 0.03 | 3.66 |
| 2013 | GA_90d_ | 1205387_PAV | 5A | 122.51 | 0.04 | 3.39 |
| 2014 | cPAR_EH_ | 1205387_PAV | 5A | 122.51 | 0.02 | 3.15 |
| 2013 | GA_90d_ | 1232988_PAV | 5A | 122.51 | 0.04 | 3.50 |
| 2013 | D_EH_ | 4008602_PAV | 5A | 123.79 | 0.01 | 3.47 |
| 2015 | D_EH_ | 4008602_PAV | 5A | 123.79 | 0.00 | 3.03 |
| 2014 | cPAR_EH_ | 1269445_PAV | 5A | 134.35 | 0.03 | 3.49 |
| 2014 | RUE_EA_ | 1269445_PAV | 5A | 134.35 | 0.03 | 3.46 |
| 2014 | D_AM_ | 1077619_SNP | 5A | 143.87 | 0.05 | 3.74 |
| 2013 | RUE_AM_ | 1139898_PAV | 5A | 148.79 | 0.03 | 3.13 |
| 2014 | CTD_Mi_ | 3028544_SNP | 5A | 148.79 | 0.04 | 3.29 |
| 2013 | RUE_AM_ | 1251421_PAV | 5A | 150.26 | 0.03 | 3.02 |
| 2013 | RUE_AM_ | 995984_PAV | 5A | 150.56 | 0.03 | 3.12 |
| 2014 | CTD_Mi_ | 1092232_PAV | 5A | 153.71 | 0.03 | 3.14 |
| 2013 | RUE_AM_ | 1721373_SNP | 5A | 153.71 | 0.04 | 3.08 |
| 2014 | NS | 2260159_SNP | 5A | 156.04 | 0.05 | 3.46 |
| 2014 | HI | 1067819_PAV | 5B | 0 | 0.03 | 3.16 |
| 2013 | RUE_AM_ | 1214483_SNP | 5B | 0 | 0.04 | 3.41 |
| 2013 | RUE_AM_ | 1229405_SNP | 5B | 0 | 0.05 | 3.66 |
| 2013 | NS | 1229405_SNP | 5B | 0 | 0.04 | 3.07 |
| 2013 | RUE_AM_ | 2277516_SNP | 5B | 0 | 0.05 | 4.23 |
| 2013 | DM_M_ | 2277516_SNP | 5B | 0 | 0.04 | 3.09 |
| 2013 | RUE_AM_ | 3028819_PAV | 5B | 0 | 0.03 | 3.16 |
| 2013 | RUE_AM_ | 3064734_SNP | 5B | 0 | 0.05 | 4.18 |
| 2013 | DM_M_ | 3064734_SNP | 5B | 0 | 0.05 | 3.78 |
| 2014 | GFR | 1104807_PAV | 5B | 8.45 | 0.03 | 3.21 |
| 2013 | D_EH_ | 1125300_SNP | 5B | 8.45 | 0.01 | 3.11 |
| 2013 | D_AM_ | 1091392_PAV | 5B | 9.14 | 0.03 | 3.39 |
| 2014 | GFR | 3028036_PAV | 5B | 9.67 | 0.03 | 3.04 |
| 2013 | D_AM_ | 3533645_PAV | 5B | 9.7 | 0.02 | 3.13 |
| 2013 | D_AM_ | 1090520_PAV | 5B | 10.86 | 0.03 | 3.44 |
| 2013 | D_AM_ | 1103298_PAV | 5B | 10.86 | 0.03 | 3.60 |
| 2014 | GFR | 1102288_PAV | 5B | 11.74 | 0.04 | 3.66 |
| 2015 | NS | 2278231_PAV | 5B | 12.14 | 0.03 | 3.05 |
| 2013 | D_AM_ | 1020131_SNP | 5B | 13.11 | 0.03 | 3.44 |
| 2013 | D_AM_ | 3945574_PAV | 5B | 13.24 | 0.02 | 3.13 |
| 2013 | D_AM_ | 4262418_PAV | 5B | 13.24 | 0.03 | 3.44 |
| 2015 | D_HA_ | 1246640_SNP | 5B | 13.28 | 0.04 | 3.42 |
| 2013 | D_AM_ | 2255194_PAV | 5B | 13.35 | 0.03 | 3.51 |
| 2013 | D_AM_ | 1090880_PAV | 5B | 13.41 | 0.03 | 4.23 |
| 2013 | D_AM_ | 1094462_PAV | 5B | 13.41 | 0.03 | 3.90 |
| 2013 | D_AM_ | 1118696_PAV | 5B | 14.29 | 0.03 | 3.21 |
| 2015 | NS | 1696830_SNP | 5B | 15.76 | 0.04 | 3.22 |
| 2013 | CTD_A_ | 2324828_PAV | 5B | 16.1 | 0.04 | 3.35 |
| 2013 | CTD_A_ | 1293393_PAV | 5B | 16.39 | 0.04 | 3.16 |
| 2013 | CTD_A_ | 4404624_PAV | 5B | 16.39 | 0.03 | 3.04 |
| 2015 | NS | 3955690_SNP | 5B | 27.12 | 0.09 | 7.13 |
| 2015 | DM_M_ | 3955690_SNP | 5B | 27.12 | 0.04 | 3.34 |
| 2015 | DM_A_ | 1089527_PAV | 5B | 28.48 | 0.06 | 4.75 |
| 2015 | cPAR_EH_ | 1089527_PAV | 5B | 28.48 | 0.03 | 3.16 |
| 2013 | GFR | 3945391_PAV | 5B | 29.08 | 0.03 | 3.12 |
| 2015 | CTD_A_ | 1153393_PAV | 5B | 30.26 | 0.03 | 3.49 |
| 2015 | Yield | 1153393_PAV | 5B | 30.26 | 0.03 | 3.01 |
| 2015 | CTD_A_ | 4018723_PAV | 5B | 30.26 | 0.04 | 3.52 |
| 2015 | CTD_A_ | 4407674_PAV | 5B | 30.26 | 0.03 | 3.21 |
| 2013 | GFR | 1025445_SNP | 5B | 30.31 | 0.04 | 3.16 |
| 2015 | CTD_A_ | 1134768_PAV | 5B | 31.82 | 0.03 | 3.08 |
| 2013 | NGS | 1275356_SNP | 5B | 31.82 | 0.04 | 3.44 |
| 2013 | NS | 1057114_PAV | 5B | 32.51 | 0.03 | 3.00 |
| 2014 | CTD_Mi_ | 1008590_SNP | 5B | 32.89 | 0.07 | 5.33 |
| 2013 | DM_M_ | 1129672_SNP | 5B | 33.3 | 0.05 | 3.05 |
| 2013 | NGS | 1225296_SNP | 5B | 33.47 | 0.04 | 3.10 |
| 2015 | D_HA_ | 1126050_SNP | 5B | 33.99 | 0.04 | 3.64 |
| 2013 | DM_M_ | 1005692_SNP | 5B | 35.2 | 0.05 | 3.70 |
| 2013 | RUE_AM_ | 1005692_SNP | 5B | 35.2 | 0.04 | 3.37 |
| 2014 | PH | 2263452_SNP | 5B | 35.36 | 0.05 | 3.61 |
| 2015 | cPAR_EH_ | 1105113_PAV | 5B | 35.4 | 0.04 | 3.65 |
| 2014 | cPAR_AM_ | 1105113_PAV | 5B | 35.4 | 0.03 | 3.21 |
| 2014 | cPAR_EH_ | 1105113_PAV | 5B | 35.4 | 0.02 | 3.03 |
| 2015 | PL | 1006147_PAV | 5B | 36.58 | 0.04 | 3.57 |
| 2015 | cPAR_EH_ | 1109364_PAV | 5B | 36.78 | 0.03 | 3.24 |
| 2014 | cPAR_EH_ | 1109364_PAV | 5B | 36.78 | 0.03 | 3.23 |
| 2013 | PH | 1109364_PAV | 5B | 36.78 | 0.03 | 3.02 |
| 2013 | D_HA_ | 3022870_PAV | 5B | 36.78 | 0.05 | 5.07 |
| 2013 | D_EH_ | 3022870_PAV | 5B | 36.78 | 0.01 | 3.26 |
| 2014 | NGS | 1092792_PAV | 5B | 38.78 | 0.04 | 3.52 |
| 2013 | D_HA_ | 4405894_PAV | 5B | 40.83 | 0.04 | 4.27 |
| 2014 | W | 1073735_PAV | 5B | 42.06 | 0.04 | 3.42 |
| 2015 | PH | 1094310_PAV | 5B | 42.14 | 0.04 | 3.50 |
| 2015 | NGS | 1129028_PAV | 5B | 42.96 | 0.03 | 3.03 |
| 2013 | CTD_A_ | 1235095_SNP | 5B | 44.76 | 0.06 | 3.70 |
| 2013 | CTD_A_ | 1371874_SNP | 5B | 44.76 | 0.05 | 3.32 |
| 2013 | CTD_A_ | 1685308_PAV | 5B | 44.76 | 0.04 | 3.65 |
| 2015 | cPAR_EH_ | 1062670_PAV | 5B | 46.52 | 0.03 | 3.09 |
| 2015 | D_EH_ | 2278009_SNP | 5B | 46.67 | 0.01 | 4.41 |
| 2013 | D_EH_ | 2278009_SNP | 5B | 46.67 | 0.01 | 4.00 |
| 2015 | cPAR_EH_ | 1236452_PAV | 5B | 47.6 | 0.04 | 3.80 |
| 2013 | RUE_AM_ | 3937758_PAV | 5B | 50.75 | 0.03 | 3.27 |
| 2015 | D_HA_ | 1860715_PAV | 5B | 51.06 | 0.04 | 3.78 |
| 2015 | D_HA_ | 995689_SNP | 5B | 51.88 | 0.04 | 3.12 |
| 2013 | CTD_A_ | 3022221_PAV | 5B | 52.71 | 0.03 | 3.10 |
| 2015 | D_HA_ | 3025480_SNP | 5B | 52.73 | 0.04 | 3.34 |
| 2015 | D_HA_ | 3384712_PAV | 5B | 52.73 | 0.03 | 3.66 |
| 2013 | NGS | 1094714_SNP | 5B | 52.91 | 0.05 | 3.46 |
| 2015 | cPAR_EH_ | 1108663_PAV | 5B | 52.91 | 0.03 | 3.73 |
| 2013 | PH | 1125934_SNP | 5B | 53.36 | 0.05 | 3.42 |
| 2015 | DM_A_ | 1100149_PAV | 5B | 53.72 | 0.03 | 3.20 |
| 2015 | HI | 1352957_SNP | 5B | 54.59 | 0.04 | 3.72 |
| 2014 | DM_M_ | 4409834_PAV | 5B | 54.76 | 0.04 | 3.37 |
| 2014 | NS | 4409834_PAV | 5B | 54.76 | 0.03 | 3.19 |
| 2015 | cPAR_EH_ | 4008439_PAV | 5B | 55.03 | 0.03 | 3.54 |
| 2015 | DM_A_ | 4405526_PAV | 5B | 55.06 | 0.04 | 3.69 |
| 2015 | cPAR_EH_ | 4405526_PAV | 5B | 55.06 | 0.03 | 3.04 |
| 2015 | DM_A_ | 4410721_PAV | 5B | 55.06 | 0.04 | 3.69 |
| 2015 | cPAR_EH_ | 4410721_PAV | 5B | 55.06 | 0.03 | 3.04 |
| 2014 | CTD_Mi_ | 2264373_SNP | 5B | 55.11 | 0.05 | 3.01 |
| 2015 | HI | 1125397_SNP | 5B | 55.31 | 0.04 | 3.34 |
| 2013 | RUE_EA_ | 980333_SNP | 5B | 55.73 | 0.04 | 3.04 |
| 2015 | cPAR_EH_ | 4410049_PAV | 5B | 56.28 | 0.03 | 3.41 |
| 2014 | D_EH_ | 1126675_SNP | 5B | 56.29 | 0.01 | 4.93 |
| 2015 | D_EH_ | 1126675_SNP | 5B | 56.29 | 0.01 | 3.81 |
| 2015 | cPAR_EH_ | 1089250_PAV | 5B | 57.24 | 0.03 | 3.44 |
| 2015 | cPAR_EH_ | 1304582_PAV | 5B | 57.24 | 0.03 | 3.16 |
| 2013 | NGS | 1217751_SNP | 5B | 61.69 | 0.05 | 4.15 |
| 2014 | PL | 1217751_SNP | 5B | 61.69 | 0.05 | 3.97 |
| 2015 | PL | 1217751_SNP | 5B | 61.69 | 0.04 | 3.49 |
| 2015 | CTD_A_ | 3935803_PAV | 5B | 62.76 | 0.04 | 3.29 |
| 2013 | cPAR_HA_ | 4410667_PAV | 5B | 63.81 | 0.04 | 3.38 |
| 2015 | CTD_Mi_ | 1299721_PAV | 5B | 65.98 | 0.03 | 3.00 |
| 2013 | D_AM_ | 1094418_PAV | 5B | 66.06 | 0.03 | 3.24 |
| 2014 | cPAR_HA_ | 1012260_PAV | 5B | 66.08 | 0.03 | 3.25 |
| 2015 | cPAR_EH_ | 988452_PAV | 5B | 66.67 | 0.03 | 3.54 |
| 2014 | DM_A_ | 1028758_SNP | 5B | 68.79 | 0.05 | 3.12 |
| 2014 | PH | 4407561_PAV | 5B | 71.45 | 0.03 | 3.17 |
| 2014 | PH | 4408002_PAV | 5B | 71.45 | 0.03 | 3.17 |
| 2015 | PH | 1092150_PAV | 5B | 73.2 | 0.03 | 3.16 |
| 2015 | HI | 1034210_SNP | 5B | 74.48 | 0.03 | 3.08 |
| 2013 | DM_M_ | 1163437_SNP | 5B | 75.21 | 0.05 | 3.37 |
| 2015 | cPAR_EH_ | 1218118_SNP | 5B | 76.01 | 0.04 | 3.20 |
| 2013 | NGS | 979900_SNP | 5B | 78.83 | 0.04 | 3.05 |
| 2013 | PL | 3029177_SNP | 5B | 79.49 | 0.04 | 3.18 |
| 2014 | cPAR_EH_ | 4003142_PAV | 5B | 80.08 | 0.03 | 3.46 |
| 2014 | RUE_EA_ | 4003142_PAV | 5B | 80.08 | 0.03 | 3.40 |
| 2014 | Yield | 4404893_PAV | 5B | 80.08 | 0.03 | 3.02 |
| 2013 | cPAR_HA_ | 1092044_PAV | 5B | 80.27 | 0.04 | 3.07 |
| 2014 | GA_90d_ | 978651_SNP | 5B | 83.43 | 0.07 | 5.60 |
| 2014 | cPAR_EH_ | 978651_SNP | 5B | 83.43 | 0.04 | 4.20 |
| 2014 | CTD_Mi_ | 1667742_SNP | 5B | 83.6 | 0.05 | 4.12 |
| 2014 | CTD_A_ | 2324612_PAV | 5B | 90.54 | 0.04 | 3.49 |
| 2015 | D_HA_ | 1077051_SNP | 5B | 91.15 | 0.04 | 3.44 |
| 2014 | D_EH_ | 1102099_SNP | 5B | 100.04 | 0.00 | 3.07 |
| 2013 | PL | 1206368_PAV | 5B | 104.54 | 0.03 | 3.40 |
| 2014 | GFR | 2275516_PAV | 5B | 107.67 | 0.04 | 3.42 |
| 2015 | D_EH_ | 1047463_PAV | 5B | 114.34 | 0.00 | 3.68 |
| 2015 | D_EH_ | 1219507_PAV | 5B | 114.34 | 0.00 | 3.00 |
| 2015 | D_EH_ | 3021257_PAV | 5B | 114.34 | 0.00 | 3.39 |
| 2015 | D_EH_ | 3026946_PAV | 5B | 114.34 | 0.00 | 3.18 |
| 2015 | D_EH_ | 3028199_PAV | 5B | 114.37 | 0.00 | 3.27 |
| 2015 | GA_90d_ | 1092753_PAV | 5B | 120.1 | 0.03 | 3.01 |
| 2014 | D_HA_ | 1068364_SNP | 5B | 123.85 | 0.04 | 3.84 |
| 2014 | cPAR_HA_ | 1068364_SNP | 5B | 123.85 | 0.04 | 3.42 |
| 2014 | PH | 3025227_SNP | 5B | 123.86 | 0.04 | 3.13 |
| 2013 | DM_A_ | 1054614_SNP | 5B | 130.72 | 0.05 | 3.22 |
| 2015 | PL | 1151573_SNP | 5B | 131.85 | 0.07 | 4.83 |
| 2015 | PH | 1151573_SNP | 5B | 131.85 | 0.05 | 3.28 |
| 2014 | HI | 4407167_PAV | 5B | 131.94 | 0.03 | 3.05 |
| 2014 | D_EH_ | 3026522_SNP | 5B | 132 | 0.00 | 3.41 |
| 2013 | PH | 1076157_SNP | 5B | 132.52 | 0.04 | 3.24 |
| 2014 | D_AM_ | 3064727_SNP | 5B | 136.42 | 0.04 | 3.39 |
| 2015 | CTD_A_ | 3064727_SNP | 5B | 136.42 | 0.04 | 3.06 |
| 2013 | NS | 1133198_SNP | 5B | 138.12 | 0.05 | 3.36 |
| 2015 | PL | 2289118_PAV | 5B | 139.69 | 0.04 | 3.39 |
| 2013 | DM_M_ | 2288863_PAV | 5B | 140.05 | 0.03 | 3.38 |
| 2013 | D_AM_ | 1130110_PAV | 5B | 148.54 | 0.03 | 3.17 |
| 2013 | D_AM_ | 3935557_PAV | 5B | 148.72 | 0.03 | 3.81 |
| 2013 | D_AM_ | 3936960_PAV | 5B | 148.72 | 0.03 | 3.81 |
| 2015 | Yield | 1101908_PAV | 5B | 153.35 | 0.03 | 3.13 |
| 2014 | PH | 1088758_PAV | 6A | 0 | 0.03 | 3.00 |
| 2015 | PL | 3022478_PAV | 6A | 0.63 | 0.04 | 4.08 |
| 2015 | PH | 3022478_PAV | 6A | 0.63 | 0.04 | 3.72 |
| 2014 | PH | 4408525_PAV | 6A | 7.4 | 0.04 | 3.94 |
| 2015 | PH | 4408525_PAV | 6A | 7.4 | 0.04 | 3.63 |
| 2014 | PH | 4409192_PAV | 6A | 7.4 | 0.04 | 3.94 |
| 2015 | PH | 4409192_PAV | 6A | 7.4 | 0.04 | 3.63 |
| 2015 | PH | 4410845_PAV | 6A | 7.4 | 0.04 | 3.71 |
| 2014 | PH | 4410845_PAV | 6A | 7.4 | 0.04 | 3.54 |
| 2015 | PH | 995648_SNP | 6A | 8.23 | 0.04 | 3.41 |
| 2014 | PH | 1206297_PAV | 6A | 8.31 | 0.03 | 3.24 |
| 2015 | cPAR_HA_ | 4409465_PAV | 6A | 8.58 | 0.03 | 3.01 |
| 2015 | PL | 1267066_PAV | 6A | 8.68 | 0.04 | 3.57 |
| 2015 | PH | 1267077_PAV | 6A | 8.68 | 0.03 | 3.25 |
| 2013 | DM_M_ | 1370667_PAV | 6A | 8.87 | 0.03 | 3.08 |
| 2015 | cPAR_EH_ | 2278345_PAV | 6A | 11.95 | 0.03 | 3.13 |
| 2014 | HI | 4004264_PAV | 6A | 15.6 | 0.03 | 3.59 |
| 2014 | W | 3948348_PAV | 6A | 15.77 | 0.10 | 8.11 |
| 2013 | GFR | 3948348_PAV | 6A | 15.77 | 0.07 | 5.65 |
| 2015 | W | 3948348_PAV | 6A | 15.77 | 0.06 | 5.59 |
| 2015 | GFR | 3948348_PAV | 6A | 15.77 | 0.06 | 5.38 |
| 2013 | W | 3948348_PAV | 6A | 15.77 | 0.06 | 5.35 |
| 2014 | NGS | 3948348_PAV | 6A | 15.77 | 0.06 | 5.12 |
| 2014 | GFR | 3948348_PAV | 6A | 15.77 | 0.05 | 4.40 |
| 2013 | NGS | 3948348_PAV | 6A | 15.77 | 0.04 | 3.82 |
| 2015 | NGS | 3948348_PAV | 6A | 15.77 | 0.03 | 3.11 |
| 2014 | W | 3956430_PAV | 6A | 15.77 | 0.10 | 8.11 |
| 2013 | GFR | 3956430_PAV | 6A | 15.77 | 0.07 | 5.65 |
| 2015 | W | 3956430_PAV | 6A | 15.77 | 0.06 | 5.59 |
| 2015 | GFR | 3956430_PAV | 6A | 15.77 | 0.06 | 5.38 |
| 2013 | W | 3956430_PAV | 6A | 15.77 | 0.06 | 5.35 |
| 2014 | NGS | 3956430_PAV | 6A | 15.77 | 0.06 | 5.12 |
| 2014 | GFR | 3956430_PAV | 6A | 15.77 | 0.05 | 4.40 |
| 2013 | NGS | 3956430_PAV | 6A | 15.77 | 0.04 | 3.82 |
| 2015 | NGS | 3956430_PAV | 6A | 15.77 | 0.03 | 3.11 |
| 2015 | DM_A_ | 1058681_SNP | 6A | 18.14 | 0.06 | 3.45 |
| 2015 | DM_A_ | 2281391_SNP | 6A | 23.76 | 0.05 | 3.13 |
| 2014 | Yield | 986482_PAV | 6A | 23.76 | 0.03 | 3.23 |
| 2015 | DM_M_ | 1228080_PAV | 6A | 24.91 | 0.04 | 3.65 |
| 2015 | DM_M_ | 4409825_PAV | 6A | 26.42 | 0.03 | 3.11 |
| 2013 | RUE_AM_ | 1095136_SNP | 6A | 27.09 | 0.05 | 3.67 |
| 2013 | RUE_AM_ | 981681_SNP | 6A | 27.14 | 0.06 | 4.68 |
| 2013 | DM_M_ | 981681_SNP | 6A | 27.14 | 0.04 | 3.27 |
| 2015 | PH | 1125124_PAV | 6A | 27.53 | 0.03 | 3.39 |
| 2015 | PH | 1136538_PAV | 6A | 27.53 | 0.04 | 3.66 |
| 2015 | HI | 1153837_SNP | 6A | 27.53 | 0.04 | 3.99 |
| 2013 | DM_M_ | 1234243_PAV | 6A | 27.53 | 0.04 | 3.25 |
| 2015 | PH | 1086660_PAV | 6A | 28.03 | 0.04 | 3.82 |
| 2015 | GA_90d_ | 1020590_SNP | 6A | 28.69 | 0.06 | 4.17 |
| 2013 | RUE_AM_ | 1863482_SNP | 6A | 28.69 | 0.04 | 3.42 |
| 2015 | D_EH_ | 1237792_PAV | 6A | 29.02 | 0.00 | 3.15 |
| 2014 | Yield | 1105942_PAV | 6A | 29.26 | 0.03 | 3.28 |
| 2013 | D_HA_ | 1001603_PAV | 6A | 33.23 | 0.03 | 3.93 |
| 2015 | D_AM_ | 4406881_PAV | 6A | 36.16 | 0.03 | 3.16 |
| 2015 | D_AM_ | 4408981_PAV | 6A | 36.16 | 0.03 | 3.16 |
| 2015 | D_AM_ | 3026119_PAV | 6A | 47.3 | 0.03 | 3.83 |
| 2013 | CTD_Mi_ | 2275227_PAV | 6A | 48.2 | 0.04 | 3.74 |
| 2015 | D_AM_ | 1224095_PAV | 6A | 48.23 | 0.02 | 3.08 |
| 2015 | D_AM_ | 2277488_PAV | 6A | 48.56 | 0.03 | 3.29 |
| 2015 | D_AM_ | 4404921_PAV | 6A | 48.64 | 0.03 | 3.50 |
| 2015 | NGS | 4405858_PAV | 6A | 48.64 | 0.03 | 3.08 |
| 2015 | NGS | 4406593_PAV | 6A | 48.64 | 0.03 | 3.08 |
| 2015 | D_AM_ | 4406667_PAV | 6A | 48.64 | 0.02 | 3.03 |
| 2015 | D_AM_ | 4407833_PAV | 6A | 48.64 | 0.02 | 3.03 |
| 2015 | NGS | 4409315_PAV | 6A | 48.64 | 0.03 | 3.08 |
| 2015 | D_AM_ | 4409832_PAV | 6A | 48.64 | 0.02 | 3.03 |
| 2015 | D_AM_ | 3952816_PAV | 6A | 48.7 | 0.02 | 3.11 |
| 2015 | D_AM_ | 4004467_PAV | 6A | 48.7 | 0.02 | 3.08 |
| 2013 | CTD_Mi_ | 1006957_SNP | 6A | 50.08 | 0.04 | 3.10 |
| 2015 | D_EH_ | 4408259_PAV | 6A | 50.22 | 0.00 | 3.23 |
| 2015 | D_EH_ | 4410081_PAV | 6A | 50.22 | 0.00 | 3.23 |
| 2013 | W | 4405431_PAV | 6A | 56.41 | 0.03 | 3.25 |
| 2015 | HI | 2258989_SNP | 6A | 59.47 | 0.05 | 4.39 |
| 2014 | CTD_A_ | 1237708_PAV | 6A | 67.99 | 0.04 | 4.29 |
| 2015 | NS | 1118334_PAV | 6A | 69.55 | 0.04 | 3.10 |
| 2013 | D_AM_ | 3950793_PAV | 6A | 73.16 | 0.03 | 3.48 |
| 2014 | PH | 3027755_SNP | 6A | 74.35 | 0.04 | 3.53 |
| 2015 | DM_M_ | 1126162_SNP | 6A | 79.7 | 0.04 | 3.07 |
| 2013 | NGS | 3034373_PAV | 6A | 79.7 | 0.04 | 3.90 |
| 2013 | HI | 3034373_PAV | 6A | 79.7 | 0.04 | 3.84 |
| 2013 | W | 3034373_PAV | 6A | 79.7 | 0.03 | 3.24 |
| 2013 | GFR | 3034373_PAV | 6A | 79.7 | 0.03 | 3.06 |
| 2013 | HI | 1202642_PAV | 6A | 83.23 | 0.03 | 3.10 |
| 2013 | D_AM_ | 3944725_PAV | 6A | 83.23 | 0.03 | 3.48 |
| 2013 | D_AM_ | 3958891_PAV | 6A | 83.23 | 0.03 | 3.48 |
| 2013 | W | 4408140_PAV | 6A | 85.2 | 0.04 | 3.41 |
| 2013 | GFR | 4408140_PAV | 6A | 85.2 | 0.03 | 3.20 |
| 2014 | cPAR_HA_ | 3953121_PAV | 6A | 85.22 | 0.03 | 3.26 |
| 2013 | D_AM_ | 3953896_PAV | 6A | 85.22 | 0.03 | 3.48 |
| 2015 | DM_M_ | 1016778_SNP | 6A | 86.48 | 0.05 | 4.06 |
| 2015 | NS | 1016778_SNP | 6A | 86.48 | 0.05 | 3.71 |
| 2013 | CTD_A_ | 1016778_SNP | 6A | 86.48 | 0.04 | 3.26 |
| 2013 | DM_M_ | 1105692_SNP | 6A | 86.85 | 0.04 | 3.07 |
| 2014 | CTD_Mi_ | 1218597_SNP | 6A | 87.03 | 0.04 | 3.36 |
| 2014 | CTD_Mi_ | 1082949_SNP | 6A | 87.77 | 0.04 | 3.40 |
| 2014 | cPAR_HA_ | 3028796_PAV | 6A | 88.72 | 0.03 | 3.07 |
| 2015 | D_EH_ | 4405809_PAV | 6A | 91.17 | 0.00 | 3.42 |
| 2015 | D_EH_ | 4410215_PAV | 6A | 91.17 | 0.00 | 3.42 |
| 2013 | HI | 1103064_PAV | 6A | 93.22 | 0.03 | 3.25 |
| 2015 | CTD_Mi_ | 1325639_SNP | 6A | 93.22 | 0.07 | 4.83 |
| 2013 | NGS | 1325639_SNP | 6A | 93.22 | 0.04 | 3.07 |
| 2015 | D_AM_ | 4404532_PAV | 6A | 98.16 | 0.03 | 3.42 |
| 2015 | D_AM_ | 4405332_PAV | 6A | 98.16 | 0.03 | 3.42 |
| 2013 | NS | 1110824_PAV | 6A | 98.65 | 0.04 | 3.32 |
| 2013 | RUE_AM_ | 1144393_PAV | 6A | 98.82 | 0.03 | 3.15 |
| 2013 | PH | 1225135_PAV | 6A | 99.41 | 0.03 | 3.05 |
| 2015 | NS | 1103762_PAV | 6B | 2.41 | 0.04 | 3.35 |
| 2014 | D_EH_ | 1695368_SNP | 6B | 2.41 | 0.01 | 4.48 |
| 2014 | GA_90d_ | 1695368_SNP | 6B | 2.41 | 0.06 | 3.71 |
| 2013 | GFR | 4398005_PAV | 6B | 2.56 | 0.04 | 3.84 |
| 2013 | cPAR_EH_ | 1103772_PAV | 6B | 2.64 | 0.03 | 3.41 |
| 2013 | D_EH_ | 996294_PAV | 6B | 3.01 | 0.01 | 3.20 |
| 2013 | D_AM_ | 3029582_PAV | 6B | 3.27 | 0.03 | 3.13 |
| 2015 | HI | 3948891_PAV | 6B | 3.53 | 0.03 | 3.10 |
| 2015 | HI | 3959828_PAV | 6B | 3.53 | 0.03 | 3.10 |
| 2013 | D_EH_ | 1268312_PAV | 6B | 3.56 | 0.01 | 3.44 |
| 2013 | GFR | 1150792_PAV | 6B | 3.83 | 0.03 | 3.25 |
| 2015 | cPAR_AM_ | 3934280_PAV | 6B | 4.06 | 0.04 | 3.28 |
| 2015 | D_AM_ | 3934280_PAV | 6B | 4.06 | 0.03 | 3.10 |
| 2015 | D_EH_ | 1719913_PAV | 6B | 4.71 | 0.00 | 3.02 |
| 2014 | W | 1123590_PAV | 6B | 5.27 | 0.04 | 3.40 |
| 2015 | NGS | 1255704_PAV | 6B | 6 | 0.03 | 3.01 |
| 2015 | cPAR_AM_ | 4005306_PAV | 6B | 6.02 | 0.04 | 3.65 |
| 2014 | GFR | 1240071_PAV | 6B | 6.6 | 0.03 | 3.08 |
| 2013 | Yield | 4409401_PAV | 6B | 6.6 | 0.03 | 3.13 |
| 2015 | cPAR_AM_ | 987856_SNP | 6B | 6.6 | 0.06 | 3.24 |
| 2015 | cPAR_AM_ | 3025373_PAV | 6B | 6.67 | 0.03 | 3.02 |
| 2015 | DM_A_ | 1265510_PAV | 6B | 8.19 | 0.03 | 3.00 |
| 2013 | GA_90d_ | 4010031_PAV | 6B | 8.19 | 0.04 | 3.40 |
| 2013 | cPAR_EH_ | 4010031_PAV | 6B | 8.19 | 0.02 | 3.14 |
| 2015 | DM_A_ | 981023_SNP | 6B | 8.19 | 0.04 | 3.07 |
| 2013 | D_HA_ | 4009269_SNP | 6B | 8.72 | 0.04 | 3.69 |
| 2015 | cPAR_AM_ | 1161159_PAV | 6B | 8.76 | 0.03 | 3.08 |
| 2015 | PL | 2260491_PAV | 6B | 8.9 | 0.03 | 3.15 |
| 2013 | cPAR_EH_ | 3953601_PAV | 6B | 9.3 | 0.03 | 3.84 |
| 2013 | cPAR_EH_ | 4412225_PAV | 6B | 9.3 | 0.03 | 3.40 |
| 2015 | GA_90d_ | 4412225_PAV | 6B | 9.3 | 0.03 | 3.39 |
| 2015 | Yield | 1132684_SNP | 6B | 12.31 | 0.06 | 4.13 |
| 2014 | DM_M_ | 1072346_PAV | 6B | 13.14 | 0.03 | 3.05 |
| 2014 | GA_90d_ | 2279614_PAV | 6B | 13.14 | 0.04 | 3.44 |
| 2014 | D_AM_ | 3022077_PAV | 6B | 13.14 | 0.04 | 3.38 |
| 2013 | CTD_Mi_ | 1062595_PAV | 6B | 13.55 | 0.04 | 3.61 |
| 2013 | W | 1069368_PAV | 6B | 14.47 | 0.03 | 3.03 |
| 2014 | CTD_A_ | 1113826_PAV | 6B | 16.28 | 0.03 | 3.29 |
| 2013 | cPAR_HA_ | 2275505_PAV | 6B | 17.7 | 0.03 | 3.11 |
| 2014 | NGS | 3021084_SNP | 6B | 18.78 | 0.05 | 4.27 |
| 2015 | CTD_Mi_ | 2260398_SNP | 6B | 19.94 | 0.06 | 3.03 |
| 2014 | D_EH_ | 4407321_PAV | 6B | 22.02 | 0.00 | 3.02 |
| 2014 | GA_90d_ | 1023726_SNP | 6B | 22.92 | 0.05 | 3.60 |
| 2014 | RUE_AM_ | 1162190_PAV | 6B | 23.19 | 0.04 | 3.32 |
| 2014 | W | 3958012_SNP | 6B | 23.73 | 0.05 | 3.17 |
| 2014 | NS | 1105240_SNP | 6B | 23.91 | 0.04 | 3.18 |
| 2013 | cPAR_EH_ | 3938048_PAV | 6B | 26.19 | 0.03 | 3.56 |
| 2015 | CTD_Mi_ | 4004534_PAV | 6B | 26.19 | 0.03 | 3.38 |
| 2013 | NGS | 997388_SNP | 6B | 26.3 | 0.05 | 3.58 |
| 2013 | cPAR_EH_ | 3570024_SNP | 6B | 27.34 | 0.03 | 3.30 |
| 2014 | HI | 4408306_PAV | 6B | 29.18 | 0.03 | 3.25 |
| 2014 | CTD_A_ | 4408306_PAV | 6B | 29.18 | 0.03 | 3.22 |
| 2013 | W | 995200_SNP | 6B | 30.64 | 0.05 | 3.71 |
| 2013 | GFR | 995200_SNP | 6B | 30.64 | 0.05 | 3.56 |
| 2014 | RUE_EA_ | 1067759_PAV | 6B | 30.95 | 0.03 | 3.02 |
| 2014 | RUE_EA_ | 1279954_PAV | 6B | 30.95 | 0.03 | 3.13 |
| 2013 | GA_90d_ | 2289521_PAV | 6B | 30.95 | 0.03 | 3.13 |
| 2013 | PL | 1128546_PAV | 6B | 31.07 | 0.04 | 3.89 |
| 2014 | D_HA_ | 1091970_PAV | 6B | 31.2 | 0.03 | 3.06 |
| 2014 | cPAR_HA_ | 3940886_PAV | 6B | 31.2 | 0.03 | 3.46 |
| 2014 | D_HA_ | 3940886_PAV | 6B | 31.2 | 0.03 | 3.32 |
| 2013 | DM_M_ | 1143173_PAV | 6B | 31.37 | 0.03 | 3.23 |
| 2013 | NS | 1104306_PAV | 6B | 31.43 | 0.03 | 3.02 |
| 2013 | GA_90d_ | 1125021_PAV | 6B | 31.49 | 0.04 | 3.26 |
| 2015 | GA_90d_ | 1107429_PAV | 6B | 31.57 | 0.05 | 4.20 |
| 2013 | cPAR_EH_ | 1162870_PAV | 6B | 32.03 | 0.02 | 3.18 |
| 2015 | DM_A_ | 4004412_PAV | 6B | 32.18 | 0.03 | 3.01 |
| 2015 | D_EH_ | 3028170_SNP | 6B | 32.95 | 0.01 | 7.01 |
| 2013 | DM_A_ | 1159426_PAV | 6B | 33.05 | 0.03 | 3.13 |
| 2014 | D_HA_ | 980657_PAV | 6B | 33.34 | 0.03 | 3.27 |
| 2014 | D_HA_ | 2277449_PAV | 6B | 34.56 | 0.03 | 3.46 |
| 2014 | D_HA_ | 1228550_PAV | 6B | 34.61 | 0.02 | 3.00 |
| 2015 | CTD_A_ | 3945402_PAV | 6B | 34.61 | 0.03 | 3.10 |
| 2013 | cPAR_HA_ | 1127811_PAV | 6B | 34.67 | 0.04 | 3.32 |
| 2014 | D_HA_ | 1001409_PAV | 6B | 34.89 | 0.03 | 3.12 |
| 2013 | Yield | 3024822_SNP | 6B | 35.53 | 0.06 | 5.06 |
| 2013 | PH | 1016455_SNP | 6B | 35.91 | 0.05 | 3.23 |
| 2013 | GFR | 3956342_PAV | 6B | 36.07 | 0.03 | 3.02 |
| 2014 | D_HA_ | 1380636_PAV | 6B | 36.34 | 0.03 | 3.80 |
| 2014 | D_EH_ | 3026491_SNP | 6B | 37.96 | 0.00 | 3.08 |
| 2013 | cPAR_HA_ | 1138977_PAV | 6B | 39.34 | 0.03 | 3.15 |
| 2013 | cPAR_HA_ | 991424_SNP | 6B | 39.34 | 0.04 | 3.21 |
| 2013 | DM_M_ | 1125597_SNP | 6B | 40.47 | 0.04 | 3.39 |
| 2013 | RUE_EA_ | 1100655_SNP | 6B | 41.07 | 0.04 | 3.24 |
| 2014 | PL | 1216241_PAV | 6B | 43.41 | 0.04 | 3.29 |
| 2014 | DM_M_ | 1121033_PAV | 6B | 44.47 | 0.03 | 3.16 |
| 2014 | PL | 979489_PAV | 6B | 44.55 | 0.03 | 3.39 |
| 2015 | D_HA_ | 4408467_PAV | 6B | 45.53 | 0.04 | 3.48 |
| 2013 | NGS | 1091395_SNP | 6B | 54.11 | 0.04 | 3.42 |
| 2014 | cPAR_HA_ | 1003657_PAV | 6B | 54.79 | 0.03 | 3.48 |
| 2013 | PH | 1095373_SNP | 6B | 55.31 | 0.04 | 3.30 |
| 2015 | PL | 1108678_PAV | 6B | 72.43 | 0.03 | 3.42 |
| 2014 | PL | 1206538_SNP | 6B | 73.06 | 0.05 | 3.28 |
| 2014 | PH | 3960395_PAV | 6B | 78.46 | 0.04 | 3.98 |
| 2015 | Yield | 4411921_SNP | 6B | 78.75 | 0.05 | 3.40 |
| 2013 | DM_A_ | 1086601_PAV | 6B | 79.59 | 0.03 | 3.01 |
| 2014 | PL | 1394611_PAV | 6B | 79.59 | 0.03 | 3.03 |
| 2013 | D_EH_ | 1089981_PAV | 6B | 81.53 | 0.01 | 3.03 |
| 2015 | PH | 1382659_PAV | 6B | 81.53 | 0.04 | 3.91 |
| 2013 | D_HA_ | 4003631_PAV | 6B | 82.09 | 0.02 | 3.12 |
| 2015 | PH | 4411562_PAV | 6B | 82.13 | 0.04 | 3.92 |
| 2015 | PL | 4411562_PAV | 6B | 82.13 | 0.03 | 3.06 |
| 2014 | PL | 1099940_PAV | 6B | 82.16 | 0.03 | 3.11 |
| 2015 | D_EH_ | 4408471_PAV | 6B | 82.79 | 0.00 | 3.09 |
| 2015 | PH | 3534106_PAV | 6B | 82.96 | 0.04 | 3.62 |
| 2015 | PH | 3937871_PAV | 6B | 82.96 | 0.03 | 3.34 |
| 2013 | DM_A_ | 3939497_PAV | 6B | 82.96 | 0.03 | 3.09 |
| 2013 | PL | 3940956_PAV | 6B | 82.98 | 0.03 | 3.28 |
| 2013 | D_HA_ | 1125282_PAV | 6B | 83.09 | 0.03 | 3.46 |
| 2015 | Yield | 2276364_PAV | 6B | 83.19 | 0.03 | 3.08 |
| 2015 | Yield | 3941298_PAV | 6B | 83.19 | 0.03 | 3.04 |
| 2015 | Yield | 3949917_PAV | 6B | 83.19 | 0.03 | 3.04 |
| 2015 | Yield | 3956383_PAV | 6B | 83.19 | 0.04 | 3.32 |
| 2013 | cPAR_HA_ | 4406343_PAV | 6B | 83.19 | 0.03 | 3.07 |
| 2014 | CTD_Mi_ | 1073031_PAV | 6B | 83.51 | 0.03 | 3.09 |
| 2015 | Yield | 1124447_PAV | 6B | 83.75 | 0.03 | 3.51 |
| 2013 | D_HA_ | 1242271_PAV | 6B | 83.9 | 0.02 | 3.09 |
| 2013 | NGS | 1095843_PAV | 7A | 3.96 | 0.04 | 3.27 |
| 2013 | GFR | 1130460_PAV | 7A | 5.32 | 0.05 | 4.24 |
| 2013 | W | 1130460_PAV | 7A | 5.32 | 0.04 | 3.55 |
| 2013 | GFR | 1140734_PAV | 7A | 5.32 | 0.03 | 3.13 |
| 2013 | NGS | 2275947_PAV | 7A | 5.32 | 0.04 | 3.52 |
| 2013 | NGS | 1124781_PAV | 7A | 6.46 | 0.03 | 3.15 |
| 2013 | NGS | 4408555_PAV | 7A | 7.13 | 0.04 | 3.69 |
| 2014 | cPAR_HA_ | 3533848_PAV | 7A | 7.84 | 0.03 | 3.64 |
| 2015 | DM_M_ | 3955932_PAV | 7A | 7.84 | 0.03 | 3.14 |
| 2013 | GA_90d_ | 3959125_PAV | 7A | 8.01 | 0.03 | 3.09 |
| 2014 | cPAR_HA_ | 2325140_PAV | 7A | 8.08 | 0.04 | 3.96 |
| 2015 | Yield | 1695340_SNP | 7A | 8.21 | 0.04 | 3.06 |
| 2014 | cPAR_HA_ | 1000950_PAV | 7A | 9.34 | 0.03 | 3.47 |
| 2014 | cPAR_HA_ | 1064039_PAV | 7A | 9.43 | 0.03 | 3.68 |
| 2014 | cPAR_HA_ | 1071972_PAV | 7A | 9.43 | 0.03 | 3.13 |
| 2015 | RUE_AM_ | 2290770_PAV | 7A | 9.81 | 0.03 | 3.35 |
| 2013 | NGS | 1119666_PAV | 7A | 10.48 | 0.03 | 3.16 |
| 2013 | cPAR_HA_ | 4005646_PAV | 7A | 14.97 | 0.04 | 3.38 |
| 2015 | NS | 4411602_PAV | 7A | 15.34 | 0.04 | 3.56 |
| 2014 | PL | 3935192_PAV | 7A | 15.73 | 0.03 | 3.24 |
| 2014 | PL | 3950848_PAV | 7A | 15.73 | 0.03 | 3.24 |
| 2014 | PL | 3956612_PAV | 7A | 15.73 | 0.03 | 3.24 |
| 2014 | PL | 3959224_PAV | 7A | 15.73 | 0.03 | 3.24 |
| 2014 | PL | 4411245_PAV | 7A | 17.19 | 0.05 | 3.87 |
| 2013 | DM_A_ | 1216004_SNP | 7A | 18.92 | 0.05 | 3.88 |
| 2013 | RUE_EA_ | 1216004_SNP | 7A | 18.92 | 0.06 | 3.84 |
| 2014 | PH | 1216004_SNP | 7A | 18.92 | 0.05 | 3.71 |
| 2014 | PL | 1216004_SNP | 7A | 18.92 | 0.04 | 3.00 |
| 2013 | cPAR_AM_ | 3021927_PAV | 7A | 19.77 | 0.04 | 3.12 |
| 2013 | Yield | 3064495_SNP | 7A | 23.91 | 0.04 | 3.03 |
| 2015 | cPAR_AM_ | 3941118_PAV | 7A | 28.18 | 0.03 | 3.21 |
| 2015 | CTD_A_ | 1087984_PAV | 7A | 31.55 | 0.04 | 3.77 |
| 2015 | D_EH_ | 1016078_PAV | 7A | 32.92 | 0.00 | 3.47 |
| 2014 | DM_A_ | 4405371_PAV | 7A | 32.92 | 0.04 | 3.68 |
| 2014 | DM_A_ | 4405886_PAV | 7A | 32.92 | 0.04 | 3.68 |
| 2014 | DM_A_ | 4410232_PAV | 7A | 32.92 | 0.04 | 3.68 |
| 2014 | DM_A_ | 2279740_PAV | 7A | 33.85 | 0.03 | 3.15 |
| 2013 | DM_M_ | 1102645_SNP | 7A | 39.65 | 0.04 | 3.19 |
| 2015 | CTD_A_ | 1714396_PAV | 7A | 40.08 | 0.05 | 4.59 |
| 2015 | CTD_A_ | 981641_PAV | 7A | 40.08 | 0.03 | 3.38 |
| 2013 | W | 1240207_PAV | 7A | 40.32 | 0.04 | 3.71 |
| 2015 | CTD_A_ | 3960223_PAV | 7A | 40.32 | 0.04 | 4.19 |
| 2015 | CTD_A_ | 3935769_PAV | 7A | 40.64 | 0.05 | 5.20 |
| 2015 | CTD_A_ | 3943939_PAV | 7A | 40.72 | 0.04 | 3.52 |
| 2015 | CTD_A_ | 1221569_PAV | 7A | 40.76 | 0.05 | 4.86 |
| 2015 | CTD_A_ | 3952170_PAV | 7A | 41.52 | 0.04 | 3.63 |
| 2013 | NS | 1143068_SNP | 7A | 42.34 | 0.04 | 3.05 |
| 2015 | NGS | 1039958_PAV | 7A | 42.82 | 0.04 | 3.58 |
| 2015 | D_EH_ | 1039958_PAV | 7A | 42.82 | 0.00 | 3.04 |
| 2014 | CTD_Mi_ | 2276806_SNP | 7A | 42.82 | 0.05 | 4.36 |
| 2015 | CTD_A_ | 4003949_PAV | 7A | 43.58 | 0.04 | 3.70 |
| 2013 | HI | 1117680_PAV | 7A | 44.16 | 0.04 | 3.67 |
| 2015 | PL | 4404658_PAV | 7A | 44.16 | 0.04 | 3.50 |
| 2013 | PL | 4404658_PAV | 7A | 44.16 | 0.03 | 3.37 |
| 2015 | PH | 4404658_PAV | 7A | 44.16 | 0.03 | 3.31 |
| 2015 | PL | 4411631_PAV | 7A | 44.16 | 0.04 | 3.50 |
| 2013 | PL | 4411631_PAV | 7A | 44.16 | 0.03 | 3.37 |
| 2015 | PH | 4411631_PAV | 7A | 44.16 | 0.03 | 3.31 |
| 2015 | CTD_A_ | 1240224_PAV | 7A | 44.34 | 0.04 | 4.10 |
| 2015 | CTD_A_ | 3022503_SNP | 7A | 44.34 | 0.05 | 3.69 |
| 2015 | PH | 2259227_SNP | 7A | 44.54 | 0.04 | 3.66 |
| 2015 | PL | 1864524_SNP | 7A | 44.75 | 0.04 | 3.13 |
| 2015 | GFR | 4410295_PAV | 7A | 46.23 | 0.04 | 4.10 |
| 2015 | W | 4410295_PAV | 7A | 46.23 | 0.04 | 3.41 |
| 2015 | GFR | 1103056_PAV | 7A | 47.24 | 0.04 | 3.43 |
| 2015 | GFR | 1133556_PAV | 7A | 47.24 | 0.05 | 4.10 |
| 2015 | W | 1133556_PAV | 7A | 47.24 | 0.04 | 3.74 |
| 2015 | PL | 3942108_PAV | 7A | 47.94 | 0.03 | 3.27 |
| 2015 | HI | 3935597_PAV | 7A | 49.71 | 0.04 | 4.15 |
| 2013 | HI | 3935597_PAV | 7A | 49.71 | 0.04 | 3.75 |
| 2015 | HI | 1085123_SNP | 7A | 51.35 | 0.04 | 3.35 |
| 2015 | HI | 1094632_PAV | 7A | 52.56 | 0.03 | 3.06 |
| 2015 | Yield | 1092933_PAV | 7A | 53.37 | 0.04 | 3.17 |
| 2015 | cPAR_AM_ | 1092933_PAV | 7A | 53.37 | 0.03 | 3.04 |
| 2015 | cPAR_AM_ | 1099321_PAV | 7A | 53.37 | 0.04 | 3.40 |
| 2015 | NGS | 1270625_PAV | 7A | 54.88 | 0.03 | 3.23 |
| 2015 | NGS | 4410656_PAV | 7A | 54.88 | 0.04 | 3.15 |
| 2015 | HI | 1106305_PAV | 7A | 57.97 | 0.03 | 3.15 |
| 2015 | CTD_Mi_ | 1138452_PAV | 7A | 58.28 | 0.03 | 3.10 |
| 2013 | PL | 1121759_SNP | 7A | 58.55 | 0.04 | 3.07 |
| 2013 | NGS | 1101320_PAV | 7A | 61.34 | 0.04 | 3.49 |
| 2014 | PH | 1279884_SNP | 7A | 63.42 | 0.05 | 3.73 |
| 2014 | HI | 1229073_PAV | 7A | 70.1 | 0.05 | 5.27 |
| 2014 | Yield | 1229073_PAV | 7A | 70.1 | 0.03 | 3.02 |
| 2015 | CTD_Mi_ | 1136367_PAV | 7A | 71.92 | 0.03 | 3.09 |
| 2013 | DM_M_ | 1276940_SNP | 7A | 71.92 | 0.04 | 3.12 |
| 2013 | PH | 1218096_PAV | 7A | 74.44 | 0.03 | 3.35 |
| 2015 | DM_A_ | 1040441_SNP | 7A | 75.27 | 0.04 | 3.10 |
| 2013 | DM_M_ | 1059377_SNP | 7A | 75.27 | 0.05 | 3.21 |
| 2015 | D_HA_ | 2278487_PAV | 7A | 75.7 | 0.03 | 3.26 |
| 2013 | NGS | 3024298_PAV | 7A | 75.85 | 0.04 | 3.16 |
| 2015 | D_HA_ | 1102644_PAV | 7A | 75.93 | 0.03 | 3.12 |
| 2015 | Yield | 2289574_SNP | 7A | 76.09 | 0.04 | 3.19 |
| 2013 | D_EH_ | 1082845_SNP | 7A | 79.35 | 0.01 | 3.67 |
| 2014 | CTD_Mi_ | 1121117_SNP | 7A | 81.76 | 0.04 | 3.21 |
| 2015 | HI | 1165766_PAV | 7A | 81.76 | 0.03 | 3.42 |
| 2013 | NGS | 1160461_SNP | 7A | 89 | 0.04 | 3.00 |
| 2015 | cPAR_EH_ | 1216389_PAV | 7A | 89.5 | 0.03 | 3.20 |
| 2013 | D_HA_ | 1240049_PAV | 7A | 89.79 | 0.02 | 3.05 |
| 2015 | CTD_Mi_ | 1128715_PAV | 7A | 90.22 | 0.03 | 3.27 |
| 2014 | D_AM_ | 3534059_PAV | 7A | 92.48 | 0.04 | 3.47 |
| 2014 | CTD_A_ | 1133449_PAV | 7A | 94.87 | 0.03 | 3.11 |
| 2013 | DM_M_ | 3064866_SNP | 7A | 97.27 | 0.04 | 3.48 |
| 2014 | D_EH_ | 3957048_PAV | 7A | 100.95 | 0.01 | 3.98 |
| 2013 | NGS | 2262108_SNP | 7A | 105.24 | 0.04 | 3.18 |
| 2013 | cPAR_AM_ | 997028_SNP | 7A | 113.45 | 0.04 | 3.16 |
| 2013 | NGS | 2264620_SNP | 7A | 122.01 | 0.04 | 3.02 |
| 2015 | CTD_A_ | 1670669_SNP | 7A | 122.85 | 0.05 | 3.66 |
| 2013 | NS | 1091331_SNP | 7A | 123.73 | 0.04 | 3.03 |
| 2015 | PL | 1010264_SNP | 7A | 135.24 | 0.06 | 3.12 |
| 2015 | PH | 1025127_PAV | 7A | 135.24 | 0.03 | 3.34 |
| 2014 | PH | 1025127_PAV | 7A | 135.24 | 0.03 | 3.20 |
| 2015 | Yield | 1025127_PAV | 7A | 135.24 | 0.03 | 3.08 |
| 2014 | CTD_Mi_ | 1110502_SNP | 7A | 135.62 | 0.05 | 3.27 |
| 2014 | NS | 1124832_SNP | 7A | 142.38 | 0.04 | 3.02 |
| 2013 | NGS | 1044837_SNP | 7A | 143.53 | 0.04 | 3.15 |
| 2013 | cPAR_HA_ | 2288870_PAV | 7A | 143.53 | 0.04 | 3.73 |
| 2013 | CTD_Mi_ | 1094835_PAV | 7A | 143.75 | 0.03 | 3.11 |
| 2013 | PH | 994221_PAV | 7A | 146.41 | 0.03 | 3.00 |
| 2013 | CTD_Mi_ | 3575400_PAV | 7A | 148.08 | 0.03 | 3.04 |
| 2014 | CTD_Mi_ | 1100092_SNP | 7A | 149.56 | 0.05 | 3.76 |
| 2013 | RUE_AM_ | 1111000_PAV | 7A | 149.56 | 0.03 | 3.23 |
| 2014 | CTD_Mi_ | 1206653_SNP | 7A | 149.56 | 0.04 | 3.22 |
| 2013 | CTD_Mi_ | 1230781_SNP | 7A | 149.56 | 0.05 | 3.41 |
| 2013 | CTD_Mi_ | 1070267_PAV | 7A | 149.62 | 0.04 | 3.61 |
| 2015 | cPAR_AM_ | 1274052_PAV | 7A | 149.67 | 0.03 | 3.18 |
| 2013 | RUE_AM_ | 990827_SNP | 7A | 149.87 | 0.04 | 3.01 |
| 2013 | cPAR_HA_ | 3024648_PAV | 7A | 150.26 | 0.03 | 3.03 |
| 2014 | D_AM_ | 1230365_PAV | 7A | 151.33 | 0.04 | 3.40 |
| 2015 | D_EH_ | 1201022_SNP | 7A | 152.87 | 0.01 | 4.50 |
| 2013 | HI | 1246509_PAV | 7A | 154.85 | 0.03 | 3.28 |
| 2013 | NS | 4410818_PAV | 7A | 155 | 0.04 | 3.29 |
| 2014 | D_EH_ | 1219087_SNP | 7A | 155.08 | 0.01 | 4.87 |
| 2015 | D_EH_ | 1219087_SNP | 7A | 155.08 | 0.00 | 3.00 |
| 2013 | Yield | 1665919_PAV | 7A | 155.69 | 0.04 | 3.30 |
| 2013 | Yield | 1668935_PAV | 7A | 155.69 | 0.03 | 3.21 |
| 2015 | RUE_AM_ | 1289210_SNP | 7A | 156.02 | 0.04 | 3.24 |
| 2013 | HI | 1165351_PAV | 7A | 156.25 | 0.04 | 4.41 |
| 2014 | cPAR_EH_ | 1078867_PAV | 7A | 156.87 | 0.03 | 3.26 |
| 2013 | Yield | 994027_SNP | 7A | 156.87 | 0.04 | 3.09 |
| 2014 | CTD_Mi_ | 987617_PAV | 7A | 160.23 | 0.04 | 3.92 |
| 2015 | HI | 2293197_PAV | 7B | 0.57 | 0.03 | 3.18 |
| 2015 | HI | 3950364_PAV | 7B | 0.86 | 0.03 | 3.14 |
| 2015 | D_HA_ | 1181312_PAV | 7B | 12.5 | 0.04 | 3.37 |
| 2015 | cPAR_HA_ | 1181312_PAV | 7B | 12.5 | 0.03 | 3.16 |
| 2013 | NS | 4404719_PAV | 7B | 14.43 | 0.06 | 5.08 |
| 2013 | cPAR_HA_ | 1045660_SNP | 7B | 14.86 | 0.05 | 3.61 |
| 2013 | NS | 4408134_PAV | 7B | 14.96 | 0.06 | 5.11 |
| 2013 | NS | 4409613_PAV | 7B | 14.96 | 0.06 | 5.11 |
| 2015 | PH | 1103135_PAV | 7B | 17.54 | 0.03 | 3.11 |
| 2013 | Yield | 2280118_SNP | 7B | 21.48 | 0.05 | 3.24 |
| 2015 | D_HA_ | 1114159_PAV | 7B | 22.1 | 0.04 | 4.33 |
| 2015 | D_HA_ | 1161197_PAV | 7B | 22.1 | 0.03 | 3.56 |
| 2014 | D_EH_ | 1083871_SNP | 7B | 22.43 | 0.01 | 4.65 |
| 2015 | D_EH_ | 1083871_SNP | 7B | 22.43 | 0.01 | 4.33 |
| 2013 | PL | 1083871_SNP | 7B | 22.43 | 0.05 | 3.55 |
| 2013 | cPAR_EH_ | 1083871_SNP | 7B | 22.43 | 0.03 | 3.28 |
| 2015 | D_HA_ | 3957311_PAV | 7B | 22.43 | 0.03 | 3.15 |
| 2013 | cPAR_HA_ | 1180291_PAV | 7B | 22.57 | 0.04 | 3.78 |
| 2013 | cPAR_HA_ | 1071948_SNP | 7B | 22.99 | 0.03 | 3.33 |
| 2013 | PH | 1111344_PAV | 7B | 24.48 | 0.04 | 4.00 |
| 2014 | PL | 987928_SNP | 7B | 36.15 | 0.04 | 3.31 |
| 2013 | PL | 3956502_SNP | 7B | 39.49 | 0.04 | 3.10 |
| 2015 | DM_A_ | 4003570_PAV | 7B | 43.76 | 0.04 | 3.71 |
| 2015 | DM_A_ | 1000207_PAV | 7B | 45.12 | 0.06 | 5.13 |
| 2015 | cPAR_EH_ | 1000207_PAV | 7B | 45.12 | 0.04 | 3.78 |
| 2013 | cPAR_AM_ | 3064400_SNP | 7B | 45.76 | 0.05 | 3.64 |
| 2015 | PH | 4404594_PAV | 7B | 46.26 | 0.04 | 3.84 |
| 2015 | PL | 4404594_PAV | 7B | 46.26 | 0.03 | 3.34 |
| 2014 | PH | 1090053_SNP | 7B | 47.58 | 0.05 | 4.10 |
| 2014 | PL | 1015949_PAV | 7B | 48.02 | 0.05 | 3.99 |
| 2014 | PH | 1015949_PAV | 7B | 48.02 | 0.03 | 3.46 |
| 2014 | PH | 1060180_SNP | 7B | 48.3 | 0.06 | 3.13 |
| 2014 | PL | 4395106_PAV | 7B | 48.55 | 0.03 | 3.23 |
| 2014 | PH | 1140964_SNP | 7B | 48.86 | 0.05 | 4.02 |
| 2013 | PL | 1140964_SNP | 7B | 48.86 | 0.05 | 3.39 |
| 2013 | NGS | 1032850_SNP | 7B | 49.02 | 0.04 | 3.19 |
| 2013 | NGS | 1055292_SNP | 7B | 49.02 | 0.04 | 3.30 |
| 2013 | NGS | 3064904_SNP | 7B | 49.42 | 0.05 | 4.11 |
| 2015 | D_AM_ | 1104601_PAV | 7B | 49.78 | 0.03 | 3.68 |
| 2015 | RUE_AM_ | 1220160_SNP | 7B | 52.69 | 0.04 | 3.28 |
| 2014 | cPAR_HA_ | 4008560_PAV | 7B | 53.84 | 0.03 | 3.08 |
| 2014 | PH | 1063567_SNP | 7B | 57.08 | 0.07 | 5.60 |
| 2015 | PH | 1063567_SNP | 7B | 57.08 | 0.06 | 5.14 |
| 2015 | PL | 1063567_SNP | 7B | 57.08 | 0.05 | 3.83 |
| 2014 | PL | 1063567_SNP | 7B | 57.08 | 0.04 | 3.25 |
| 2014 | PH | 1193598_SNP | 7B | 57.08 | 0.04 | 3.23 |
| 2013 | DM_A_ | 1193598_SNP | 7B | 57.08 | 0.04 | 3.19 |
| 2015 | D_EH_ | 2278592_PAV | 7B | 57.08 | 0.00 | 3.15 |
| 2014 | NGS | 4409440_PAV | 7B | 57.37 | 0.03 | 3.18 |
| 2014 | NGS | 4410233_PAV | 7B | 57.37 | 0.03 | 3.18 |
| 2014 | NGS | 4411277_PAV | 7B | 57.37 | 0.03 | 3.18 |
| 2015 | PH | 1216720_PAV | 7B | 57.51 | 0.06 | 5.24 |
| 2015 | PL | 1216720_PAV | 7B | 57.51 | 0.03 | 3.01 |
| 2013 | GA_90d_ | 4406996_PAV | 7B | 57.74 | 0.03 | 3.24 |
| 2015 | D_EH_ | 1116425_PAV | 7B | 57.87 | 0.00 | 3.89 |
| 2014 | cPAR_EH_ | 1126253_PAV | 7B | 57.87 | 0.03 | 3.26 |
| 2013 | GA_90d_ | 1126253_PAV | 7B | 57.87 | 0.03 | 3.13 |
| 2013 | GA_90d_ | 1667120_PAV | 7B | 57.87 | 0.03 | 3.04 |
| 2014 | PH | 1021164_SNP | 7B | 58.25 | 0.04 | 3.47 |
| 2015 | D_EH_ | 1162030_PAV | 7B | 58.64 | 0.00 | 3.14 |
| 2013 | Yield | 1270909_PAV | 7B | 58.8 | 0.04 | 3.49 |
| 2015 | PH | 3938801_PAV | 7B | 59.36 | 0.03 | 3.41 |
| 2014 | RUE_EA_ | 1115475_PAV | 7B | 59.58 | 0.03 | 3.05 |
| 2014 | D_AM_ | 1279301_PAV | 7B | 59.75 | 0.04 | 3.44 |
| 2014 | D_EH_ | 2276347_SNP | 7B | 62.08 | 0.01 | 6.21 |
| 2015 | D_EH_ | 2276347_SNP | 7B | 62.08 | 0.01 | 4.34 |
| 2013 | cPAR_EH_ | 2276347_SNP | 7B | 62.08 | 0.04 | 3.07 |
| 2013 | NGS | 2276691_SNP | 7B | 73.51 | 0.04 | 3.33 |
| 2013 | PH | 3937899_PAV | 7B | 75.21 | 0.03 | 3.37 |
| 2013 | PH | 3533789_PAV | 7B | 75.95 | 0.03 | 3.87 |
| 2015 | D_EH_ | 992944_SNP | 7B | 76.03 | 0.01 | 7.25 |
| 2014 | D_EH_ | 992944_SNP | 7B | 76.03 | 0.01 | 4.47 |
| 2013 | cPAR_EH_ | 992944_SNP | 7B | 76.03 | 0.05 | 4.13 |
| 2013 | PL | 992944_SNP | 7B | 76.03 | 0.05 | 3.50 |
| 2013 | NGS | 3064729_SNP | 7B | 76.61 | 0.05 | 3.53 |
| 2013 | GA_90d_ | 3956303_PAV | 7B | 77.78 | 0.04 | 3.09 |
| 2013 | cPAR_AM_ | 4005210_PAV | 7B | 83.16 | 0.03 | 3.04 |
| 2014 | NS | 1218598_PAV | 7B | 93.44 | 0.04 | 3.37 |
| 2015 | RUE_AM_ | 1125835_SNP | 7B | 95.03 | 0.08 | 5.99 |
| 2015 | DM_M_ | 1125835_SNP | 7B | 95.03 | 0.06 | 4.73 |
| 2015 | NGS | 1125835_SNP | 7B | 95.03 | 0.05 | 4.04 |
| 2015 | GFR | 4329239_PAV | 7B | 95.88 | 0.03 | 3.39 |
| 2015 | W | 4329239_PAV | 7B | 95.88 | 0.03 | 3.22 |
| 2014 | NGS | 4008614_PAV | 7B | 96.2 | 0.03 | 3.12 |
| 2015 | DM_A_ | 1150868_PAV | 7B | 97.45 | 0.04 | 3.62 |
| 2015 | CTD_A_ | 4406173_PAV | 7B | 98.69 | 0.03 | 3.04 |
| 2015 | DM_A_ | 3021556_PAV | 7B | 100.16 | 0.03 | 3.27 |
| 2015 | GA_90d_ | 1228518_PAV | 7B | 100.19 | 0.03 | 3.23 |
| 2015 | GA_90d_ | 1033633_PAV | 7B | 101.58 | 0.03 | 3.31 |
| 2015 | DM_A_ | 1075061_PAV | 7B | 101.58 | 0.04 | 3.35 |
| 2015 | DM_A_ | 1113117_PAV | 7B | 101.58 | 0.04 | 3.67 |
| 2015 | DM_A_ | 1115279_PAV | 7B | 102.27 | 0.04 | 3.45 |
| 2014 | Yield | 1109003_PAV | 7B | 108.77 | 0.03 | 3.16 |
| 2015 | D_EH_ | 1212126_PAV | 7B | 110.99 | 0.00 | 3.51 |
| 2015 | D_AM_ | 1212636_PAV | 7B | 111.13 | 0.03 | 3.82 |
| 2014 | D_AM_ | 3938625_PAV | 7B | 111.13 | 0.04 | 3.70 |
| 2015 | D_EH_ | 4396862_PAV | 7B | 111.13 | 0.00 | 4.07 |
| 2015 | D_EH_ | 4005312_PAV | 7B | 111.2 | 0.00 | 3.18 |
| 2015 | D_EH_ | 1129312_PAV | 7B | 111.22 | 0.01 | 4.12 |
| 2015 | D_EH_ | 1109812_PAV | 7B | 111.23 | 0.00 | 3.52 |
| 2015 | D_EH_ | 1117841_PAV | 7B | 111.23 | 0.00 | 3.58 |
| 2015 | D_EH_ | 1119155_PAV | 7B | 111.23 | 0.00 | 3.75 |
| 2015 | D_EH_ | 1127024_PAV | 7B | 111.23 | 0.00 | 3.44 |
| 2015 | D_EH_ | 1150487_PAV | 7B | 111.23 | 0.00 | 3.27 |
| 2015 | D_EH_ | 1153435_PAV | 7B | 111.23 | 0.00 | 3.47 |
| 2015 | D_EH_ | 1160044_PAV | 7B | 111.23 | 0.00 | 3.40 |
| 2015 | D_EH_ | 1255029_PAV | 7B | 111.23 | 0.00 | 3.05 |
| 2015 | D_EH_ | 3028390_PAV | 7B | 111.23 | 0.00 | 3.34 |
| 2015 | D_EH_ | 3958614_PAV | 7B | 111.23 | 0.00 | 3.54 |
| 2015 | D_EH_ | 3027787_SNP | 7B | 111.65 | 0.01 | 4.31 |
| 2015 | cPAR_AM_ | 1258850_PAV | 7B | 112.18 | 0.03 | 3.21 |
| 2015 | cPAR_AM_ | 1266721_PAV | 7B | 112.18 | 0.04 | 3.53 |
| 2015 | cPAR_AM_ | 1110920_PAV | 7B | 116.03 | 0.03 | 3.05 |
| 2013 | CTD_A_ | 1233517_PAV | 7B | 116.05 | 0.03 | 3.17 |
| 2015 | GFR | 4007960_PAV | 7B | 118.3 | 0.08 | 7.54 |
| 2015 | W | 4007960_PAV | 7B | 118.3 | 0.08 | 7.22 |
| 2013 | W | 4007960_PAV | 7B | 118.3 | 0.07 | 6.73 |
| 2014 | W | 4007960_PAV | 7B | 118.3 | 0.08 | 6.61 |
| 2015 | NGS | 4007960_PAV | 7B | 118.3 | 0.07 | 6.30 |
| 2013 | NGS | 4007960_PAV | 7B | 118.3 | 0.07 | 6.22 |
| 2013 | GFR | 4007960_PAV | 7B | 118.3 | 0.07 | 6.19 |
| 2014 | NGS | 4007960_PAV | 7B | 118.3 | 0.06 | 5.34 |
| 2014 | GFR | 4007960_PAV | 7B | 118.3 | 0.03 | 3.43 |
| 2015 | cPAR_AM_ | 2303939_PAV | 7B | 118.87 | 0.03 | 3.24 |
| 2015 | CTD_Mi_ | 4407137_PAV | 7B | 119.2 | 0.04 | 3.77 |
| 2015 | cPAR_AM_ | 1262374_PAV | 7B | 119.28 | 0.04 | 3.54 |
| 2015 | cPAR_AM_ | 2303250_PAV | 7B | 119.28 | 0.03 | 3.20 |
| 2014 | NS | 4003353_PAV | 7B | 119.32 | 0.03 | 3.09 |
| 2015 | cPAR_AM_ | 1140402_PAV | 7B | 119.75 | 0.03 | 3.11 |
| 2013 | cPAR_HA_ | 3029727_PAV | 7B | 125.23 | 0.03 | 3.08 |
| 2013 | PL | 1031870_PAV | 7B | 125.38 | 0.04 | 3.79 |
| 2013 | PL | 1110872_PAV | 7B | 125.38 | 0.04 | 3.64 |
| 2014 | PH | 1713700_PAV | 7B | 125.96 | 0.03 | 3.00 |
| 2013 | cPAR_EH_ | 3945074_PAV | 7B | 126.01 | 0.02 | 3.09 |
| 2015 | CTD_Mi_ | 4395603_PAV | 7B | 126.34 | 0.04 | 3.71 |
| 2015 | GA_90d_ | 1233538_PAV | 7B | 127.27 | 0.03 | 3.15 |
| 2013 | Yield | 2289109_PAV | 7B | 127.27 | 0.03 | 3.02 |
| 2013 | PH | 986363_SNP | 7B | 128.37 | 0.06 | 5.65 |
| 2013 | PL | 986363_SNP | 7B | 128.37 | 0.05 | 4.52 |
| 2013 | cPAR_HA_ | 1123088_PAV | 7B | 128.71 | 0.03 | 3.13 |
| 2015 | HI | 1010318_PAV | 7B | 128.9 | 0.03 | 3.14 |
| 2013 | cPAR_HA_ | 3023071_PAV | 7B | 128.93 | 0.04 | 3.40 |
| 2013 | D_HA_ | 3023071_PAV | 7B | 128.93 | 0.02 | 3.02 |
| 2013 | cPAR_HA_ | 1265688_PAV | 7B | 129.24 | 0.04 | 3.87 |
| 2013 | cPAR_HA_ | 1266724_PAV | 7B | 129.24 | 0.04 | 3.45 |
| 2013 | cPAR_HA_ | 3534390_PAV | 7B | 129.24 | 0.03 | 3.03 |
| 2013 | cPAR_HA_ | 3943783_PAV | 7B | 129.24 | 0.03 | 3.05 |
| 2013 | cPAR_HA_ | 3950256_PAV | 7B | 129.24 | 0.03 | 3.18 |
| 2013 | cPAR_HA_ | 4407434_PAV | 7B | 129.24 | 0.04 | 3.37 |
| 2013 | cPAR_HA_ | 1260963_PAV | 7B | 129.67 | 0.04 | 3.78 |
| 2013 | cPAR_HA_ | 1260995_PAV | 7B | 132.18 | 0.04 | 3.44 |

*NS, number of spikes m^-2^; NGS, number of grains spike^-1^; W, grain weight; HI, harvest index; DEH, days from emergence to heading; DHA, days from heading to anthesis; DAM, days from anthesis to maturity; GFR, grain filling rate; PL, peduncle length; PH, plant height; DM_A_, dry matter at anthesis; DM_M_, dry matter at maturity; CTD_A_, canopy temperature depression at anthesis; CTD_Mi_, canopy temperature depression at milky-dough grain stage; cPAR_EH_, absorbed radiation from emergence to heading; cPAR_HA_, absorbed radiation from heading to anthesis; cPAR_AM_, absorbed radiation from anthesis to maturity; RUE_EA_, radiation use efficiency from emergence to anthesis; RUE_AM_, radiation use efficiency from anthesis to maturity; GA_90d_, green area accumulated at 90 days from emergence.*

**Supplementary TABLE S4 ǀ** QTL hotspots.

| QTL hotspot | Marker interval | Marker interval (cM) | Marker interval Svevo  (bp) | N MTAs | N MTAs > FDR | Gene | Trait | eigenQTL |
| --- | --- | --- | --- | --- | --- | --- | --- | --- |
| 1A.1 | 1258330 - 1265597 | 6.16 - 8.40 | 91880 - 4578786 | 10 | 0 |  |  |  |
| 1A.2 | 1253011 - 1091468 | 25.31 | 11807383 - 15070642 | 3 | 0 |  |  | eigenQTL1A.1 |
| 1A.3 | 1010408 - 994367 | 62.16 - 67.69 | 42071954 - 48141530 | 6 | 0 |  |  |  |
| 1A.4 | 1011115 - 1104124 | 80.23 - 86.78 | 341299153 - 395707647 | 10 | 0 |  |  | eigenQTL1A.3 |
| 1A.5 | 1099207 - 1229247 | 102.19 - 103.83 | 470196645 - 474435171 | 3 | 0 |  |  | eigenQTL1A.4 |
| 1A.6 | 3023013 - 2362198 | 117.13 - 118.26 | 498547918 - 502415618 | 8 | 2 | Glu-A1 | HMW-GS | eigenQTL1A.5 |
| 1A.7 | 1088769 - 1126768 | 131.88 - 133.6 | 511317924 - 513507234 | 3 | 0 |  |  |  |
| 1A.8 | 1104662 - 1125738 | 150.35 - 152.2 | 526234610 - 531010392 | 2 | 0 |  |  |  |
| 1A.9 | 1003066 - 983365 | 171.85 - 174.33 | 537004445 - 538069994 | 3 | 1 |  |  |  |
| 1A.10 | 1255608 - 2289064 | 191.82 | 554055824 - 563907571 | 3 | 0 |  |  |  |
| 1A.11 | 1125323 - 2282401 | 236.8 - 240.07 | 572493216 - 576806745 | 9 | 0 |  |  |  |
| 1A.12 | 1086532 - 1091952 | 250.14 - 251.28 | 580627786 - 581972945 | 5 | 0 |  |  | eigenQTL1A.6 |
| 1B.1 | 1119717 - 1120149 | 3.58 - 9.59 | 3733178 - 799159 | 33 | 2 |  |  |  |
| 1B.2 | 1269704 - 997400 | 49.8 - 51.58 | 23025564 - 36627769 | 12 | 1 |  |  | eigenQTL1B.3 |
| 1B.3 | 1089943 - 1088924 | 80.42 - 83.3 | 318259349 - 320087687 | 5 | 0 |  |  | eigenQTL1B.4 |
| 1B.4 | 1091112 - 1104284 | 94.95 - 103.29 | 464242427 - 480540712 | 8 | 0 |  |  | eigenQTL1B.5 |
| 1B.5 | 1092345 - 1075055 | 109.71 - 111.63 | 517065777 - 526871560 | 2 | 0 |  |  |  |
| 1B.6 | 983383 - 1103954 | 114.18 - 116.58 | 510750170 - 512991521 | 6 | 0 |  |  |  |
| 1B.7 | 1164132 - 1138184 | 123.55 - 123.64 | 518183146 - 524601651 | 4 | 0 |  |  |  |
| 1B.8 | 1088904 - 983716 | 133.86 - 133.87 | 541898773 - 542806579 | 3 | 0 |  |  |  |
| 1B.9 | 987187 - 1108652 | 142.15 - 142.57 | 553854341 - 556010277 | 4 | 0 |  |  |  |
| 1B.10 | 2279939 - 1404162 | 147.3 - 152.02 | 564010694 - 570894674 | 7 | 0 |  |  |  |
| 1B.11 | 1209708 - 2277983 | 159.81 - 162.40 | 605893532 - 607625318 | 11 | 2 |  |  |  |
| 1B.12 | 1093470 - 999132 | 194.78 - 203.41 | 626692547 - 634435630 | 13 | 1 |  |  | eigenQTL1B.7 |
| 1B.13 | 1002331 - 1261486 | 223.47 - 223.52 | 643040758 - 643441668 | 11 | 0 |  |  |  |
| 2A.1 | 1233368 - 1068370 | 8.29 - 13.34 | 10533187 - 36292042 | 7 | 1 | Ppd-A1 | Photoperiod sensitivity | eigenQTL2A.1 |
| 2A.2 | 2300556 - 991483 | 58.21 - 77.93 | 536500073 - 574093335 | 427 | 177 | TaSus2-2A | Grain weight | eigenQTL2A.3 |
| 2A.3 | 1097075 - 1053641 | 87.88 | 710724866 - 711248515 | 3 | 1 | Ppo-A1 | Polyphenol oxidase | eigenQTL2A.5 |
| 2A.4 | 1378535 - 1021420 | 113.38 - 117.05 | 745356024 - 752280137 | 4 | 0 |  |  | eigenQTL2A.7 |
| 2A.5 | 1130790 - 993911 | 120.2 - 125.1 | 762865326 - 766618939 | 24 | 1 |  |  |  |
| 2B.1 | 1022276 - 1045248 | 0.92 - 2.05 | 3271658 - 5453739 | 5 | 0 |  |  |  |
| 2B.2 | 1092111 - 1023751 | 17.84 | 18801896 - 19248444 | 6 | 0 |  |  |  |
| 2B.3 | 1140483 - 1108293 | 21.44 - 23.11 | 23747329 - 24290057 | 3 | 1 | Ppd-B1 | Photoperiod sensitivity | eigenQTL2B.1 |
| 2B.4 | 985860 - 1036899 | 37.15 - 37.61 | 52905640 - 57457472 | 12 | 1 |  |  | eigenQTL2B.3 |
| 2B.5 | 1067798 - 2324269 | 40.46 | 59524758 - 67088946 | 6 | 0 |  |  |  |
| 2B.6 | 1123167 - 2258931 | 43.8 - 44.08 | 68688216 - 70610437 | 5 | 0 |  |  |  |
| 2B.7 | 994059 - 2278639 | 48.35 - 90.58 | 79849121 - 744258244 | 158 | 4 | Ppo-B2, TaGS2-B1 | Polyphenol oxidase, grain size | eigenQTL2B4,5,6 |
| 2B.8 | 1115174 - 1382299 | 94.68 - 94.83 | 758639083 - 760789049 | 3 | 0 |  |  |  |
| 2B.9 | 1157809 - 1095148 | 104.91 - 109.09 | 772825017 - 774351041 | 22 | 0 |  |  | eigenQTL2B.7 |
| 3A.1 | 1084913 - 1235733 | 3.58 - 5.13 | 1781140 - 1108060 | 13 | 2 |  |  | eigenQTL3A.1 |
| 3A.2 | 1139347 - 1100100 | 8.9 | 12799494 - 14715458 | 3 | 0 |  |  |  |
| 3A.3 | 1002102 - 1002102 | 13.11 - 13.20 | 16638480 - 16638480 | 31 | 0 |  |  | eigenQTL3A.2 |
| 3A.4 | 1221097 - 1103174 | 17.78 - 20.28 | 19785588 - 21468237 | 4 | 0 |  |  |  |
| 3A.5 | 1093449 - 1103922 | 30.03 - 30.89 | 33126175 - 40604077 | 10 | 0 |  |  |  |
| 3A.6 | 1190017 - 1091472 | 34.06 - 36.24 | 36731623 - 49699217 | 5 | 0 |  |  |  |
| 3A.7 | 1058280 - 1117640 | 40.62 - 41.22 | 396956290 - 428003578 | 4 | 2 |  |  | eigenQTL3A.3 |
| 3A.8 | 1075440 - 1220348 | 46.75 - 51.77 | 564479362 - 590415277 | 14 | 0 |  |  |  |
| 3A.9 | 1104683 - 1262350 | 60.70 - 61.93 | 637341839 - 638350859 | 4 | 0 |  |  | eigenQTL3A.5 |
| 3A.10 | 2278035 - 1127468 | 113.23 - 118.46 | 694063712 - 703580616 | 5 | 0 |  |  | eigenQTL3A.7 |
| 3A.11 | 2265545 - 1100688 | 125.21 - 128.91 | 709220874 - 722522001 | 6 | 0 |  |  |  |
| 3A.12 | 1026080 - 1205701 | 133.67 - 137.41 | 726473111 - 729520313 | 4 | 0 | Pod-A1 | Peroxidase activity | eigenQTL3A.8 |
| 3B.1 | 1210301 - 1002215 | 3.48 - 5.83 | 2837475 - 5041882 | 5 | 0 |  |  |  |
| 3B.2 | 1057406 - 1151200 | 8.48 - 13.38 | 6108933 - 7222726 | 21 | 0 |  |  | eigenQTL3B.1 |
| 3B.3 | 1104660 - 1091797 | 17.37 - 17.82 | 19088596 - 20360390 | 8 | 0 |  |  |  |
| 3B.4 | 1203577 - 1071633 | 20.97 - 32.8 | 25599931 - 35796968 | 14 | 0 |  |  | eigenQTL3B.2 |
| 3B.5 | 1051813 - 1135622 | 35.54 | 48236843 - 50909950 | 2 | 2 |  |  |  |
| 3B.6 | 997119 - 2288920 | 50.81 - 61.89 | 135863638 - 478523761 | 17 | 0 |  |  | eigenQTL3B.3 |
| 3B.7 | 2281763 - 1696726 | 64.05 - 68.64 | 551908173 - 596757337 | 13 | 1 |  |  | eigenQTL3B.4 |
| 3B.8 | 2256158 - 2253892 | 77.76 - 84.64 | 713647705 - 731749417 | 10 | 0 |  |  | eigenQTL3B.5 |
| 3B.9 | 1086466 - 1029956 | 90.55 - 95.18 | 749215327 - 763670764 | 5 | 0 |  |  |  |
| 3B.10 | 1130599 - 1028425 | 100.09 - 100.91 | 765615149 - 775230878 | 4 | 0 |  |  | eigenQTL3B.6 |
| 3B.11 | 2275611 - 991025 | 112.86 - 116.95 | 779371203 - 783044579 | 22 | 0 |  |  | eigenQTL4A.6 |
| 3B.12 | 1094389 - 1101844 | 123.48 - 124.12 | 792147339 - 792179647 | 4 | 0 |  |  | eigenQTL4A.7 |
| 3B.13 | 1083013 - 1116790 | 137.99 - 139.03 | 815473183 - 818849394 | 7 | 0 |  |  |  |
| 3B.14 | 1218326 - 1055442 | 156.7 - 157.06 | 820277765 - 830298823 | 4 | 0 | *TaCOMT-3B* | Lodging tolerance |  |
| 4A.1 | 1103219 - 1082281 | 19.84 - 21.99 | 76164392 - 109207621 | 7 | 3 |  |  | eigenQTL4A.1 |
| 4A.2 | 1100988 - 1092501 | 24.24 - 26.08 | 128226021 - 161196183 | 11 | 1 |  |  | eigenQTL4A.2 |
| 4A.3 | 1211780 - 1091178 | 28.5 - 30.86 | 537347277 - 537351517 | 7 | 1 |  |  | eigenQTL4A.3 |
| 4A.4 | 1092069 - 1076361 | 46.97 - 47.12 | 588721592 - 606037529 | 3 | 0 |  |  |  |
| 4A.5 | 1863523 - 1219379 | 66.76 - 72.6 | 610010727 - 610148810 | 6 | 0 |  |  |  |
| 4A.6 | 1033160 - 1017781 | 94.29 - 97.15 | 663911391 - 671497079 | 8 | 1 |  |  | eigenQTL4A.5 |
| 4A.7 | 1091597 - 1860333 | 114.8 - 116.17 | 701340586 - 707837156 | 6 | 0 |  |  | eigenQTL4A.6 |
| 4A.8 | 1074779 - 1053370 | 122.06 - 124.37 | 717796896 - 722043709 | 5 | 0 |  |  | eigenQTL4A.7 |
| 4A.9 | 1229745 - 1126083 | 129.17 - 134.5 | 723605907 - 731636612 | 7 | 2 | TaALP-4A | Preharvest sprouting tolerance |  |
| 4B.1 | 1121395 - 1279588 | 17.44 - 23.86 | 19094241 - 24345048 | 9 | 0 |  |  |  |
| 4B.2 | 1281577 - 1669277 | 31.24 - 35.75 | 51845400 - 266816260 | 11 | 0 |  |  |  |
| 4B.3 | 996108 - 1042220 | 39.44 - 44.26 | 559557406 - 611355193 | 6 | 0 |  |  |  |
| 4B.4 | 1089749 - 1076771 | 47.48 - 49.62 | 596207715 - 623776874 | 4 | 0 | Rht-B1 | Plant height | eigenQTL4B.1 |
| 4B.5 | 1090999 - 2275360 | 52.42 | 630839733 - 643212499 | 2 | 0 |  |  |  |
| 4B.6 | 1213366 - 1016952 | 74.86 - 77.89 | 666183621 - 669247402 | 6 | 0 |  |  | eigenQTL4B.2 |
| 5A.1 | 1069206 - 1069206 | 8.82 | 6005011 - 6005011 | 3 | 1 |  |  |  |
| 5A.2 | 1108615 - 1238050 | 17.79 - 20.01 | 12792063 - 19754455 | 4 | 1 |  |  | eigenQTL5A.1 |
| 5A.3 | 992653 - 1040771 | 34.73 - 39.34 | 197886610 - 395204187 | 11 | 0 |  |  | eigenQTL5A.2 |
| 5A.4 | 1037130 - 1100228 | 48.57 - 55.38 | 437798235 - 457994363 | 13 | 2 |  |  | eigenQTL5A.3 |
| 5A.5 | 1302011 - 1103803 | 58.39 - 63.62 | 490966355 - 515928886 | 16 | 4 |  |  | eigenQTL5A.4 |
| 5A.6 | 1106579 - 1001832 | 79.61 - 86.69 | 530234335 - 532806148 | 19 | 0 | Vrn-A1, Rht12 | Vernalization, plant height | eigenQTL5A.5 |
| 5A.7 | 1268209 - 1215828 | 89.64 - 89.79 | 553551866 - 554226214 | 3 | 0 |  |  |  |
| 5A.8 | 1144964 - 2275454 | 106.35 - 108.68 | 569190244 - 580319248 | 11 | 0 |  |  |  |
| 5A.9 | 1294255 - 983084 | 111.35 - 112.36 | 573111413 - 595690230 | 2 | 0 |  |  | eigenQTL5A.6 |
| 5A.10 | 2281403 - 1668685 | 122.51 - 123.79 | 624456220 - 625374443 | 6 | 0 |  |  |  |
| 5A.11 | 1077619 - 1006069 | 148.79 - 153.71 | 651080155 - 661609283 | 6 | 0 |  |  |  |
| 5B.1 | 1106371 - 1080478 | 0 | 2574772 - 2783500 | 9 | 0 |  |  |  |
| 5B.2 | 1042680 - 1864468 | 8.45 - 16.39 | 14693385 - 26376206 | 21 | 4 |  |  |  |
| 5B.3 | 1114548 - 993288 | 27.12 - 47.6 | 72644042 - 495534224 | 41 | 1 |  |  | eigenQTL5B.1 |
| 5B.4 | 2257217 - 1090157 | 50.75 - 57.24 | 496171743 - 526579133 | 26 | 0 |  |  | eigenQTL5B.2 |
| 5B.5 | 2289116 - 1088160 | 61.69 - 68.79 | 528679912 - 542917858 | 10 | 0 |  |  | eigenQTL5B.3 |
| 5B.6 | 1088160 - 1092044 | 71.45 - 80.27 | 542917858 - 569576778 | 12 | 0 |  |  |  |
| 5B.7 | 2324612 - 1223061 | 90.54 - 91.15 | 593830789 - 595074692 | 2 | 0 |  |  |  |
| 5B.8 | 1047463 - 3024925 | 114.34 - 114.37 | 647238520 - 648079073 | 5 | 1 |  |  | eigenQTL5B.6 |
| 5B.9 | 1086577 - 1093350 | 130.72 - 132.52 | 671316173 - 676152023 | 6 | 0 |  |  |  |
| 5B.10 | 1106570 - 1052428 | 136.42 - 140.05 | 683009042 - 690582803 | 5 | 0 |  |  |  |
| 5B.11 | 1111515 - 1059931 | 148.54 - 148.72 | 699840251 - 699190832 | 3 | 0 |  |  | eigenQTL5B.8 |
| 6A.1 | 997897 - 1111092 | 0.00 - 0.63 | 129624 - 592562 | 3 | 0 |  |  |  |
| 6A.2 | 992973 - 1240246 | 7.40 - 8.87 | 2902440 - 5023607 | 12 | 12 |  |  |  |
| 6A.3 | 1700305 - 1251862 | 15.77 | 10056406 - 10237680 | 18 | 1 | Rht25 | Plant height | eigenQTL6A.1 |
| 6A.4 | 1041418 - 1721876 | 23.76 - 29.26 | 13857479 - 16914898 | 16 | 0 |  |  |  |
| 6A.5 | 2304914 - 1024924 | 47.3 - 50.22 | 99618625 - 446918657 | 16 | 0 |  |  |  |
| 6A.6 | 1237708 - 1237708 | 67.99 - 69.55 | 552132834 - 552132770 | 2 | 0 |  |  |  |
| 6A.7 | 1237708 - 1018852 | 73.16 - 74.35 | 552132770 - 577029150 | 2 | 0 |  |  | eigenQTL6A.2 |
| 6A.8 | 2265652 - 1018852 | 79.70 | 575029214 - 577029150 | 5 | 0 |  |  |  |
| 6A.9 | 1695128 - 1690605 | 83.23 - 88.72 | 590622705 - 596198785 | 14 | 1 |  |  |  |
| 6A.10 | 1126329 - 1101071 | 91.17 - 93.22 | 598564802 - 601482515 | 5 | 0 |  |  |  |
| 6A.11 | 1092748 - 985671 | 98.16 - 99.41 | 603859544 - 611608841 | 5 | 0 |  |  |  |
| 6B.1 | 1065518 - 1076977 | 2.41 - 9.30 | 2428634 - 17651099 | 31 | 0 |  |  | eigenQTL6B.1 |
| 6B.2 | 2275416 - 1096087 | 12.31 - 19.94 | 23346911 - 47668605 | 10 | 0 |  |  | eigenQTL6B.3 |
| 6B.3 | 1095171 - 1091969 | 22.02 - 23.91 | 45583505 - 54555113 | 5 | 2 |  |  |  |
| 6B.4 | 1162120 - 1084826 | 26.19 - 45.53 | 499419007 - 640841391 | 42 | 0 | GPC-B1 | Protein content | eigenQTL6B.4 |
| 6B.5 | 1088849 - 1084601 | 54.79 - 55.31 | 650066114 - 654944436 | 3 | 0 |  |  |  |
| 6B.6 | 1108678 - 1064160 | 72.43 - 73.06 | 681223777 - 674881312 | 2 | 0 |  |  |  |
| 6B.7 | 2254957 - 1124447 | 78.46 - 83.75 | 684181124 - 695250301 | 24 | 0 |  |  | eigenQTL6B.6 |
| 7A.1 | 1080430 - 1405038 | 5.32 - 10.48 | 3583214 - 2169172 | 16 | 0 | *TaSST-A2* | Grain weight |  |
| 7A.2 | 1269206 - 1036713 | 14.97 - 19.77 | 17295305 - 27869431 | 12 | 0 |  |  |  |
| 7A.3 | 1087984 - 1093406 | 31.55 - 33.85 | 40289407 - 45767629 | 6 | 2 | *1-FEH-w3* | Drought tolerance | eigenQTL7A.1 |
| 7A.4 | 1102565 - 1228158 | 40.08 - 54.88 | 49595507 - 74375841 | 40 | 0 | *TaTEF-7A* | Grain weight |  |
| 7A.5 | 989082 - 1106305 | 57.97 - 58.55 | 80954519 - 83031889 | 3 | 1 |  |  |  |
| 7A.6 | 1229073 - 1159943 | 70.1 - 71.92 | 114795463 - 133346658 | 4 | 0 |  |  |  |
| 7A.7 | 1159943 - 2289574 | 74.44 - 76.09 | 133346594 - 135962604 | 7 | 0 |  |  | eigenQTL7A.3 |
| 7A.8 | 1088846 - 979642 | 79.35 - 81.76 | 242223641 - 533239512 | 3 | 0 |  |  |  |
| 7A.9 | 1372059 - 993154 | 89.00 - 90.22 | 616702357 - 621857049 | 4 | 0 |  |  |  |
| 7A.10 | 1075946 - 1091331 | 122.01 - 123.73 | 672978933 - 674423760 | 3 | 0 |  |  |  |
| 7A.11 | 1028228 - 2257142 | 135.24 | 691833209 - 693246439 | 5 | 0 |  |  |  |
| 7A.12 | 2288870 - 1124832 | 142.38 - 143.75 | 702331963 - 705275991 | 4 | 1 |  |  |  |
| 7A.13 | 994221 - 1101480 | 146.41 - 156.87 | 704012443 - 727245403 | 22 | 3 |  |  | eigenQTL7A.5 |
| 7B.1 | 1045660 - 1086594 | 12.5 - 14.96 | 7027175 - 13347233 | 6 | 1 |  |  |  |
| 7B.2 | 1073035 - 1128465 | 21.48 - 24.48 | 39435850 - 60598863 | 11 | 1 |  |  |  |
| 7B.3 | 2279028 - 1068405 | 43.76 - 49.78 | 206528084 - 479255213 | 17 | 0 |  |  | eigenQTL7B.1 |
| 7B.4 | 1030723 - 1100692 | 52.69 - 53.84 | 565143461 - 572331052 | 2 | 3 |  |  |  |
| 7B.5 | 1193598 - 1279301 | 57.08 - 59.75 | 582639470 - 594668252 | 23 | 1 |  |  |  |
| 7B.6 | 3064364 - 1096322 | 62.08 | 595788739 - 613941912 | 3 | 1 |  |  |  |
| 7B.7 | 2276691 - 2276847 | 73.51 - 77.78 | 622146287 - 640322150 | 9 | 2 |  |  | eigenQTL7B.2 |
| 7B.8 | 1039951 - 1096643 | 93.44 - 102.27 | 655173893 - 683337590 | 15 | 0 |  |  | eigenQTL7B.4 |
| 7B.9 | 1008216 - 1086253 | 110.99 - 112.18 | 691581021 - 694293591 | 19 | 8 |  |  | eigenQTL7B.5 |
| 7B.10 | 2276401 - 1102372 | 116.03 - 119.75 | 698840038 - 699203533 | 17 | 1 |  |  |  |
| 7B.11 | 1129098 - 1248567 | 125.33 - 129.67 | 705581006 - 710345552 | 21 | 1 | Psy-B1, Vrn-B3 | Phytoene synthase, vernalization | eigenQTL7B.6 |
